# Facilitators and Barriers to Digital Mental Health Interventions for Depression, Anxiety, and Stress in Adolescents and Young Adults: Scoping Review

**DOI:** 10.2196/62870

**Published:** 2025-03-24

**Authors:** Shimin Zhu, Yongyi Wang, Yuxi Hu

**Affiliations:** 1 Department of Applied Social Sciences Hong Kong Polytechnic University Hong Kong SAR China (Hong Kong); 2 Mental Health Research Centre The Hong Kong Polytechnic University Hong Kong SAR, China Hong Kong SAR China (Hong Kong)

**Keywords:** digital mental health interventions, adolescents, young adults, common mental disorders, thematic analysis, relative frequency of occurrence

## Abstract

**Background:**

Digital mental health interventions (DMHIs) offer unique strengths as emerging services with practical applications for adolescents and young adults (AYAs) experiencing depression, anxiety, and stress. Although promising, acceptance and participation in DMHIs vary across interventions, participants, and contexts. It is essential to delineate and synthesize the factors that promote or hinder DMHI use.

**Objective:**

This review aims to assess and synthesize the facilitators and barriers to accessing DMHIs for depression, anxiety, and stress in AYAs through a scoping review.

**Methods:**

A comprehensive search was conducted across multiple databases, including PubMed, Web of Science, PsycINFO, CNKI, OpenGrey, and APA PsycExtra, up to October 31, 2023. Articles examining facilitators and barriers to DMHIs among AYAs with disorders or symptoms of depression, anxiety, and stress were included. Data synthesis and analysis involved quality assessment, thematic analysis, and relative frequency meta-analysis.

**Results:**

A total of 27 records met the eligibility criteria, and 14 facilitators and 13 barriers were identified across the external, intervention, and individual levels. The relative frequency meta-analysis indicated that factors influencing AYAs’ use of DMHIs varied based on delivery modes. Among these factors, “quality and effect” emerged as the predominant theme—high quality and effect served as the primary facilitator, while low quality and effect acted as a barrier across both portable and nonportable devices, as well as single and multiple platforms.

**Conclusions:**

The uptake of DMHIs among AYAs is influenced by a complex interplay of facilitators and barriers, particularly those related to quality and effect. Our syntheses provide crucial guidance for intervention designers, emphasizing the importance of user-centered approaches that balance scientific rigor with engaging and adaptive features. Enhancing the alignment of DMHIs with adolescent needs can improve both adoption and real-world mental health impact.

**Trial Registration:**

PROSPERO CRD42023479880; https://www.crd.york.ac.uk/PROSPERO/view/CRD42023479880

## Introduction

Digital mental health interventions (DMHIs) have emerged and gained popularity as new tools and approaches for mental health services, driven by the development of the internet and mobile devices [1]. Nearly half of the global population with mental health needs lacks access to treatment or services, whereas more than half has access to smartphones and the internet. This contrast underscores the growing shift of mental health services toward mobile and digital health solutions [2]. DMHIs refer to intervention processes and programs that deliver mental health services through the web, technology, and mobile platforms [3,4]. Various forms of DMHIs have been found effective in promoting mental health, including smart digital apps, such as innovative communication apps designed to enhance communication and support young people experiencing low mood and suicidal thoughts [5]; web-based programs, such as a psychoeducational multimedia program for young people suffering from or at a high risk of depression, as well as their families, caregivers, friends, and professionals [6,7]; interactive games [8]; and email and text message communication [9,10].

DMHIs offer several advantages and have strong potential to become widely adopted service options, particularly for adolescents and young adults (AYAs). First, DMHIs align with the digital era and are well-suited to the new generation, who are familiar with, comfortable using, and frequent users of the web, digital media, and screen devices. According to the International Telecommunication Union’s Facts and Figures 2023, 79% of people aged 15-24 years use the internet globally [11]. Second, compared with face-to-face help-seeking or treatment, DMHIs can alleviate AYAs’ feelings of shame and effectively address concerns about stigmatization [12]. This was confirmed by young people’s feedback on a DMHI app, which suggested that discreet and easy-to-conceal apps helped mitigate the stigma associated with mental health problems [13]. Third, DMHIs are user-friendly. Some services adopt youth-friendly language and expressions while adjusting their layout to enhance usability [12]. Additionally, DMHIs offer flexibility, as they are not restricted by time, allowing users to engage with them according to their schedules [14]. Fourth, DMHIs provide high accessibility. Unlike traditional psychotherapy approaches, such as counseling, which require significant time for queuing and waiting, DMHIs have greatly improved AYAs’ access to mental health support through digital and online platforms [15]. At the same time, DMHIs can help mitigate the inaccessibility of mental health services caused by geographical remoteness or exceptional circumstances, such as lockdowns during infectious disease outbreaks [16,17]. Lastly, DMHIs offer scalability, as they have the potential to reach a wider audience due to their anonymity, accessibility, cost-effectiveness, and ability to provide timely feedback [4].

AYAs’ health, including mental health, plays a crucial role throughout the life course [18]. Health during this period is fundamental to their development, shaping long-term well-being and influencing the foundation of a healthy life for the next generation [18]. However, common mental disorders (CMDs) among adolescents are highly prevalent and contribute significantly to the burden of noncommunicable diseases [19-21]. CMDs encompass distress states characterized by anxiety, depression, and unexplained somatic symptoms [22]. According to data from the World Health Organization (WHO) and various regions, 1 in 7 individuals aged 10-19 years had a mental disorder in 2021, accounting for 13% of the global burden of disease in this age group [19]. Moreover, a 2023 survey in the United States found that nearly 90% of youth faced mental health challenges [23]. In the postpandemic era, CMDs present a growing challenge, highlighting the need for more accessible mental health services [24].

CMDs in AYAs require early and timely intervention; however, the uptake of mental health services remains inadequate [25-27]. The incidence of mental disorders has been reported to increase significantly after the age of 14 years [28]. Without timely intervention, these disorders can persist into adulthood, potentially impairing both physical and mental health and limiting opportunities for a fulfilling life [19]. Although mental health services and resources are currently available [18], a large proportion of AYAs do not access them [26]. Reasons for low service utilization are stigma, limited knowledge, low trust in the therapeutic relationship, high costs, accessibility issues, and other barriers [27], preventing those in need from receiving early interventions. DMHIs may serve as a viable alternative for youth requiring mental health services.

Although DMHIs offer many advantages [8,10,17], AYAs’ intention to use them and their usage patterns vary. For effectiveness, a web-based mental health intervention program for adolescents found that participants with high adherence (using the site for 30 minutes or more per week) reported significantly lower depression and stress levels, along with improved well-being [7]. Another study examining the effectiveness of a new computerized cognitive behavioral therapy program found that it led to meaningful improvements in participants’ depression levels [8]. Nevertheless, the usage, intention, engagement, and adherence to DMHIs remain relatively low [29]. For instance, a study in the United Kingdom examining young people’s attitudes toward computerized therapy found that only 25% expressed interest, another 25% were not interested, and half were unsure [30]. Additionally, several intervention studies have reported inadequate engagement and completion rates, with low user adherence (participants completing less than half of the intervention components) and high attrition rates (over 20%) [31]. Given the high demand for mental health services for CMDs among AYAs, it is essential to explore users’ perspectives on the factors that facilitate or hinder their use of DMHIs. Identifying these facilitators and barriers is crucial for the effective development and promotion of DMHIs.

Previous studies have rarely focused on the experiences, attitudes, or perceptions of AYAs regarding DMHIs as a primary research objective [32,33]. Much of the existing research has collected participants’ feedback only after evaluating a specific DMHI [17,33]. As a result, current findings on the facilitators and barriers to DMHI utilization are largely indirect and fragmented, highlighting the need for a more cohesive and comprehensive synthesis.

This study aims to conduct a scoping review to examine the existing literature on the facilitators and barriers to DMHI use among AYAs experiencing depression, anxiety, and stress. In this study, facilitators are defined as factors that enhance access to, usage of, or intention to use DMHIs, while barriers are factors that hinder use or reduce the intention to use them [34]. Synthesizing these facilitators and barriers will provide critical insights for promoting DMHIs to better address mental health needs.

## Methods

### Scoping Review Framework and Registration

This scoping review followed the Preferred Reporting Items for Systematic Reviews and Meta-Analyses Extension for Scoping Reviews (PRISMA-ScR) checklist to identify and map key characteristics, facilitators, barriers, and related themes in AYAs’ use of DMHIs (Multimedia Appendix 1) [35]. The review protocol was registered on PROSPERO (CRD42023479880).

### Search Strategy

The following major electronic databases were searched as of October 31, 2023: PubMed, Web of Science, PsycINFO, and CNKI. Although ScienceDirect was initially listed in our registered protocol, it was excluded from the final search due to significant content overlap with other databases. However, we ensured comprehensive coverage by searching additional relevant databases. Given that DMHIs are emerging technologies, no start date was set for the literature search to capture a broader range of publications. To supplement the search strategy, we manually retrieved bibliographies from relevant studies and included gray literature sources (OpenGrey and APA PsycExtra). The searches were restricted to English and Chinese. Further details are provided in Multimedia Appendix 2.

### Eligibility Criteria

This research included studies examining facilitators and barriers to DMHIs among AYAs with disorders or symptoms of depression, anxiety, and stress. Reviews, recommendations, comments, newspapers, letters, conference abstracts, and research from other stakeholders’ perspectives were used to enhance the understanding of the topic but were excluded from the analysis. The following exclusion criteria were applied: (1) studies involving participants with serious illnesses (eg, heart failure or trauma), as these conditions could impose greater limitations on their thoughts and behaviors; (2) studies with participants younger than 10 or older than 26 years; (3) studies on digital health services unrelated to mental health; (4) studies assessing only the effectiveness of DMHIs, participants’ attitudes, or willingness—without discussing influencing factors; and (5) studies with unavailable full texts due to access restrictions or incomplete records were excluded to ensure transparency and replicability of the review.

### Study Selection and Data Extraction

The literature screening process was conducted using EndNote 20 (Clarivate Plc), and Microsoft Excel 2020 was used to record the extracted data. Two independent researchers (YW and YH) reviewed the literature and extracted data. First, YW identified and removed duplicate records, which were then manually checked for accuracy by YH. Second, the researchers independently screened studies based on titles and abstracts, followed by a full-text review to determine eligibility. Third, data extraction was conducted independently by both researchers. Any discrepancies at each stage were discussed and resolved by 3 researchers (YW, YH, and SZ). The extracted data from each included study comprised 4 dimensions: (1) metadata and study context (eg, authors, year, study design, and sample size); (2) characteristics of the study population (eg, country or region, race, sample type, and basic demographics); (3) characteristics of DMHIs (eg, type, delivery mode, number of sessions, and inclusion of self-help or self-directed tools); and (4) facilitators and barriers influencing AYAs with CMDs in accessing DMHIs.

### Data Synthesis and Analysis

In line with the WHO’s 3-level digital health outcomes assessment framework [36], which includes the health system, provider, and client perspectives, this review adopted a thematic analysis framework comprising 3 levels: (1) external level, (2) intervention level, and (3) individual level.

Thematic analysis in this study followed a 6-phase process (Textbox 1) [37].

**Thematic analysis.**

1. **Familiarization with data**
  All researchers (SZ, YW, and YH) thoroughly read the literature, annotating key insights and recording analytical ideas for discussion.
2. **Initial coding**
  YW and YH independently conducted manual coding, identifying and categorizing relevant data segments on facilitators and barriers. Their coded results were compared and collated to ensure accuracy and comprehensiveness.
3. **Theme development**
  SZ, YW, and YH discussed and organized the codes into themes, subthemes, and levels, refining their relationships. YW then supplemented and structured the original data for clarity.
4. **Review of themes**
  YW reexamined the data extraction, subthemes, and thematic framework, engaging in discussions with SZ and YH until a consensus was reached.
5. **Definition and refinement**
  The essence of each theme and subtheme was defined. YW drafted the initial narrative, which was then reviewed and refined by SZ and YH for coherence, consistency, and minimal overlap.
6. **Final reporting**
  A structured and logical report was generated, presenting the thematic findings.

After identifying themes and subthemes, a relative frequency meta-analysis was conducted to assess the occurrence of each facilitator and barrier across different delivery modes: completely nonportable devices, portable devices, single-platform, and multiple-platform interventions. Studies that did not specify the delivery model were excluded. The analysis used the relative frequency of occurrence (RFO) with 95% CIs, performed using the metaprop function in R (R Foundation) [38].

### Quality Assessment

The quality of the included literature was assessed using the Mixed Methods Appraisal Tool (MMAT) [39]. This study applied the 2018 version, which was developed through a literature review of critical appraisal tools, user interviews, and electronic Delphi consultations with international experts. The MMAT evaluates 5 research categories: (1) qualitative studies, (2) randomized controlled trials, (3) nonrandomized studies, (4) quantitative descriptive studies, and (5) mixed methods studies. In addition to 2 general screening questions applicable to all study types, 5 specific criteria were established for each research category—qualitative, quantitative randomized controlled trials, quantitative nonrandomized studies, quantitative descriptive studies, and mixed methods studies [39].

## Results

### Search Selection

**Figure 1:**
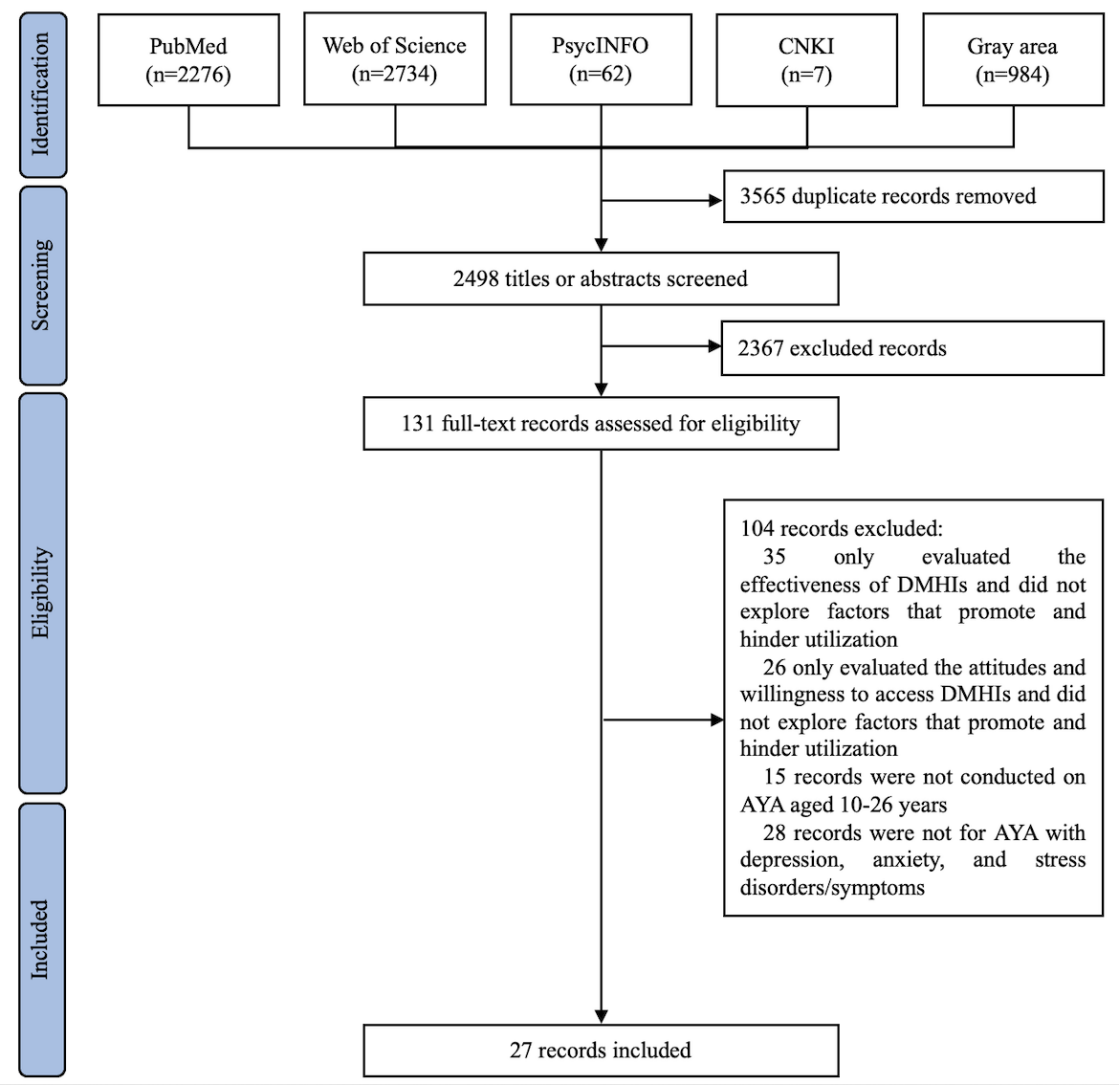
PRISMA-ScR (Preferred Reporting Items for Systematic Reviews and Meta-Analyses extension for Scoping Reviews) flow diagram. AYA: adolescents and young adult; DMHI: digital mental health intervention.

A total of 6063 records were retrieved from electronic databases and gray literature sources. After removing duplicates, 2498 records remained. Title and abstract screening identified 131 studies for full-text review, of which 27 met the inclusion and exclusion criteria and were included in data extraction, synthesis, and analysis (Figure 1; also see Multimedia Appendix 1).

### Description of Included Studies

Table 1 presents the characteristics of the included studies. Among the 27 studies, 7 (26%) were quantitative, 6 (22%) were qualitative, and 14 (52%) applied mixed methods. Geographically, 4 (15%) studies were conducted in England, 5 (19%) in the United States, 3 (11%) in New Zealand, 7 (26%) in Australia, 2 (7%) in Ireland, and 2 (7%) in China. Additionally, 1 (4%) study was conducted in each of Canada, Sweden, and South Africa, while 1 (4%) spanned both Chile and Colombia. Regarding participant recruitment channels, 5 (19%) studies recruited participants from health institutions, 4 (15%) from the community, 6 (22%) from schools, and 2 (7%) from youth centers and a survey, respectively. Additionally, 10 (37%) studies recruited participants from multiple sources, including health institutions, schools, communities, teams, and existing research studies. The DMHIs examined in the included studies can be broadly categorized into web/internet-based, computer-based, app-based, game-based, and other formats. Specifically, 13 (48%) studies delivered DMHIs via web/internet-based programs, 6 (22%) were app-based, and 2 (7%) were game-based. One study (4%) utilized a text messaging tool, while 1 (4%) implemented a chatbot. Several interventions employed hybrid delivery methods, such as text messaging combined with web-based programs (1/27, 4%), computer-based combined with web-based programs (2/27, 7%), and web-based combined with app-based interventions (1/27, 4%). Regarding conflicts-of-interest disclosures, 19 studies included a conflicts-of-interest component, with 7 studies [5,8,10,16,17,40,41] explicitly declaring potential or actual conflicts of interest.

Regarding the mental health symptoms and therapeutic approaches covered by the DMHIs in the included studies, 22 out of 27 (81%) aimed to improve depressive symptoms. Additionally, 14 studies addressed other mental health challenges, including anxiety, stress, worry, anger, and suicidal ideation. Notably, 3 studies [12,32,42] specifically targeted anxiety disorders. Among the 20 studies that explicitly stated a theoretical foundation for intervention development, 14 incorporated cognitive behavioral therapy, with 3 [8,15,30] utilizing computerized cognitive behavioral therapy. Furthermore, 2 studies [17,43] were based on peer support, 1 on behavioral activation [44], 1 on behavior change and resiliency [40], 1 on positive psychology [7], and 1 on mindfulness [45].

**Table 1:** Characteristics of articles included in this scoping review.

Study	Study design	Location	Race^a^	Sample type	Number of participants^a^	Participants’ characteristics^a^	Type and delivery mode	Sessions	Self-help/self-directed tool
Horgan et al [43]	Mixed methods	Ireland	98.3% White and 1.7% Asian or Asian Irish	School	118	Age: 18-24 years, mean 20.6 years, 64.4% males	A website; no specific mode of delivery	Not specific	No, peer support
Giovanelli et al [16]	Mixed methods	United States	64% White, 14% Asian, 14% Black, and 7% mixed race	Community and school	14 (interviews: 5)	Age: 15-18 years, mean 16 years	Appa Health, a smartphone app	Video sessions weekly	Not specific
Van Voorhees Benjamin et al [40]	Quantitative	United States	23% African American, 5% Hispanic, 6% Asian, and 4% other	Health institution	83	Age: 14-21 years, mean (SD) 17.4 (2.14) years, 56% females	An internet-based depression prevention program; no specific mode of delivery	Not specific	Not specific
Suffoletto et al [10]	Quantitative	United States	92.30% White, 1.92% Black, and 5.77% more than one	Health institution (primary care and mental health clinic)	52 at baseline (45 completed follow-ups)	Age (of the control group): mean (SD): 18.7 (0.48) years, 100% females; age (of the intervention group): mean (SD): 18.7 (0.42) years, 79% females	MoST-MH, an automated mobile support tool, delivered via mobile phones	Not specific	Yes
Goodyear-Smith et al [51]	Mixed methods	New Zealand	Not specific	School and community	30	Age: <25 years, 93% females	YouthCHAT, questions delivered on an e-tablet	Not specific	Yes
Gericke et al [33]	Qualitative	South Africa	77.78% White and 22.22% Black	School	9	Age: 17-20 years, mean (SD) 18.9 (1.2) years, 66.67% females	ICare, a transdiagnostic semiguided iCBT^b^ intervention; no specific mode of delivery	7	Not specific
Sweeney et al [14]	Quantitative	Australia	Not specific	Community	217	Age: 13-18 years, mean 16.98 years, 71.9% females	Online therapy; no specific mode of delivery	Not specific	Not specific
Sit et al [44]	Mixed methods	China	Not specific	School	38 (interviews: 6)	Age: 18-25 years	Step-by-Step, a mobile app, delivered via smartphones or laptops	5	Yes
Thabrew et al [5]	Mixed methods	New Zealand	15% Māori, 65% New Zealand European, 15% Asian, and 4% Middle Eastern, Latin American, and African	Health institution and community	Quantitative: 26 young people; qualitative: 13 young people	Age (of young people): 16-25 years, mean: 17.7 years, 65% females	“Village,” a digital communication app, delivered via smartphones	Not specific	Not sure
Lilja et al [32]	Mixed methods	Sweden	Not specific	Health institution	14	Age: 13-18 years, 93% females	An iCBT program (Anxiety Help for Adolescents, a guided internet-delivered self-help treatment program); no specific mode of delivery	Not specific	Yes
Monshat et al [45]	Qualitative	Australia	Not specific	Community	13	Age: 16-26 years, mean: 22 years, 60% females	Online mindfulness training program; no specific mode of delivery	Not specific	Not specific
Kruzan et al [47]	Qualitative	United States	56% White, 10% Asian, 8% Black or African American, 2% American Indian or Alaskan Native, 12% more than one race, and 14% not reported	Health institution	50	Age: 18-25 years, 76% females	Online screening, delivered via mobile phones	The first asynchronous remote community group: 6; the second asynchronous remote community group: 8	Yes
Kuosmanen et al [15]	Mixed methods	Ireland	Not specific	Youth centers	40	Age: 15-20 years	SPARX-R, a computerized mental health game	Not specific	Yes
Dingwall et al [49]	Mixed methods	Australia	Not specific	Community	33 at baseline (30 completed the 4-week follow-ups)	Age (of 30 young people): 12-18 years, mean (SD): 14.0 (1.55) years, 43.33% females	AIMhi-Y app; smartphone based	4	Not specific
Clark et al [42]	Qualitative	Australia	Not specific	Health institution, research institution, community, and school	29	Age: 12-18 years, mean: 15.17 years	Computerized mental health help‐seeking	Not specific	Not specific
Wozney et al [12]	Mixed methods	Canada	Not specific	Community	Cycle 1: 4 young people; cycle 2: 4 young people	Age (of young people): <20 (the age range for selecting participants was 15-24 years old), 50% females	Breathe, an internet-based cognitive behavioral therapy program; delivered via computers, phones, and emails	2	Yes
Birrell et al [17]	Mixed methods	Australia	Not specific	School	166	Age, mean (SD): 15.3 (0.41) years	Mind your Mate, a mobile app; delivered via smartphones	Not specific	Yes
Stallard et al [30]	Mixed methods	Australia	Not specific	Health institution	37	Age: 8-17 years, mean 14.5 years	Computerized therapy	Not specific	Not specific
Kornfield et al [50]	Qualitative	United States	54.84% White, 16.13% more than 1 race, 12.90% Black or African American, 9.68% Asian, and 6.45% not reported	Survey	Discussion group: 22; co-design workshops: 9	Age: 18-25 years	Automated text messaging tool; delivered via mobile phones	Not specific	Yes
Grist et al [53]	Quantitative	England	Not specific	School	775	Age: 11-16 years	Internet and smartphone/tablet apps	Not specific	Yes
Bevan Jones et al [6]	Qualitative	England	Not specific	Health institution and study	Interviews: 4 young people; focus groups: 29 young people in 3 groups	Age (of young people interviewed): 13-18 years, 75% females; age (of young people in focus groups): 13-19 years, 68.97% females	MoodHwb, a web-based program; delivered via tablets or laptops	Not specific	Yes
Bevan Jones et al [46]	Mixed methods	England	95% White (young people interviewed)	Health institution, school, team, and study	Quantitative: 43 young people at baseline (36 completed the follow-ups); qualitative: 19 young people	Age (of young people at baseline): 13-23 years, mean (SD): 16.3 (2.36) years, 79% females; age (of young people interviewed): 14-19 years, mean (SD): 16.5 (1.78) years, 74% females	MoodHwb, a multiplatform program	Not specific	Yes
Merry et al [8]	Quantitative	New Zealand	58.5%-60.2% New Zealand, 22.6%-25.5% European Māori, 7.5%-8.5% Pacific people, 4.3%-8.6% Asian, and 1.1%-3.2% other	Health institution and school	Baseline: 187; postintervention: 170; 3-month follow-up: 168	Age: 12-19 years	Computerized cognitive behavioral therapy intervention (SPARX); a game	7 modules	Yes
Martínez et al [41]	Mixed methods	Chile and Colombia	Not specific	School	199	Age, mean (SD): 14.8 (1.0) years, 53.27% females	Take Care of Your Mood, an internet-based program for prevention and early intervention; delivered via computers or smartphones	Not specific	Not specific
Manicavasagar et al [7]	Mixed methods	Australia	Not specific	School and community	235	Age: 12-18 years	A web-based positive psychology program; delivered via computers	Not specific	Yes
Sawrikar et al [48]	Quantitative	England	Not specific	Community and school	248	Age: 17-25 years, mean (SD): 23.31 (1.91) years, 40.7% females	Digital mental health interventions; delivered via the internet or on a smartphone	Not specific	Not specific
He et al [52]	Mixed methods	China	92.57% Han	Community and school	148	Age, mean (SD): 18.78 (0.88) years	Chatbot, a software program with artificial intelligence; delivered via WeChat platform, e-book, etc	25.54 sessions on average	Yes

^a^The “Race,” “Number of participants,” and “Participants’ characteristics” columns only present data on adolescents and young adults and exclude relevant data from other stakeholders (eg, parents, clinicians, and school staff).

^b^iCBT: internet-based cognitive behavioral therapy.

### Quality Assessment

The quality assessment (Table 2) indicated that qualitative studies and quantitative randomized controlled trials were generally of high quality. However, all quantitative descriptive studies exhibited varying degrees of sample representativeness.

**Table 2:** Quality assessment of articles included by the Mixed Methods Appraisal Tool, 2018 version.

	Qualitative					Quantitative randomized controlled trials					Quantitative nonrandomized					Quantitative descriptive					Mixed methods				
	1	2	3	4	5	1	2	3	4	5	1	2	3	4	5	1	2	3	4	5	1	2	3	4	5
Horgan et al [43]	Y^a^	Y	N^b^	C^c^	Y	—^d^	—	—	—	—	—	—	—	—	—	Y	N	Y	N	C	Y	Y	Y	C	N
Giovanelli et al [16]	Y	Y	Y	Y	Y	—	—	—	—	—	—	—	—	—	—	Y	N	Y	Y	Y	Y	Y	Y	Y	N
Van Voorhees Benjamin et al [40]	—	—	—	—	—	Y	C	Y	Y	N	—	—	—	—	—	—	—	—	—	—	—	—	—	—	—
Suffoletto et al [10]	—	—	—	—	—	Y	Y	Y	Y	Y	—	—	—	—	—	—	—	—	—	—	—	—	—	—	—
Goodyear-Smith et al [51]	Y	Y	Y	Y	Y	—	—	—	—	—	—	—	—	—	—	C	N	Y	C	Y	Y	Y	Y	Y	N
Gericke et al [33]	Y	Y	Y	Y	Y	—	—	—	—	—	—	—	—	—	—	—	—	—	—	—	—	—	—	—	—
Sweeney et al [14]	—	—	—	—	—	—	—	—	—	—	—	—	—	—	—	C	N	Y	C	Y	—	—	—	—	—
Sit et al [44]	Y	Y	Y	C	Y	—	—	—	—	—	N	Y	Y	C	Y	—	—	—	—	—	Y	Y	Y	Y	C
Thabrew et al [5]	Y	Y	Y	Y	Y	—	—	—	—	—	—	—	—	—	—	C	C	Y	N	Y	Y	Y	Y	Y	N
Lilja et al [32]	Y	Y	Y	Y	Y	—	—	—	—	—	—	—	—	—	—	C	N	Y	Y	Y	Y	Y	Y	Y	N
Monshat et al [45]	Y	Y	Y	Y	Y	—	—	—	—	—	—	—	—	—	—	—	—	—	—	—	—	—	—	—	—
Kruzan et al [47]	Y	Y	C	Y	Y	—	—	—	—	—	—	—	—	—	—	—	—	—	—	—	—	—	—	—	—
Kuosmanen et al [15]	Y	Y	C	Y	Y	—	—	—	—	—	—	—	—	—	—	C	N	Y	C	Y	Y	Y	Y	Y	N
Dingwall et al [49]	Y	C	Y	Y	Y	—	—	—	—	—	N	Y	Y	Y	Y	—	—	—	—	—	Y	Y	Y	C	N
Clark et al [42]	Y	Y	Y	C	Y	—	—	—	—	—	—	—	—	—	—	—	—	—	—	—	—	—	—	—	—
Wozney et al [12]	Y	Y	Y	Y	Y	—	—	—	—	—	—	—	—	—	—	Y	N	Y	Y	Y	Y	Y	Y	Y	N
Birrell et al [17]	Y	Y	C	C	Y	Y	N	Y	N	N	—	—	—	—	—	—	—	—	—	—	Y	Y	Y	Y	N
Stallard et al [30]	Y	Y	Y	Y	Y	—	—	—	—	—	—	—	—	—	—	C	N	Y	Y	Y	Y	Y	Y	Y	N
Kornfield et al [50]	Y	Y	Y	C	Y	—	—	—	—	—	—	—	—	—	—	—	—	—	—	—	—	—	—	—	—
Grist et al [53]	—	—	—	—	—	—	—	—	—	—	—	—	—	—	—	C	N	Y	C	Y	—	—	—	—	—
Bevan Jones et al [6]	Y	Y	Y	Y	Y	—	—	—	—	—	—	—	—	—	—	—	—	—	—	—	—	—	—	—	—
Bevan Jones et al [46]	Y	Y	Y	Y	Y	—	—	—	—	—	—	—	—	—	—	Y	C	Y	C	Y	Y	Y	Y	Y	C
Merry et al [8]	—	—	—	—	—	Y	Y	Y	Y	Y	—	—	—	—	—	—	—	—	—	—	—	—	—	—	—
Martínez et al [41]	Y	Y	Y	C	Y	—	—	—	—	—	—	—	—	—	—	Y	C	Y	Y	Y	Y	Y	Y	Y	C
Manicavasagar et al [7]	Y	C	C	Y	Y	Y	Y	Y	Y	Y	—	—	—	—	—	—	—	—	—	—	Y	Y	Y	Y	C
Sawrikar et al [48]	—	—	—	—	—	—	—	—	—	—	—	—	—	—	—	Y	N	Y	C	Y	—	—	—	—	—
He et al [52]	Y	Y	C	C	Y	Y	N	Y	Y	N	—	—	—	—	—	—	—	—	—	—	Y	Y	Y	Y	N

^a^Y: Yes.

^b^N: No.

^c^C: Cannot tell.

^d^—: Not applicable.

### Thematic Synthesis

#### Overview

**Figure 2:**
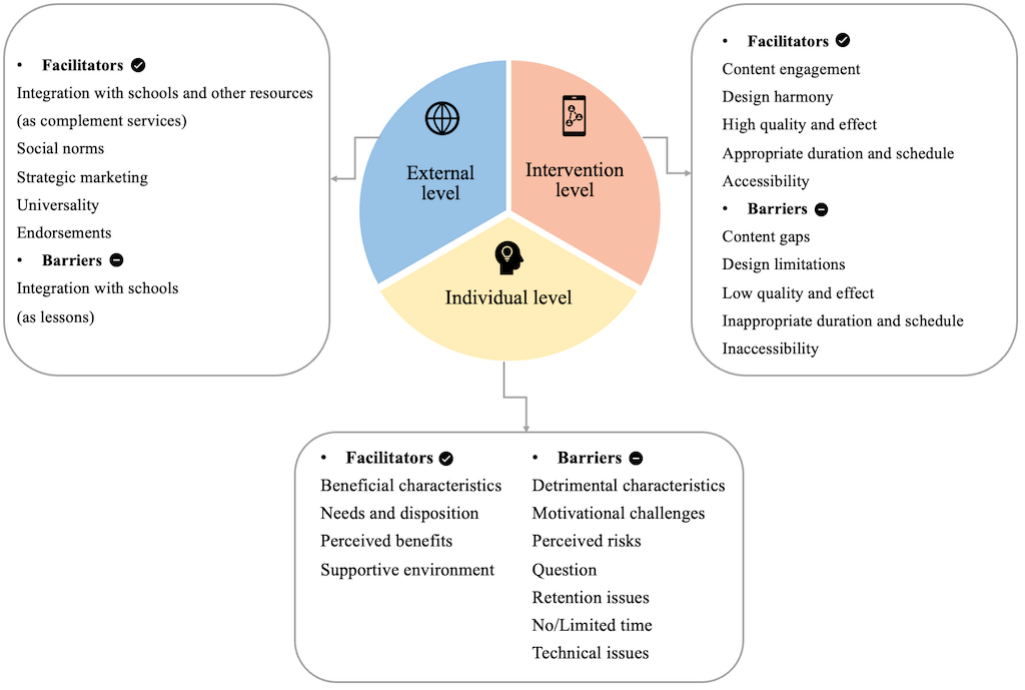
Themes of facilitators and barriers under the three-level framework.

The thematic analysis identified facilitators and barriers across 3 levels: external, intervention, and individual. Figure 2 presents the themes, while Multimedia Appendix 3 provides an overview of the subthemes and corresponding examples. A detailed breakdown of all themes, subthemes, and supporting extracts from the original studies is available in Multimedia Appendix 4.

#### Facilitators: External Level

It is recommended that the adoption and implementation of DMHIs be integrated with other services [46,47], with schools serving as a key environment for AYAs [6]. Social norms, particularly subjective norms, exert pressure based on the expectations of AYAs [48]. Additionally, the strategic marketing and broad accessibility of DMHIs play a crucial role in shaping user motivation [6,45,47], stigma, and social isolation [15]. Endorsements from friends, peers, care providers, professionals, and reputable programs can further enhance assessment and engagement [10,47].

#### Facilitators: Intervention Level

Literature suggests that key factors promoting widespread acceptance of DMHIs are closely tied to program features, including diverse information types, personalization, comprehensive support, and communication sessions for feedback and sharing [6,14,16,32,33,40,41,44-47,49,50]. Testimonials and entertainment elements were recommended and well-received, alongside retention strategies such as notifications, messages, and incentives [15,41,44,46,49]. Evidence indicates that multimedia delivery, a professional and polished design, co-design, character inclusion, personalization, diverse presentation formats, and appropriate language are critical for enhancing product quality and user appeal [6,10,12,15,16,32,33,41,42,44-47,49,50]. Many participants were surprised by the high quality of the programs and appreciated their overall design and feel [44,49]. Concrete benefits, such as the ability to track progress, were highly praised [14]. Factors contributing to sustained acceptance included an engaging and enjoyable experience, positive emotional responses, ease of interaction and use, high relevance, and appealing visual design [5-7,10,12,14-16,40,41,43-46,48,49,51,52]. The importance of appropriate durations and flexible schedules was also emphasized, ensuring convenience and adaptability to users’ needs [6,12,16,41,44-46]. High accessibility further expanded user reach, allowing engagement at any time and from remote locations [6,14,16,41]. Additionally, many participants identified free or low-cost access as a crucial factor in promoting DMHI use [14,46,47].

#### Facilitators: Individual Level

Female participants reported significantly higher perceived helpfulness of the intervention [14]. Individuals with more severe psychological symptoms, greater knowledge, and prior experience with online therapies demonstrated stronger motivation to seek help and enroll [14,42,48]. AYAs were more likely to engage with DMHIs if they had genuine mental health needs [6,33,43,50,53], preferred solitude or home-based interventions, valued anonymity and autonomy [5,30,33,45], and held positive attitudes toward mental health issues and technology [14,48]. Frequently cited perceived benefits of DMHI use included support during difficult periods, perceived usefulness, privacy, and improved time management [33,49,51]. Additionally, a supportive environment—encompassing both technical infrastructure and interpersonal influences—played a crucial role in facilitating engagement [6,47].

#### Barriers: External Level

Several studies presented an opposing perspective, suggesting that integrating DMHIs with schools could reduce their appeal [46] and lower participation rates [40,54]. Students might find the association with school tasks frustrating [46], and school policies restricting the use of electronic mobile devices could further limit engagement [54].

#### Barriers: Intervention Level

Some content components faced criticism. Religious overtones, particularly elements related to meditation and spirituality, were not well received [45]. Additionally, the absence of therapist support and direct human interaction led to frustration and disappointment among users [14,33,44]. Issues such as robotic responses, inappropriate multimedia elements, and poor language choices in design were perceived as barriers, causing confusion and disengagement [5,45,46]. Opinions on personalization varied: while excessive customization [15,44] could create confusion, difficulty, and cognitive burden [5,15], insufficient personalization resulted in overly generalized content [14]. Regarding quality and effectiveness, unattractive design, lack of relevance, and unsatisfactory user experiences were significant hindering factors [7,14,15,51]. Repetitiveness was particularly noted for diminishing the initial appeal of a DMHI website over time [7]. Inappropriate durations and scheduling further contributed to negative experiences [15,44,45,51]. Additionally, inaccessibility—primarily due to technical issues and financial constraints—was identified as an objective barrier to engagement [5,16,47,49,52].

#### Barriers: Individual Level

Physical illness was identified as a potential reason preventing participants from attending to or completing DMHIs on time [8]. Additional barriers included a lack of confidence and necessary connections to engage with internet-based therapies [17,32]. A lack of motivation was cited as a reason for not even downloading the app [17]. Participants who preferred face-to-face support or had reservations about human-like messaging systems expressed hesitation toward DMHIs [30,50,53]. Perceived risks were particularly emphasized by many AYAs, including concerns about privacy, security, and credibility, as well as fears of stigma and cyberbullying [14,42]. Nonuse or noncompletion of DMHIs was often attributed to doubts regarding their helpfulness, validity, and usefulness, as well as low priority, low interest, and a lack of persistence [5,8,14,17,33,43,45,49,53]. Additionally, limited time and technical issues remained significant barriers to engagement [7,8,14,17,33,49].

### Relative Frequency Meta-Analysis

**Figure 3:**
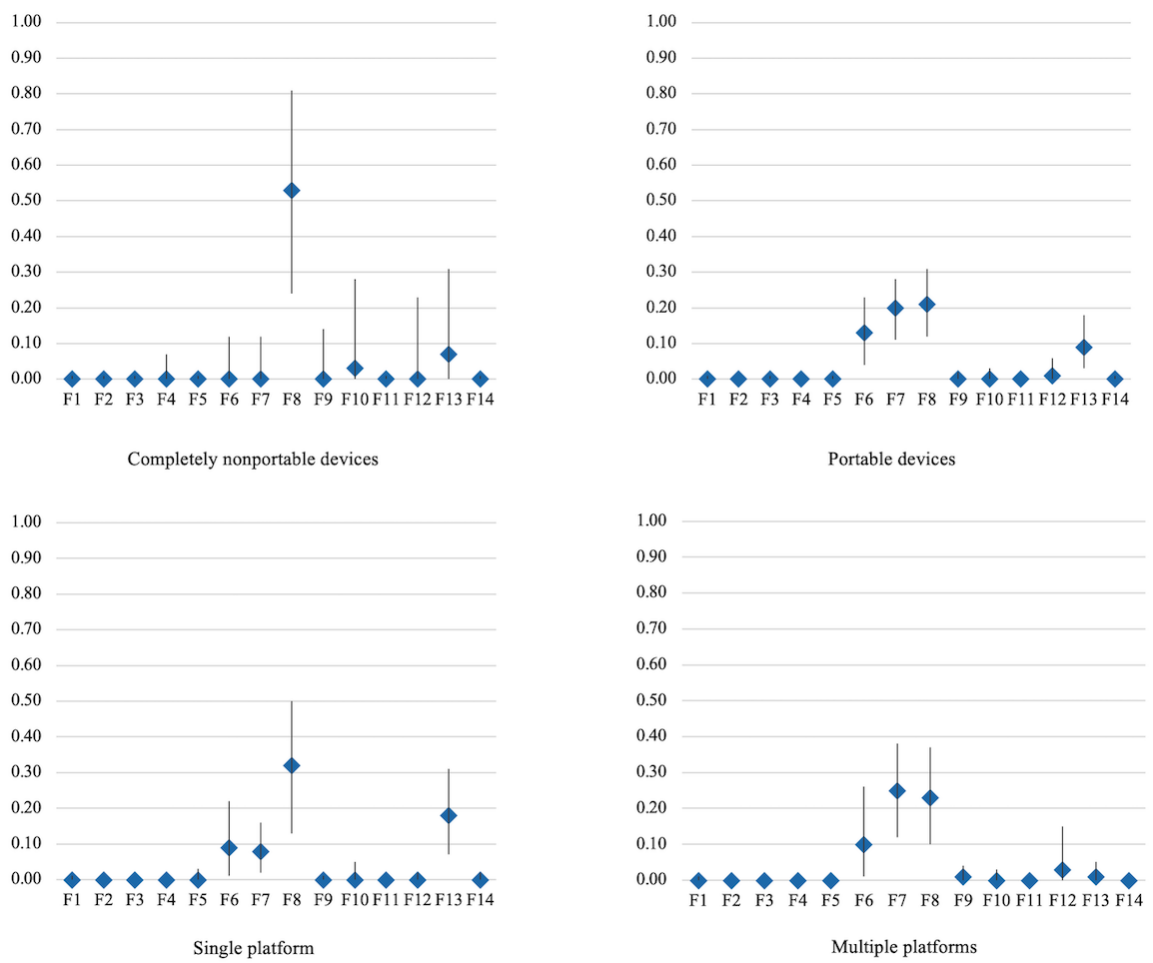
Relative frequency of occurrence of facilitators in the 4 delivery modes.

**Figure 4:**
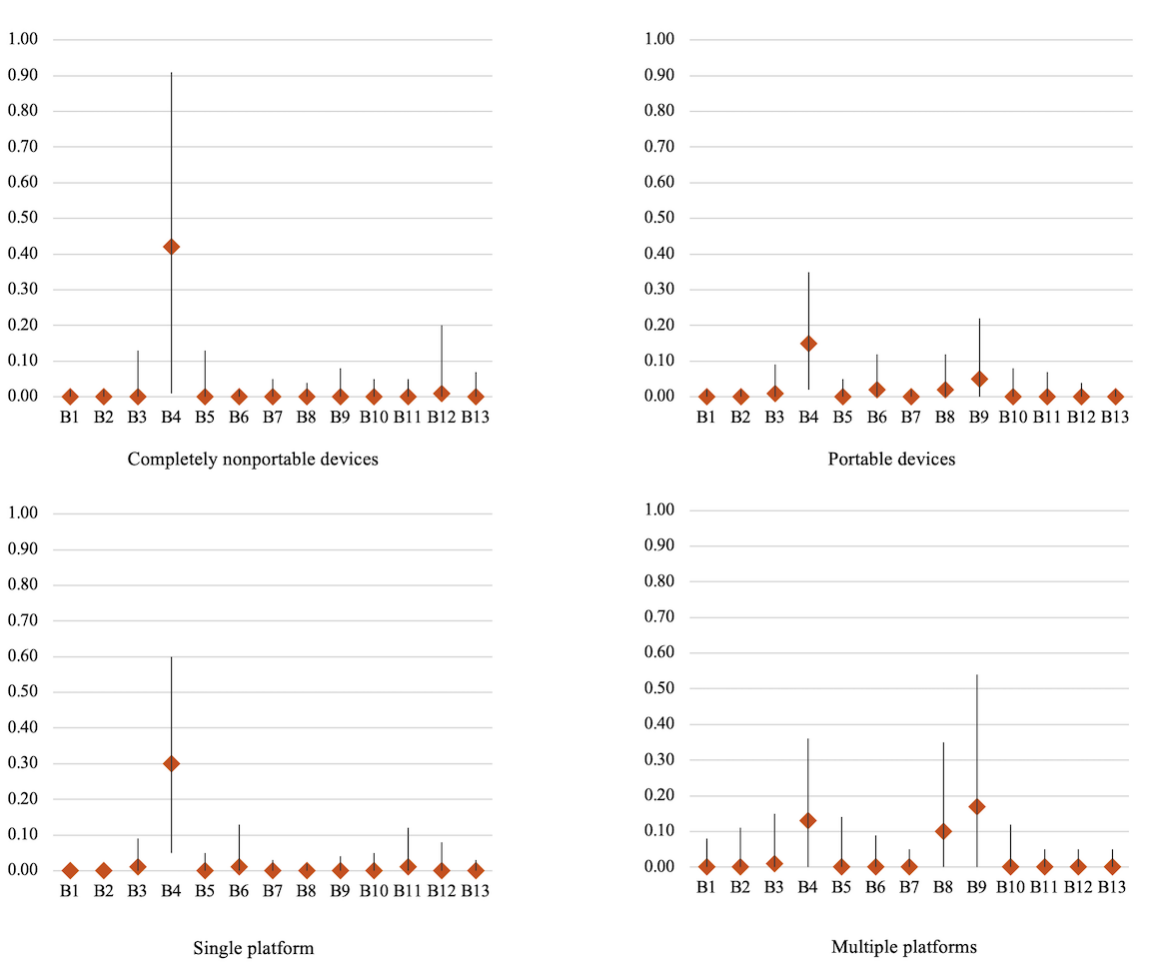
Relative frequency of occurrence of barriers in the 4 delivery modes.

Based on the generated themes, the predominant facilitators and barriers varied across different delivery modes, as shown in Figures 3 and 4. For completely nonportable devices, quality and effect emerged as the most relevant facilitator (RFO=53%, 95% CI 0.24-0.81) and barrier (RFO=42%, 95% CI 0.01-0.91) to DMHI use. By contrast, for portable devices, the primary facilitators included high quality and effect (RFO=21%, 95% CI 0.12-0.31), design harmony (RFO=20%, 95% CI 0.11-0.28), and content engagement (RFO=13%, 95% CI 0.04-0.23), while the dominant barrier was low quality and effect (RFO=15%, 95% CI 0.02-0.35). For single-platform DMHIs, usage was primarily associated with 2 key facilitators: high quality and effect (RFO=32%, 95% CI 0.13-0.50) and perceived benefits (RFO=18%, 95% CI 0.07-0.31). The predominant barrier was low quality and effect (RFO=30%, 95% CI 0.05-0.60). Regarding DMHIs available across multiple platforms, the most influential facilitators were design harmony (RFO=25%, 95% CI 0.12-0.38), high quality and effect (RFO=23%, 95% CI 0.10-0.37), and content engagement (RFO=10%, 95% CI 0.01-0.26). The primary barriers were perceived risks (RFO=17%, 95% CI 0-0.54) and low quality and effect (RFO=13%, 95% CI 0-0.36). Further details are provided in Multimedia Appendix 5.

## Discussion

### Principal Findings

This review systematically identified facilitators and barriers to AYAs’ access to DMHIs by analyzing quantitative, qualitative, and mixed methods literature using a 6-step thematic analysis approach. Following WHO guidelines, a 3-level theoretical framework was used to categorize these influential factors. Fourteen facilitators were identified, including integration with schools and other resources, social norms, and strategic marketing. Conversely, 13 barriers were recognized, such as integration with schools, content gaps, and design limitations.

The DMHIs examined in our review primarily targeted depression and were largely based on cognitive behavioral therapy. However, their development and application vary widely. Some studies have shown that DMHIs incorporating mindfulness training and peer support can effectively alleviate psychological challenges, including anxiety, stress, and resilience [43,45]. These findings underscore the potential of diverse DMHI approaches in mental health support and contribute to the growing digital landscape of psychological interventions.

During the RFO analysis, we focused specifically on digital modes of intervention, excluding studies that did not specify their delivery modes. Research supports the notion that different platform types may lead to variations in functionality, usage, and performance [55]. In our study, the theme of “quality and effect” emerged as the predominant facilitator and barrier across all DMHI delivery modes. This finding suggests that the perceived quality and effectiveness of DMHIs were central concerns for both participants and researchers. High-quality interventions that demonstrate positive outcomes are more likely to enhance user engagement, support sustained use, and encourage recommendations to others [51]. Conversely, interventions that fail to meet user expectations or inadequately address their needs may result in disengagement or abandonment [7].

When examining the subthemes, the most common and consistent points across the 3 levels were summarized. Participants showed greater willingness to engage when DMHIs were integrated with other resources [46,47], possibly due to increased accessibility and a reduced risk of judgment or embarrassment [56]. Previous research and professionals particularly valued the benefits of combining DMHIs with traditional medical resources and incorporating them into daily life [54,57,58]. The use of multimedia, also referred to as “aesthetics” or “visual assets,” was widely accepted and recommended for its ability to attract attention, generate interest, facilitate understanding, and enhance usability and satisfaction [12,59,60]. Perceived helpfulness or usefulness aligns with the Technology Acceptance Model, as it influences participants’ attitudes toward use, thereby shaping behavioral intentions and actual usage behaviors [61]. Negative emotional experiences led to reduced engagement among AYAs [15], with certain emotions, such as frustration and nervousness, contributing to physical and mental stress [62]. Privacy, security, and credibility concerns remained common barriers in DMHIs, similar to traditional psychotherapy. Despite technological advances and digitization, these remote services pose unique and heightened privacy risks to clients [54,63].

Furthermore, 2 factors—DMHI integration with schools and personalization—can serve as both facilitators and barriers. On the one hand, integrating DMHIs into school settings may significantly enhance accessibility to psychological support by streamlining delivery to students and potentially addressing gaps in resources and teacher training [46]. Embedding mental health interventions within the school environment allows students to access support without requiring external referrals or additional logistical arrangements. On the other hand, integrating DMHIs with schools may make them less appealing to students and could evoke negative feelings [45,46]. Additionally, strict school regulations on smart device usage may limit students’ ability to engage freely with DMHIs [54], potentially reducing their effectiveness by restricting flexible and convenient access to these digital tools. Regarding personalization, participants frequently highlighted its advantages, such as greater flexibility, tailored monitoring experiences, feedback, and additional benefits [44,45]. However, some participants viewed personalization negatively, as it often required additional actions and steps, adding to their burden and inconvenience [15]. Balancing the positive and negative aspects of these 2 factors requires strategies that maximize benefits while minimizing drawbacks. Suggested approaches include offering opt-in participation and flexible access options, allowing students to decide whether to engage and choose between on-campus or off-campus access to school-integrated DMHIs to enhance their sense of control and reduce stress. Additionally, making personalization optional by preconfiguring basic settings can improve ease of use, enabling users to engage with personalized features only to the extent they require.

Although many randomized controlled trials indicated that DMHIs were satisfying [8,10], they were not considered a substitute for traditional mental health services but rather an augmentation, potentially expanding accessibility and enhancing engagement [56,64,65]. From users’ perspectives, the most intuitive feedback was their preference for face-to-face communication, along with concerns and dissatisfaction regarding privacy issues and the mechanical, fixed responses of DMHIs [5,14,66]. However, this does not imply that users are inclined to reject DMHIs; rather, they support DMHIs as a supplementary form of care alongside traditional mental health services [65]. From a scientific research and development perspective, a hybrid model integrating DMHIs with traditional mental health services can maximize the advantages of both, such as addressing low retention rates in DMHIs and assisting mental health professionals in providing follow-up care [67,68]. Consequently, the notion that DMHIs can serve as a “digital glue” to enhance user engagement in mental health services is more widely accepted, as it facilitates a seamless transition between digital and nondigital services [56]. Therefore, supporting hybrid digital and traditional mental health services should be the central focus for the future development and implementation of DMHIs.

An effective design process is crucial for the success of DMHIs in the mental health field, with a particular emphasis on human-centered or user-centered design [69,70]. Developers and designers must adopt this approach to refine and enhance DMHIs, ensuring they align with users’ needs. Future research should focus on identifying the key components among the myriad and complex facilitators and barriers, sharpening the design process, and exploring which types of DMHIs are best suited for different mental health conditions.

Additionally, basic user characteristics may serve as unique influencing factors. Current findings suggest that women tend to spend more time using mental health apps [71], whereas men show greater interest in simulation game-based DMHIs [72]. Limited research has explored differences in DMHI usage based on race and ethnicity, though some evidence indicates that people of color may face barriers to accessing culturally responsive care in DMHIs [3]. Further systematic exploration and synthesis are needed to identify specific cultural factors that facilitate or hinder DMHI uptake among AYAs.

This scoping review summarized the facilitators and barriers to DMHIs for AYAs with depression, anxiety, and stress, categorizing them in a structured manner. By synthesizing the literature, the review offers insights for future intervention service designers and therapists, supporting the translation of DMHIs from research to practice—an essential step for advancing mental and public health. However, our review has some limitations. First, due to our inclusion and exclusion criteria, the quality of the included studies varies, with some not meeting high-quality standards. Second, the participants’ characteristics and age ranges in the reviewed studies were constrained by our exclusion criteria. Factors that facilitate or hinder adults or older adults with CMDs from engaging with DMHIs may similarly affect AYAs. Third, we excluded studies published in languages other than English and Chinese, which may have led to the omission of culturally specific factors. Another potential limitation is the search strategy. While we used keyword searches across multiple databases, this approach may have been less comprehensive than using built-in search structures (eg, MeSH terms in PubMed), potentially resulting in the omission of relevant studies. Finally, we acknowledge that the frequency of an individual factor does not necessarily indicate its significance, underscoring the need for follow-up studies to clarify its importance.

### Conclusion

This scoping review systematically searched, screened, and synthesized the literature on facilitators and barriers to DMHIs for AYAs with depression, anxiety, and stress. Through thematic synthesis, we identified a series of themes and subthemes at the external, intervention, and individual levels, highlighting key factors influencing the use and adoption of DMHIs. By consolidating these factors, this review provides insights that can inform the design and implementation of more effective DMHIs for AYAs.

## Data Availability

The data sets generated or analyzed during this study are available from the corresponding author upon reasonable request.

## Appendix

Multimedia Appendix 1 PRISMA-ScR checklist.

Multimedia Appendix 2 Literature search strategy.

Multimedia Appendix 3 Themes, subthemes definitions, and related examples.

Multimedia Appendix 4 Facilitators and barriers.

Multimedia Appendix 5 Metaprop results.

## Abbreviations

AYA: adolescents and young adult
CMDs: common mental disorders
DMHI: digital mental health intervention
MMAT: Mixed Methods Appraisal Tool
PRISMA-ScR: Preferred Reporting Items for Systematic Reviews and Meta-Analyses Extension for Scoping Reviews
RFO: relative frequency of occurrence
WHO: World Health Organization
