# Supplementary material for: Facilitators and Barriers to Digital Mental Health Interventions for Depression, Anxiety, and Stress in Adolescents and Young Adults: Scoping Review
Source: J Med Internet Res. 2025 Mar 24;27:e62870. doi: 10.2196/62870 (PMC11988281; doi:10.2196/62870)
Supplement: Multimedia Appendix 2 [file jmir_v27i1e62870_app2.docx]

LITERATURE SEARCH STRATEGY

| Databases | Limiters | Total | Query |
| --- | --- | --- | --- |
| PubMed | / | 2276 | (digital mental health interventions) AND (teen OR children OR students OR youth OR adolescents) AND (depression OR anxiety OR stress); (digital mental health service) AND (teen OR children OR students OR youth OR adolescents) AND (depression OR anxiety OR stress); (digital mental health technology) AND (teen OR children OR students OR youth OR adolescents) AND (depression OR anxiety OR stress); (digital mental health apps) AND (teen OR children OR students OR youth OR adolescents) AND (depression OR anxiety OR stress); (digital mental health program) AND (teen OR children OR students OR youth OR adolescents) AND (depression OR anxiety OR stress); (digital mental health system) AND (teen OR children OR students OR youth OR adolescents) AND (depression OR anxiety OR stress); (digital mental health care) AND (teen OR children OR students OR youth OR adolescents) AND (depression OR anxiety OR stress) |
| Web of Science | / | 2734 | (digital mental health interventions) AND (teen OR children OR students OR youth OR adolescents) AND (depression OR anxiety OR stress); (digital mental health service) AND (teen OR children OR students OR youth OR adolescents) AND (depression OR anxiety OR stress); (digital mental health technology) AND (teen OR children OR students OR youth OR adolescents) AND (depression OR anxiety OR stress); (digital mental health apps) AND (teen OR children OR students OR youth OR adolescents) AND (depression OR anxiety OR stress); (digital mental health program) AND (teen OR children OR students OR youth OR adolescents) AND (depression OR anxiety OR stress); (digital mental health system) AND (teen OR children OR students OR youth OR adolescents) AND (depression OR anxiety OR stress); (digital mental health care) AND (teen OR children OR students OR youth OR adolescents) AND (depression OR anxiety OR stress) |
| PsycINFO | / | 62 | (digital mental health interventions) AND (teen OR children OR students OR youth OR adolescents) AND (depression OR anxiety OR stress); (digital mental health service) AND (teen OR children OR students OR youth OR adolescents) AND (depression OR anxiety OR stress); (digital mental health technology) AND (teen OR children OR students OR youth OR adolescents) AND (depression OR anxiety OR stress); (digital mental health apps) AND (teen OR children OR students OR youth OR adolescents) AND (depression OR anxiety OR stress); (digital mental health program) AND (teen OR children OR students OR youth OR adolescents) AND (depression OR anxiety OR stress); (digital mental health system) AND (teen OR children OR students OR youth OR adolescents) AND (depression OR anxiety OR stress); (digital mental health care) AND (teen OR children OR students OR youth OR adolescents) AND (depression OR anxiety OR stress) |
| CNKI | / | 7 | (digital mental health interventions) AND (teen OR children OR students OR youth OR adolescents) AND (depression OR anxiety OR stress); (digital mental health service) AND (teen OR children OR students OR youth OR adolescents) AND (depression OR anxiety OR stress); (digital mental health technology) AND (teen OR children OR students OR youth OR adolescents) AND (depression OR anxiety OR stress); (digital mental health apps) AND (teen OR children OR students OR youth OR adolescents) AND (depression OR anxiety OR stress); (digital mental health program) AND (teen OR children OR students OR youth OR adolescents) AND (depression OR anxiety OR stress); (digital mental health system) AND (teen OR children OR students OR youth OR adolescents) AND (depression OR anxiety OR stress); (digital mental health care) AND (teen OR children OR students OR youth OR adolescents) AND (depression OR anxiety OR stress) |
| OPENGREY | / | 743 | (digital mental health interventions) AND (teen OR children OR students OR youth OR adolescents) AND (depression OR anxiety OR stress); (digital mental health service) AND (teen OR children OR students OR youth OR adolescents) AND (depression OR anxiety OR stress); (digital mental health technology) AND (teen OR children OR students OR youth OR adolescents) AND (depression OR anxiety OR stress); (digital mental health apps) AND (teen OR children OR students OR youth OR adolescents) AND (depression OR anxiety OR stress); (digital mental health program) AND (teen OR children OR students OR youth OR adolescents) AND (depression OR anxiety OR stress); (digital mental health system) AND (teen OR children OR students OR youth OR adolescents) AND (depression OR anxiety OR stress); (digital mental health care) AND (teen OR children OR students OR youth OR adolescents) AND (depression OR anxiety OR stress) |
| APA PsycExtra | publications | 241 | (digital mental health interventions) AND (teen OR children OR students OR youth OR adolescents) AND (depression OR anxiety OR stress); (digital mental health service) AND (teen OR children OR students OR youth OR adolescents) AND (depression OR anxiety OR stress); (digital mental health technology) AND (teen OR children OR students OR youth OR adolescents) AND (depression OR anxiety OR stress); (digital mental health apps) AND (teen OR children OR students OR youth OR adolescents) AND (depression OR anxiety OR stress); (digital mental health program) AND (teen OR children OR students OR youth OR adolescents) AND (depression OR anxiety OR stress); (digital mental health system) AND (teen OR children OR students OR youth OR adolescents) AND (depression OR anxiety OR stress); (digital mental health care) AND (teen OR children OR students OR youth OR adolescents) AND (depression OR anxiety OR stress) |

Note: The strategy was conducted simultaneously. Considering that DMHIs are emerging technologies, no start time was set for the literature search to retrieve a wider range of publications. The searches in all the databases above were completed on October 31, 2023. Search terms in CNKI were translated into simplified Chinese.
