# Supplementary material for: Facilitators and Barriers to Digital Mental Health Interventions for Depression, Anxiety, and Stress in Adolescents and Young Adults: Scoping Review
Source: J Med Internet Res. 2025 Mar 24;27:e62870. doi: 10.2196/62870 (PMC11988281; doi:10.2196/62870)
Supplement: Multimedia Appendix 3 [file jmir_v27i1e62870_app3.docx]

**Themes, subthemes definitions, and related examples**

| Level-Theme (Definitions) | |
| --- | --- |
| Subthemes (Definitions) | Examples |
| *Facilitators - External level* | |
| *Theme 1. Integration with Schools and Other Resources (Combining DMHIs with the school setting or curriculum and other resources as complement services.)* | |
| - 1. Integration with Schools (Combining DMHIs with the school setting or curriculum.) | …schools were considered an important setting for the intervention, particularly personal, social, and health education sessions.[6] |
| - 1. Integration with Others (Combining DMHIs with health services, jobs, and other resources.) | Several participants mentioned that they took the online screener because it was included as a resource in school _1.1_, their job, or part of a professional training.[47] |
| *Theme 2. Social Norms (The informal rules that govern behaviour in groups and societies.)* | |
| —— | Higher intentions to use DMHIs were significantly correlated with …, social norm, ….[48] |
| *Theme 3. Strategic Marketing (The activity of presenting and advertising DHMIs.)* | |
| 3.1. Avenues (Choices and ways of marketing.) | Aside from formal avenues of advertising one interviewee suggested the study have a Facebook and a Twitter presence.[45] |
| 3.2. Focus (Key points in the marketing.) | They suggested instead focusing on the likely benefits of the programme.[45] |
| 3.3. Naming (Appropriate naming of programs.) | The name and promotion of the program was discussed, and the use of the term ‘mood’ was considered more acceptable than ‘well-being’ to young people.[6] |
| *Theme 4. Universality (Universal nature and status.)* | |
| —— | Universal delivery of cCBT can reduce stigma and social isolation.[15] |
| *Theme 5. Endorsements (Approval, support and recognition from professionals, peers, and famous programs.)* | |
| 5.1. Care Providers (Endorsements by caregivers.) | …, the study was introduced by a care provider, increasing trust in the intervention.[10] |
| 5.2. Peers (Endorsements by friends and those of the same status.) | Endorsements from friends and others “like them” were most likely to get them to try a service.[47] |
| 5.3 Reputable Programs (Endorsements by well-known programs.) | Endorsements from reputable programs or mental health professionals were also perceived positively and contributed to their willingness and interest in the service.[47] |
| *Facilitators - Intervention level* | |
| *Theme 1. Content Engagement (The engaging sections contained in DMHIs.)* | |
| - 1. Information (Details, facts, materials and resources for mental health and services.) | Several stated that the self-help section was ‘motivational’ and their favorite section, and some asked for more self help approaches in specific situations.[46] |
| - 1. Personalisation (Sections that are customised or tailored to the individual.) | Most found “My goals” to be helpful and motivating.[46] |
| - 1. Support (Provision of encouragement and assistance.) | Other participants agreed that sufficient support – in terms of app features (e.g., technical assistance, frequently asked questions) as well as human support (e.g., a coach) – was essential for sustained engagement.[47] |
| - 1. Communication (Ability to express ideas, feedback, and feelings.) | All interviewees agreed that an online forum, which enabled discussion about their programme experiences, was highly desirable and was likely to boost retention, ….[45] |
| - 1. Testimonial (Sections that contain raises and evidence.) | Young people, parents, carers, and a small number of the professionals suggested adding stories from ‘celebrities’, ….[46] |
| - 1. Entertainment (Activities used to entertain people.) | The participants also suggested introducing games and videos to improve the program’s level of interactivity and entertainment value: “I would like it to be more fun, with games and videos”.[41] |
| - 1. Retention Booster (Promote participant retention.) | For instance, the app should … provide … sufficient notification to remind the participants to do the exercise or activities that they planned….[44] |
| *Theme 2. Design Harmony (The cohesive and user-friendly aspects of DMHI design.)* | |
| 2.1. Multimedia (Using various ways, such as sound, pictures, and videos, to convey information.) | The introduction of designs for the program, including elements such as illustrations, characters, metaphors, moving images, and audio, helped to guide group discussions.[6] |
| 2.2. Good Look (Pleasant and satisfying appearance.) | Participants liked the look and feel of the app, ….[49] |
| 2.3. Co-design (The design process involving user representatives and professionals.) | Participants thought that having mental health professional involved in the creation of such a service (at a minimum) was essential, ….[47] |
| 2.4. Characters (Unusual people, such as real people, role models, and celebrities.) | The stories could include both older and younger characters, role models (e.g., famous people and their stories), ….[49] |
| 2.5. Personalisation (Customised or individually tailored schemes and approaches.) | They said that the individualised interaction helped to make iCBT more personable and helped them to consolidate the learning in each session.[33] |
| 2.6. Multi-presentation (Different forms of presenting information, such as files, hierarchies, and visualisations.) | Focus group participants agreed there should be levels of information, with a hierarchy of sections and subsections.[6] |
| 2.7. Appropriate Language (Language style that is common or accessible.) | Two interviewees encouraged the use of slang and mobile telephone text message language ….[45] |
| *Theme 3.* *High Quality and Effect (Produced good results and intended outcomes.)* | |
| 3.1. Overall (Good overall effects.) | Some noted they were surprised by the high quality, …[46] |
| 3.2. Effect (Good specific effects.) | The participants also stated that the CTA website … was a useful tool for depression detection.[41] |
| 3.3. Interesting/Engaging (Pleasant and attention-grabbing.) | One in three considered programs being interesting (n=78, 37.5%) as beneficial.[14] |
| 3.4. Good Emotional Experience (Positive emotions that are subjectively felt or realised.) | …, with an example: “always makes me laugh! _3.4_ Ha ha ha ha, the pressure suddenly disappeared, and I am so happy _3.4_”.[52] |
| 3.5. Ease of Interaction (Easy sharing, communicating, and collaborating during DMHIs intake.) | Third, using text messaging …, thus increasing ease of interaction _3.5_.[10] |
| 3.6. Ease of Use (The usage is not hard and complicated.) | It was easy to use. We’re all used to the technology.[51] |
| 3.7. Relevancy (Closely connected to users.) | Overall, both young people and clinicians were positive about the age-appropriateness of the program content; its relevance for use by anxious adolescents….[12] |
| 3.8. Visual Perception (Visually pleasing experience.) | Both user groups generally found the site to be user-friendly and visually pleasing.[12] |
| *Theme 4. Appropriate Duration and Schedule (Appropriate length of time that DMHIs last or continue, and arrangements.)* | |
| 4.1. Appropriate Duration (Suitable, correct, and acceptable length of DMHIs.) | To avoid boredom they suggested no more than four videos each week with a maximum duration of 10 min each (three suggested a maximum of 5 min).[45] |
| 4.2. Appropriate Schedule (Suitable, correct, and acceptable timetables.) | Sunday morning was suggested by two interviewees as a suitable time for each weekly module of the programme…[45] |
| *Theme 5. Accessibility (Ease in entering or reaching DMHIs.)* | |
| 5.1. Multiplatform (Various operating systems or environments.) | Interview participants suggested that the program should be multiplatform, ….[6] |
| 5.2. Free/Low Cost (No or low financial burden.) | Not surprisingly, many participants … said they would be more willing to try services if they were free.[47] |
| 5.3. Ease of Access (Easily enter or reach DMHIs.) | …be in the comfort of your own home, and do things more remotely.[16] |
| *Facilitators - Individual level* | |
| *Theme 1. Beneficial Characteristics (Personal features and traits that facilitate using DMHIs.)* | |
| - 1. Gender (females) (The fact of being female.) | In the final model, participants who were female, … reported significantly greater perceived helpfulness.[14] |
| - 1. High Symptom Severity (Bad or serious mental condition.) | The motivation to “get help” was conceptualised as being directly associated with symptom severity ….[42] |
| - 1. Great Knowledge (Good information, understanding and skills.) | In the final model, participants who … reported … greater knowledge of online therapies … reported significantly greater perceived helpfulness.[14] |
| - 1. Previous Experiences (The things, event and activities that happened before.) | Higher intentions to use DMHIs were significantly correlated with previous use of DMHIs (yes), ….[48] |
| *Theme 2. Needs and Disposition (Demands and personal tendency toward DMHIs.)* | |
| 2.1. Needs (Demands for DMHIs.) | If I had a mental health problem and apps were available, I would use them.[53] |
| 2.2. Preferences (Great interest in or desire for DMHIs.) | If given the choice, the vast majority (88%, [29]) would prefer to use a computer program at home.[30] |
| 2.3. Positive Attitudes/Beliefs (Good thoughts and feelings about DMHIs.) | … less stigmatised mental health attitudes significantly predicted greater perceived benefits.[14] |
| *Theme 3. Perceived Benefits (Perceived advantages and good results.)* | |
| 3.1. Helpfulness/Usefulness (Being useful/helpful or possible to use/help) | It helped one reflect on their life or “check back in with yourself”.[49] |
| 3.2. Privacy/Security (Being alone, unobserved, and undisturbed by others.) | Security and confidentiality were also key considerations, ….[6] |
| 3.3. Time Management (Using time effectively for activities.) | Using waiting time … Stopped me from being bored.[51] |
| *Theme 4. Supportive Environment (Conditions for personal life, growth, and development.)* | |
| 4.1. Technical Environment (Technology-related conditions.) | Participants across the interviews and groups noted that using digital technologies was a valid approach to engagement, as young people use these in everyday life, ….[6] |
| 4.2. Interpersonal Catalysts (Relationships between people that make a change.) | Some participants described … hitting a personal low, which prompted them look for answers online.[47] |
| *Barriers - External level* | |
| *Theme 1. Integration with Schools (Combining DMHIs with the school setting or curriculum as lessons.)* | |
| —— | However, some young people noted that associating it with schools might make it less appealing.[46] |
| *Barriers - Intervention level* | |
| *Theme 1. Content Gaps (The missing or inappropriate sections in DMHIs.)* | |
| - 1. Cultural/Religious/Spirituality Issues (Issues related customs, beliefs, faith, religion, soul.) | All interviewees suggested not having pictures of meditators on the site ….[45] |
| - 1. Support Lacking (Lack of encouragement and assistance.) | The majority of adolescents rated the following factors as at least moderately problematic: …, being without therapist support (n = 107, 51.5%), ….[14] |
| - 1. Communication Lacking (Inability to expression of ideas, feedback, and feelings.) | Nonetheless, most participants reported … disappointment with the lack of immediate responsiveness, ….[33] |
| *Theme 2. Design Limitations (The constraints and challenges of DMHI design.)* | |
| 2.1. Roboticism (Stiff responses like a robot.) | I’m not sure how useful it was because he mentioned that it was like canned responses ….[5] |
| 2.2. Multimedia Issues (Using inappropriate ways of giving information.) | All young people stated they preferred the illustrative approach to a more inappropriate ways of giving information one.[46] |
| 2.3. Inappropriate Language (Language style that is unpleasant.) | All interviewees advised against the use of the word ‘homework’ as it may remind participants of their university or school homework and thus be off putting.[45] |
| 2.4. Burden (Things that confuse and cause challenges.) | …, a quarter (25.0%) of the participants also agreed that it was difficult for them to find their way around the program, ….[15] |
| 2.5. Personalisation Lacking (Too general, lacking customisation.) | The majority of adolescents rated the following factors as at least moderately problematic: …, information being too general, ….[14] |
| *Theme 3. Low Quality and Effect (Produced poor results and outcomes.)* | |
| 3.1. Unattractive (Not good, interesting, or pleasant.) | The site’s design was not appealing to me, ….[7] |
| 3.2. Irrelevancy (Lack of importance to or connection with people or situations.) | Four participants (11%) reported that Bite Back did not seem relevant for them.[7] |
| 3.3. Poor Using Experience (Unfavourable feelings in the process of using.) | While some enjoyed the games, others said they were too easy or too slow and ….[7] |
| 3.4. Poor Emotional Experiences (Bad emotions that are subjectively felt or realised.) | Almost a half (42.8%) of the participants had felt annoyed or frustrated going through the program.[15] |
| 3.5. Repetitiveness (Doing the same or similar thing again or more than once.) | The website was very similar each time I visited it and thus lost the initial flair it once had.[7] |
| 3.6. Low Effect (Poor specific effects.) | In terms of dislikes, the participants referred ... the advice being too ‘hard going’ (i.e. difficult to deal with) by focusing unduly on negative aspects of mental health.[15] |
| *Theme 4. Inappropriate Duration and Schedule (Inappropriate length of time that DMHIs last or continue, and arrangements.)* | |
| 4.1. Inappropriate Duration (Unsuitable, incorrect, and unacceptable length of DMHIs.) | All interviewees preferred a series of short videos each week rather than one long presentation.[45] |
| 4.2. Inappropriate Schedule (Unsuitable, incorrect, and unacceptable timetables.) | Other concerns raised by the users about the app were that…, app notifications were not frequent enough or occurred at an unwanted time.[44] |
| *Theme 5. Inaccessibility (Difficulty in entering or reaching DMHIs.)* | |
| 5.1. Technological Issues (Inaccessibility due to lack of technical support or technology-related usage issues.) | Barriers to use included not having the app on their own phone (due to it only being available on Android devices for the trial), ….[49] |
| 5.2. High Cost (High financial burden.) | In total, 2 of these 3 participants cited high costs associated with mental health services as a major barrier.[16] |
| *Barriers - Individual level* | |
| *Theme 1. Detrimental Characteristics (Personal features and traits that hinder using DMHIs.)* | |
| - 1. Physically Unwell (The body is not in a healthy state.) | The most common reasons for non-completion were … being physically unwell and unable to attend appointments.[8] |
| - 1. Lack of Confidence (Uncertainty about the ability to do something.) | …, they lacked confidence in their own ability to work therapeutically via the internet.[32] |
| - 1. Lack of Connection (Lack of being related to other people.) | Participants’ reasons for not downloading the app were as follows, …, no one to connect to, ….[17] |
| *Theme 2. Motivational Challenges (Lack of enthusiasm and personal tendency toward DMHIs.)* | |
| 2.1. No Motivation (Lack of enthusiasm for doing something.) | Participants’ reasons for not downloading the app were as follows, … no motivation, ….[17] |
| 2.2. Preferences (Great interest in or desire for other types of mental health services.) | …, three quarters of young people would prefer to meet face to face and talk with someone, ….[30] |
| 2.3. Negative Attitudes/Beliefs (Bad thoughts and feelings about DMHIs.) | …, participants had reservations about human-like support from a messaging system, ….[50] |
| *Theme 3. Perceived Risks (Perceived disadvantages and bad results.)* | |
| 3.1. Privacy/Security/Credibility Concerns (Concern about not being alone and watched or disturbed by other people, and the quality that makes people believe and trust.) | One interviewee pointed out that young people may be reluctant to undertake the programme because of concerns about privacy (e.g. when using a family or other public computer).[45] |
| 3.2. Stigma and Cyber Bullying Concerns (Feelings of disapproval and being frightened or hurt by others through the internet.) | According to this theme, help-seeking was conceptualised as involving “risk,” more specifically exposure to peer stigma and cyber bullying.[42] |
| *Theme 4. Question (Doubts or suspicions.)* | |
| 4.1. Question the Helpfulness (Doubts or suspicions about the helpfulness.) | The most common reasons for non-completion were …, not finding the resource helpful, ….[8] |
| 4.2. Question the Validity (Doubts or suspicions about the validity.) | Some participants questioned the validity of the Web site, ….[43] |
| 4.3. Question the Usefulness (Doubts or suspicions about the usefulness.) | Participants’ reasons for not downloading the app were as follows, … not useful, ….[17] |
| *Theme 5. Retention Issues (Problems that make it hard to keep on and continue.)* | |
| 5.1. Low Priority (Not considered necessary to address or conduct first.) | Barriers to use included …, forgetting about it, ….[49] |
| 5.2. Low Interest (Lack of attention, not wanting to know more.) | The most common reasons for non-completion were …, lack of interest, ….[8] |
| 5.3. Cannot Preserve (Unable to keep on or continue.) | Ten interviewees commented that …, persevering in the programme to the end and completing home practices were likely to prove difficult.[45] |
| *Theme 6. No/Limited Time (Lack of time or lack of sufficient time.)* | |
| —— | Of the 36 participants who responded, 21 (58%) cited that the reason for their underusage was time constraints.[7] |
| *Theme 7. Technical Issues (Personal reasons related to techniques, skills, or devices.)* | |
| —— | Technical issues accounted for 5 participants’ (14%) underusage, predominantly issues with Internet access.[7] |
