# Supplementary material for: Facilitators and Barriers to Digital Mental Health Interventions for Depression, Anxiety, and Stress in Adolescents and Young Adults: Scoping Review
Source: J Med Internet Res. 2025 Mar 24;27:e62870. doi: 10.2196/62870 (PMC11988281; doi:10.2196/62870)
Supplement: Multimedia Appendix 4 [file jmir_v27i1e62870_app4.docx]

THEMES AND SUB-THEMES OF FACILITATORS AND BARRIERS

Facilitators

Facilitators-External level

| Theme | Sub-theme | Original description and references |
| --- | --- | --- |
| 1. Integration with Schools and Other Resources | - 1. Integration with Schools | 1. With regard to implementation, schools were considered an important setting for the intervention _1.1_, particularly personal, social, and health education sessions. [1] 2. Some noted that the use of MoodHwb could complement existing services _1.2_, for example, by allowing young people to reflect on their entries with counsellors, General Practitioners, and primary mental health and specialist CAMHS practitioners. [2] 3. It was also felt that the program could be integrated into health and other services _1.2_. [1] 4. several participants mentioned that they took the online screener because it was included as a resource in school _1.1_, their job, or part of a professional training _1.2_. [3] |
|  | - 1. Integration with Others |  |
| 1. Social Norms | —— | 1. Higher intentions to use DMHIs were significantly correlated with previous use of DMHIs (yes), as well as positive attitude, social norm _2_, perceived behavioural control, perceived usefulness, perceived ease of use, and trust of DMHIs. [4] |
| 1. Strategic Marketing | - 1. Avenues | 1. Aside from formal avenues of advertising one interviewee suggested the study have a Facebook and a Twitter presence _3.1_. [5] 2. They suggested instead focusing on the likely benefits _3.2_ of the programme. [5] 3. Participants also expressed that advertisements and/or marketing should be upfront about the program aims and evidence for its effectiveness _3.2_. [3] 4. The name and promotion of the program was discussed, and the use of the term ‘mood’ _3.3_ was considered more acceptable than ‘well-being’ to young people. [1] 5. The program should not only have greater promotion within the participants’ schools but also on social media and other platforms _3.1_. [6] |
|  | - 1. Focus |  |
|  | - 1. Naming |  |
| 1. Universality | —— | 1. Universal delivery _4_ of cCBT can reduce stigma and social isolation. [7] |
| 1. Endorsements | - 1. Care Providers | 1. First, the study was introduced by a care provider _5.1_, increasing trust in the intervention. [8] 2. Endorsements from friends and others “like them” _5.2_ were most likely to get them to try a service. [3] 3. Endorsements from reputable programs _5.3_ or mental health professionals _5.1_ were also perceived positively and contributed to their willingness and interest in the service. [3] |
|  | - 1. Peers |  |
|  | - 1. Reputable Programs |  |

Facilitators-Intervention level

| Theme | Sub-theme | Original description and references |
| --- | --- | --- |
| 1. Content Engagement | - 1. Information | 1. Four interviewees suggested using ‘progress bars’ at the top of each page, whether a teaching module _1.3_ or outcome measurement _1.2_, so as to orient participants, create a sense of fulfilment and encourage completion. [5] 2. For instance, the app should avoid many open-ended questions in the questionnaire, provide sufficient information _1.1_ about the program (e.g., what participants are asked to do in the app) and sufficient notification _1.7_ to remind the participants to do the exercise or activities that they planned, offer timely responses _1.4_ by e-helpers, and provide additional assurances of confidentiality. [9] 3. The introduction _1.1_ made me want to complete the program. [10] 4. The participants also stated that the CTA website provided useful information on depression _1.1_ and was a useful tool for depression detection. [6] 5. They greatly appreciated that the program was guided by specialists _1.3_ who cared about them and were available to support them. [6] 6. Participants suggested that the instructions _1.1_ were clear and the questionnaire length was appropriate, but the questions in the questionnaire are repeated (e.g., weekly assessment of depressive symptoms), so they may not have variation and the 2-weeks interval. [9] 7. One participant suggested that if longer videos were made for the program, a short video summary _1.1_ should still be included. [11] 8. Group participants agreed and recommended developing other interactive elements (e.g., to set goals _1.2_ and to save links to helpful resources _1.1_). [1] 9. Most participants said that they valued the feedback _1.4_ received from the eCoach _1.3_ at the end of each session and believed this was an important component of the programme. [12] 10. They said that the individualised interaction _1.4_ helped to make iCBT more personable and helped them to consolidate the learning in each session. [12] 11. All users universally reported a preference for female e-helpers _1.3_ for phone call support because of ease or willingness of disclosure of their problems to women. [9] 12. They suggested that the app could include videos as an alternative to illustrated narratives for those who preferred videos to text, and to add notifications _1.7_ when sessions were unlocked, accompanied by messages _1.7_ from their e-helper to keep themselves on track with the program, enhance notifications _1.7_ by making them visible similar to social networking applications, add additional timeslots for e-helpers to reply to user questions _1.4_, add functions that facilitated interaction _1.4_ between the participant and characters and other study participants (e.g., group chat _1.4_), add incentives _1.7_ within the app to encourage participants to engage in activities, and limit the amount of personal content requested by the app for the e-helper’s monitoring. [9] 13. In general, the adolescents in the study said they were satisfied with their contact with the therapist _1.3_, even those who did not have much contact. [13] 14. They considered it easy to use, with practical exercises _1.2_, relatable stories _1.5_, and engaging illustrated narrations _1.5_, although a few students considered some scenarios to be less relevant or difficult to understand. [9] 15. Over two thirds of participants rated the following factors as at least moderately beneficial: use at home (n = 166, 79.9%), use any time (n = 159, 76.4%), low cost (n = 159, 76.4%), the alleviation of embarrassment (n = 150, 72.1%), ability to track progress (n = 150, 72.1%), ease of accessibility (n = 150, 72.1%), avoiding waitlists to support (n = 130, 62.5%), and greater interactivity that self-help resources _1.1_ (n = 127, 61.1%). [14] 16. Participants explicitly expressed a desire for iCBT to include more human interaction and more opportunities _1.4_ for relating to others. [12] 17. All interviewees agreed that an online forum _1.4_, which enabled discussion about their programme experiences, was highly desirable and was likely to boost retention, significantly through: clarifying aspects of the teaching; sharing and overcoming difficulties with practice; and encouraging participants to remain engaged and complete home practice sessions. [5] 18. Suggestions were made to help overcome some of these reported barriers, which overwhelmingly included adding more content and variety, including additional characters or stories _1.5_ and more interactive or challenging games _1.6_. [15] 19. Most found “My goals _1.2_” to be helpful and motivating. [2] 20. Several stated that the self-help section _1.1_ was ‘motivational’ and their favorite section, and some asked for more self-help approaches _1.1_ in specific situations. [2] 21. Participants also highlighted the importance of providing input and feedback _1.4_, such that their patterns of engagement and responses would help shape the content delivered. [16] 22. All participants made favourable comments about the personal stories _1.5_, and how they were from a range of perspectives. Young people, parents, carers, and a small number of the professionals suggested adding stories from ‘celebrities’ _1.5_, and including photos, animations, videos, or comic strips. [2] 23. Overwhelmingly, playing the games _1.6_ was mentioned as a favourite part of the app, as was watching the videos. [15] 24. The stories _1.5_ could include both older and younger characters, role models (e.g., famous people and their stories), and more in-depth, place-specific stories. [15] 25. One participant suggested having a bit more instruction _1.1_ or a tutorial _1.3_ on how to work through the app. [15] 26. “Stuff I Like _1.2_” ——Some young people had used this to add songs and images and felt that it helped to personalize the program. [2] 27. Most noted that the information _1.1_ was important and comprehensive, particularly on anxiety, eating and weight issues, and physical health _1.1_. [2] 28. There should be information _1.1_ on mood, the signs, symptoms and effects of depression, the difference with “normal sadness,” and related issues such as anxiety and accounts of personal experiences. Many young people and parents wished to highlight to adolescents that they are “not alone” in their experiences of depression. [1] 29. Other participants agreed that sufficient support _1.3_ – in terms of app features _1.3_ (e.g., technical assistance, frequently asked questions) as well as human support _1.3_ (e.g., a coach) - was essential for sustained engagement. [3] 30. All 5 participants expressed having had a highly positive experience with their mentors _1.3_. [11] 31. Although suggesting ‘as little text as possible’ (2-3 sentences in each page) on webpages they asked that teaching material _1.3_ be available as a downloadable PDF file as well as videos. [5] 32. Other participants felt that a mental health coach _1.3_ could be a useful complement. [3] 33. The students who were more positive about SPARX-R expressed that playing a serious game like SPARX-R was easy to learn from and fun. The game being activity-based _1.6_ and interactive and wanting to progress from one level to the next kept the students engaged. The gaming approach was considered to assist learning and concentration. [7] 34. The participants mentioned three aspects of SPARX-R that they liked: 1) the gaming elements _1.6_, such as customizing your avatar and completing challenges,…. [7] 35. A consistent message from all interviewees was that any form of feedback _1.4_ or communication _1.4_ from the programme was likely to improve retention. In addition to forms of feedback _1.4_ already mentioned, email (even if automated and using a ‘no-reply’ address), and text message reminders, were thought to be likely to be helpful without being intrusive. [5] 36. Participants in the interviews stated there should be different levels of information and a clear explanation of the program and its aims _1.1_. [1] 37. Third, using text messaging to conduct most communication _1.4_ limited the amount of extra steps traditionally involved with apps or web pages, thus increasing ease of interaction. [8] 38. Additional audio was mentioned by a few participants that could be used in various ways. For example, a voice with encouraging feedback _1.4_ (e.g., “Well done. You’re doing great”) or to read out the stories for those who have trouble or do not like reading. [15] 39. Three interviewees suggested providing brief personalized feedback _1.2_ about scale scores and graphs showing changes at follow-up assessments _1.2_. [5] 40. In these cases, the online screener _1.2_ was seen as an objective measure that could provide them with the assurance they desired_._ [3] 41. In terms of recommendations for video content and engagement, one participant recommended that mentors use the videos to further engage with participants on weekly content and discussions _1.4_ while in the Appa Health program. [11] 42. Other participants recommended that the videos expand to further share more ‘personal experiences’ _1.4_ or to illustrate more longer-term approaches to understanding and handling one’s mental health _1.1_. [11] 43. The participants also suggested incorporating more spaces for communication and opportunities for personal expression _1.4_ and group interaction. [6] 44. The participants also suggested introducing games and videos to improve the program’s level of interactivity and entertainment value _1.6_: ‘I would like it to be more fun, with games and videos’. [6] 45. Another important issue for adolescents is closeness and face-to-face interaction _1.4_. Some participants expected to have more availability of face-to-face activities. [6] |
|  | - 1. Personalisation |  |
|  | - 1. Support |  |
|  | - 1. Communication |  |
|  | - 1. Testimonial |  |
|  | - 1. Entertainment |  |
|  | - 1. Retention Booster |  |
| 1. Design Harmony | - 1. Multimedia | 1. All participants commented positively about the quality and age-appropriateness of videos _2.1_, images _2.1_, animations _2.1_, logo _2.1_, and “comic book” style illustrations _2.1_ but suggested adding more visual cues _2.1_ for key concepts. [17] 2. All participants expressed positive attitudes toward the Appa Health program short-form CBT skill videos _2.1_. [11] 3. Young people, parents and carers described the illustrations _2.1_ and animations _2.1_ as ‘friendly’ and ‘sophisticated’ without being ‘patronizing’. [2] 4. Adolescents appreciated how the program alternated between text, pictures, and films _2.1_, and that several people with different anxiety problems _2.4_ were presented in the program. [13] 5. The introduction of designs for the program, including elements such as illustrations _2.1_, characters _2.4_, metaphors _2.6_, moving images _2.1_, and audio _2.1_, helped to guide group discussions. [1] 6. Instead they suggested pictures _2.1_ of young people ‘being happy, being active and having fun’ perhaps enhanced with online animation _2.1_. [5] 7. When asked specifically about what the program should include, the main recommendations were to show true stories of adolescents _2.4_ on the website and incorporate “videos about the experience of other people _2.4_”. [6] 8. They said that the individualised _2.5_ interaction helped to make iCBT more personable and helped them to consolidate the learning in each session. [12] 9. They considered it easy to use, with practical exercises, relatable stories, and engaging illustrated _2.1_ narrations, although a few students considered some scenarios to be less relevant or difficult to understand. [9] 10. All interviewees were in favour of information delivery in video format _2.1_ rather than through text or audio. [5] 11. All interviewees preferred a series of short videos _2.1_ each week rather than one long presentation. [5] 12. Several described how they adapted their time or work with the treatment to fit in with other demands in their life _2.5_. [13] 13. Nearly all praised the mix of rating scales (and corresponding face icons) _2.6_ and multiple-choice questions _2.6_ at the start of the program (which could also be answered subsequently to monitor mood), particularly for their functionality, ease of use, and “fun” element. [2] 14. Many participants felt that increased use of videos _2.1_ (rather than text) and the use of “real” people _2.4_ (rather than actors, presenters or celebrities) in the videos would be helpful in encouraging adolescent males to use computerised anxiety treatments. [18] 15. They liked the flow _2.6_ of information, and how text was broken up (e.g., by illustrations) _2.1_, “bite size,” _2.6_ and “short and snappy.” _2.6_ [2] 16. Additional audio _2.1_ was mentioned by a few participants that could be used in various ways. For example, a voice _2.1_ with encouraging feedback (e.g., “Well done. You’re doing great” [P24]) or to read out the stories for those who have trouble or do not like reading (P35, 31). [15] 17. They suggested that the app could include videos _2.1_ as an alternative to illustrated narratives for those who preferred videos to text _2.5_, and to add notifications when sessions were unlocked, accompanied by messages from their e-helper to keep themselves on track with the program, enhance notifications by making them visible _2.6_ similar to social networking applications, add additional timeslots for e-helpers to reply to user questions, add functions that facilitated interaction between the participant and characters and other study participants (e.g., group chat), add incentives within the app to encourage participants to engage in activities, and limit the amount of personal content requested by the app for the e-helper’s monitoring. [9] 18. Participants liked the look _2.2_ and feel of the app, it “felt natural. It wasn’t something that was like foreign”. [15] 19. Less than half of the participants agreed that they liked the look _2.2_ of SPARX-R (46.5%) or that they thought that the program was fun (39.2%). [7] 20. Four interviewees suggested using ‘progress bars’ _2.5_ at the top of each page, whether a teaching module or outcome measurement, so as to orient participants, create a sense of fulfilment and encourage completion. [5] 21. Participants thought that having mental health professional involved _2.3_ in the creation of such a service (at a minimum) was essential, as it would make the service more targeted for mental health and they would be more compelled to give it a try. [3] 22. Third, using text messaging _2.1_ to conduct most communication limited the amount of extra steps traditionally involved with apps or web pages, thus increasing ease of interaction. [8] 23. All participants made favourable comments about the personal stories, and how they were from a range of perspectives (quote 9). Young people, parents, carers, and a small number of the professionals suggested adding stories from ‘celebrities,’ and including photos _2.1_, animations _2.1_, videos _2.1_, or comic strips _2.1_. [2] 24. One interviewee suggested dividing _2.6_ questions into sections (e.g. titled ‘positive stuff’ etc.) to make the task less daunting. [5] 25. The stories could include both older and younger characters _2.4_, role models _2.4_ (e.g., famous people and their stories), and more in-depth, place-specific stories. [15] 26. “Stuff I Like” ——Some young people had used this to add songs _2.1_ and images _2.1_, and felt that it helped to personalize _2.5_ the program. [2] 27. Most recommended tailoring or personalizing _2.5_ the program, for example, by using a log-in, tailoring or modifying the content according to the needs of the user, allowing the user to save or upload information, and to set goals. [1] 28. While automated personalization _2.5_ was seen as a strength, youth testers also provided feedback about their desire for control over aspects of program workflow. [17] 29. Participants felt they could easily and efficiently navigate the intervention, that the site was user friendly, the menu bar _2.6_ was easy to use, and that buttons and menu options worked as they expected them to. [17] 30. Many approved of the general tone _2.7_, stating it was ‘sensitive’ and ‘affirming’. [2] 31. Two interviewees encouraged the use of slang and mobile telephone text message language _2.7_ (e.g. abbreviating ‘you’ to ‘u’) especially in headings. [5] 32. All interviewees advised against the use of the word ‘homework’ as it may remind participants of their university or school homework and thus be off putting. Suggested alternatives were ‘home practice’ _2.7_, ‘meditation practice’ _2.7_, ‘rest’ or ‘timeout’ _2.7_. [5] 33. Comments relating to this theme referred to avoiding jargonistic, medical or formal language (i.e. use the term ‘support’_2.7_ rather than ‘treatment’) and a preference for more informal methods of communication (i.e. Facebook chat _2.1_, messenger or text _2.1_ as opposed to email) when communicating with the online buddy or other forum members. [18] 34. While participants were open to receiving a variety of content types, with the specific schedule generally being unpredictable, they also recognized that there were some contexts where they would want to set their preferences more actively _2.5_. [16] 35. As recommended by participants, the information was developed to be clear, factual_2_, comprehensive, age-appropriate, avoiding jargon, but not patronizing _2.7_. [1] 36. Another interviewee suggested phrasing the initial registration step as an ‘application process’ _2.7_. [5] 37. the MoST-MH intervention adapted over time _2.5_ so that if an individual was not in need of support, the check-ins were stepped down (i.e., occurred monthly), reducing the burden on individuals who seemed to not need help at that time. [8] 38. Although suggesting ‘as little text as possible’ (2-3 sentences in each page) on webpages they asked that teaching material be available as a downloadable PDF file _2.6_ as well as videos _2.1_. [5] 39. A professional look _2.2_ and feel was also important to participants. For example, several participants mentioned that they would not engage with a service that looked “cheesy”, “spammy” or “sketchy” These descriptive characteristics were associated with lower quality and a non-professional service that was unlikely to be helpful – or worse could present some risk to safety or mental health. [3] 40. The participants mentioned three aspects of SPARX-R that they liked: 1) the gaming elements, such as customizing your avatar _2.5_ and completing challenges,…. [7] 41. Everyone highlighted the advantages of multimedia _2.1_, given its role in the lives of young people and that the program should also target those concerned about a young person. [1] 42. In summary, participants, in particular young people, gave favourable feedback on the design features, including the overall presentation _2.2_, structure, and interactive and graphic elements _2.1_. [2] 43. The text messages _2.1_ sent as reminders were thought to prompt use. [15] 44. In addition to forms of feedback already mentioned, email _2.1_ (even if automated and using a ‘no-reply’ address), and text message _2.1_ reminders, were thought to be likely to be helpful without being intrusive. [5] 45. Focus group participants agreed there should be levels of information, with a hierarchy of sections and subsections _2.6_. [1] 46. Participants in the interviews stated there should be different levels of information _2.6_ and a clear explanation of the program and its aims. [1] 47. Would like it with more colour _2.2_. [19] 48. Participants felt that lightweight human support could increase the legitimacy of the program, but it should be optional _2.5_. [3] 49. Two forms of reward were suggested by interviewees to ensure programme and outcome measure completion…. (1) by highlighting the opportunity _2.5_ to benefit other young people a sense of ‘doing good’ and ‘being part of a bigger thing’ may be created. (2) physical rewards _2.5_: a certificate; a token reward (e.g. sticker); a prize draw for items of monetary value. [5] 50. All interviewees agreed that an online forum, which enabled discussion about their programme experiences, was highly desirable and was likely to boost retention, significantly through: clarifying aspects of the teaching _2.5_; sharing and overcoming difficulties with practice _2.5_; and encouraging participants to remain engaged and complete home practice sessions _2.5_. [5] 51. To help with adherence and encourage support, participants in the interviews and groups suggested it would be helpful for the young person to be able to use the program with others, including family, carers, friends, or professionals, as well as independently _2.5_. [1] 52. Three interviewees suggested providing brief personalized feedback about scale scores and graphs _2.1_ showing changes at follow-up assessments. [5] 53. On the whole, participants agreed that the language used was accessible _2.7_, simple, and jargon-free, but ‘not too dumbed down’. [2] 54. With respect to the monitoring assessments, the participants recommended diversifying the questions _2.6_ and suggested that the program “should ask us more questions about what we do outside of school, or about what we do in our spare time”. [6] 55. According to participants, the staff should be available to give an individual and “timely solution” to the specific needs of each user _2.5_, “to act immediately, because that’s when things happen”. [6] |
|  | - 1. Good Look |  |
|  | - 1. Co-design |  |
|  | - 1. Characters |  |
|  | - 1. Personalisation |  |
|  | - 1. Multi-presentation |  |
|  | - 1. Appropriate Language |  |
| 1. High Quality and Effect | - 1. Overall | 1. Some noted they were surprised by the high quality _3.1_, and that they had reservations about it previously because it might be “academic,” “dry,” or “overwhelming.” [2] 2. All participants commented positively about the quality _3.1_ and age-appropriateness _3.7_ of videos, images, animations, logo, and “comic book” style illustrations but suggested adding more visual cues for key concepts. [17] 3. Participants also stated that they found the psychoeducational videos on the website entertaining _3.3_ and informative _3.2_. [6] 4. Young people, parents and carers described the illustrations and animations as “friendly” _3.8_ and “sophisticated” _3.8_ without being “patronizing”. [2] 5. Nearly all praised the mix of rating scales (and corresponding face icons) and multiple-choice questions at the start of the program (which could also be answered subsequently to monitor mood), particularly for their functionality _3.2_, ease of use _3.6_, and “fun” _3.3_ element. [2] 6. Participants liked the look and feel _3.1_ of the app, it “felt natural _3.6_. It wasn’t something that was like foreign.” [15] 7. It has a New Zealand look and feel _3.1._ [20] 8. Both user groups generally found the site to be user-friendly and visually pleasing _3.8_. [17] 9. Most found “My goals” to be helpful _3.1_ and motivating _3.4_. [2] 10. They considered it easy to use _3.6_, with practical exercises, relatable stories, and engaging illustrated narrations, although a few students considered some scenarios to be less relevant or difficult to understand. [9] 11. The keywords of personalization theme are “thinking”, “learning”, “depression”, “mood” and “intelligence”, with an example: “The best experience is that sometimes XiaoE’s answers are indeed valuable and can really target some of my questions _3.7_, which is very intelligent and promotes thinking”. [21] 12. Lastly, one participant also recommended including video content on feeling “overwhelmed” and “overworked” due to school commitments and demands _3.7_. [11] 13. Most found the overall design attractive _3.3_, and more interesting _3.3_ and appropriate _3.7_ than designs for existing resources. [2] 14. Although most users and buddies found Village appealing to use _3.3_ and easy to navigate, a few experienced difficulties downloading or using the app for the first time. [22] 15. Participants felt they could easily and efficiently navigate the intervention, that the site was user friendly _3.6_, the menu bar was easy to use _3.6_, and that buttons and menu options worked as they expected _3._ them to. [17] 16. Third, using text messaging to conduct most communication limited the amount of extra steps traditionally involved with apps or web pages, thus increasing ease of interaction _3.5_. [8] 17. Over two thirds of participants rated the following factors as at least moderately beneficial: use at home (n = 166, 79.9%), use any time (n = 159, 76.4%), low cost (n = 159, 76.4%), the alleviation of embarrassment (n = 150, 72.1%) _3.4_, ability to track progress _3.2_ (n = 150, 72.1%), ease of accessibility (n = 150, 72.1%), avoiding waitlists to support (n = 130, 62.5%), and greater interactivity _3.5_ that self-help resources (n = 127, 61.1%). [14] 18. The participants also stated that the CTA website provided useful information on depression and was a useful tool for depression detection _3.2_. [6] 19. They suggested that the app could include videos as an alternative to illustrated narratives for those who preferred videos to text, and to add notifications when sessions were unlocked, accompanied by messages from their e-helper to keep themselves on track with the program _3.2_, enhance notifications by making them visible similar to social networking applications, add additional timeslots for e-helpers to reply to user questions, add functions that facilitated interaction between the participant and characters and other study participants (e.g., group chat), add incentives within the app to encourage participants to engage in activities, and limit the amount of personal content requested by the app for the e-helper’s monitoring. [9] 20. Several stated that the self-help section was ‘motivational’ _3.4_ and their favourite section, and some asked for more self-help approaches in specific situations. [2] 21. One interviewee suggested dividing questions into sections (e.g. titled ‘positive stuff’ etc.) to make the task less daunting _3.4_. [5] 22. Two forms of reward were suggested by interviewees to ensure programme and outcome measure completion _3.2_. [5] 23. The participants mentioned three aspects of SPARX-R that they liked: 1) the gaming elements, such as customizing your avatar and completing challenges, 2) ease of use _3.6_, and 3) helpfulness, particularly around managing emotions. [7] 24. Presentation (questionnaire): ‘It was alright. Simple _3.6_.’ [19] 25. The questions weren’t difficult _3.6_ and were honest questions. [19] 26. Of the 62 participants who used the program for 6 weeks, 49 reported that Bite Back was fun _3.3_ (79% rated mildly agree to strongly agree), with 52 agreeing that the activities were interesting _3.3_, and 56 reporting that the site was easy to use _3.6_ (90% rated mildly agree to strongly agree). [23] 27. One in three considered programs being interesting _3.3_ (n=78, 37.5%) as beneficial. [14] 28. Playing the game was enjoyable _3.4_. [7] 29. Participants suggested that the instructions were clear _3.2_ and the questionnaire length was appropriate, but the questions in the questionnaire are repeated (e.g., weekly assessment of depressive symptoms), so they may not have variation and the 2-weeks interval. [9] 30. Others considered it to be worthwhile, innovative _3.2_, but not dynamic, as it did not respond to user inputs. [9] 31. Less than half of the participants agreed that they liked the look of SPARX-R (46.5%) or that they thought that the program was fun _3.3_ (39.2%). Although more than half (55.5%) of the participants considered the program interesting _3.3_, nearly a half (46.5%) also indicated that they found the program boring. [7] 32. Most felt that YouthCHAT was appropriate for their age group _3.7_ and would recommend it to others. [19] 33. In general, participants were pleased _3.4_ with the content, and found it comprehensive _3.2_. [2] 34. The keywords for the emotion theme are “happy”, “relax”, “stress”, “catharsis” and “company”, with an example: “always makes me laugh! _3.4_ Ha ha ha ha, the pressure suddenly disappeared, and I am so happy _3.4_”. [21] 35. Overall, both young people and clinicians were positive about the age-appropriateness _3.7_ of the program content; its relevance _3.7_ for use by anxious adolescents and the length of each module. [17] 36. Many young people and parents wished to highlight to adolescents that they are “not alone” _3.4_ in their experiences of depression. [1] 37. XiaoE is very sweet, I like to talk to XiaoE, he will accompany and accept me, so I don’t feel lonely _3.4_. [21] 38. Overall, young people, parents, and carers felt that the amount of information included in each section was adequate _3.2_. [2] 39. Both groups rated the ease of use _3.6_ and clarity highly, and approved of the design elements, although parents and carers were slightly less positive than young people. [2] 40. Complementing our finding high MoST-MH engagement, we found high usability ratings, suggesting the ease of use _3.6_ of text messages and web-based interfaces as well as the brief clear nature of support messaging. [8] 41. It was easy to use _3.6_. We’re all used to the technology. [19] 42. Higher intentions to use DMHIs were significantly correlated with previous use of DMHIs (yes), as well as positive attitude, social norm, perceived behavioural control, perceived usefulness, perceived ease of use _3.6_, and trust of DMHIs. [4] 43. Most of the participants considered SPARX-R easy to use _3.6_, with 71.4% agreeing that the language was easy to understand _3.6_, 75.0% agreeing that they understood what they were supposed to do on the program, and 71.4% agreeing that their computer skills were adequate for using the program. [7] 44. Ease of use _3.6_ [22] 45. Many approved of the general tone, stating it was ‘sensitive’ _3.2_ and ‘affirming’ _3.2_. [2] 46. As recommended by participants, the information was developed to be clear _3.2_, factual _3.2_, comprehensive _3.2_, age-appropriate _3.7_, avoiding jargon, but not patronizing. [1] 47. The lessons ‘made sense’ _3.7_ to me/The lessons related well _3.7_ to my own life [7] 48. The lessons in the module ‘made sense’ to me _3.7_./This module struck a chord with my own life _3.7_./One of the story examples struck a chord with my own life _3.7_. [10] 49. Four interviewees suggested using ‘progress bars’ at the top of each page, whether a teaching module or outcome measurement, so as to orient participants, create a sense of fulfilment _3.4_ and encourage completion _3.2_. [5] 50. Interactivity _3.5_ improved learning and concentration [7] 51. The stories could include both older and younger characters, role models (e.g., famous people and their stories), and more in-depth _3.2_, place-specific _3.2_ stories. [15] 52. Most noted that the information was important _3.2_ and comprehensive _3.2_, particularly on anxiety, eating and weight issues, and physical health. [2] 53. The students who were more positive about SPARX-R expressed that playing a serious game like SPARX-R was easy to learn from _3.6_ and fun _3.3_. The game being activity-based and interactive _3.5_ and wanting to progress from one level to the next kept the students engaged. [7] 54. Participants suggested that the tone, language, and terminology should be at the level of the young person _3.7_ and discussed how much text would be appropriate. [1] 55. Overall, participants felt that the program was clearly and consistently structured, easily navigated _3.6_ and user-friendly _3.6_. [2] 56. They also advised there should be clear structure and navigation _3.6_. [1] 57. Although most users and buddies found Village appealing to use and easy to navigate _3.6_, a few experienced difficulties downloading or using the app for the first time. [22] 58. Participants felt they could easily and efficiently navigate _3.6_ the intervention, that the site was user friendly, the menu bar was easy to use, and that buttons and menu options worked as they expected them to. [17] 59. Some stated that the program could help counteract stigma _3.4_ and making it freely available would help with this. [2] 60. They described a sense of not being alone _3.4_, and of having a shared understanding of each other’s difficulties _3.4_. [24] 61. This module was easy to read _3.6_./This module was easy to understand _3.6_. [10] 62. The introduction made me want to complete _3.2_ the program. [10] 63. It is made especially for young people _3.7_. [20] |
|  | - 1. Effect |  |
|  | - 1. Interesting/   Engaging |  |
|  | - 1. Good Emotional Experience |  |
|  | - 1. Ease of Interaction |  |
|  | - 1. Ease of Use |  |
|  | - 1. Relevancy |  |
|  | - 1. Visual Perception |  |
| 1. Appropriate Duration and Schedule | - 1. Appropriate Duration | 1. Although participants liked the length of the modules _4.1_ the amount of information on each page was consistently scored lower across both cycles and user groups. [17] 2. To avoid boredom they suggested no more than four videos each week with a maximum duration of 10 min each _4.1_ (three suggested a maximum of 5 min). [5] 3. One interviewee suggested that the whole week’s page not be longer than one mouse scroll long _4.1_. [5] 4. Sunday morning was suggested by two interviewees as a suitable time _4.2_ for each weekly module of the programme to become available as ‘young people check their email and Facebook on a Sunday afternoon.’ [5] 5. In addition to noting the need for the e-intervention to be less text-intensive and more interactive, participants also suggested truncating the intervention _4.1_. [12] 6. “I think the website should be more appealing, interactive, and entertaining, with less text _4.1_”. [6] 7. Participants suggested that the instructions were clear and the questionnaire length was appropriate _4.1_, but the questions in the questionnaire are repeated (e.g., weekly assessment of depressive symptoms), so they may not have variation and the 2-weeks interval. [9] 8. They suggested that the app could include videos as an alternative to illustrated narratives for those who preferred videos to text, and to add notifications when sessions were unlocked, accompanied by messages from their e-helper to keep themselves on track with the program, enhance notifications by making them visible similar to social networking applications, add additional timeslots for e-helpers to reply to user questions, add functions that facilitated interaction between the participant and characters and other study participants (e.g., group chat), add incentives within the app to encourage participants to engage in activities, and limit the amount of personal content _4.1_ requested by the app for the e-helper’s monitoring. [9] 9. Participants all reported liking the length of the videos _4.1_, expressing that it facilitated engagement with the content. [11] 10. Overall, young people, parents, and carers felt that the amount of information included in each section was adequate _4.1_. [2] 11. Participants suggested that the tone, language, and terminology should be at the level of the young person and discussed how much text would be appropriate _4.1_. [1] 12. Although suggesting ‘as little text as possible’ _4.1_ (2-3 sentences in each page) on webpages they asked that teaching material be available as a downloadable PDF file as well as videos. [5] 13. I can learn things by myself at my own pace 4.2. [20] |
|  | - 1. Appropriate Schedule |  |
| 1. Accessibility | - 1. Multiplatform | 1. Interview participants suggested that the program should be multiplatform _5.1_, and there should be a mood monitor and an app. [1] 2. Participants mentioned that e-mails are not an appropriate way of contacting them, and that contact “should be more direct”. In this regard, they recommend complementing the program with other sites and social media platforms, such as Facebook and Instagram _5.1_. [6] 3. Some stated that the program could help counteract stigma and making it freely available _5.2_ would help with this. [2] 4. Not surprisingly, many participants cited financial barriers to existing services and said they would be more willing to try services if they were free _5.2_. [3] 5. Over two thirds of participants rated the following factors as at least moderately beneficial: use at home _5.3_ (n = 166, 79.9%), use any time _5.3_ (n = 159, 76.4%), low cost _5.2_ (n = 159, 76.4%), the alleviation of embarrassment (n = 150, 72.1%), ability to track progress (n = 150, 72.1%), ease of accessibility _5.3_ (n = 150, 72.1%), avoiding waitlists to support (n = 130, 62.5%), and greater interactivity that self-help resources (n = 127, 61.1%). [14] 6. Adolescents strongly endorsed the availability of online therapies freely _5.2_ online, in schools, at the doctor, and in mental health clinics. [14] 7. Likelihood of CMHHS determined by ease of access _5.3_. [18] 8. cCBT makes mental health support more accessible _5.3_. [7] 9. On the whole, participants agreed that the language used was accessible _5.3_, simple, and jargon-free, but ‘not too dumbed down’. [2] 10. All interviewees commented that an online mindfulness programme would be of interest to their peers. They suggested that many young people would prefer it to a live programme as (i) the latter would take too much time; (ii) it would involve travelling to where training was held which would be a ‘hassle’ _5.3_; [5] 11. So it was really nice for [Appa] to just, you know, be in the comfort of your own home _5.3_, and do things more remotely _5.3_. [11] 12. I could do it at home _5.3_./ I could do it at school or in the clinic _5.3_. [20] 13. Participants expressed a perception that the iCBT was more accessible and convenient _5.3_ than traditional psychotherapy. [12] |
|  | - 1. Free/Low Cost |  |
|  | - 1. Ease of Access |  |

Facilitators-Individual level

| Theme | Sub-theme | Original description and references |
| --- | --- | --- |
| 1. Beneficial Characteristics | - 1. Gender (females) | 1. In the final model, participants who were female _1.1_, had no prior experience with online therapies _1.4_, and reported greater technology liking, greater knowledge _1.3_ of online therapies and less stigmatized mental health attitudes reported significantly greater perceived helpfulness. [14] 2. The motivation to “get help” was conceptualised as being directly associated with symptom severity _1.2_ and, more specifically, how much the anxiety was interfering in a young person’s life. [18] 3. Higher intentions to use DMHIs were significantly correlated with previous use of DMHIs (yes) _1.4_, as well as positive attitude, social norm, perceived behavioural control, perceived usefulness, perceived ease of use, and trust of DMHIs. [4] |
|  | - 1. High Symptom Severity |  |
|  | - 1. Great Knowledge |  |
|  | - 1. Previous Experiences |  |
| 1. Needs and Disposition | - 1. Needs | 1. All interview and group participants described how there was a need for this program _2.1_, especially given the lack of specialist CAMHS and Web-based resources. [1] 2. Most participants said that they were inclined to use the iCBT because they had been aware of their symptoms and they had accepted that they needed help _2.1_. [12] 3. Use the internet for mental health purposes _2.1_. [25] 4. If I had a mental health problem _2.1_ and apps were available, I would use them. [25] 5. Higher intentions to use DMHIs were significantly correlated with previous use of DMHIs (yes), as well as positive attitude _2.3_, social norm, perceived behavioural control, perceived usefulness, perceived ease of use, and trust of DMHIs. [4] 6. In the final model, greater technology liking _2.2_ and less stigmatized mental health attitudes _2.3_ significantly predicted greater perceived benefits. [14] 7. Appealing to Motivation to Help Oneself _2.1_——“If I know I need help, that might be why I’m on the Mental Health America website.” [16] 8. An important aspect that all young people in the study raised was the independence of the treatment work _2.2_. [13] 9. The participants sought and offered advice _2.1_, focusing on both practical information and emotional support. [24] 10. If given the choice, the vast majority (29, 88%) would prefer to use a computer program at home _2.2_. [26] 11. Participants said that iCBT gave them greater autonomy and control _2.2_ over their treatment. [12] 12. Given young people’s preference for autonomy _2.2_, we explored comfort with various degrees of human support more deeply as part of the second focus group. [3] 13. Some participants expressed greater comfort in using a resource completely on their own _2.2_. [3] 14. They suggested that many young people would prefer it to a live programme as…(iv)‘if you’re all anxious you wouldn’t want to sit with a group of people’ _2.2_. [5] 15. While automated personalization was seen as a strength, youth testers also provided feedback about their desire for control over aspects of program workflow _2.2_ (e.g., wanting to decide for themselves if parents should be notified of intervention progress; ability to see a summary of their progress over time, ability to self-select when and how often reminder emails were sent to them). [17] 16. Outcomes from linear regression indicated that greater perceived usefulness (β = .24) and trust of DMHIs _2.3_ (β = .28) have significant small to moderate positive associations with higher intentions to use DMHIs. [4] 17. However, some who had prior experience with mental health services found guided responses ‘robotic’ and said they would prefer greater freedom to compose more personally relevant messages _2.2_. [22] 18. Higher intentions to use DMHIs were significantly correlated with previous use of DMHIs (yes), as well as positive attitude, social norm, perceived behavioural control _2.2_, perceived usefulness, perceived ease of use, and trust of DMHIs. [4] |
|  | - 1. Preferences |  |
|  | - 1. Positive Attitudes/Beliefs |  |
| 1. Perceived Benefits | - 1. Helpfulness/Usefulness | 1. [I learned] to be patient and ask for help _3.1_ if you don’t know what to do [7] 2. Most found “My goals” to be helpful _3.1_ and motivating. [2] 3. Participants suggested the app could help them or their mates get help in tough times _3.1_. “I used it to like give me strategies and that to deal with tough times.” [15] 4. Most participants reported that they found the anonymity _3.2_ of the iCBT appealing, saying this facilitated their initial engagement with the programme. [12] 5. “practical, real and convenient, can help me _3.1_”. [21] 6. It helped one reflect on their life or “check back in with yourself” _3.1_. [15] 7. Participants reported that iCBT facilitated self-disclosure and emotional expression _3.1_, saying that they found it easier to acknowledge their feelings and thoughts online. [12] 8. Most participants said that the iCBT intervention was helpful because it enabled them to learn new skills _3.1_ which were practical and effective. [12] 9. This may not only help them to express _3.1_ negative emotional states but also allows them to handle _3.1_ negative emotions. [6] 10. Consequently, many sought external validation _3.1_ of their symptoms from the self-screener, or a second opinion _3.1_ that could confirm their intuition about having a mental health condition. [3] 11. In these cases, the online screener was seen as an objective measure that could provide them with the assurance they desired _3.1_. [3] 12. Others came with the expectation that the screener results would help them determine next steps or locate resources _3.1_. [3] 13. Security and confidentiality _3.2_ were also key considerations, and this was reflected, for example, in the password-protected log-in and encryption of data. [1] 14. Participants felt that the program could help with communication _3.1_ between young people and their family, carers and friends. [2] 15. The participants mentioned three aspects of SPARX-R that they liked: 1) the gaming elements, such as customizing your avatar and completing challenges, 2) ease of use, and 3) helpfulness _3.1_, particularly around managing emotions. [7] 16. The iPad helped pick up issues _3.1_ that I didn’t think I needed help with.[19] 17. It helped me cut down and reflect _3.1_ on the multiple things I need help with. [19] 18. The gaming approach was considered to assist learning and concentration _3.1_. [7] 19. The majority said it helped them think about and identify problems _3.1_ and talk with their doctor, and it also helped their doctor be aware of these issues. [19] 20. Reasons for use included that it helped them feel relaxed _3.1_, helped if they were bored _3.1_, used it if their other phone was flat, checked in, and helped if they were feeling down _3.1_. “I really like it because it’s helped me like get happy.” _3.1_ [15] 21. In terms of subjective experience, participants reported that it helped them feel relaxed, happy, calm, or safe _3.1_. [15] 22. Another said it helped them “feel better about myself.” _3.1_ [15] 23. Outcomes from linear regression indicated that greater perceived usefulness (β = .24) _3.1_ and trust of DMHIs (β = .28) have significant small to moderate positive associations with higher intentions to use DMHIs. [4] 24. Higher intentions to use DMHIs were significantly correlated with previous use of DMHIs (yes), as well as positive attitude, social norm, perceived behavioural control, perceived usefulness _3.1_, perceived ease of use, and trust of DMHIs. [4] 25. The majority of adolescents perceived online therapies as helpful _3.1_, albeit to different degrees. [14] 26. Support with messaging _3.1_; Improved mental health knowledge _3.1_; Positive impact on relationships _3.1_ [22] 27. The program was useful/worth doing _3.1_/I have used the tips and advice _3.1_ in my own life/I learned something new _3.1_ from the program [7] 28. Using waiting time _3.3_——Doing the YouthCHAT was way better in the waiting room than looking lost… Stopped me from being bored. [19] 29. Time to think _3.3_——The iPad helped [give us more time] to think _3.3_ about our answers. [19] 30. Over two thirds of participants rated the following factors as at least moderately beneficial: use at home (n = 166, 79.9%), use any time (n = 159, 76.4%), low cost (n = 159, 76.4%), the alleviation of embarrassment (n = 150, 72.1%), ability to track progress (n = 150, 72.1%), ease of accessibility (n = 150, 72.1%), avoiding waitlists to support _3.3_ (n = 130, 62.5%), and greater interactivity that self-help resources (n = 127, 61.1%). [14] 31. I found this module helpful _3.1_. [10] 32. The participants commented that the anonymity _3.2_ of the site was good and for many it was their first time sharing their feelings. [24] 33. (Theme: Privacy _3.2_/feeling comfortable) When you’re answering questions on the iPad, it’s different to talking to a person, so you kind of feel more comfortable. [19] 34. But then [with Appa], I thought, well, it’s like, only 30 minutes, and I can talk to my mentor whenever and the videos I can watch and I can look back on. And I just realized it was a lot more helpful _3.1_ than what I had before. [11] 35. For instance, the app should avoid many open-ended questions in the questionnaire, provide sufficient information about the program (e.g., what participants are asked to do in the app) and sufficient notification to remind the participants to do the exercise or activities that they planned, offer timely responses by e-helpers, and provide additional assurances of confidentiality _3.2_. [9] |
|  | - 1. Privacy/Security |  |
|  | - 1. Time Management |  |
| 1. Supportive Environment | - 1. Technical Environment | 1. Participants across the interviews and groups noted that using digital technologies was a valid approach to engagement, as young people use these in everyday life _4.1_, although there were concerns about those without internet access. [1] 2. They suggested that many young people would prefer it to a live programme as…(iii) ‘the net’s where we spend all our time anyway’ _4.1_…. [5] 3. Common intrapersonal catalysts _4.2_ included recognizing emotional or mood symptoms, shifts from a personal normal, such as diminished desire to engage in activities or lethargy, or, in more extreme cases, hitting a personal low. [3] 4. Some participants described a marked increase in symptoms as hitting a personal low _4.2_, which prompted them look for answers online. [3] 5. Interpersonal changes _4.2_ such as breakups or feeling apathetic towards others, were described as particularly concerning. [3] |
|  | - 1. Interpersonal Catalysts |  |

Barriers

Barriers-External level

| Theme | Sub-theme | Original description and references |
| --- | --- | --- |
| 1. Integration with Schools | —— | 1. However, some young people noted that associating it with schools might make it less appealing _1_. [2] 2. Also, all participants in this study were more likely to visit (MI: 90%, BA: 78%) the Internet program than those in a school based study of a wellness-oriented site _1_ (27%) or in surveys of adolescents (18%). [10] |

Barriers-Intervention level

| Theme | Sub-theme | Original description and references |
| --- | --- | --- |
| 1. Content Gaps | - 1. Cultural/Religious/ Spirituality Issues | 1. All interviewees suggested not having pictures of meditators _1.1_ on the site as it may communicate a religious overtone. [5] 2. The majority of adolescents rated the following factors as at least moderately problematic: the inability to ask questions _1.3_ (n = 128, 61.5%), not finishing the program (n = 116, 55.8%), information being too general (n = 112, 53.8%), being without therapist support _1.2_ (n = 107, 51.5%), and the privacy of personal information (n = 106, 51.0%). [14] 3. Four interviewees suggested avoiding any reference to ‘spirituality’ or even ‘meditation’ _1.1_ as they might think it’s some spiritual bull ... ! [5] 4. Others considered it to be worthwhile, innovative, but not dynamic, as it did not respond to user inputs _1.3_. [9] 5. Nonetheless, most participants reported frustration with the lack of direct human contact _1.2_ and disappointment with the lack of immediate responsiveness _1.3_, articulating an expectation that the iCBT would mimic the responsiveness and immediacy of face-to-face therapy. [12] |
|  | - 1. Support Lacking |  |
|  | - 1. Communication Lacking |  |
| 1. Design Limitations | - 1. Roboticism | 1. In terms of dislikes, the participants referred to the program being boring and too easy to play, having too much talk/writing, being too slow, the need for more customization _2.4_, and the advice being too ‘hard going’ (i.e. difficult to deal with) by focusing unduly on negative aspects of mental health. [7] 2. All young people stated they preferred the illustrative approach to a more photographic _2.2_ one. [2] 3. However, some who had prior experience with mental health services found guided responses ‘robotic’ _2.1_ and said they would prefer greater freedom to compose more personally relevant messages. [22] 4. For instance, the app should avoid many open-ended questions _2.4_ in the questionnaire, provide sufficient information about the program (e.g., what participants are asked to do in the app) and sufficient notification to remind the participants to do the exercise or activities that they planned, offer timely responses by e-helpers, and provide additional assurances of confidentiality. [9] 5. I’m not sure how useful it was because he mentioned that it was like canned responses _2.1_ that he wasn’t able to like add much. [22] 6. All interviewees advised against the use of the word ‘homework’ _2.3_ as it may remind participants of their university or school homework and thus be off putting. [5] 7. Orientation challenges _2.4_——It was kind of confusing to get around, especially. Like if the, it’s not like a tutorial on how to use it. You kind of just turn right into it and like had no idea of what to do initially. [22] 8. However, a quarter (25.0%) of the participants also agreed that it was difficult for them to find their way around the program _2.4_, with another 28.6% of participants agreeing that that the program was ‘a lot of work’ and over a third (35.7%) agreeing that the modules were too long. [7] 9. The majority of adolescents rated the following factors as at least moderately problematic: the inability to ask questions (n = 128, 61.5%), not finishing the program (n = 116, 55.8%), information being too general _2.5_, (n = 112, 53.8%), being without therapist support (n = 107, 51.5%), and the privacy of personal information (n = 106, 51.0%). [14] |
|  | - 1. Multimedia Issues |  |
|  | - 1. Inappropriate Language |  |
|  | - 1. Burden |  |
|  | - 1. Personalisation Lacking |  |
| 1. Low Quality and Effect | - 1. Unattractive | 1. The site’s design was not appealing _3.1_ to me, it looked directed at 10-13-year-olds _3.2_. [23] 2. All students did not find SPARX-R personally helpful. Some of these students explained that this was because of not feeling depressed and, therefore, the contents not being personally relevant _3.2_. [7] 3. Four participants (11%) reported that Bite Back did not seem relevant for them _3.2_. [23] 4. They considered it easy to use, with practical exercises, relatable stories, and engaging illustrated narrations, although a few students considered some scenarios to be less relevant _3.2_ or difficult to understand _3.3_. [9] 5. While some enjoyed the games, others said they were too easy or too slow _3.3_ and preferred more challenging games to be included. [23] 6. In terms of dislikes, the participants referred to the program being boring _3.1_ and too easy to play _3.3_, having too much talk/writing, being too slow _3.3_, the need for more customization, and the advice being too ‘hard going _3.3_’ (i.e. difficult to deal with) by focusing unduly on negative aspects of mental health _3.6_. [7] 7. When the topic of CMHHS for anxiety was introduced by the facilitator (before Chilled Out was shown), many participants seemed unfamiliar with and/or confused _3.4_ by the concept. [18] 8. Barriers to use included not having the app on their own phone (due to it only being available on Android devices for the trial), forgetting about it, its repetitiveness _3.5_, and having better things to do or no time. [15] 9. Others considered it to be worthwhile, innovative, but not dynamic _3.3_, as it did not respond to user inputs. [9] 10. But then afterwards it was all too repetitive and much too the same _3.5_, things I knew already there. [23] 11. The website was very similar each time _3.5_ I visited it and thus lost the initial flair it once had. [23] 12. Furthermore, 6 participants (17%) stated that the content of the website was not sufficient to sustain their interest _3.1_ for an hour a week. [23] 13. Although more than half (55.5%) of the participants considered the program interesting, nearly a half (46.5%) also indicated that they found the program boring _3.1_. [7] 14. Some of the questions are hard _3.3_ and I didn’t understand _3.4_ them. [19] 15. Fewer adolescents considered losing interest (n = 95, 45.7%), finding time (n = 88, 42.4%), computer problems (n = 81, 39.0%), computer access (n = 80, 38.5%), and difficulty _3.3_ of tasks (n = 78, 37.5%) to be problematic. [14] 16. Only a small minority had any objections to YouthCHAT questioning (e.g. questions too hard _3.3_, too difficult _3.3_ or too many), and all in fact completed the entire questionnaire. [19] 17. Almost a half (42.8%) of the participants had felt annoyed _3.4_ or frustrated _3.4_ going through the program. [7] 18. Orientation challenges——It was kind of confusing _3.4_ to get around, especially. Like if the, it’s not like a tutorial on how to use it. You kind of just turn right into it and like had no idea of what to do initially _3.4_. [22] 19. Others suggested that not knowing what else to do _3.4_ once they had finished all the quests prevented them from using the app again. [15] 20. All interviewees advised against the use of the word ‘homework’ as it may remind participants of their university or school homework and thus be off putting _3.4_. [5] 21. Four interviewees suggested avoiding any reference to ‘spirituality’ or even ‘meditation’ as they might think it’s some spiritual bull _3.4_ ... ! [5] 22. A minority (10.7%) indicated that the program was upsetting _3.4_ and a quarter (25%) stated that they were worried about privacy. [7] 23. Focusing on depression encourages negative feelings _3.4_ [7] 24. It annoys me as it doesn’t allow you to be happy _3.4_ [7] 25. “The content is too rigid _3.3_. It will make people feel bored and irritable _3.4_ if used for a long time” [21] |
|  | - 1. Irrelevancy |  |
|  | - 1. Poor Using Experience |  |
|  | - 1. Poor Emotional Experiences |  |
|  | - 1. Repetitiveness |  |
|  | - 1. Low Effect |  |
| 1. Inappropriate Duration and Schedule | - 1. Inappropriate Duration | 1. However, a quarter (25.0%) of the participants also agreed that it was difficult for them to find their way around the program, with another 28.6% of participants agreeing that that the program was ‘a lot of work’ and over a third (35.7%) agreeing that the modules were too long _4.1_. [7] 2. All interviewees preferred a series of short videos each week rather than one long presentation _4.1_. [5] 3. Only a small minority had any objections to YouthCHAT questioning (e.g. questions too hard, too difficult or too many _4.1_), and all in fact completed the entire questionnaire. [19] 4. In terms of dislikes, the participants referred to the program being boring and too easy to play, having too much talk/writing _4.1_, being too slow, the need for more customization, and the advice being too ‘hard going’ (i.e. difficult to deal with) by focusing unduly on negative aspects of mental health. [7] 5. They suggested that many young people would prefer it to a live programme as (i) the latter would take too much time _4.1_ [5] 6. Other concerns raised by the users about the app were that the audio exercise was too long _4.1_ that the user would get bored, app notifications were not frequent enough or occurred at an unwanted time _4.2_. [9] |
|  | - 1. Inappropriate Schedule |  |
| 1. Inaccessibility | - 1. Technological Issues | 1. Technological issues _5.1_ [22]——There was a little hitch up with the signup but once we got around that it was, yeah smooth sailing.” (Buddy 11, friend) / “The only kind of thing that kind of sucked was how you only get notifications whilst the app was open and not when it was closed.” / “Another one I found issue within the app is friend requests. It was really confusing.” / “Occasionally actually it would log me out. I’m not sure why but sometimes I get logged out and have to relog in but that’s just a small thing.” 2. Although most users and buddies found Village appealing to use and easy to navigate, a few experienced difficulties downloading or using the app for the first time _5.1_. [22] 3. Barriers to use included not having the app on their own phone (due to it only being available on Android devices for the trial) _5.1_, forgetting about it, its repetitiveness, and having better things to do or no time. [15] 4. The key words extracted from technology theme are “glitches”, “lag”, “system”, “crash” and “inflexible” _5.1_, with an example: “crashed when I just entered the interface, and some glitches need to be optimized”, mainly focused on the evaluation of technology, the theme named “technology”. [21] 5. Not surprisingly, many participants cited financial barriers _5.2_ to existing services and said they would be more willing to try services if they were free. [3] 6. In total, 2 of these 3 participants cited high costs _5.2_ associated with mental health services as a major barrier. [11] |
|  | - 1. High Cost |  |

Barriers-Individual level

| Theme | Sub-theme | Original description and references |
| --- | --- | --- |
| 1. Detrimental Characteristics | - 1. Physically Unwell | 1. The most common reasons for non-completion were technical glitches, lack of time, lack of interest, not finding the resource helpful, or being physically unwell and unable to attend appointments _1.1_. [20] 2. In many cases, they lacked confidence _1.2_ in their own ability to work therapeutically via the internet. [13] 3. Participants’ reasons for not downloading the app were as follows, from most to least frequent: no motivation, not useful, forgot to download it, high storage requirement, missed the lesson with download instructions, no time to use the app, no one to connect to _1.3_, and privacy concerns. [27] |
|  | - 1. Lack of Confidence |  |
|  | - 1. Lack of Connection |  |
| 1. Motivational Challenges | - 1. No Motivation | - - 1. Although 51.5% of girls reported that they would use an app if they had a mental health problem, only 16.1% of had actually used one, and 44.3% of this symptomatic group did not think an app would help them and/or expressed a preference for face-to-face help _2.2_. [25]     2. Given the choice, three quarters of young people would prefer to meet face to face and talk with someone _2.2_, with less than a quarter expressing a positive preference to use a computerised programme. [26]     3. Lack of control over CMHHS decision-making _2.2_ [18]     4. Participants also highlighted concerns about whether the decisions relating to CMHHS were within the program user’s control _2.2_. [18]     5. Participants’ reasons for not downloading the app were as follows, from most to least frequent: no motivation _2.1_, not useful, forgot to download it, high storage requirement, missed the lesson with download instructions, no time to use the app, no one to connect to, and privacy concerns. [27]     6. Most participants stated that they preferred accessing the site on smartphones instead of computers _2.2_, although they recognized that sometimes they have difficulties accessing the internet. [6]     7. We found that, overall, participants had reservations about human-like support from a messaging system _2.3_, and they therefore laid out a number of constraints for sending supportive messages, as well as suggesting alternative ways the system might sustain their motivation. [16] |
|  | - 1. Preferences |  |
|  | - 1. Negative Attitudes/Beliefs |  |
| 1. Perceived Risks | - 1. Privacy/Security/   Credibility Concerns | 1. The majority of adolescents rated the following factors as at least moderately problematic: the inability to ask questions (n = 128, 61.5%), not finishing the program (n = 116, 55.8%), information being too general (n = 112, 53.8%), being without therapist support (n = 107, 51.5%), and the privacy of personal information _3.1_ (n = 106, 51.0%). [14] 2. Participants’ reasons for not downloading the app were as follows, from most to least frequent: no motivation, not useful, forgot to download it, high storage requirement, missed the lesson with download instructions, no time to use the app, no one to connect to, and privacy concerns _3.1_. [27] 3. One interviewee pointed out that young people may be reluctant to undertake the programme because of concerns about privacy _3.1_ (e.g. when using a family or other public computer). [5] 4. During testing sessions young people in particular emphasized concerns about online privacy and credibility _3.1_. [17] 5. Even when some assurances could be made as to the confidential nature of some CMHHS (such as security systems used in computerised treatment programs), participants remained concerned as to how “private” CMHHS would be _3.1_. [18] 6. A minority (10.7%) indicated that the program was upsetting and a quarter (25%) stated that they were worried about privacy _3.1_. [7] 7. In regards to the former, participants expressed concerns relating to the “anonymity” _3.1_ of help-seeking and the possibility that help-seeking would involve exposure to mental health stigma _3.2_. [18] 8. According to this theme, help-seeking was conceptualised as involving “risk,” more specifically exposure to peer stigma and cyber bullying _3.2_. [18] 9. Security _3.1_ and confidentiality _3.1_ were key issues in the interviews and groups, especially for young people. [1] |
|  | - 1. Stigma and Cyber Bullying Concerns |  |
| 1. Question | - 1. Question the Helpfulness | 1. I don’t believe they would help me _4.1_. [25] 2. Some participants questioned the validity _4.2_ of the Web site, and how sharing problems or reading about other people’s problems could be of benefit. [24] 3. Although 51.5% of girls reported that they would use an app if they had a mental health problem, only 16.1% of had actually used one, and 44.3% of this symptomatic group did not think an app would help them _4.1_ and/or expressed a preference for face-to-face help. [25] 4. The most common reasons for non-completion were technical glitches, lack of time, lack of interest, not finding the resource helpful _4.1_, or being physically unwell and unable to attend appointments. [20] 5. Participants’ reasons for not downloading the app were as follows, from most to least frequent: no motivation, not useful _4.3_, forgot to download it, high storage requirement, missed the lesson with download instructions, no time to use the app, no one to connect to, and privacy concerns. [27] 6. I’m not sure how useful it was _4.3_ because he mentioned that it was like canned responses that he wasn’t able to like add much. [22] |
|  | - 1. Question the Validity |  |
|  | - 1. Question the Usefulness |  |
| 1. Retention Issues | - 1. Low Priority | 1. Barriers to use included not having the app on their own phone (due to it only being available on Android devices for the trial), forgetting about it _5.1_, its repetitiveness, and having better things to do _5.1_ or no time. [15] 2. Participants’ reasons for not downloading the app were as follows, from most to least frequent: no motivation, not useful, forgot to download it _5.1_, high storage requirement, missed the lesson with download instructions _5.1_, no time to use the app, no one to connect to, and privacy concerns. [27] 3. Fewer adolescents considered losing interest _5.2_ (n = 95, 45.7%), finding time (n = 88, 42.4%), computer problems (n = 81, 39.0%), computer access (n = 80, 38.5%), and difficulty of tasks (n = 78, 37.5%) to be problematic. [14] 4. The most common reasons for non-completion were technical glitches, lack of time, lack of interest _5.2_, not finding the resource helpful, or being physically unwell and unable to attend appointments. [20] 5. The majority of adolescents rated the following factors as at least moderately problematic: the inability to ask questions (n = 128, 61.5%), not finishing the program _5.3_ (n = 116, 55.8%), information being too general (n = 112, 53.8%), being without therapist support (n = 107, 51.5%), and the privacy of personal information (n = 106, 51.0%). [14] 6. Ten interviewees commented that although the idea of learning mindfulness skills is likely to appeal to many young people, persevering in the programme to the end and completing home practices were likely to prove difficult _5.3_. [5] 7. Participants said that the intervention was time-consuming and sustained mental effort was required to engage with the online content, making it difficult to persist _5.3_ with treatment and to stay motivated to complete the intervention. [12] |
|  | - 1. Low Interest |  |
|  | - 1. Cannot Preserve |  |
| 1. No/Limited Time | —— | 1. Barriers to use included not having the app on their own phone (due to it only being available on Android devices for the trial), forgetting about it, its repetitiveness, and having better things to do or no time _6_. [15] 2. Of the 36 participants who responded, 21 (58%) cited that the reason for their underusage was time constraints _6_. [23] 3. Fewer adolescents considered losing interest (n = 95, 45.7%), finding time _6_  (n = 88, 42.4%), computer problems (n = 81, 39.0%), computer access (n = 80, 38.5%), and difficulty of tasks (n = 78, 37.5%) to be problematic. [14] 4. The most common reasons for non-completion were technical glitches, lack of time _6_, lack of interest, not finding the resource helpful, or being physically unwell and unable to attend appointments. [20] 5. Participants’ reasons for not downloading the app were as follows, from most to least frequent: no motivation, not useful, forgot to download it, high storage requirement, missed the lesson with download instructions, no time to use the app _6_, no one to connect to, and privacy concerns. [27] 6. Participants said that the intervention was time-consuming _6_ and sustained mental effort was required to engage with the online content, making it difficult to persist with treatment and to stay motivated to complete the intervention. [12] |
| 1. Technical Issues | —— | 1. Fewer adolescents considered losing interest (n = 95, 45.7%), finding time (n = 88, 42.4%), computer problems _7_ (n = 81, 39.0%), computer access _7_ (n = 80, 38.5%), and difficulty of tasks (n = 78, 37.5%) to be problematic. [14] 2. Technical issues _7_ accounted for 5 participants’ (14%) underusage, predominantly issues with Internet access. [23] 3. Participants’ reasons for not downloading the app were as follows, from most to least frequent: no motivation, not useful, forgot to download it, high storage requirement _7_, missed the lesson with download instructions, no time to use the app, no one to connect to, and privacy concerns. [27] |

References

1. Bevan Jones R, Thapar A, Rice F, Beeching H, Cichosz R, Mars B, et al. A Web-Based Psychoeducational Intervention for Adolescent Depression: Design and Development of MoodHwb. JMIR Ment Health. 2018 Feb 15;5(1):e13. PMID: 29449202. doi: 10.2196/mental.8894.

2. Bevan Jones R, Thapar A, Rice F, Mars B, Agha SS, Smith D, et al. A Digital Intervention for Adolescent Depression (MoodHwb): Mixed Methods Feasibility Evaluation. JMIR Ment Health. 2020 Jul 17;7(7):e14536. PMID: 32384053. doi: 10.2196/14536.

3. Kruzan KP, Meyerhoff J, Nguyen T, Mohr DC, Reddy M, Kornfield R. "I Wanted to See How Bad it Was": Online Self-screening as a Critical Transition Point Among Young Adults with Common Mental Health Conditions. Proc SIGCHI Conf Hum Factor Comput Syst. 2022 Apr;2022. PMID: 35531062. doi: 10.1145/3491102.3501976.

4. Sawrikar V, Mote K. Technology acceptance and trust: Overlooked considerations in young people's use of digital mental health interventions. Health Policy and Technology. 2022;11(4). doi: 10.1016/j.hlpt.2022.100686.

5. Monshat K, Vella-Brodrick D, Burns J, Herrman H. Mental health promotion in the Internet age: a consultation with Australian young people to inform the design of an online mindfulness training programme. Health Promot Int. 2012 Jun;27(2):177-86. PMID: 21398335. doi: 10.1093/heapro/dar017.

6. Martinez V, Espinosa-Duque D, Jimenez-Molina A, Rojas G, Vohringer PA, Fernandez-Arcila M, et al. Feasibility and Acceptability of "Cuida tu Animo" (Take Care of Your Mood): An Internet-Based Program for Prevention and Early Intervention of Adolescent Depression in Chile and Colombia. Int J Environ Res Public Health. 2021 Sep 13;18(18). PMID: 34574553. doi: 10.3390/ijerph18189628.

7. Kuosmanen T, Fleming TM, Barry MM. The implementation of SPARX-R computerized mental health program in alternative education: Exploring the factors contributing to engagement and dropout. Children and Youth Services Review. 2018;84:176-84. doi: 10.1016/j.childyouth.2017.11.032.

8. Suffoletto B, Goldstein T, Gotkiewicz D, Gotkiewicz E, George B, Brent D. Acceptability, Engagement, and Effects of a Mobile Digital Intervention to Support Mental Health for Young Adults Transitioning to College: Pilot Randomized Controlled Trial. JMIR Form Res. 2021 Oct 14;5(10):e32271. PMID: 34647893. doi: 10.2196/32271.

9. Sit HF, Hong IW, Burchert S, Sou EKL, Wong M, Chen W, et al. A Feasibility Study of the WHO Digital Mental Health Intervention Step-by-Step to Address Depression Among Chinese Young Adults. Front Psychiatry. 2021;12:812667. PMID: 35069297. doi: 10.3389/fpsyt.2021.812667.

10. Van Voorhees BW, Fogel J, Pomper BE, Marko M, Reid N, Watson N, et al. Adolescent Dose and Ratings of an Internet-Based Depression Prevention Program: A Randomized Trial of Primary Care Physician Brief Advice versus a Motivational Interview. J Cogn Behav Psychother. 2009;9(1):1-19. PMID: 20694059.

11. Giovanelli A, Sanchez Karver T, Roundfield KD, Woodruff S, Wierzba C, Wolny J, et al. The Appa Health App for Youth Mental Health: Development and Usability Study. JMIR Form Res. 2023 Oct 4;7:e49998. PMID: 37792468. doi: 10.2196/49998.

12. Gericke F, Ebert DD, Breet E, Auerbach RP, Bantjes J. A qualitative study of university students’ experience of Internet-based CBT for depression. Counselling and Psychotherapy Research. 2021;21(4):792-804. doi: 10.1002/capr.12465.

13. Lilja JL, Rupcic Ljustina M, Nissling L, Larsson AC, Weineland S. Youths' and Parents' Experiences and Perceived Effects of Internet-Based Cognitive Behavioral Therapy for Anxiety Disorders in Primary Care: Mixed Methods Study. JMIR Pediatr Parent. 2021 Nov 1;4(4):e26842. PMID: 34723830. doi: 10.2196/26842.

14. Sweeney GM, Donovan CL, March S, Forbes Y. Logging into therapy: Adolescent perceptions of online therapies for mental health problems. Internet Interv. 2019 Mar;15:93-9. PMID: 30792959. doi: 10.1016/j.invent.2016.12.001.

15. Dingwall KM, Povey J, Sweet M, Friel J, Shand F, Titov N, et al. Feasibility and Acceptability of the Aboriginal and Islander Mental Health Initiative for Youth App: Nonrandomized Pilot With First Nations Young People. JMIR Hum Factors. 2023 Jun 7;10:e40111. PMID: 37285184. doi: 10.2196/40111.

16. Kornfield R, Meyerhoff J, Studd H, Bhattacharjee A, Williams JJ, Reddy M, et al. Meeting Users Where They Are: User-centered Design of an Automated Text Messaging Tool to Support the Mental Health of Young Adults. Proc SIGCHI Conf Hum Factor Comput Syst. 2022 Apr;2022. PMID: 35574512. doi: 10.1145/3491102.3502046.

17. Wozney L, Baxter P, Newton AS. Usability evaluation with mental health professionals and young people to develop an Internet-based cognitive-behaviour therapy program for adolescents with anxiety disorders. BMC Pediatr. 2015 Dec 16;15:213. PMID: 26675420. doi: 10.1186/s12887-015-0534-1.

18. Clark LH, Hudson JL, Dunstan DA, Clark GI. Capturing the Attitudes of Adolescent Males’ Towards Computerised Mental Health Help-Seeking. Australian Psychologist. 2020;53(5):416-26. doi: 10.1111/ap.12341.

19. Goodyear-Smith F, Corter A, Suh H. Electronic screening for lifestyle issues and mental health in youth: a community-based participatory research approach. BMC Med Inform Decis Mak. 2016 Nov 8;16(1):140. PMID: 27821128. doi: 10.1186/s12911-016-0379-z.

20. Merry SN, Stasiak K, Shepherd M, Frampton C, Fleming T, Lucassen MF. The effectiveness of SPARX, a computerised self help intervention for adolescents seeking help for depression: randomised controlled non-inferiority trial. BMJ. 2012 Apr 18;344:e2598. PMID: 22517917. doi: 10.1136/bmj.e2598.

21. He Y, Yang L, Zhu X, Wu B, Zhang S, Qian C, et al. Mental Health Chatbot for Young Adults With Depressive Symptoms During the COVID-19 Pandemic: Single-Blind, Three-Arm Randomized Controlled Trial. J Med Internet Res. 2022 Nov 21;24(11):e40719. PMID: 36355633. doi: 10.2196/40719.

22. Thabrew H, Kumar H, Steadman E. Acceptability and Feasibility of “Village,” a Digital Communication App for Young People Experiencing Low Mood, Thoughts of Self-harm, and Suicidal Ideation to Obtain Support From Family and Friends: Mixed Methods Pilot Open Trial. JMIR Form Res. 2023 Mar 13;7:e41273. PMID: 36912882. doi: 10.2196/41273.

23. Manicavasagar V, Horswood D, Burckhardt R, Lum A, Hadzi-Pavlovic D, Parker G. Feasibility and effectiveness of a web-based positive psychology program for youth mental health: randomized controlled trial. J Med Internet Res. 2014 Jun 4;16(6):e140. PMID: 24901900. doi: 10.2196/jmir.3176.

24. Horgan A, McCarthy G, Sweeney J. An evaluation of an online peer support forum for university students with depressive symptoms. Arch Psychiatr Nurs. 2013 Apr;27(2):84-9. PMID: 23540518. doi: 10.1016/j.apnu.2012.12.005.

25. Grist R, Cliffe B, Denne M, Croker A, Stallard P. An online survey of young adolescent girls' use of the internet and smartphone apps for mental health support. BJPsych Open. 2018 Jul;4(4):302-6. PMID: 30083383. doi: 10.1192/bjo.2018.43.

26. Stallard P, Velleman S, Richardson T. Computer Use and Attitudes Towards Computerised Therapy Amongst Young People and Parents Attending Child and Adolescent Mental Health Services. Child Adolesc Ment Health. 2010 May;15(2):80-4. PMID: 32847246. doi: 10.1111/j.1475-3588.2009.00540.x.

27. Birrell L, Debenham J, Furneaux-Bate A, Prior K, Spallek S, Thornton L, et al. Evaluating a Peer-Support Mobile App for Mental Health and Substance Use Among Adolescents Over 12 Months During the COVID-19 Pandemic: Randomized Controlled Trial. J Med Internet Res. 2023 Sep 27;25:e45216. PMID: 37756116. doi: 10.2196/45216.
