# Supplementary material for: Facilitators and Barriers to Digital Mental Health Interventions for Depression, Anxiety, and Stress in Adolescents and Young Adults: Scoping Review
Source: J Med Internet Res. 2025 Mar 24;27:e62870. doi: 10.2196/62870 (PMC11988281; doi:10.2196/62870)
Supplement: Multimedia Appendix 5 [file jmir_v27i1e62870_app5.docx]

SUPPLEMENTARY FIGURES OF METAPROP

Completely Non-Portable Devices (CNPD)

Facilitators


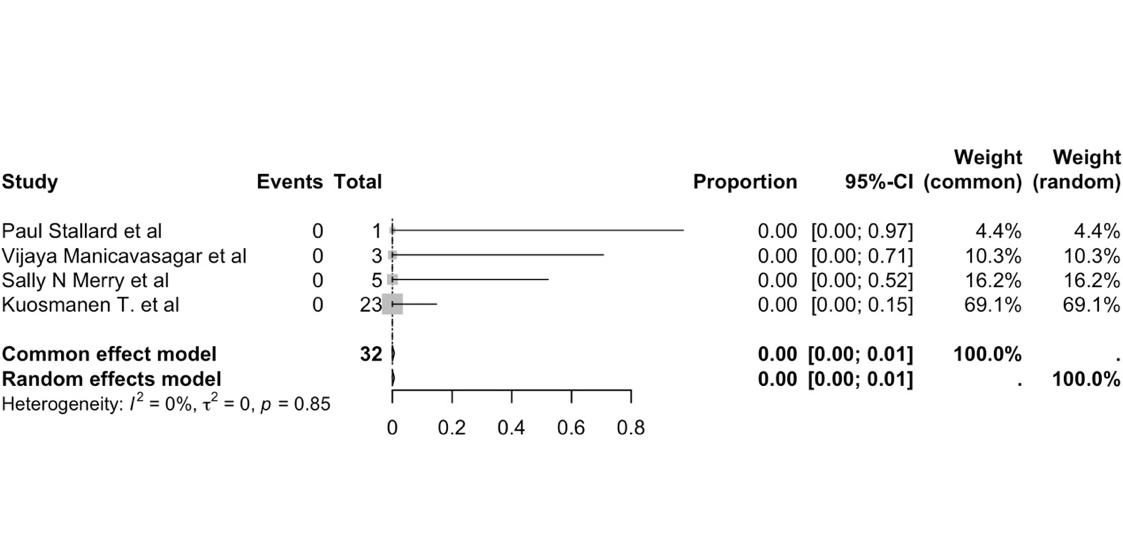


Figure 1a. Proportion of Integration with Schools and Other Resources (F1), Social Norms (F2), Strategic Marketing (F3), Endorsements (F5), Beneficial Characteristics (F11), Supportive Environment (F14) in CNPD group


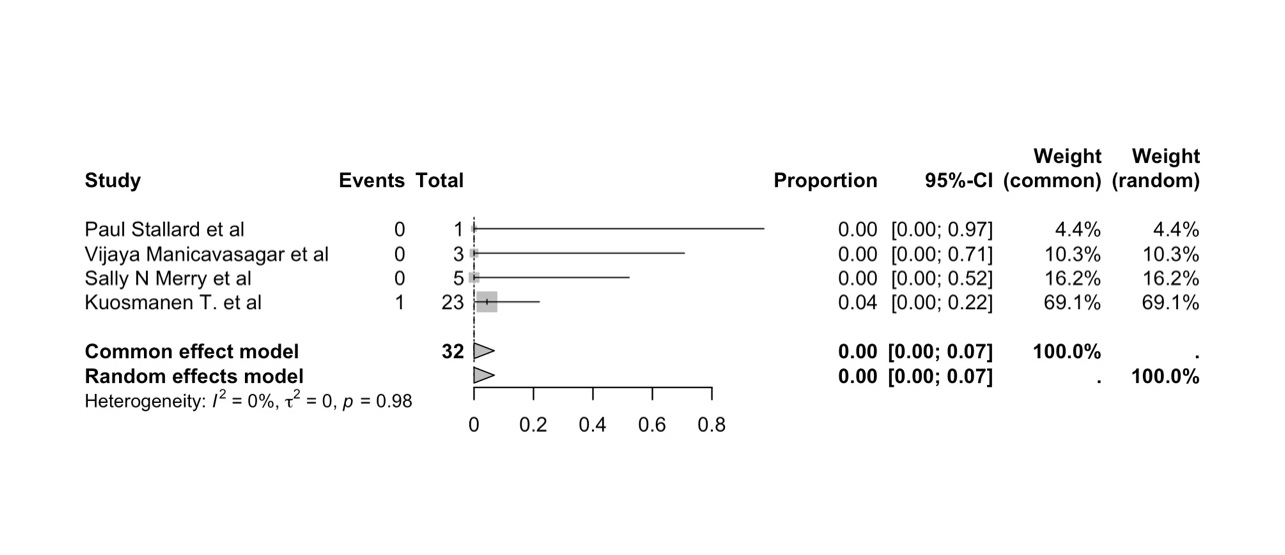


Figure 1b. Proportion of Universality (F4) in CNPD group


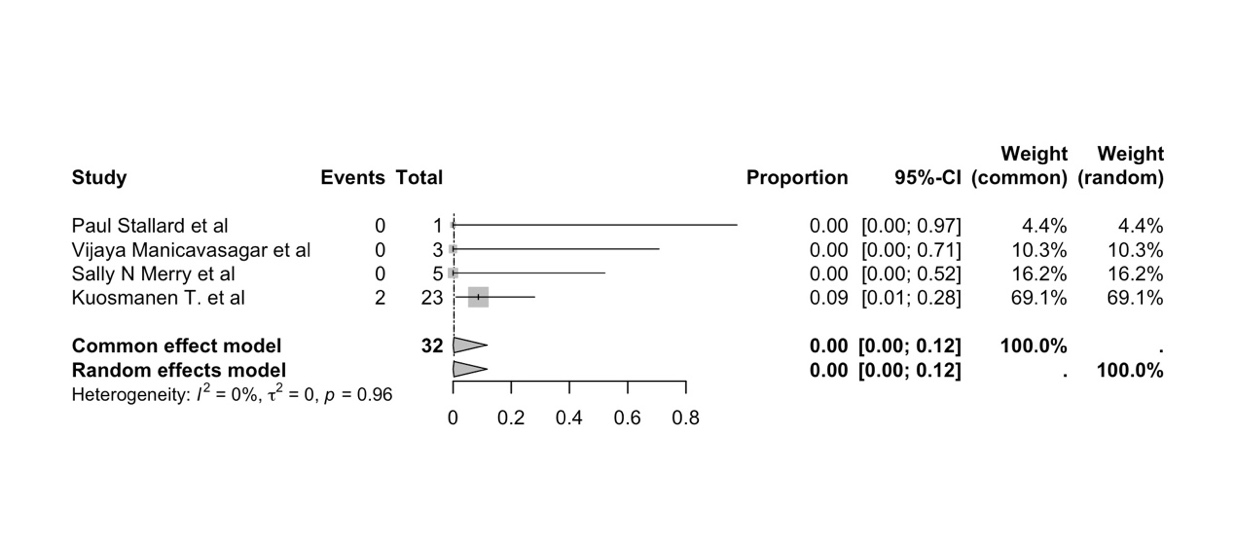


Figure 1c. Proportion of Content Engagement (F6), Design Harmony (F7) in CNPD group


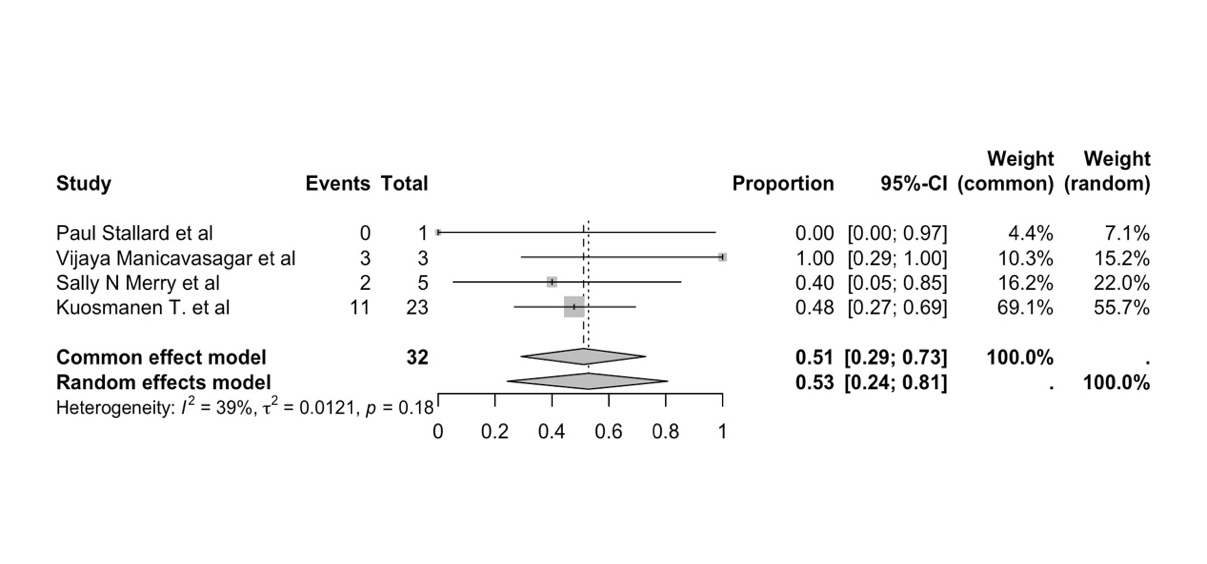


Figure 1d. Proportion of High Quality and Effect (F8) in CNPD group


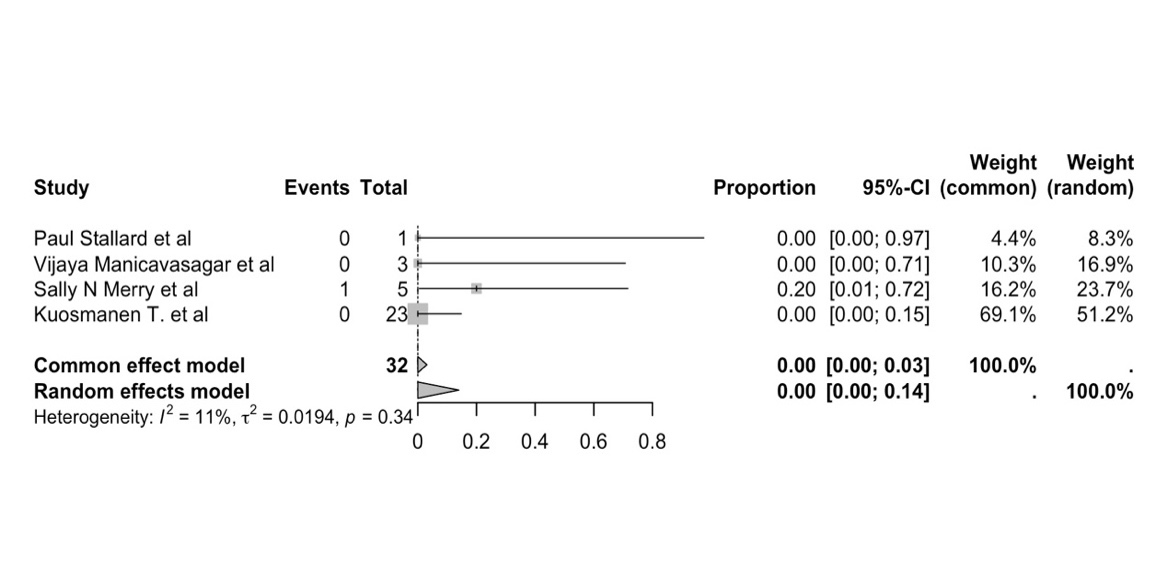


Figure 1e. Proportion of Appropriate Duration and Schedule (F9) in CNPD group


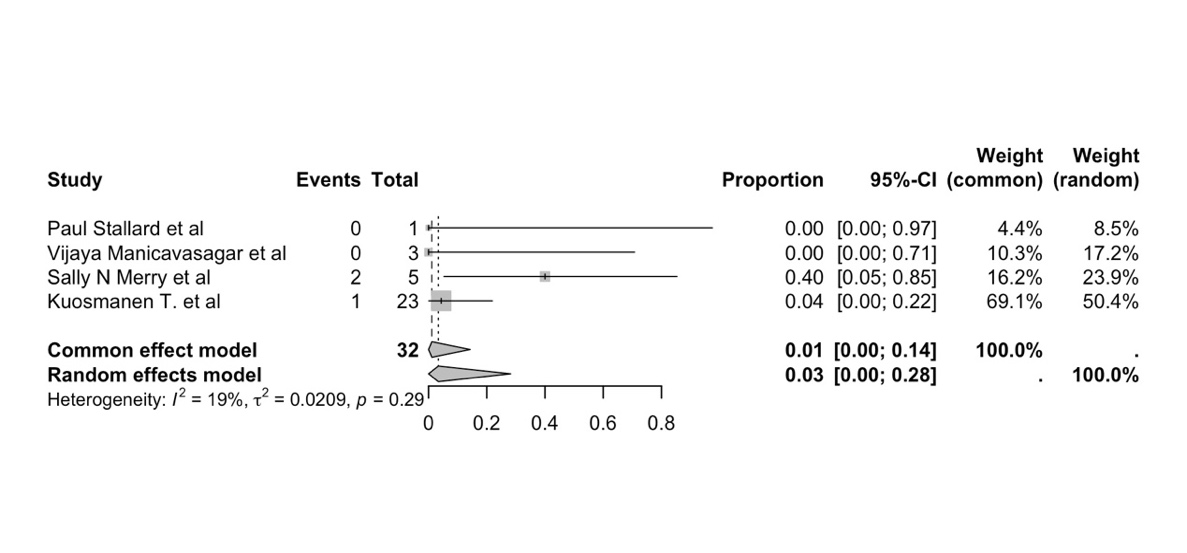


Figure 1f. Proportion of Accessibility (F10) in CNPD group


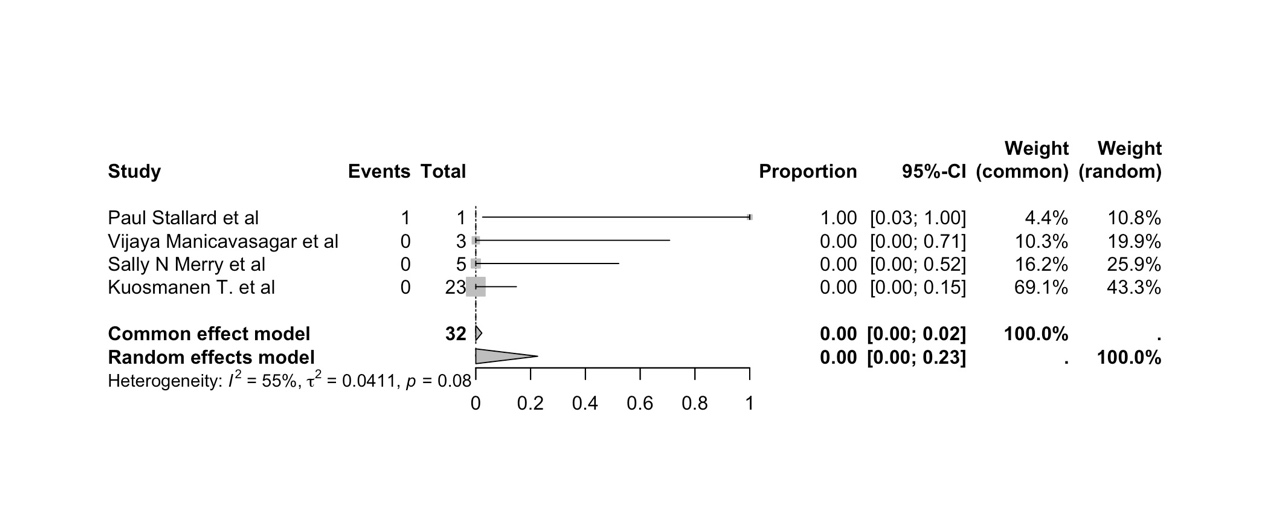


Figure 1g. Proportion of Needs and Disposition (F12) in CNPD group


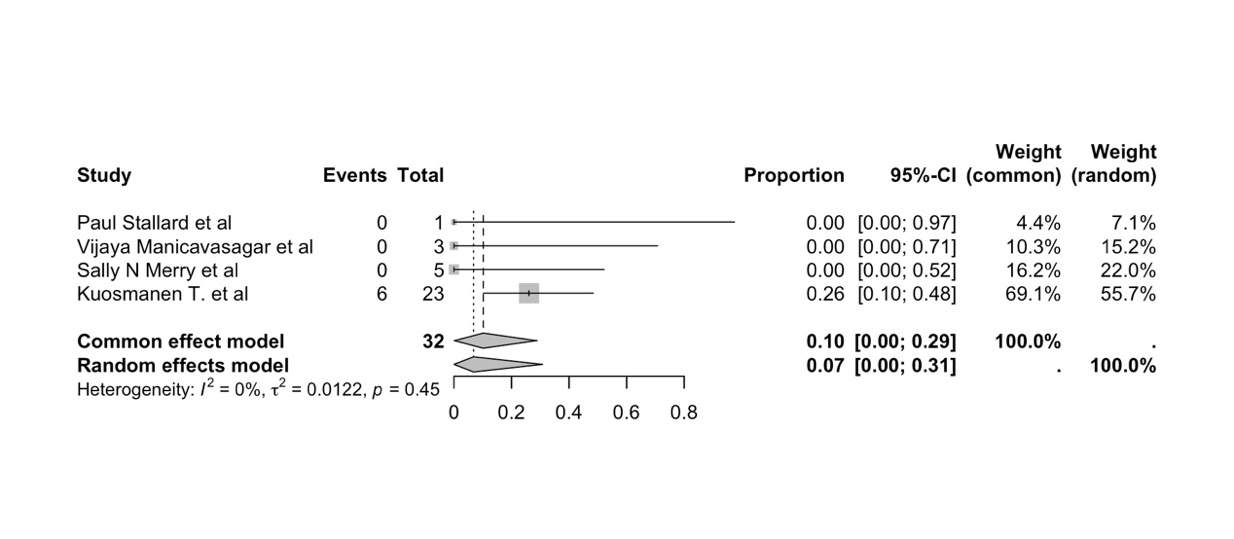


Figure 1h. Proportion of Perceived Benefits (F13) in CNPD group

Completely Non-Portable Devices (CNPD)

Barriers


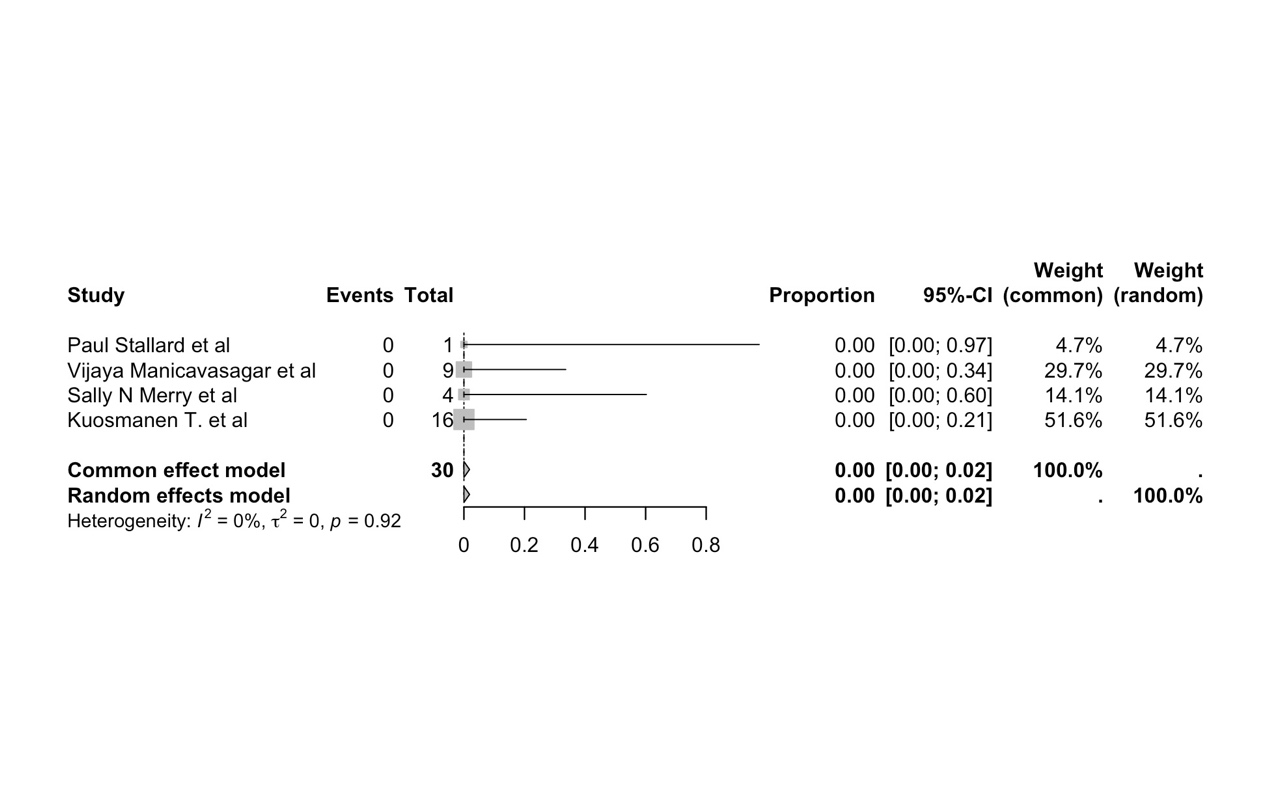


Figure 2a. Proportion of Integration with Schools (B1), Content Gaps (B2), Inaccessibility (B6) in CNPD group


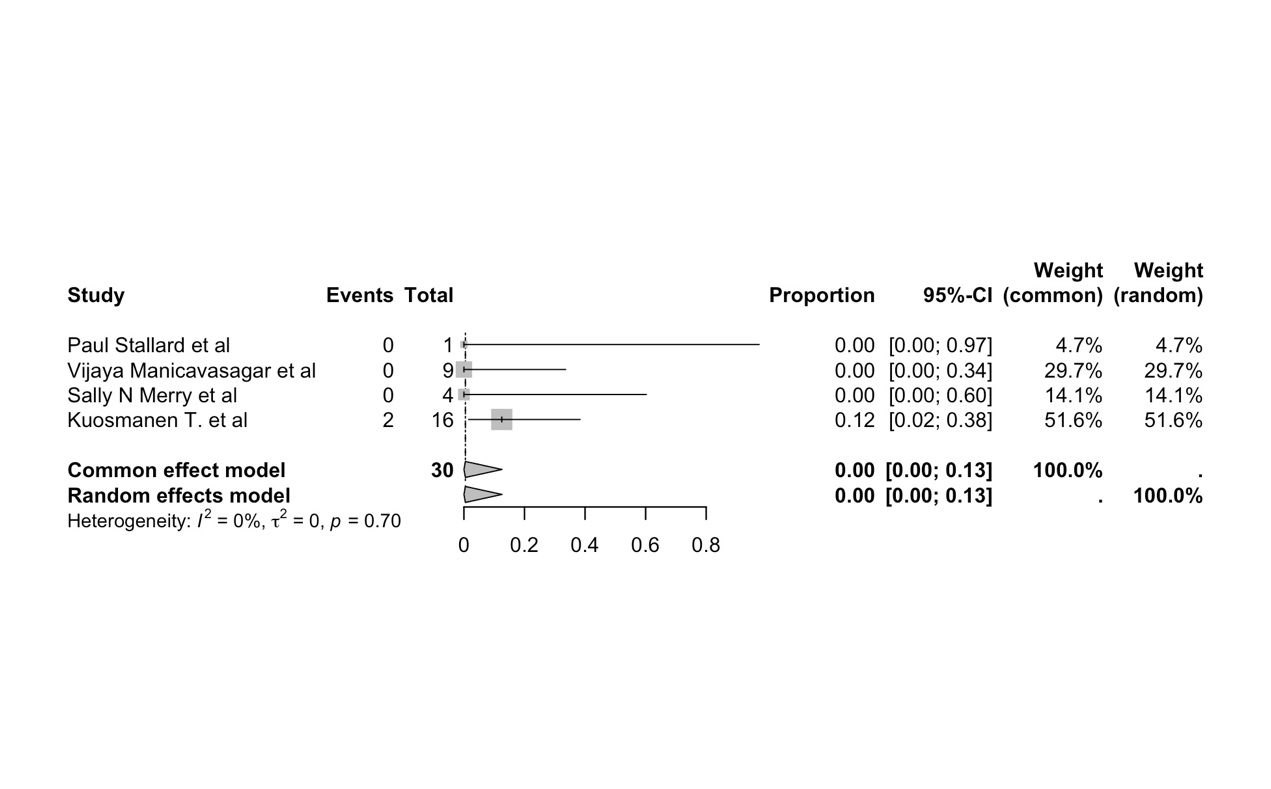


Figure 2b. Proportion of Design Limitations (B3) in CNPD group


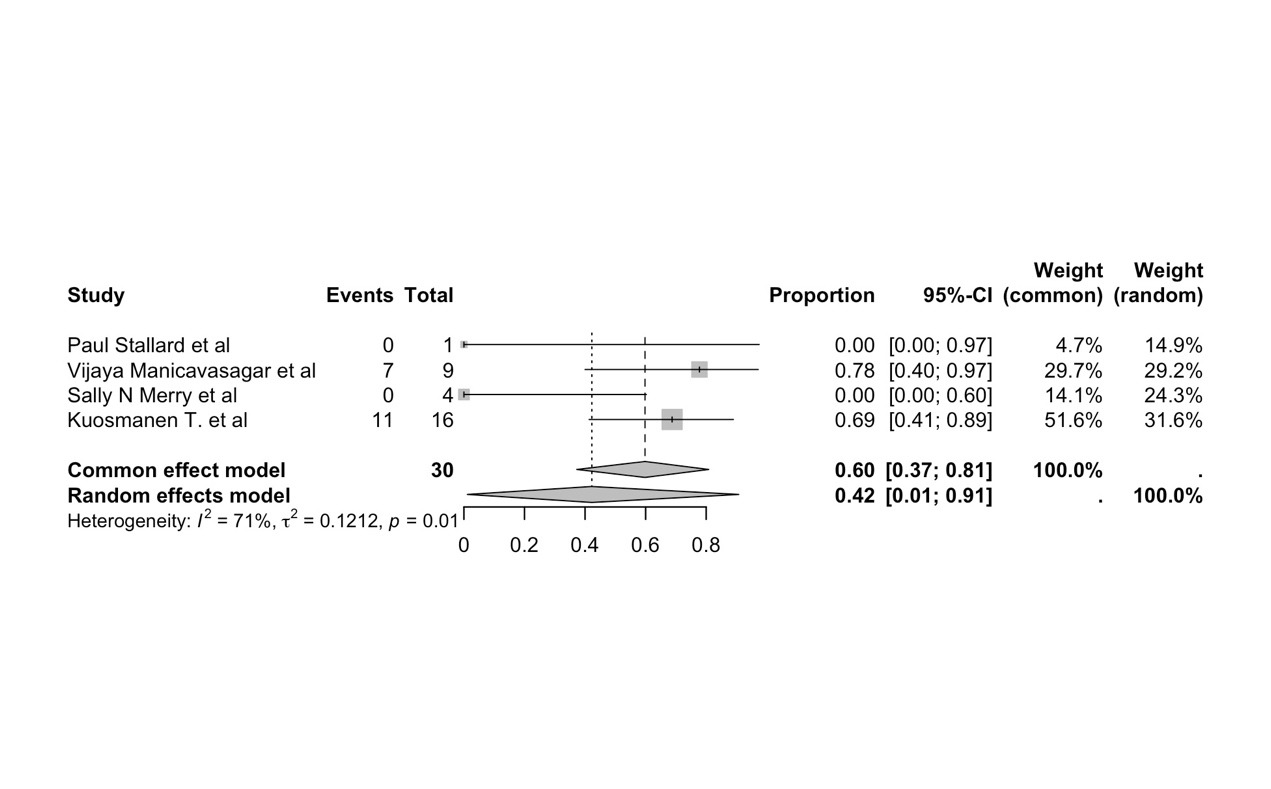


Figure 2c. Proportion of Low Quality and Effect (B4) in CNPD group


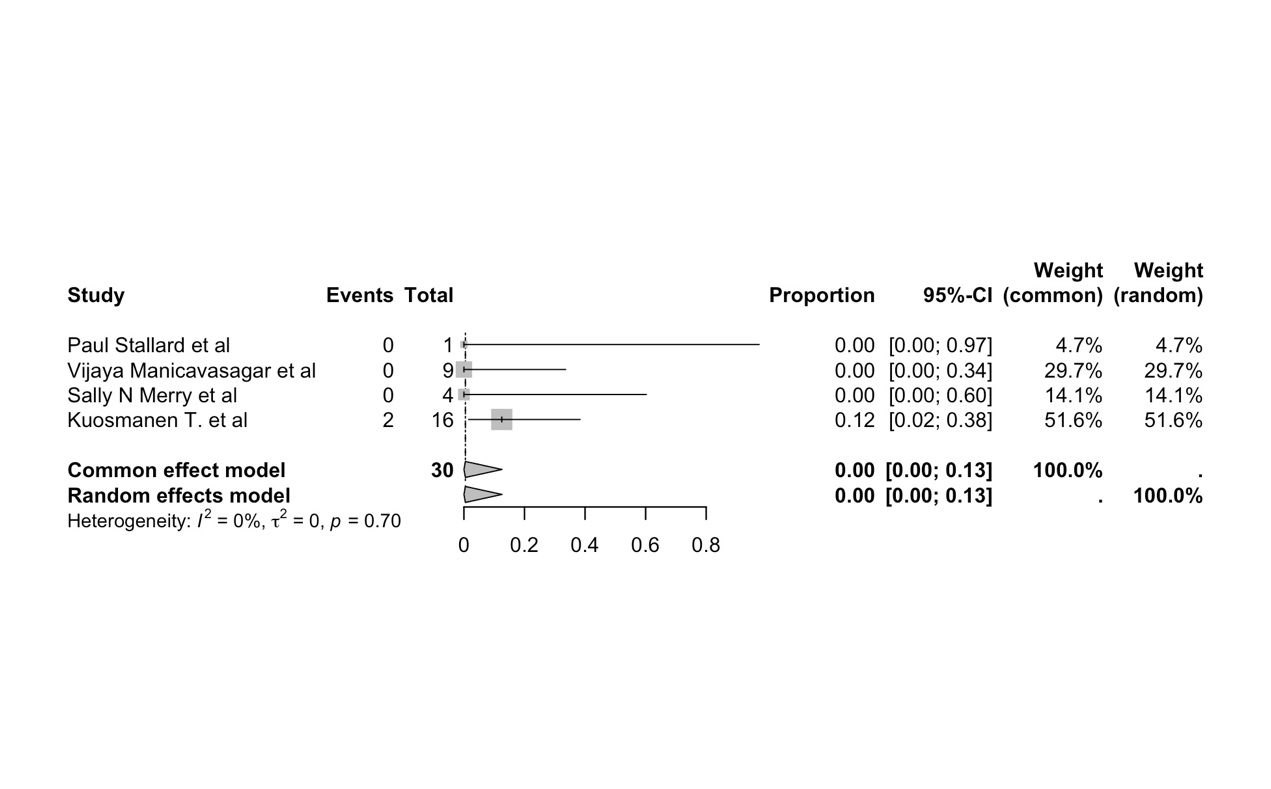


Figure 2d. Proportion of Inappropriate Duration and Schedule (B5) in CNPD group


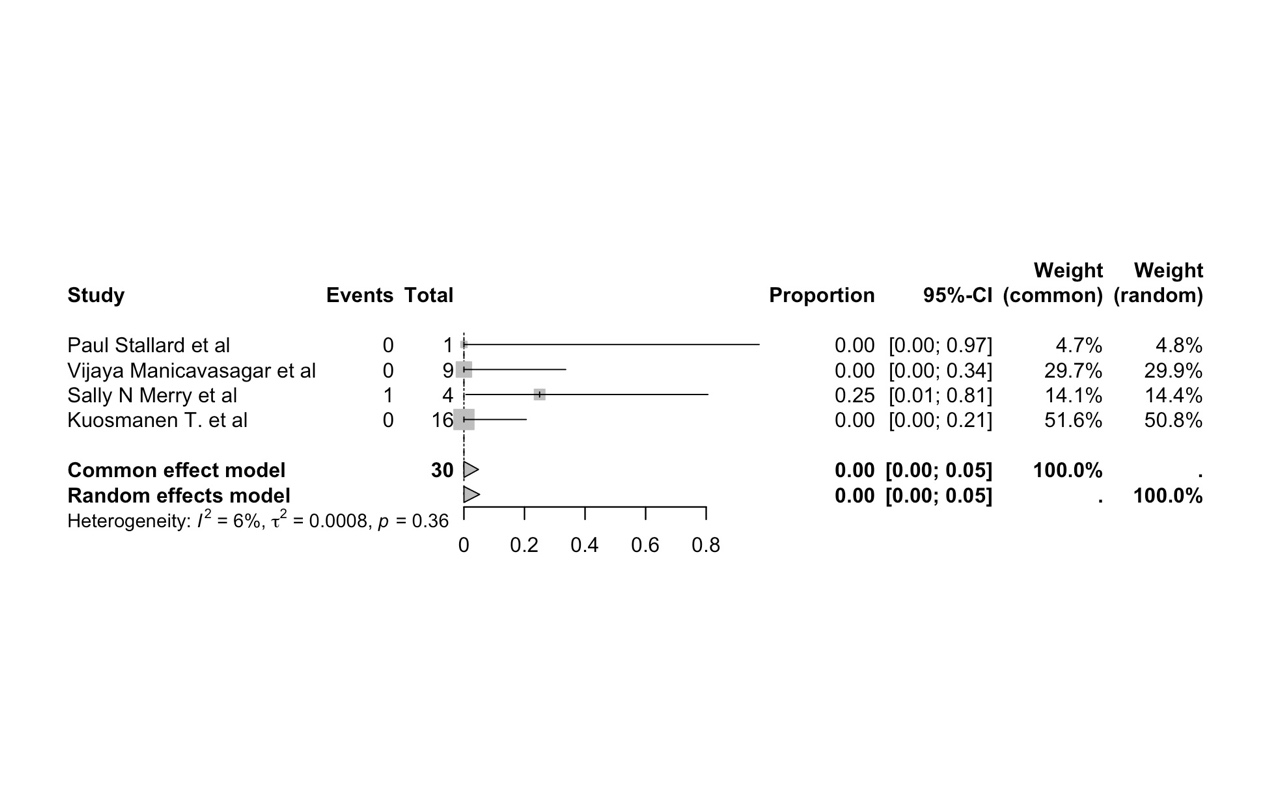


Figure 2e. Proportion of Detrimental Characteristics (B7) in CNPD group


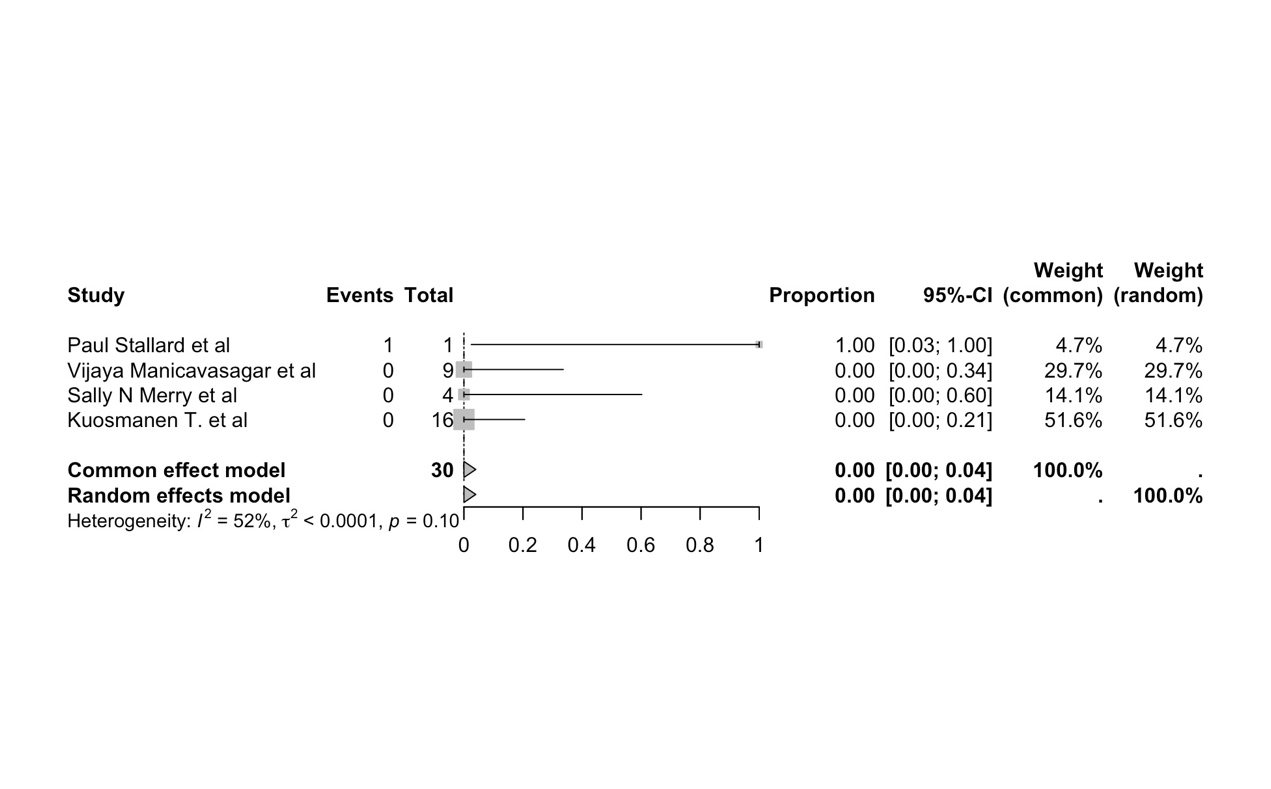


Figure 2f. Proportion of Motivational Challenges (B8) in CNPD group


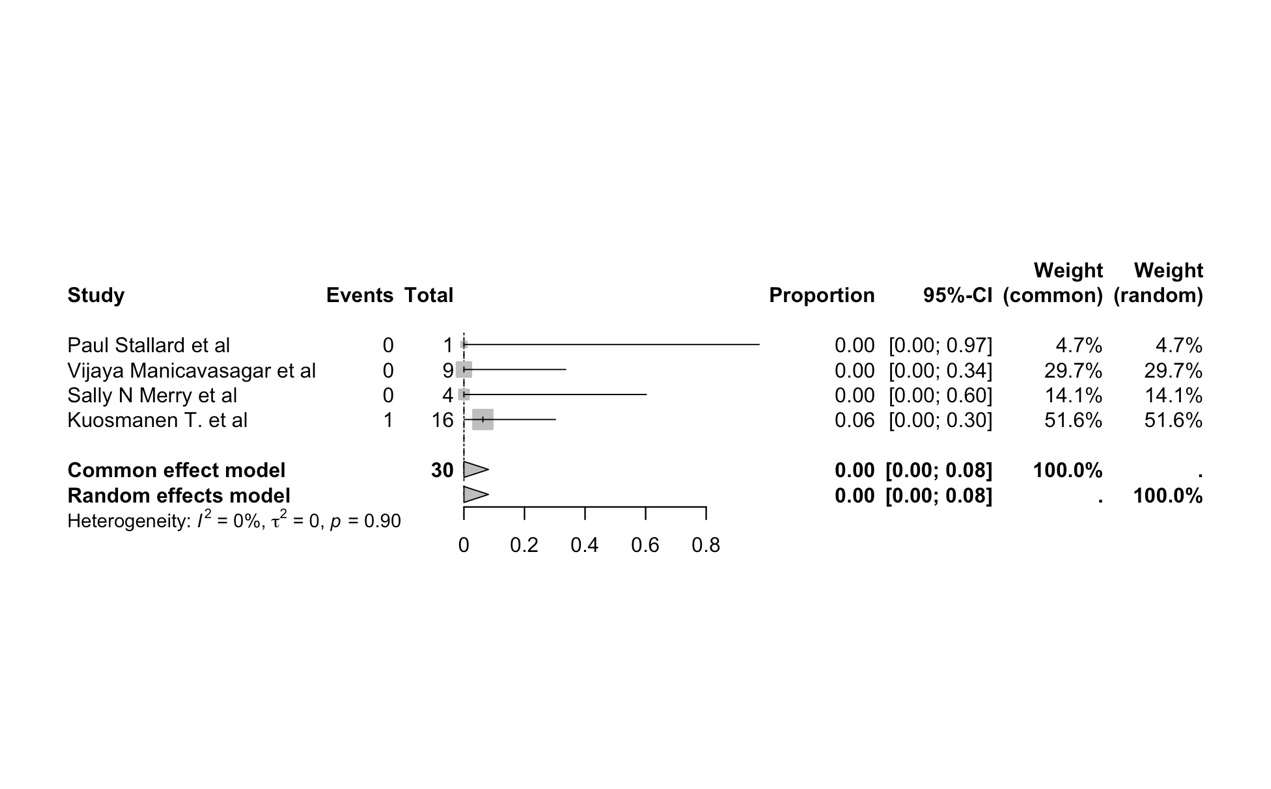


Figure 2g. Proportion of Perceived Risks (B9) in CNPD group


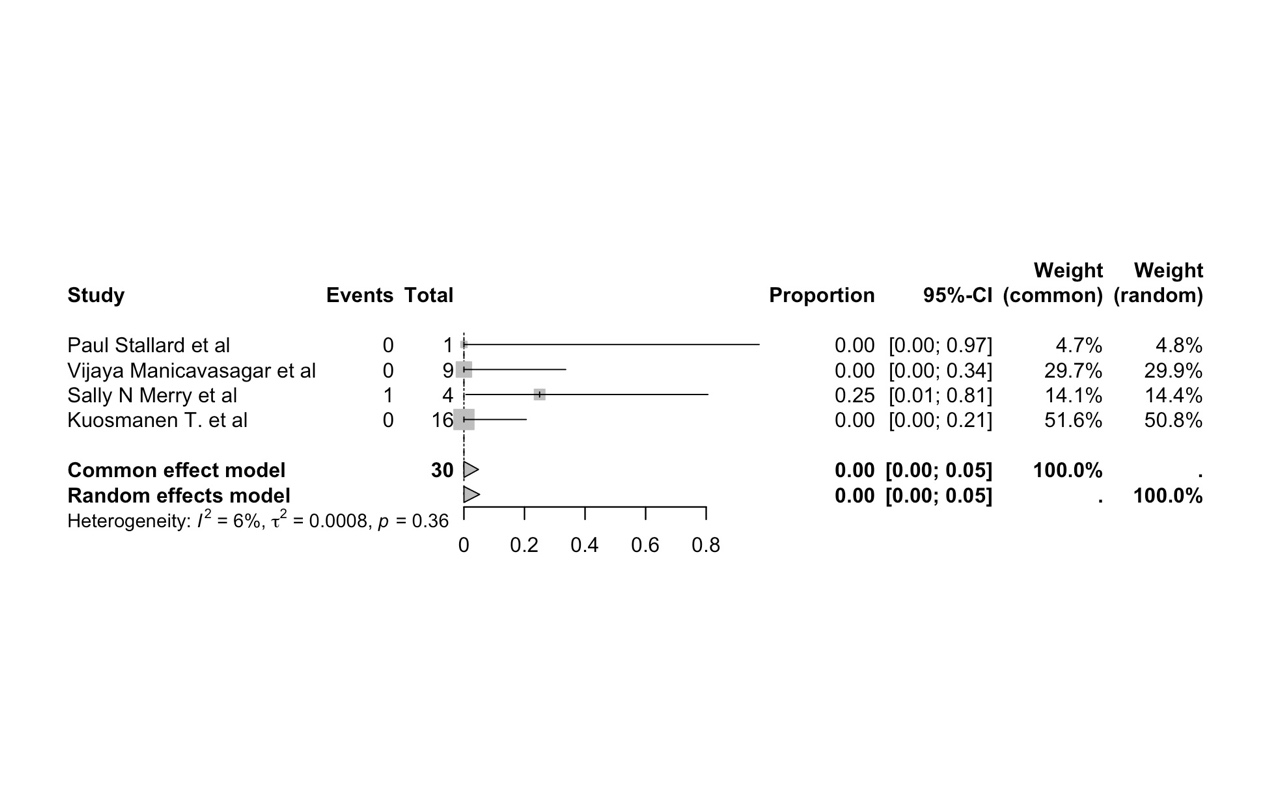


Figure 2h. Proportion of Question (B10) in CNPD group


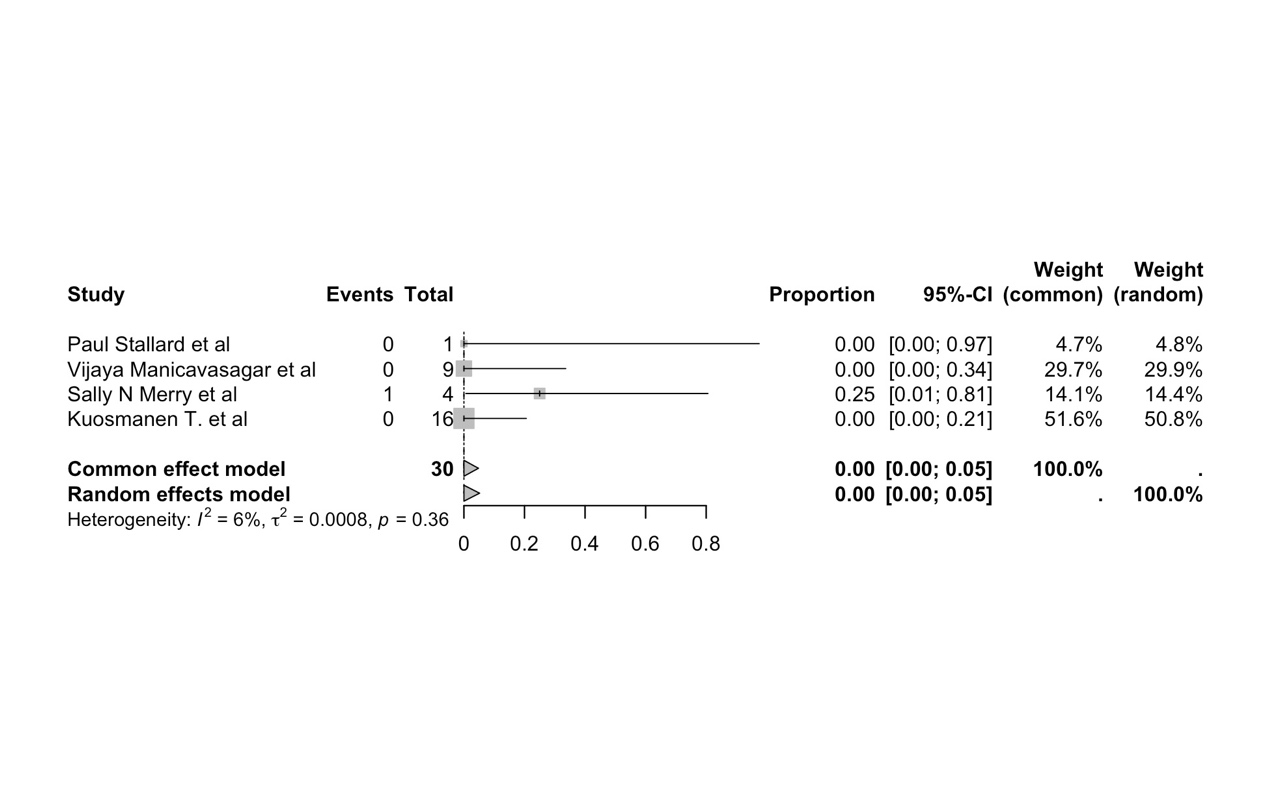


Figure 2i. Proportion of Retention Issues (B11) in CNPD group


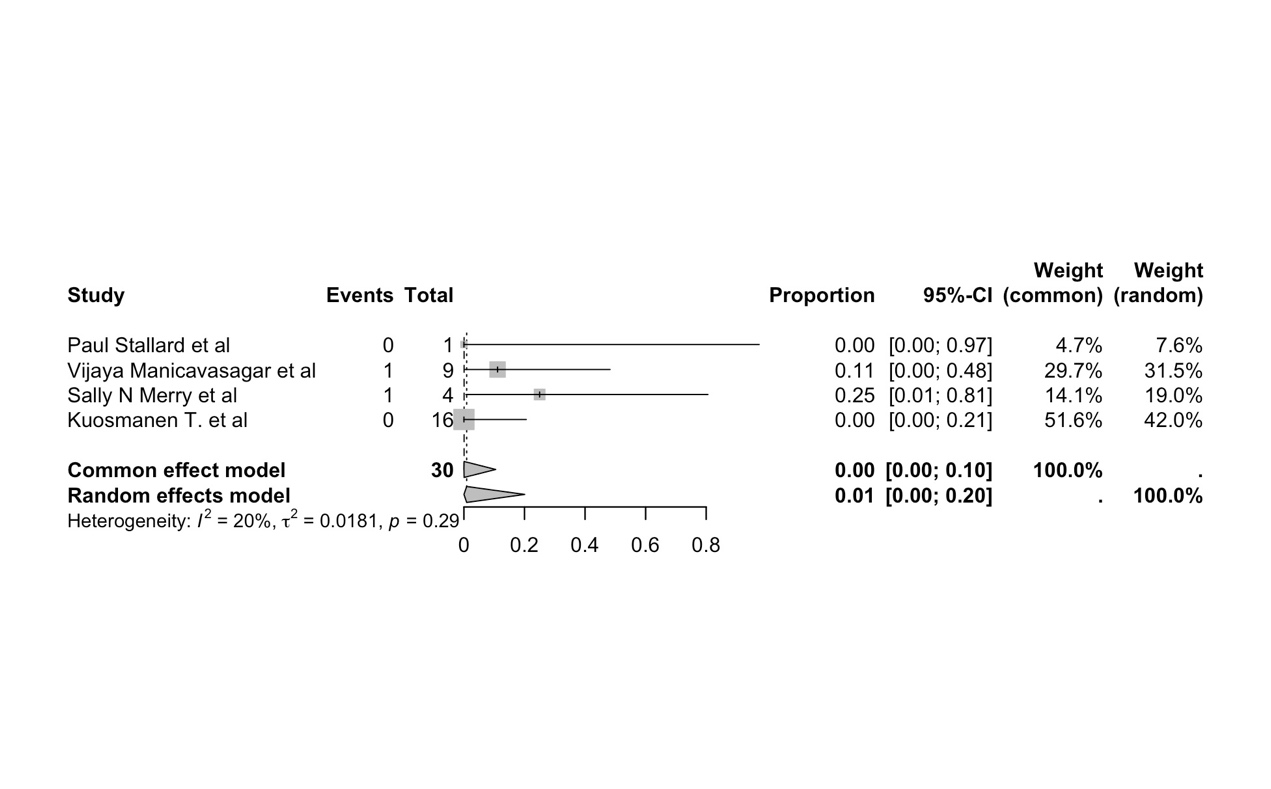


Figure 2j. Proportion of No/Limited Time (B12) in CNPD group


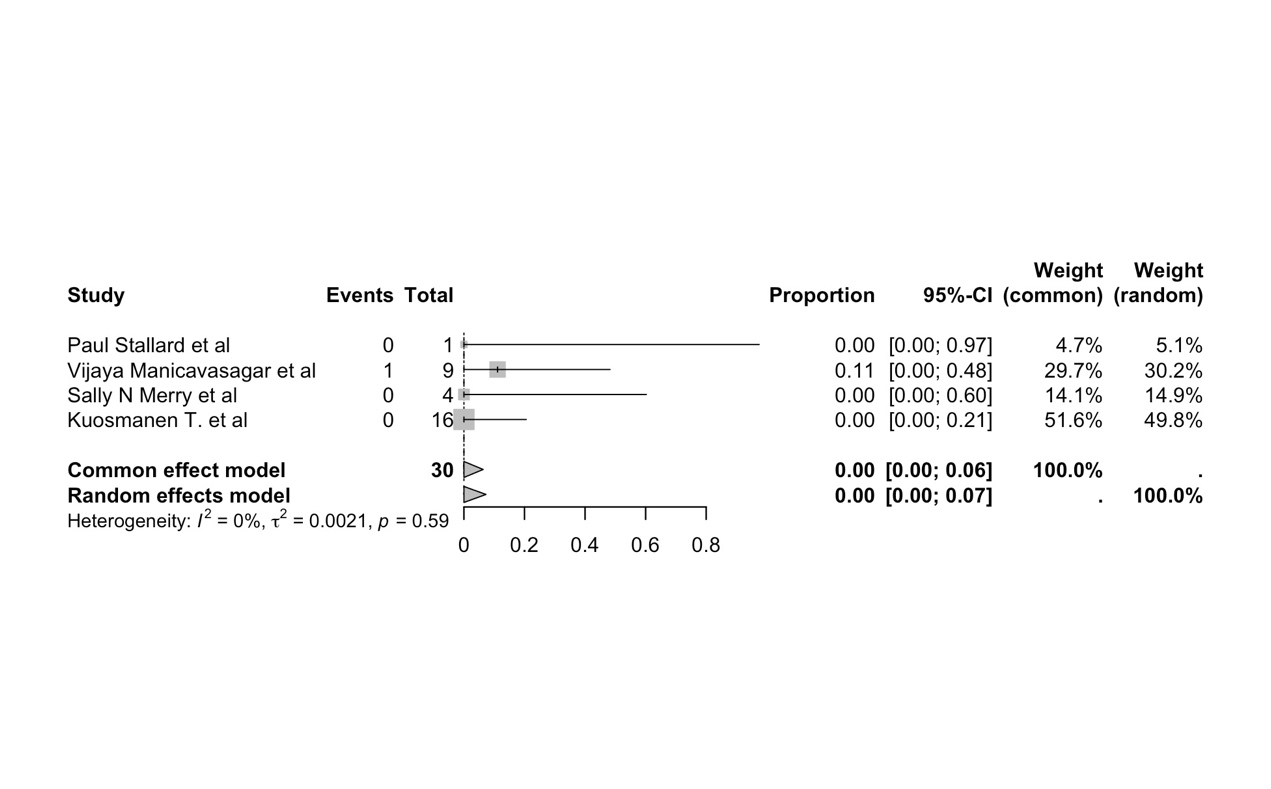


Figure 2k. Proportion of Technical Issues (B13) in CNPD group

Portable Devices (PD)

Facilitators


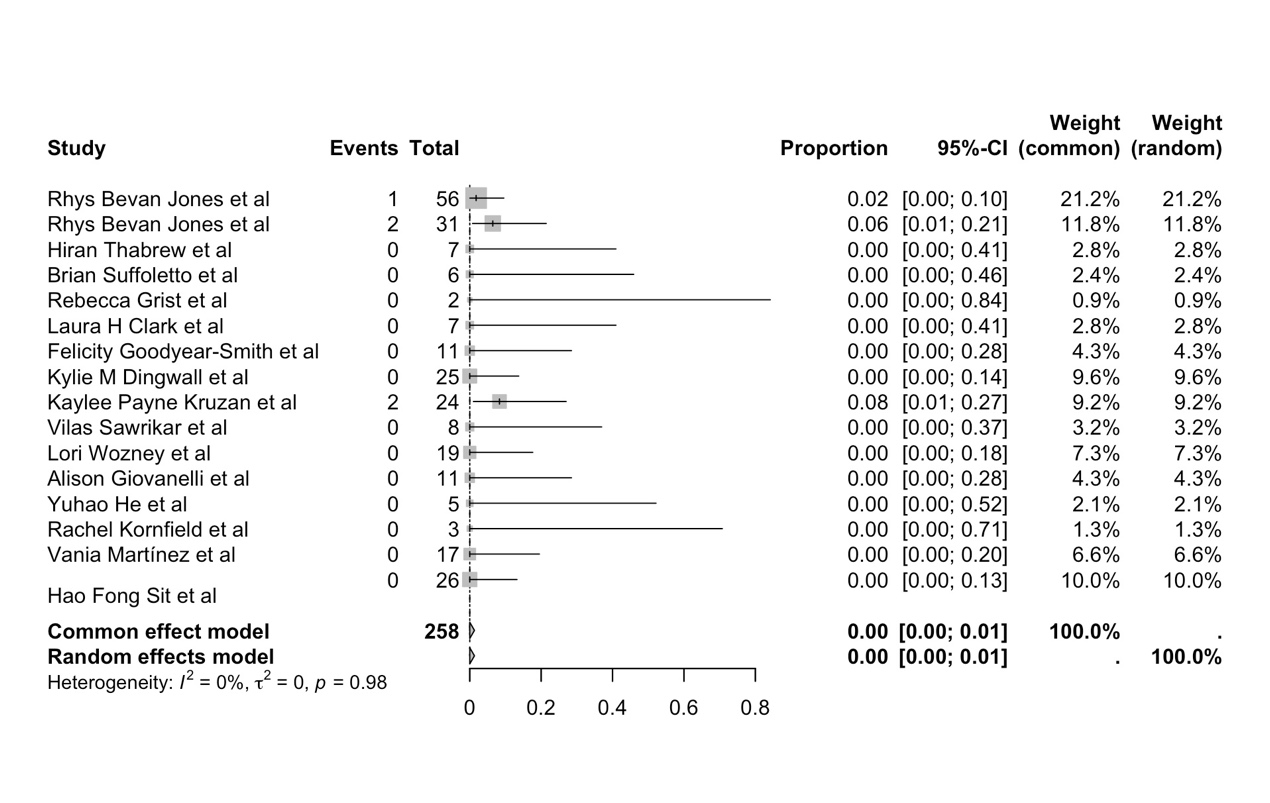


Figure 3a. Proportion of Integration with Schools and Other Resources (F1) in PD group


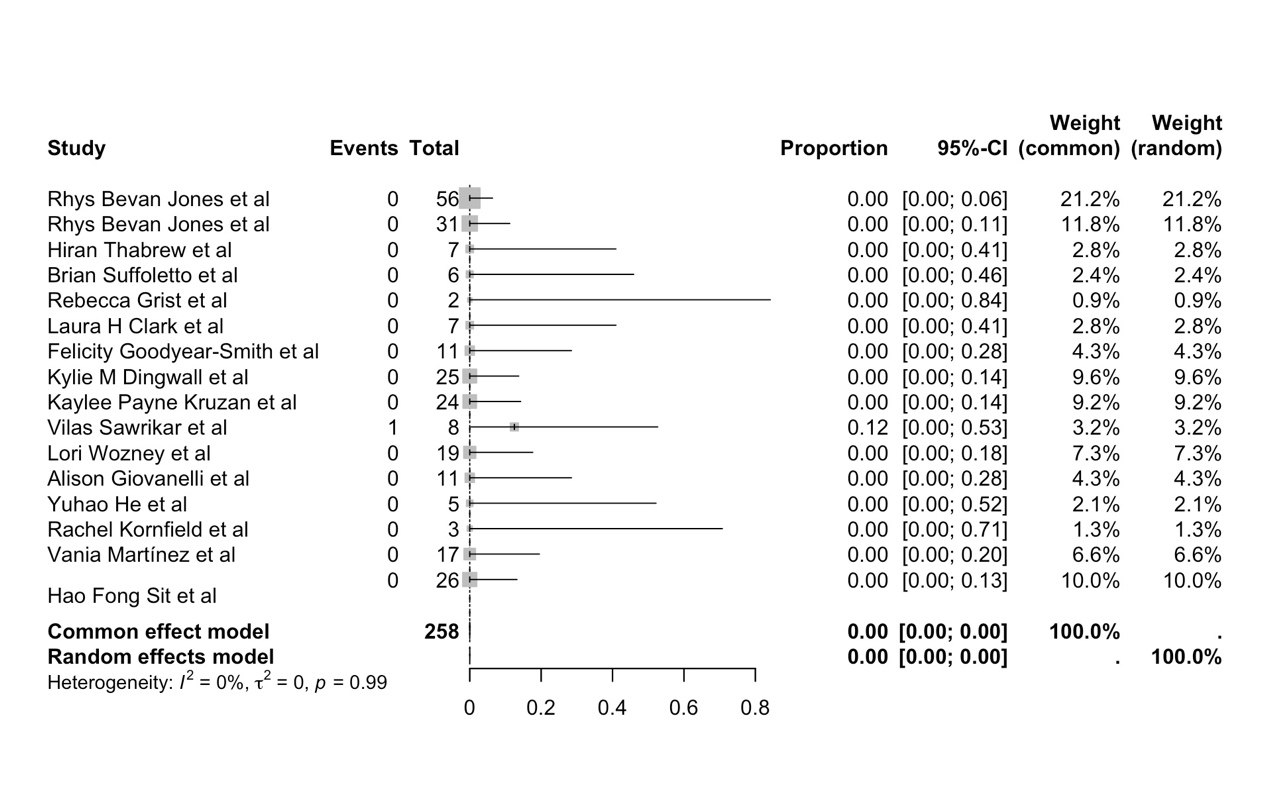


Figure 3b. Proportion of Social Norms (F2) in PD group


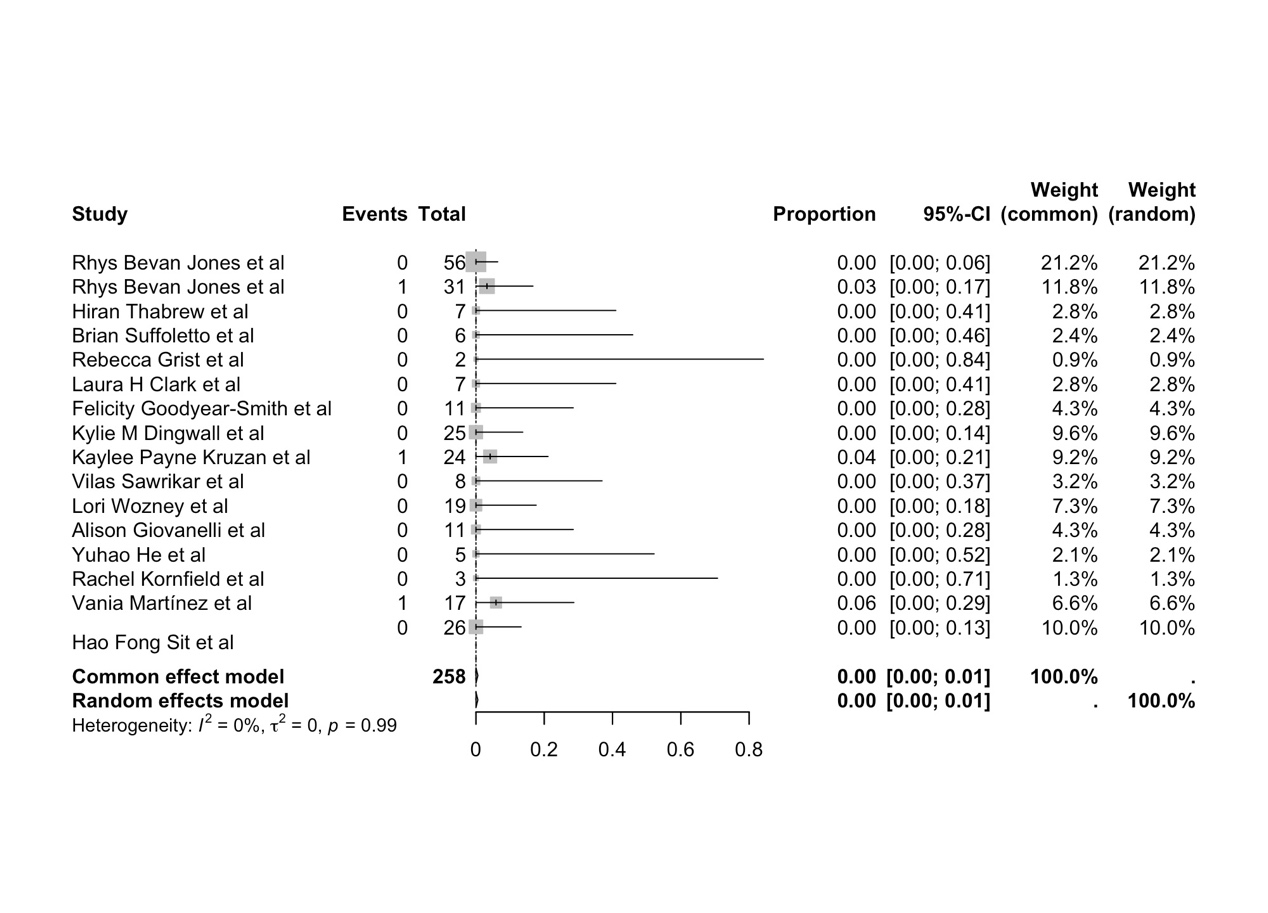


Figure 3c. Proportion of Strategic Marketing (F3) in PD group


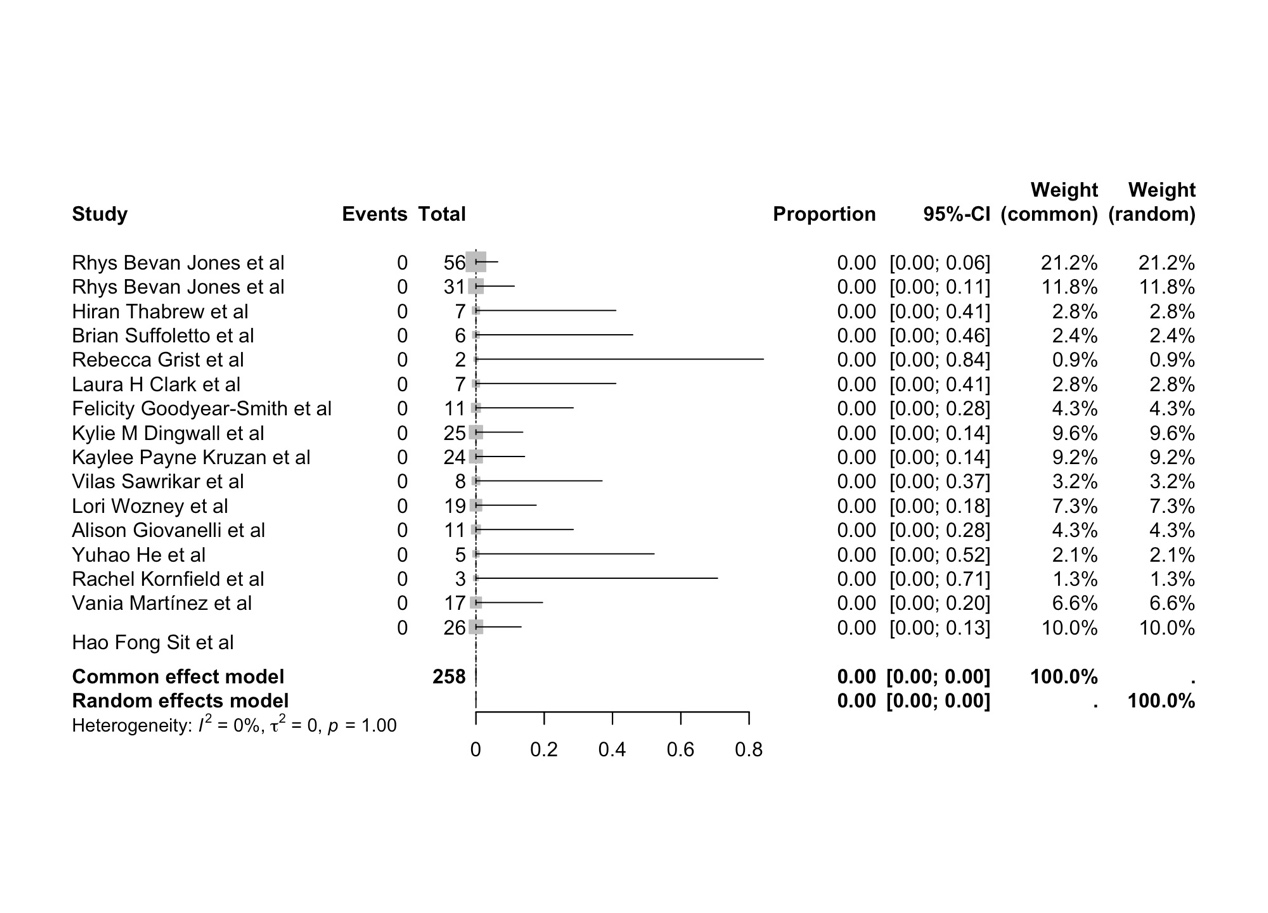


Figure 3d. Proportion of Universality (F4) in PD group


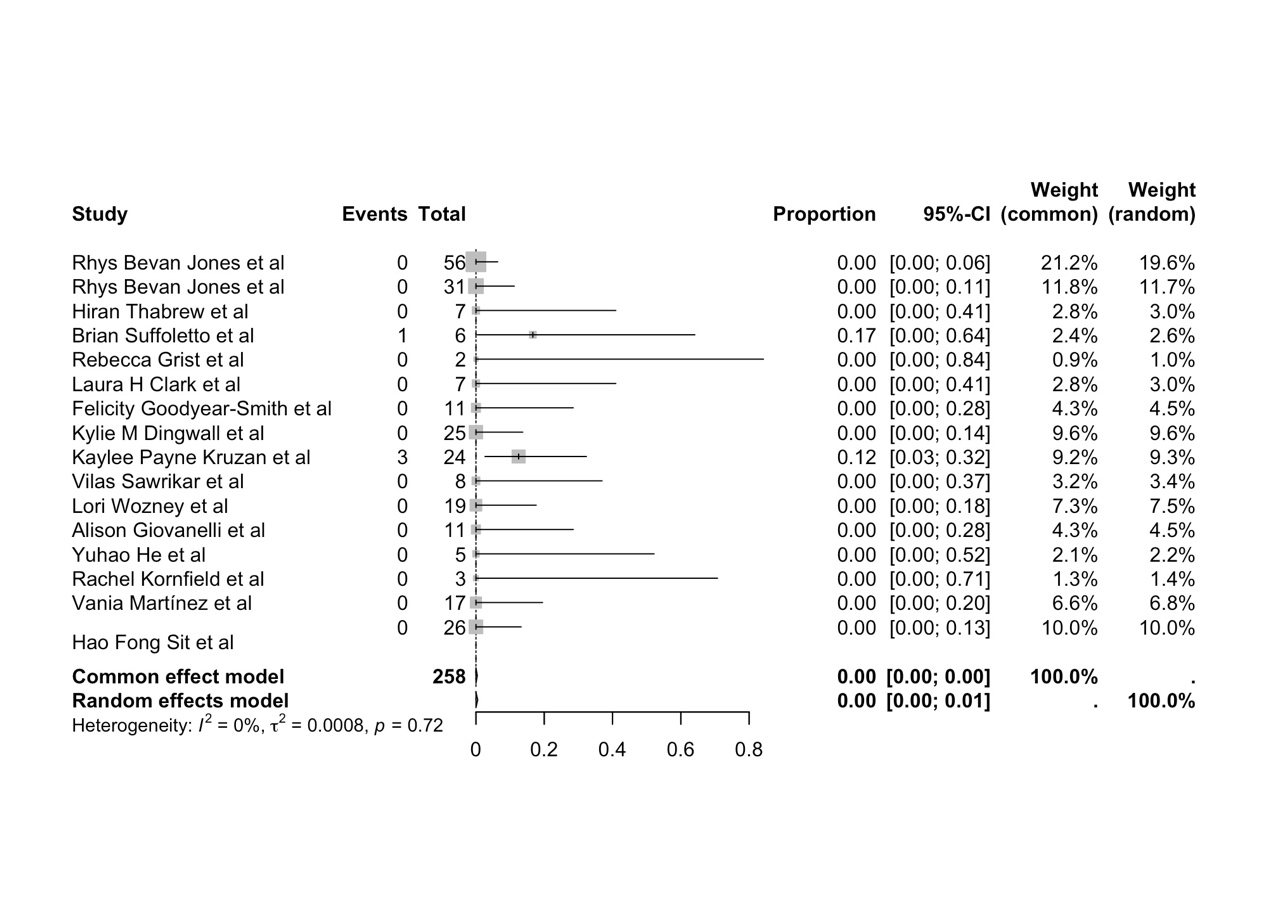


Figure 3e. Proportion of Endorsements (F5) in PD group


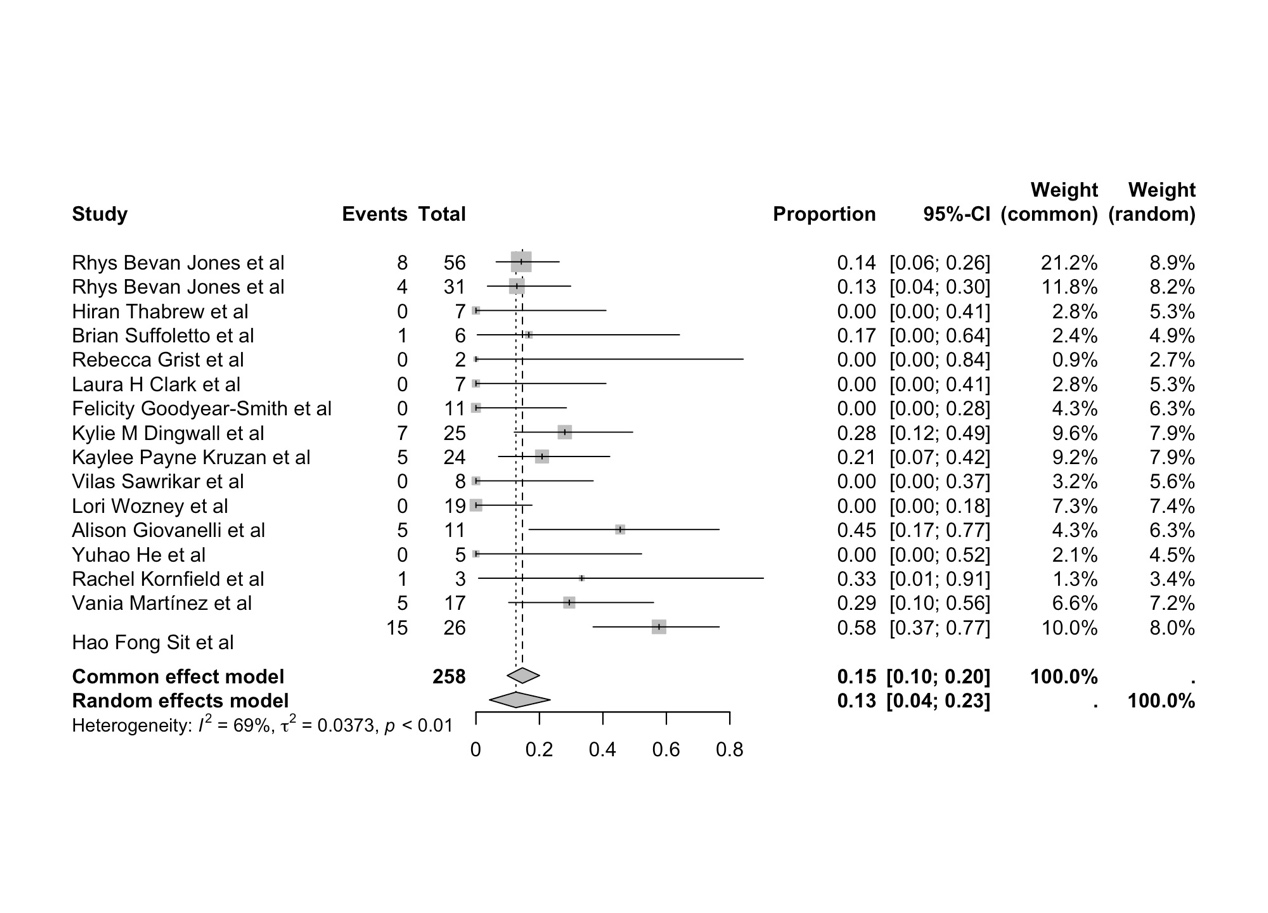


Figure 3f. Proportion of Content Engagement (F6) in PD group


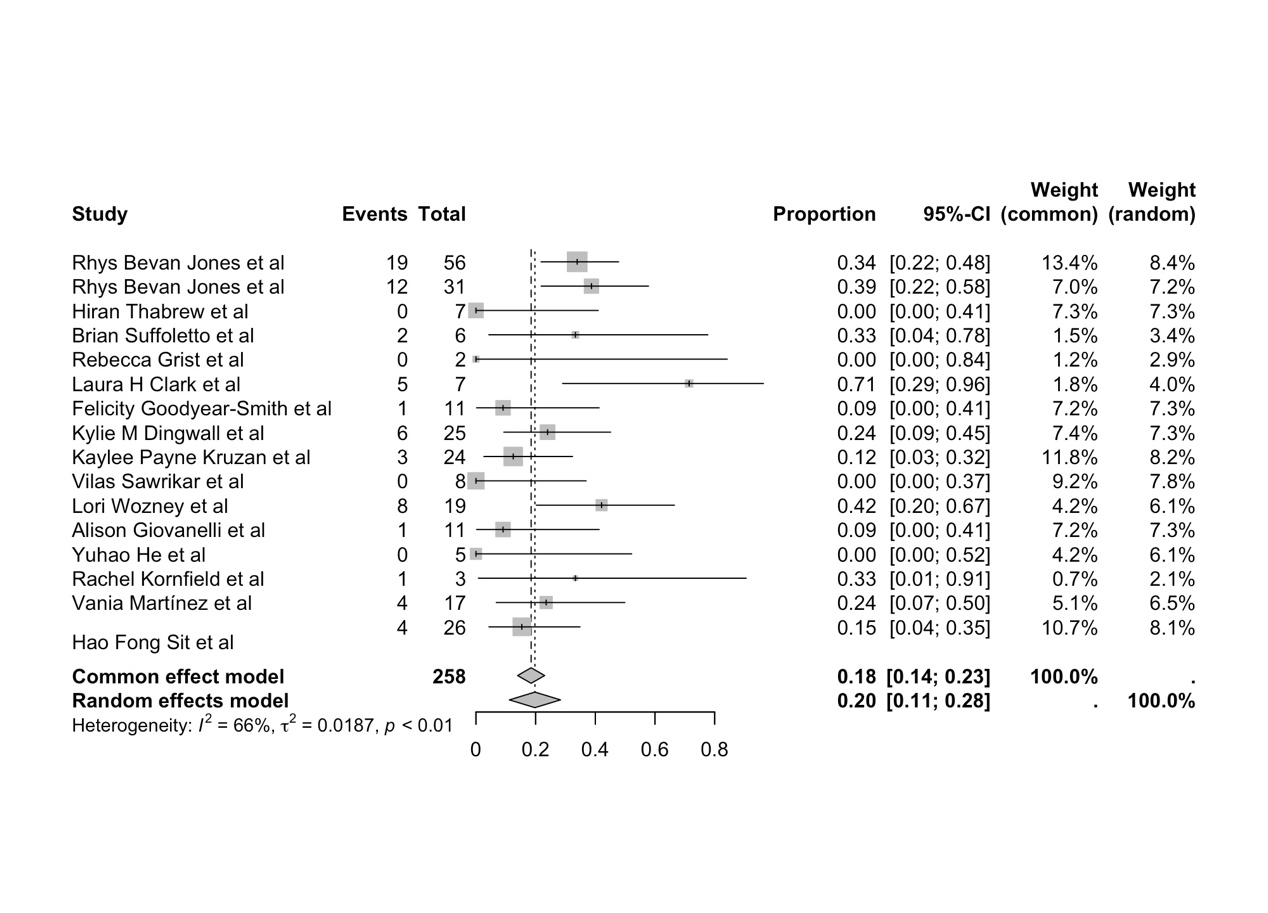


Figure 3g. Proportion of Design Harmony (F7) in PD group


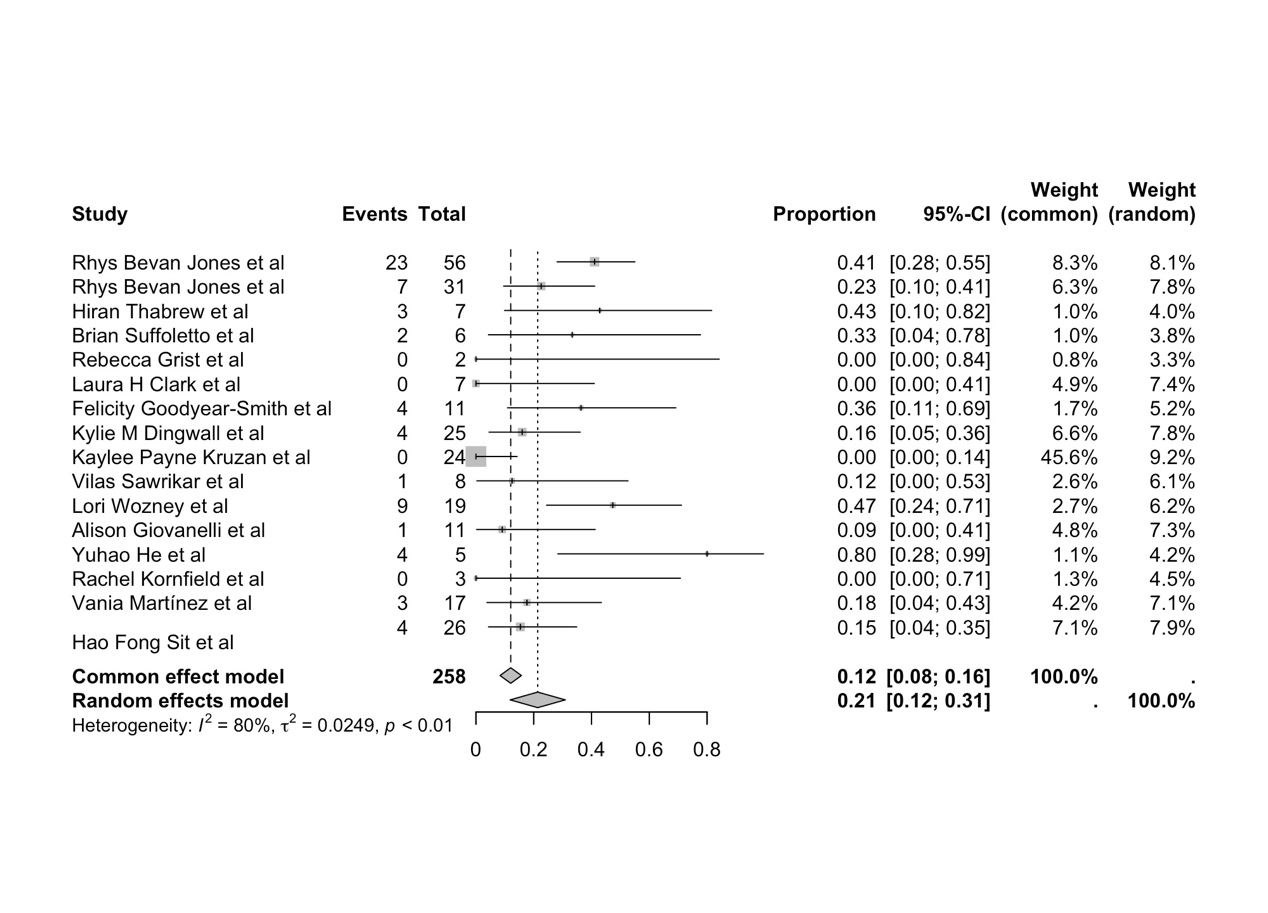


Figure 3h. Proportion of High Quality and Effect (F8) in PD group


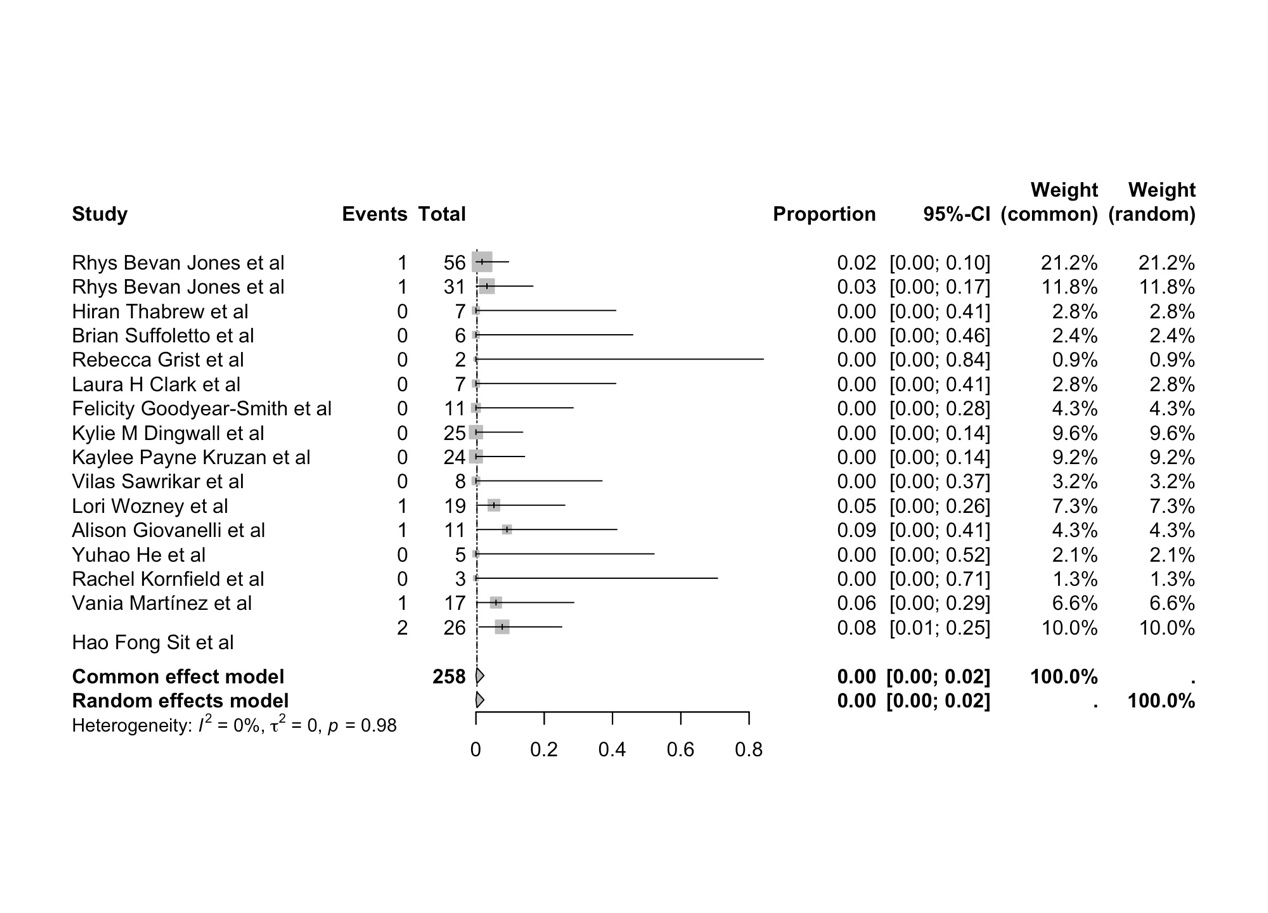


Figure 3i. Proportion of Appropriate Duration and Schedule (F9) in PD group


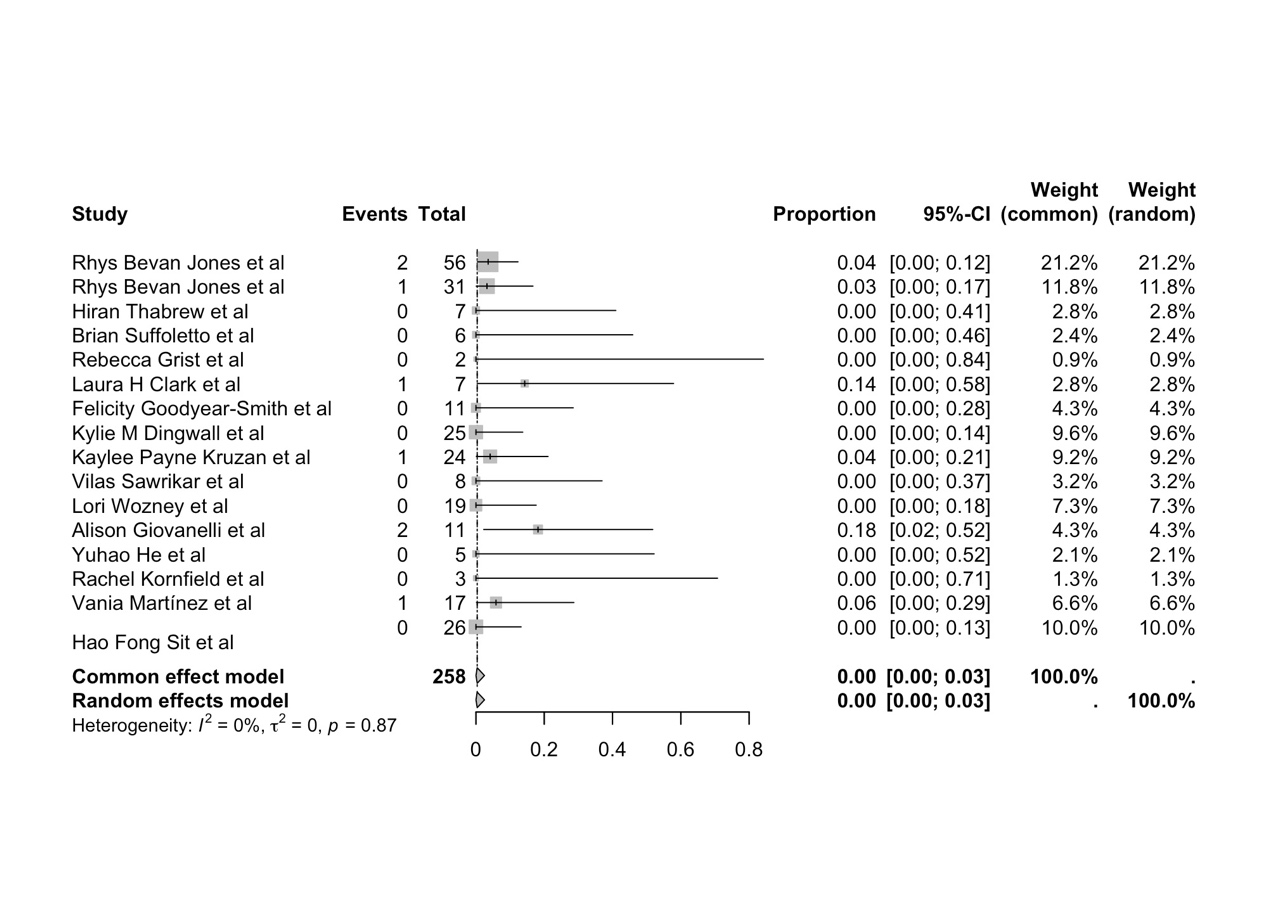


Figure 3j. Proportion of Accessibility (F10) in PD group


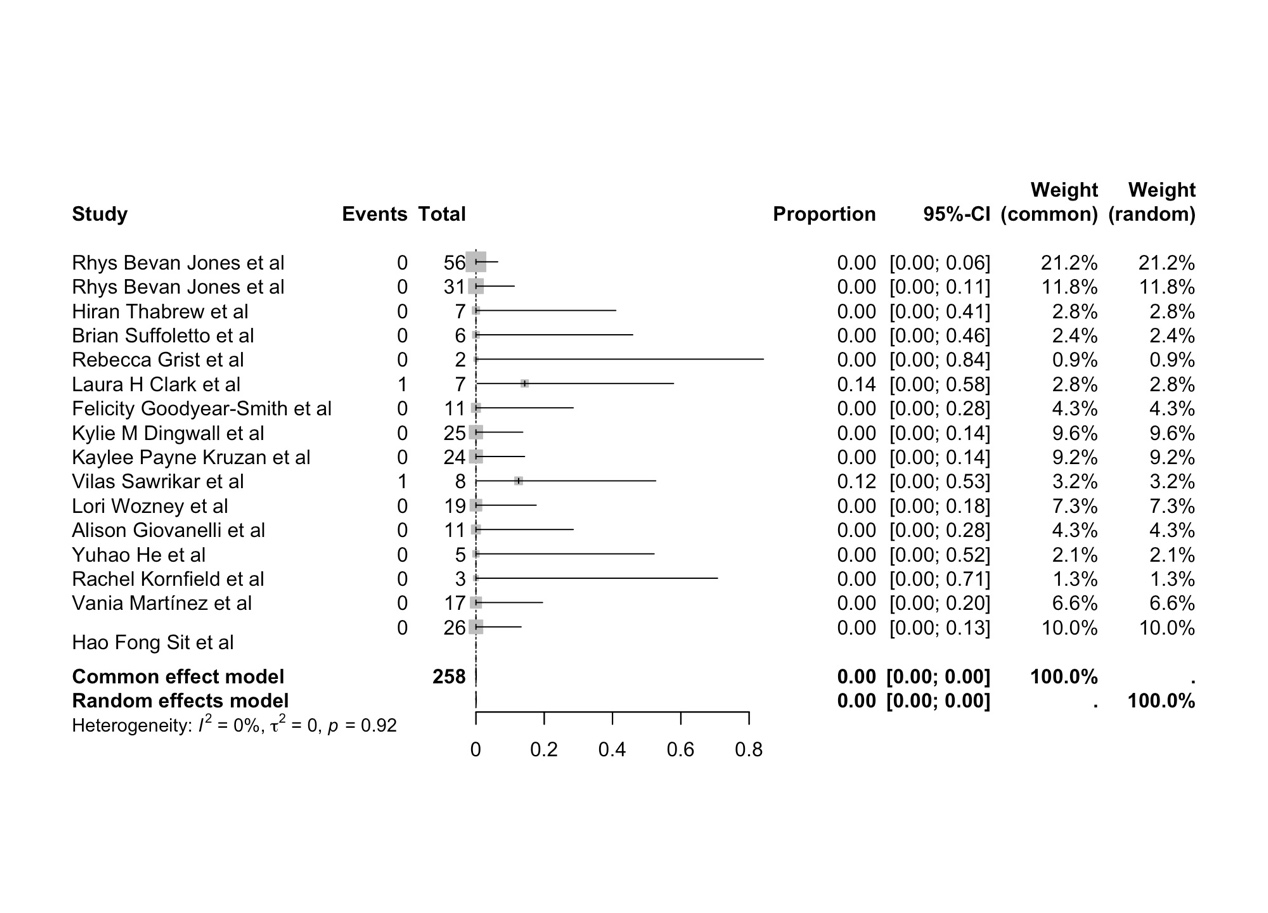


Figure 3k. Proportion of Beneficial Characteristics (F11) in PD group


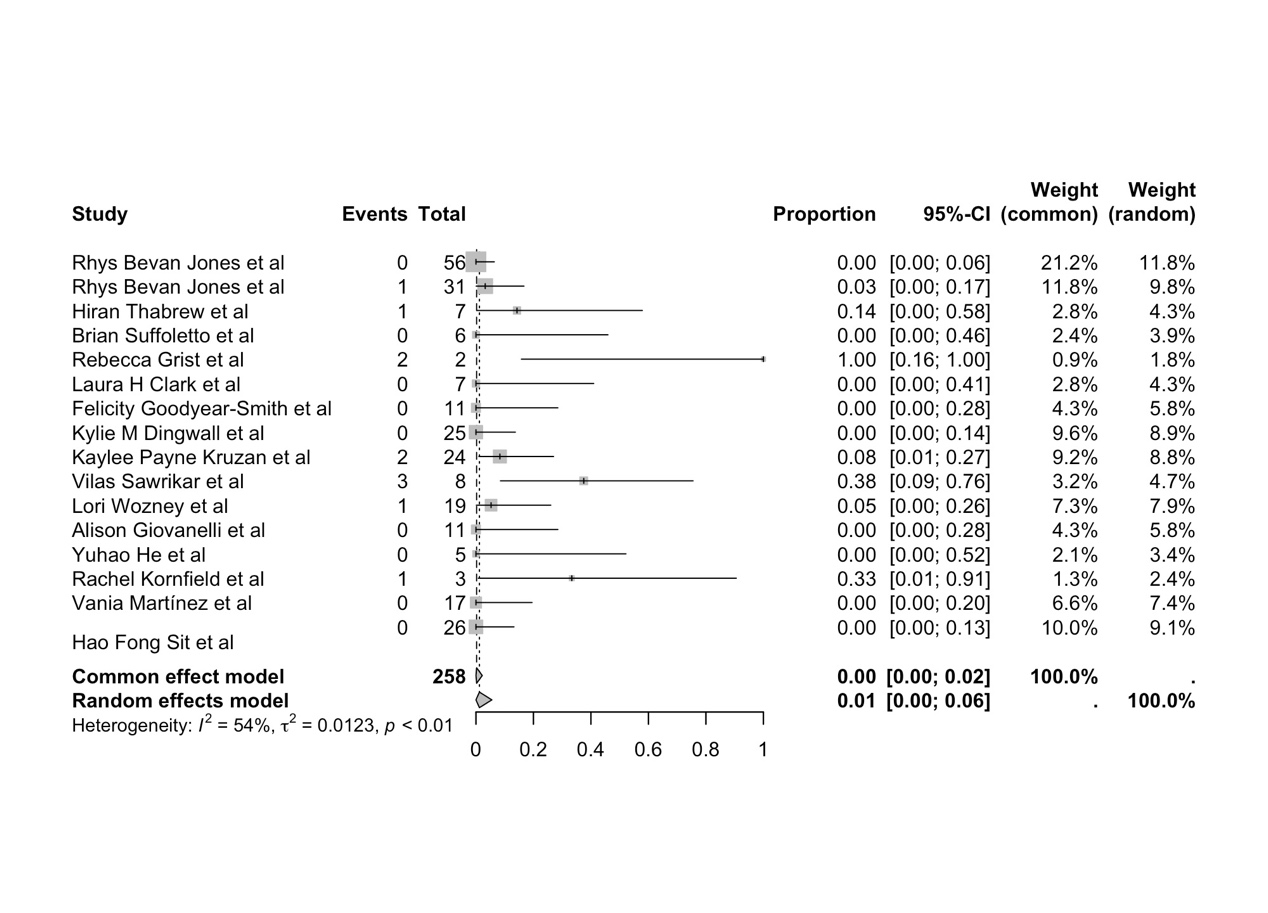


Figure 3l. Proportion of Needs and Disposition (F12) in PD group


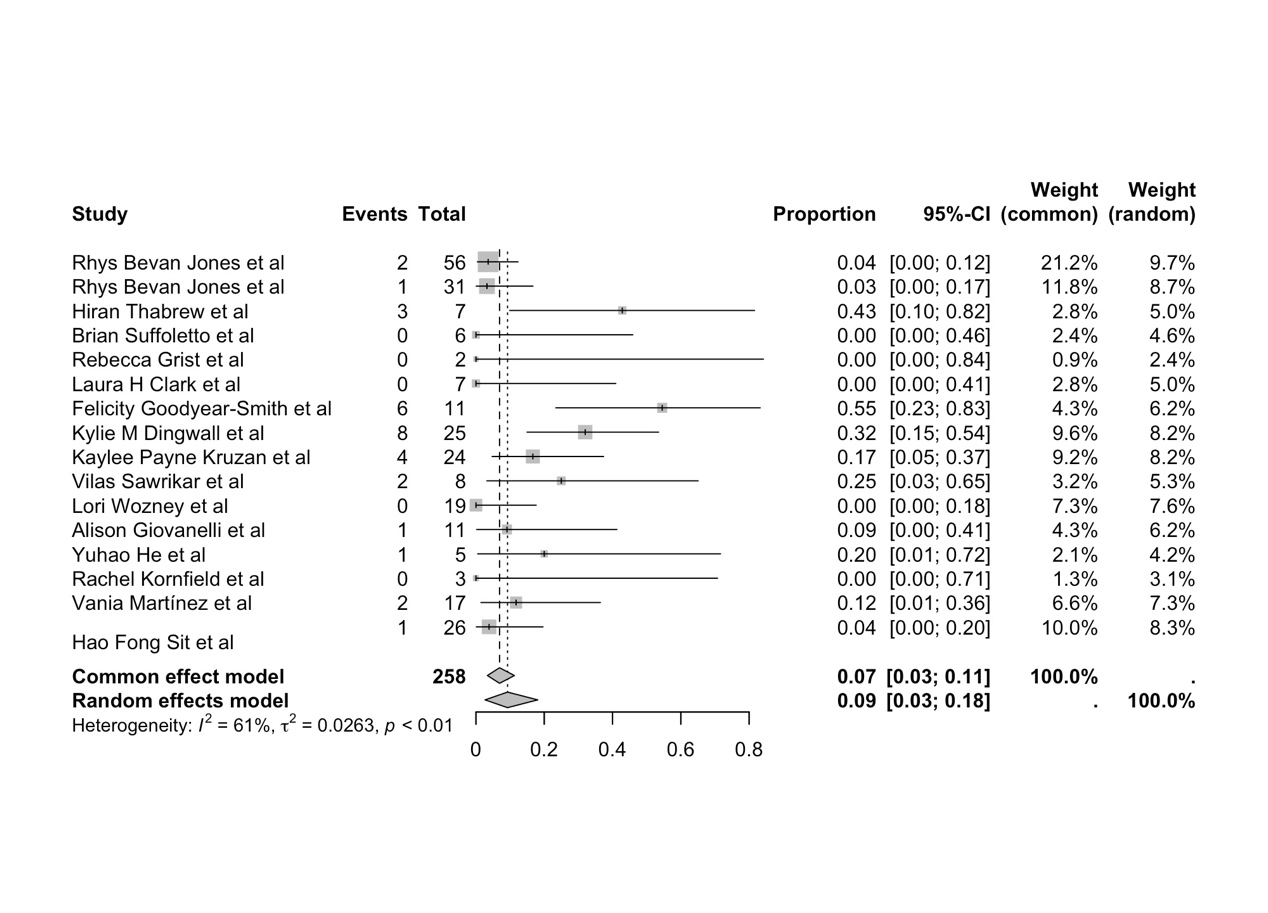


Figure 3m. Proportion of Perceived Benefits (F13) in PD group


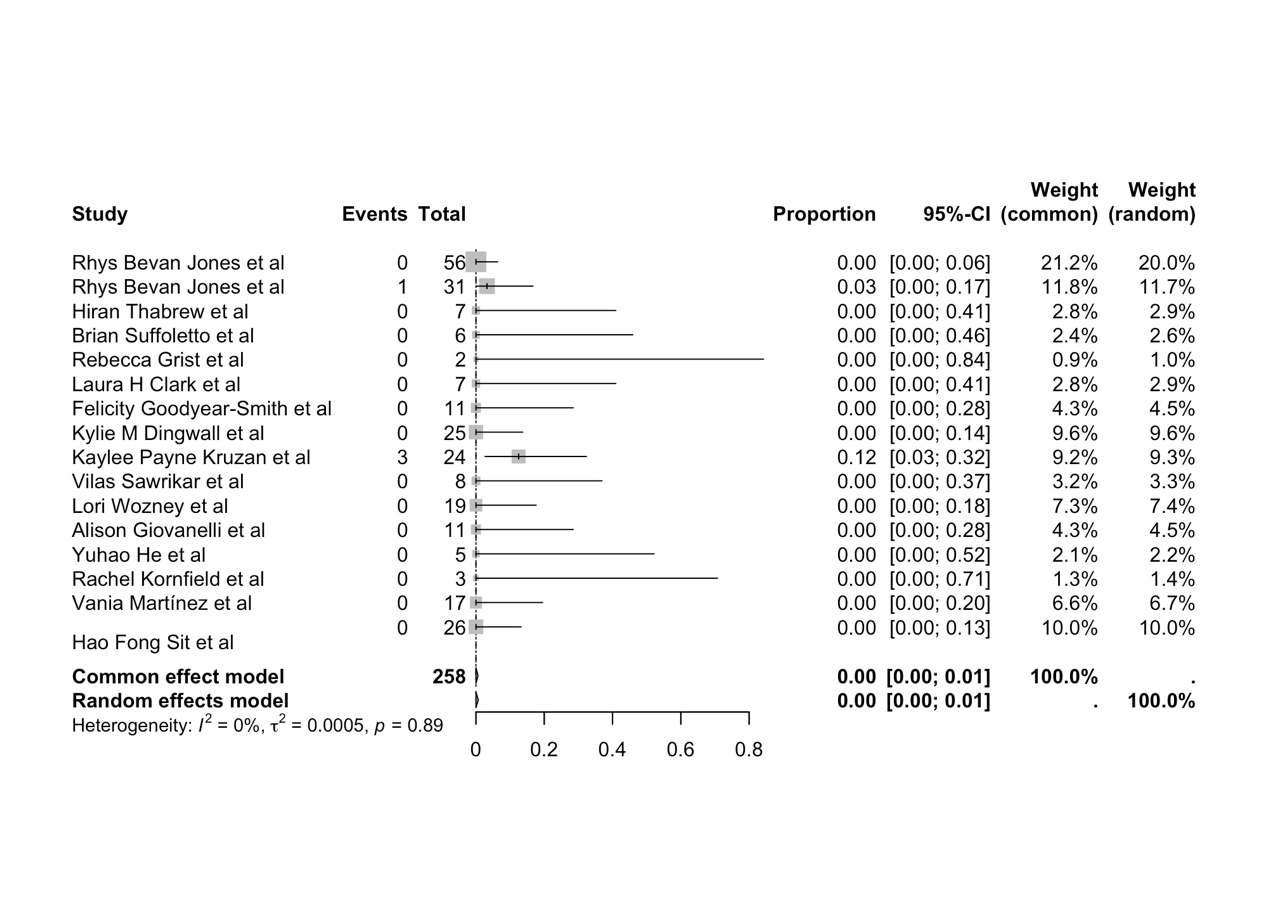


Figure 3n. Proportion of Supportive Environment (F14) in PD group

Portable Devices (PD)

Barriers


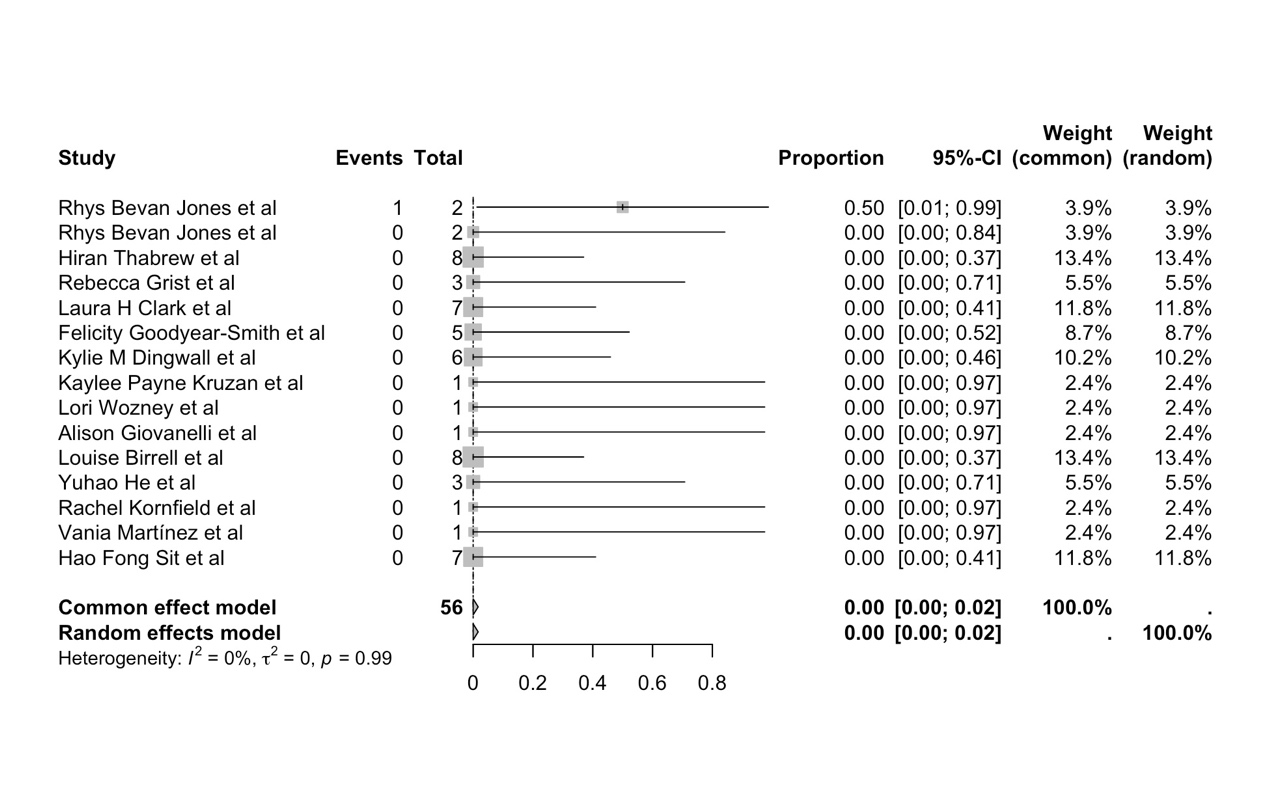


Figure 4a. Proportion of Integration with Schools (B1) in PD group


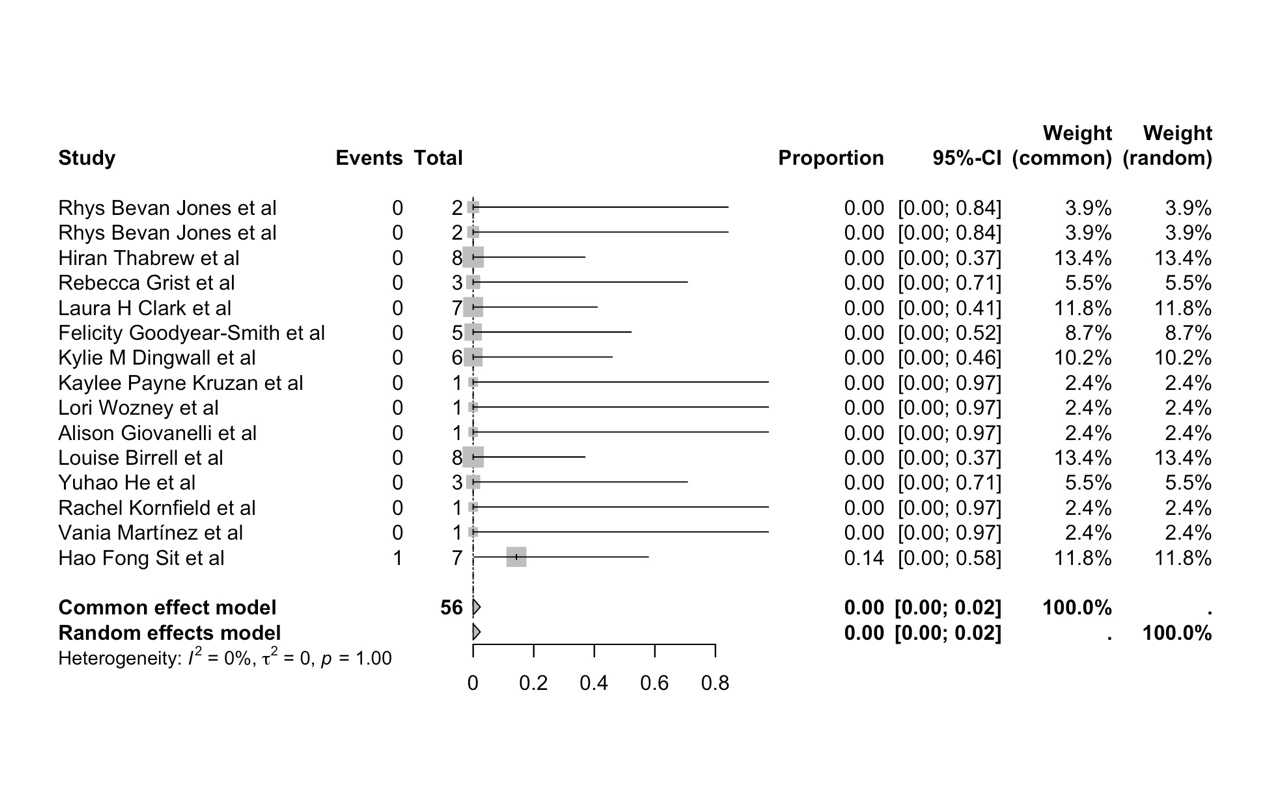


Figure 4b. Proportion of Content Gaps (B2) in PD group


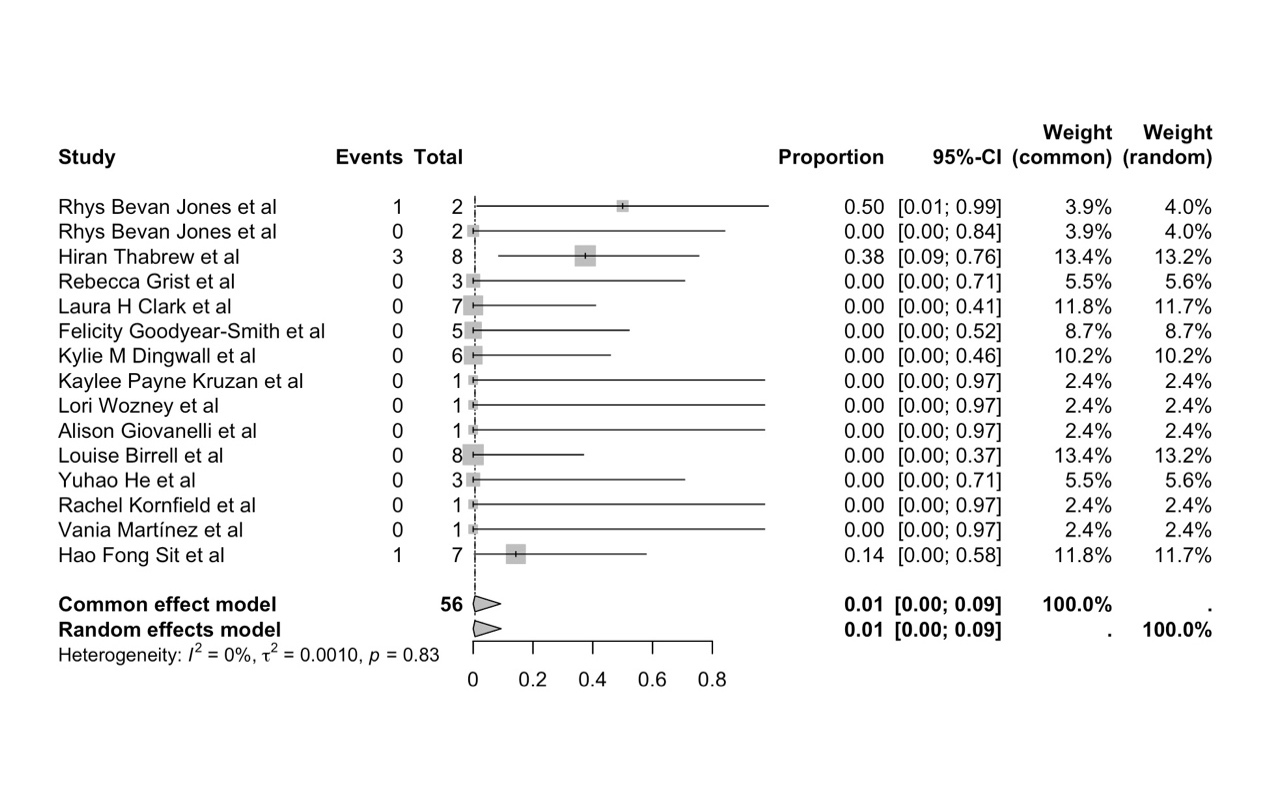


Figure 4c. Proportion of Design Limitations (B3) in PD group


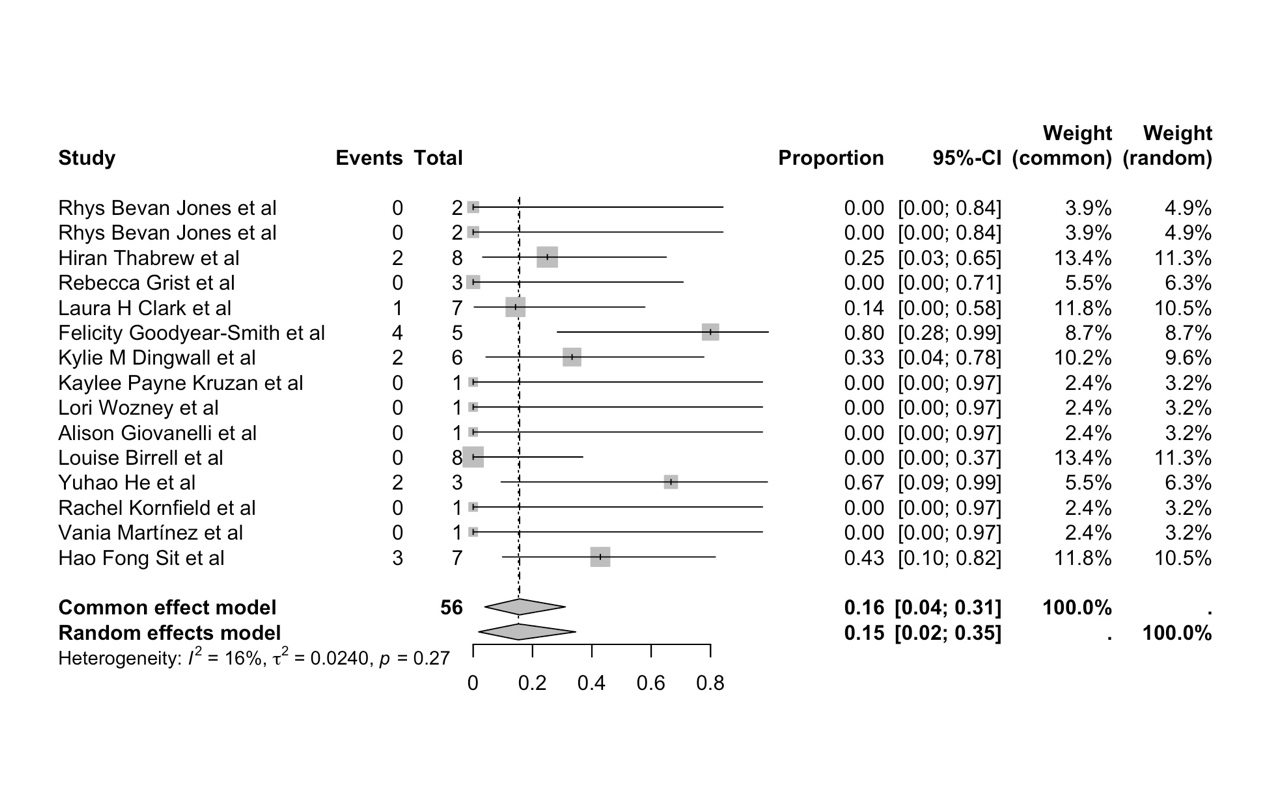


Figure 4d. Proportion of Low Quality and Effect (B4) in PD group


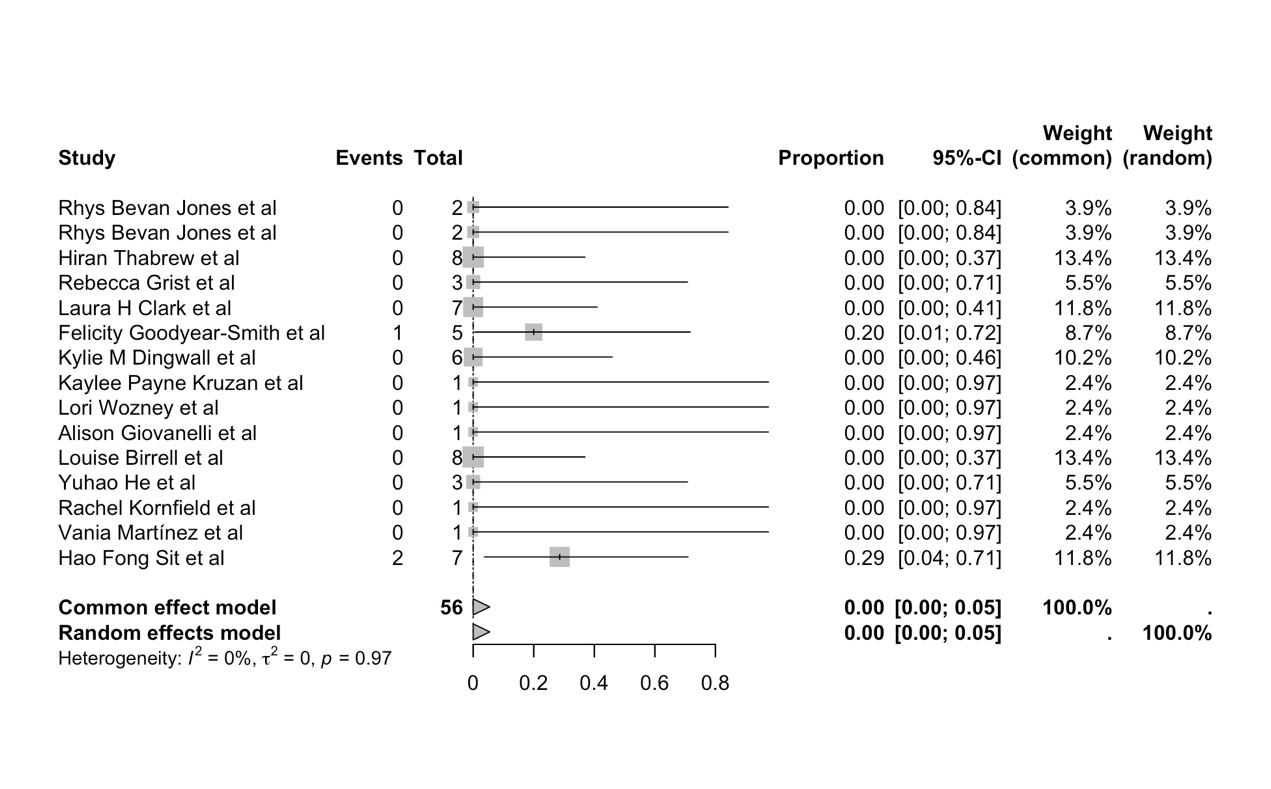


Figure 4e. Proportion of Inappropriate Duration and Schedule (B5) in PD group


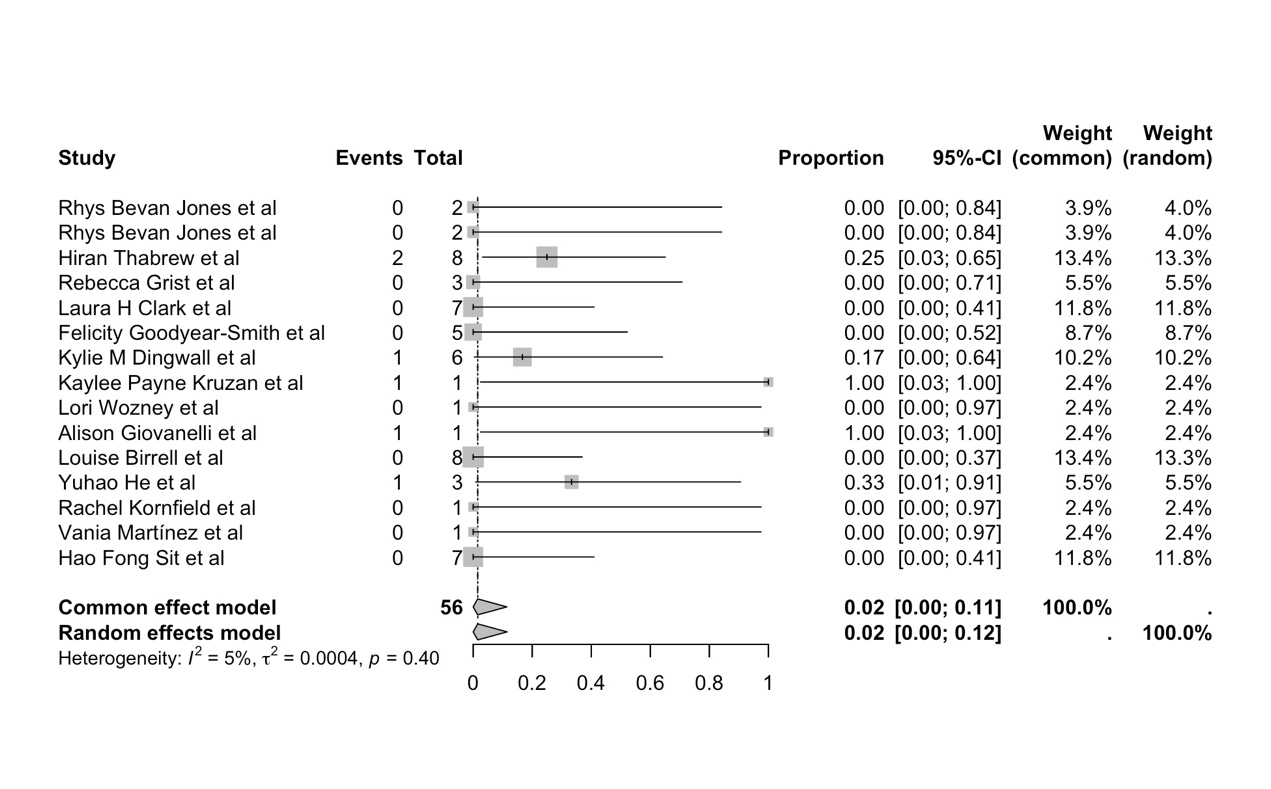


Figure 4f. Proportion of Inaccessibility (B6) in PD group


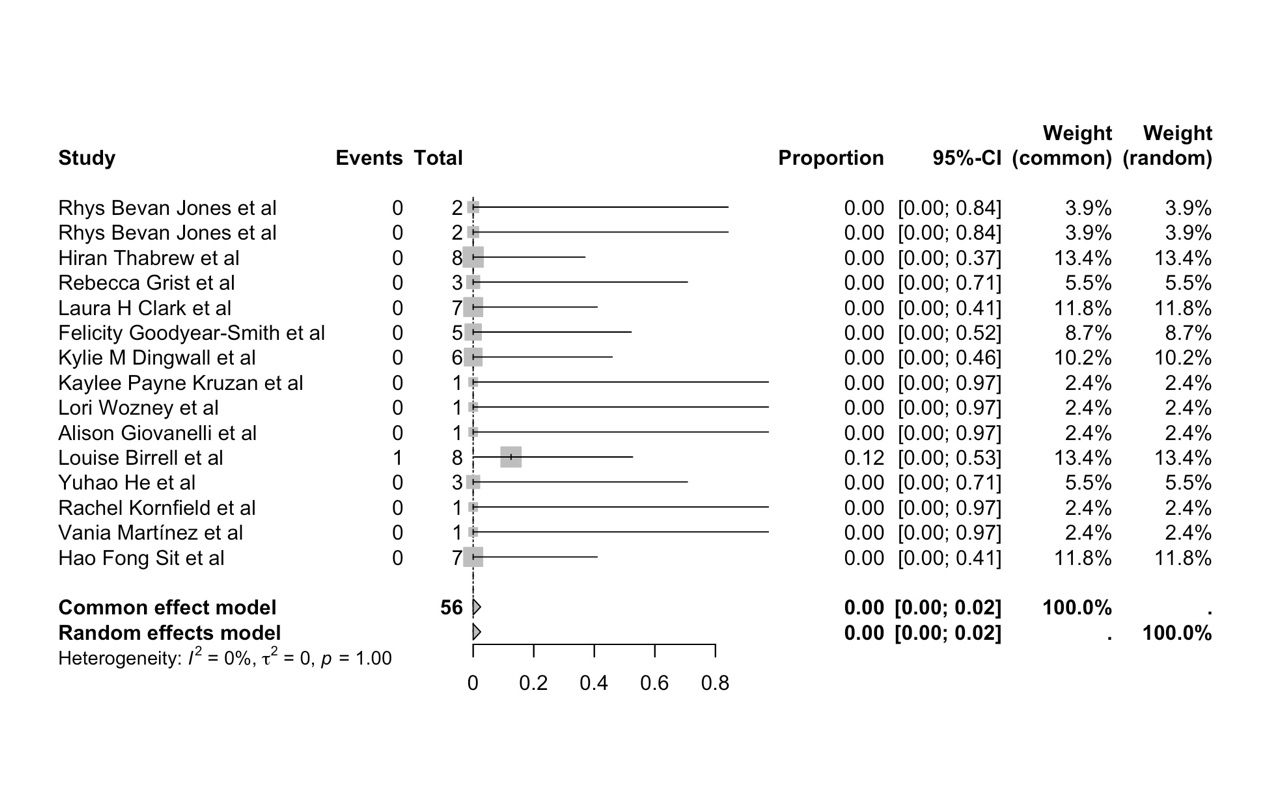


Figure 4g. Proportion of Detrimental Characteristics (B7) in PD group


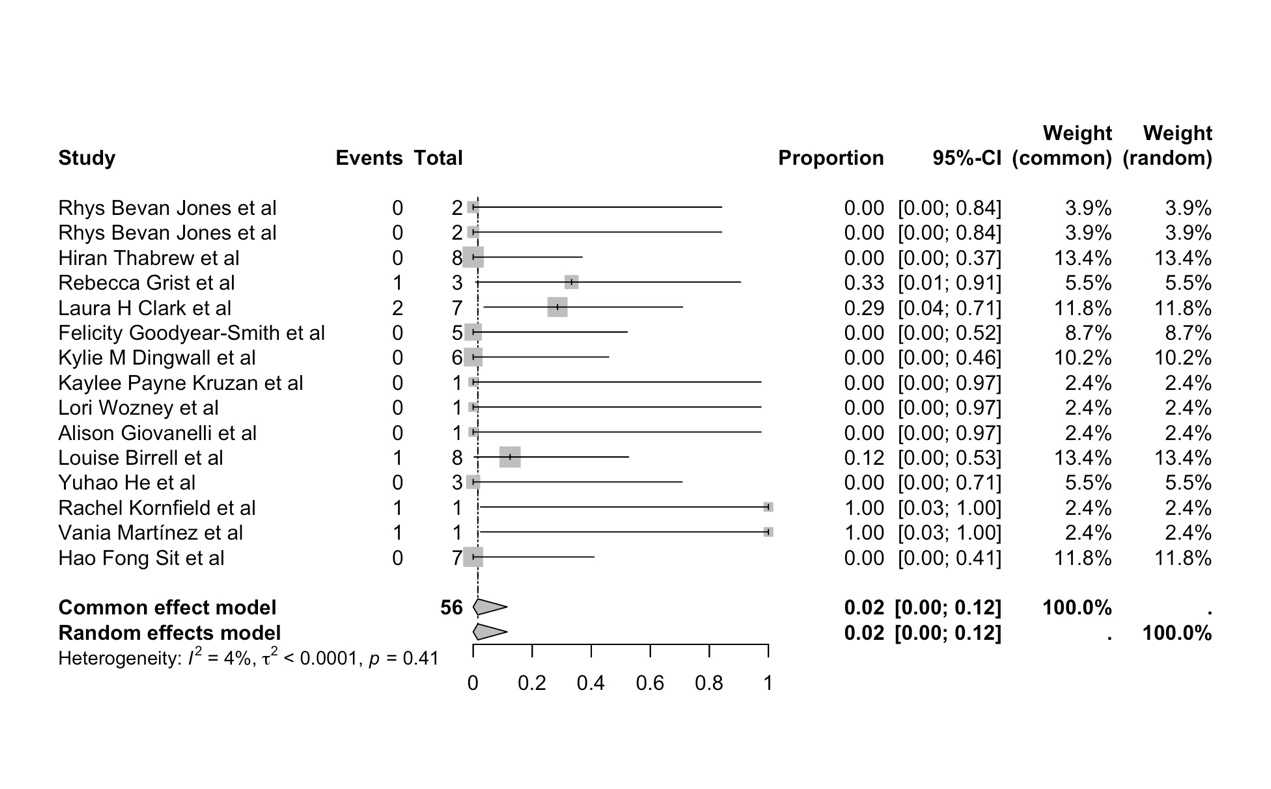


Figure 4h. Proportion of Motivational Challenges (B8) in PD group


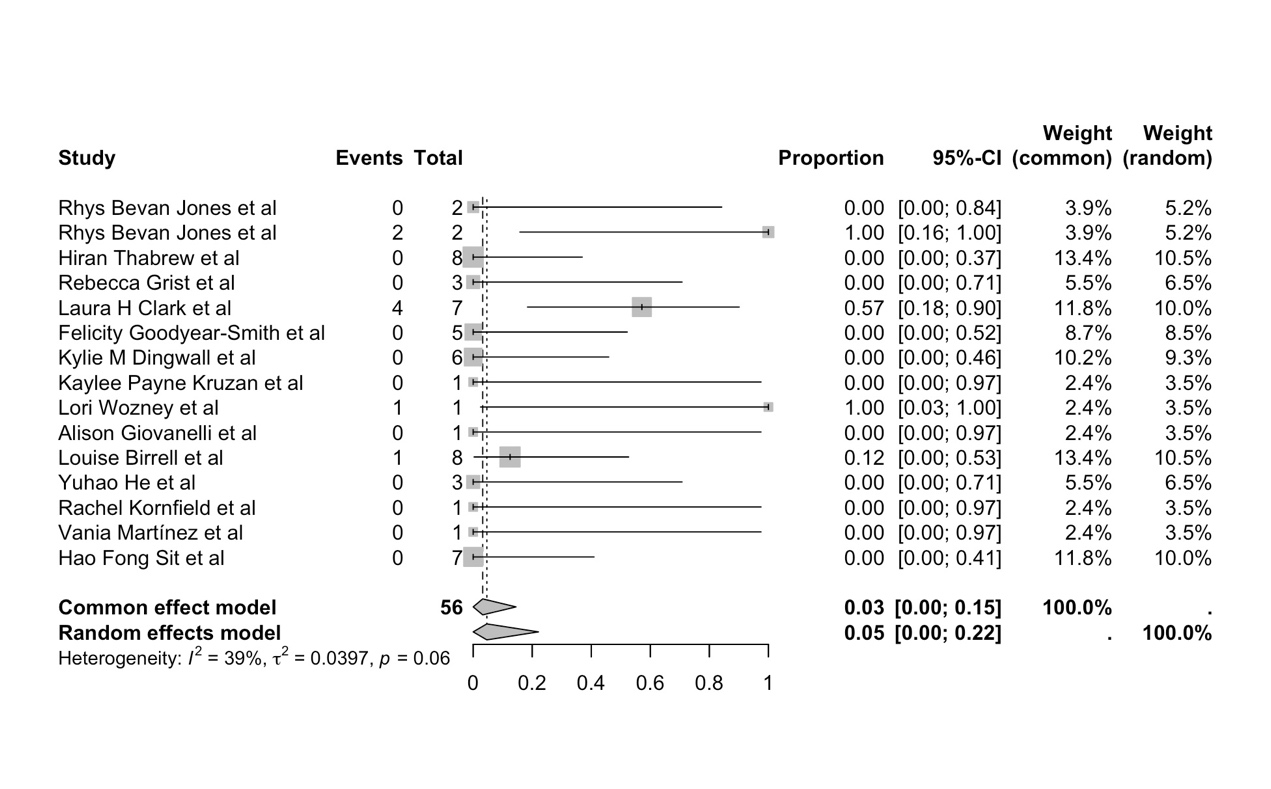


Figure 4i. Proportion of Perceived Risks (B9) in PD group


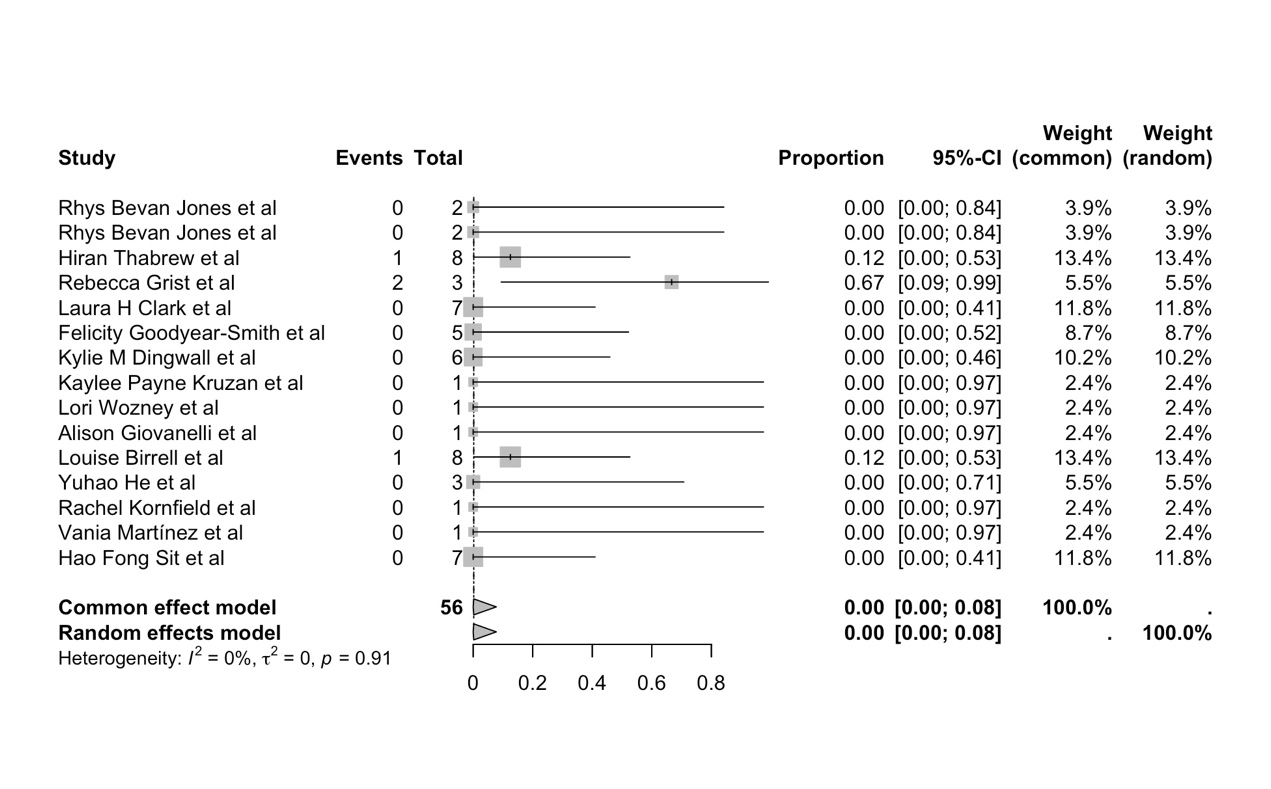


Figure 4j. Proportion of Question (B10) in PD group


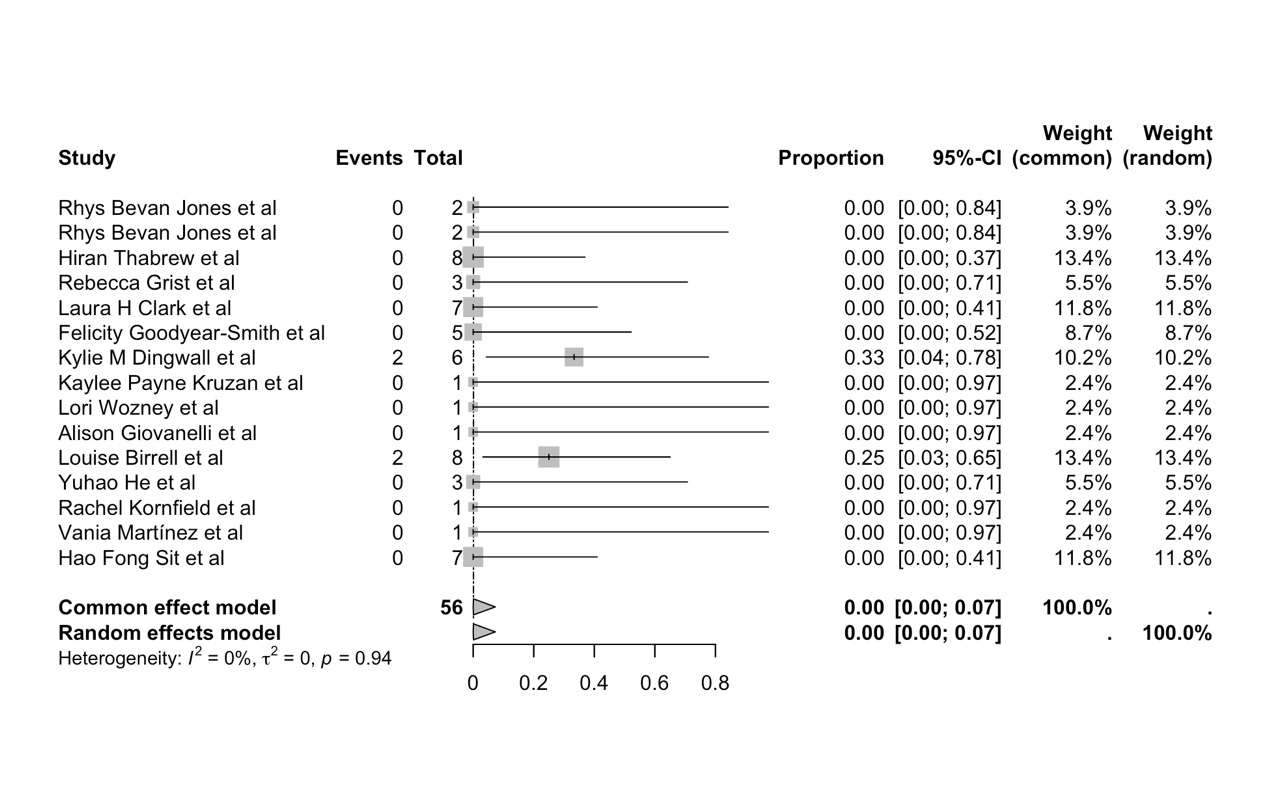


Figure 4k. Proportion of Retention Issues (B11) in PD group


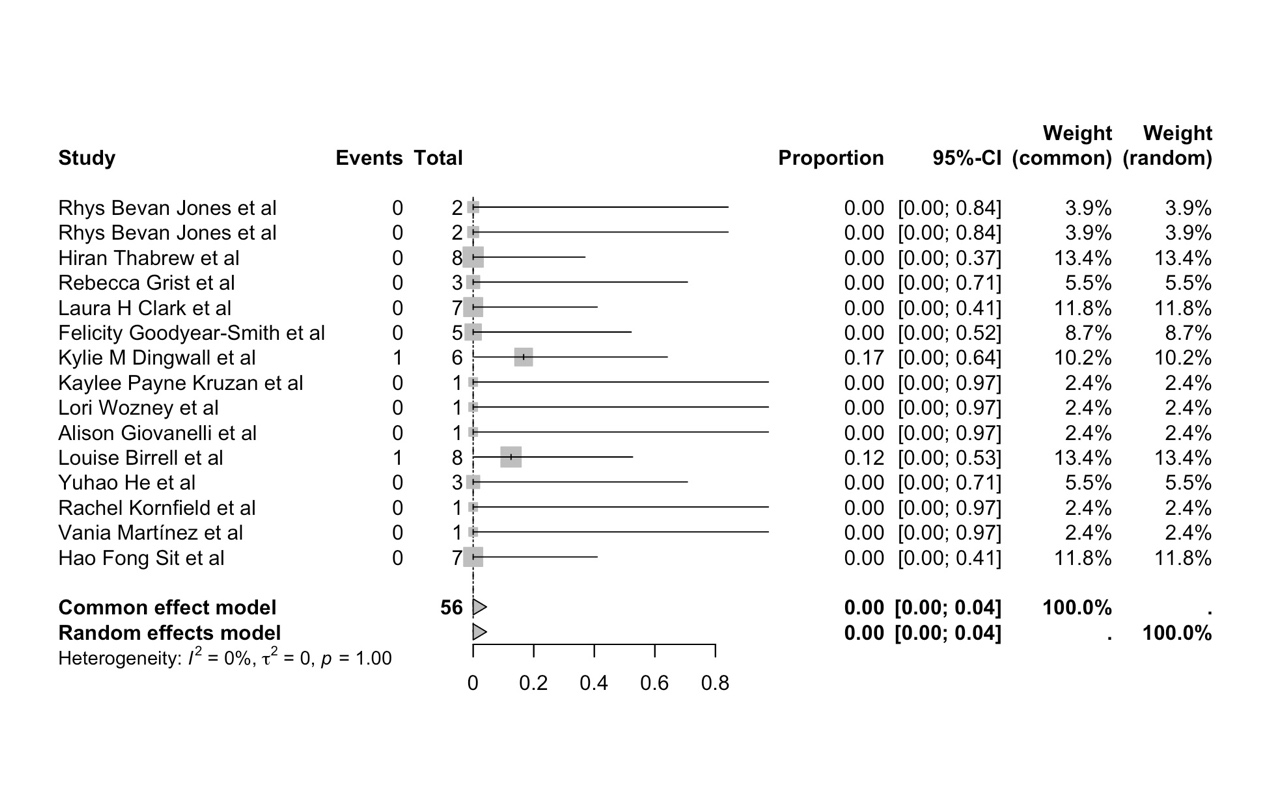


Figure 4l. Proportion of No/Limited Time (B12) in PD group


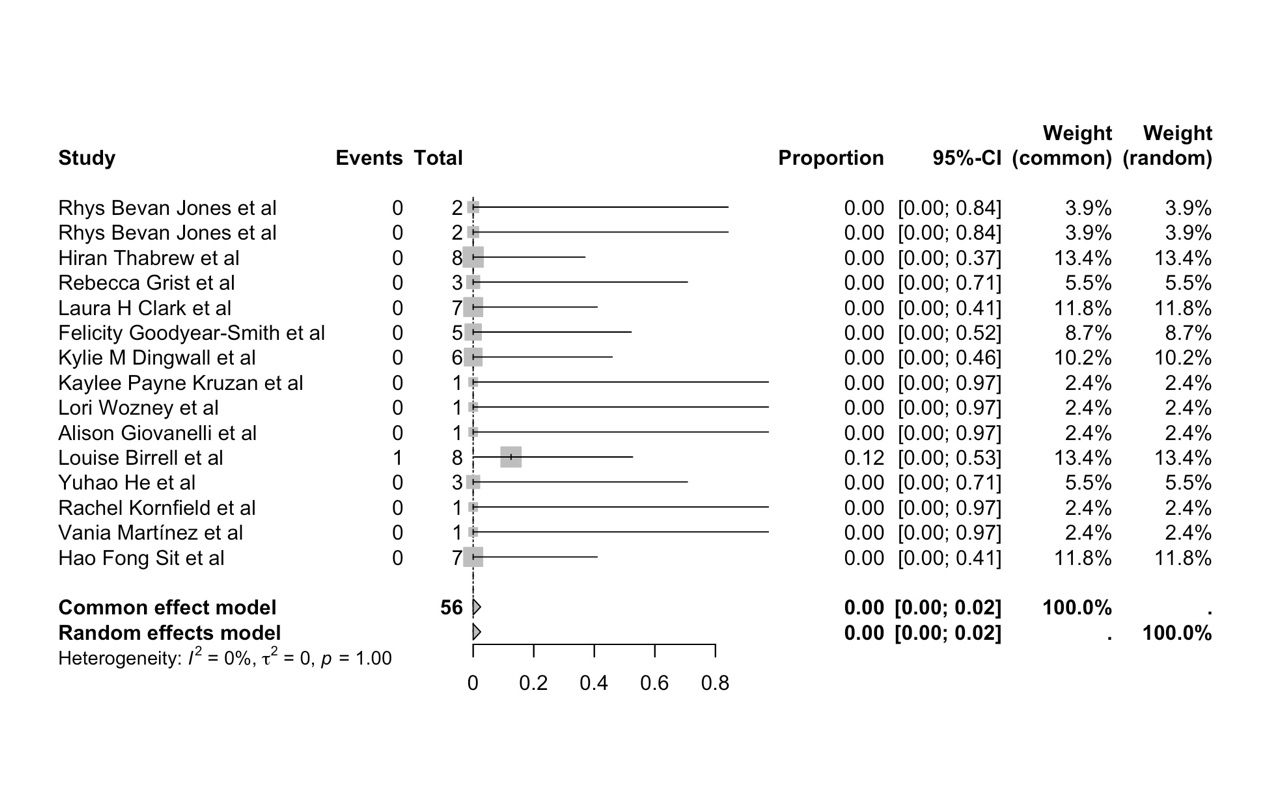


Figure 4m. Proportion of Technical Issues (B13) in PD group

Single Platform (SP)

Facilitators


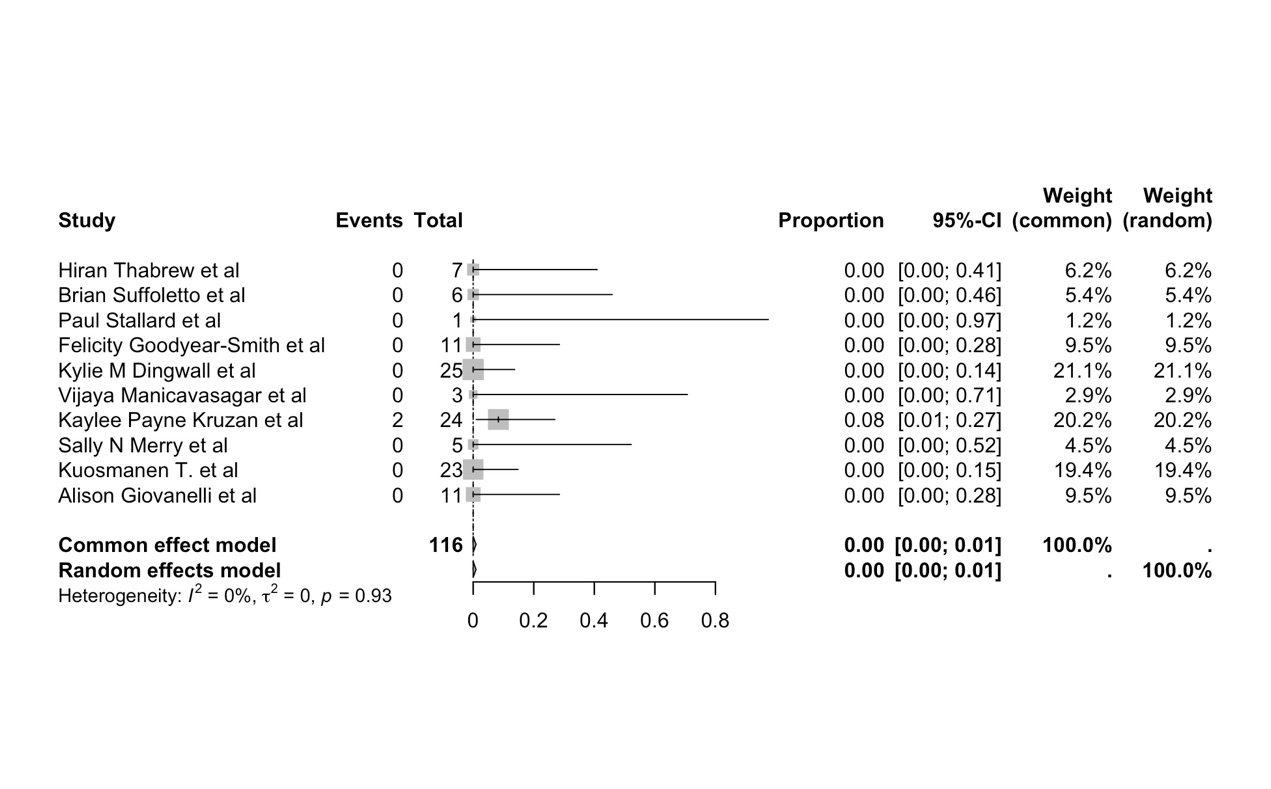


Figure 5a. Proportion of Integration with Schools and Other Resources (F1) in SP group


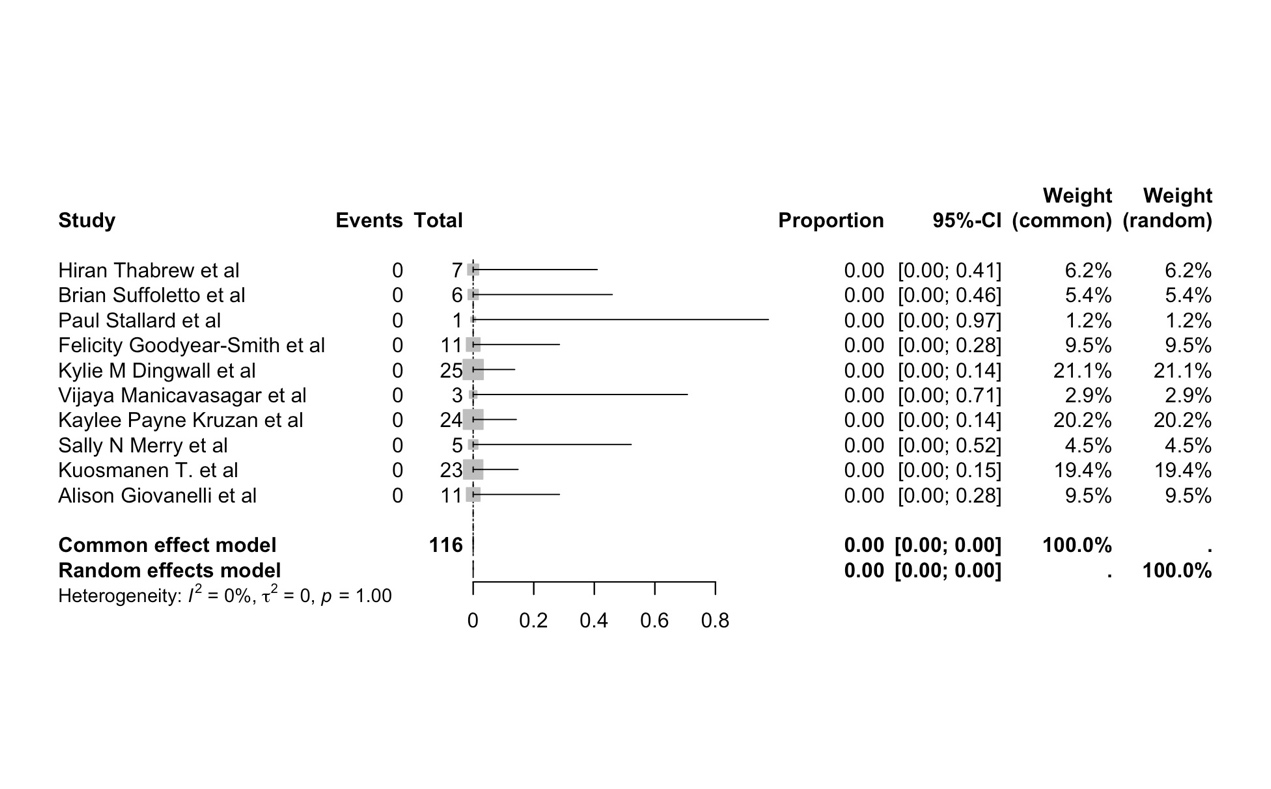


Figure 5b. Proportion of Social Norms (F2) in SP group


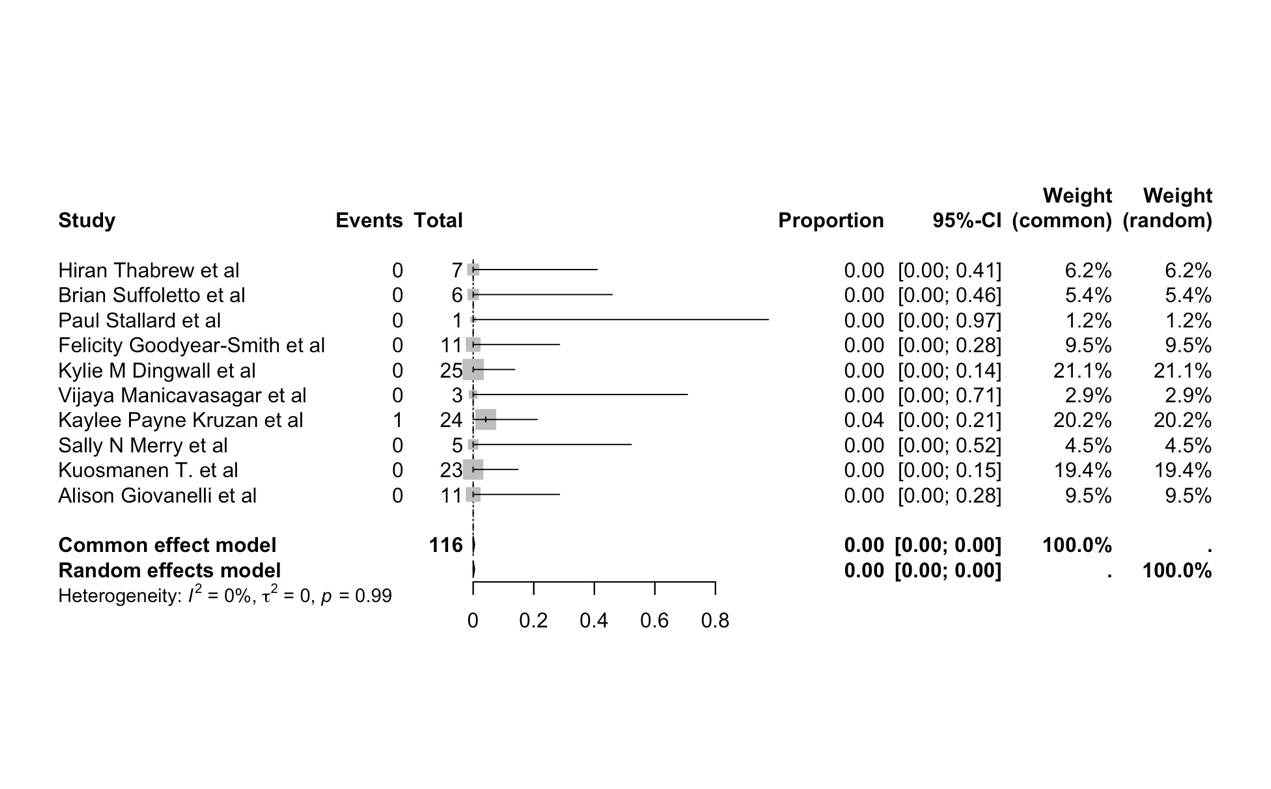


Figure 5c. Proportion of Strategic Marketing (F3) in SP group


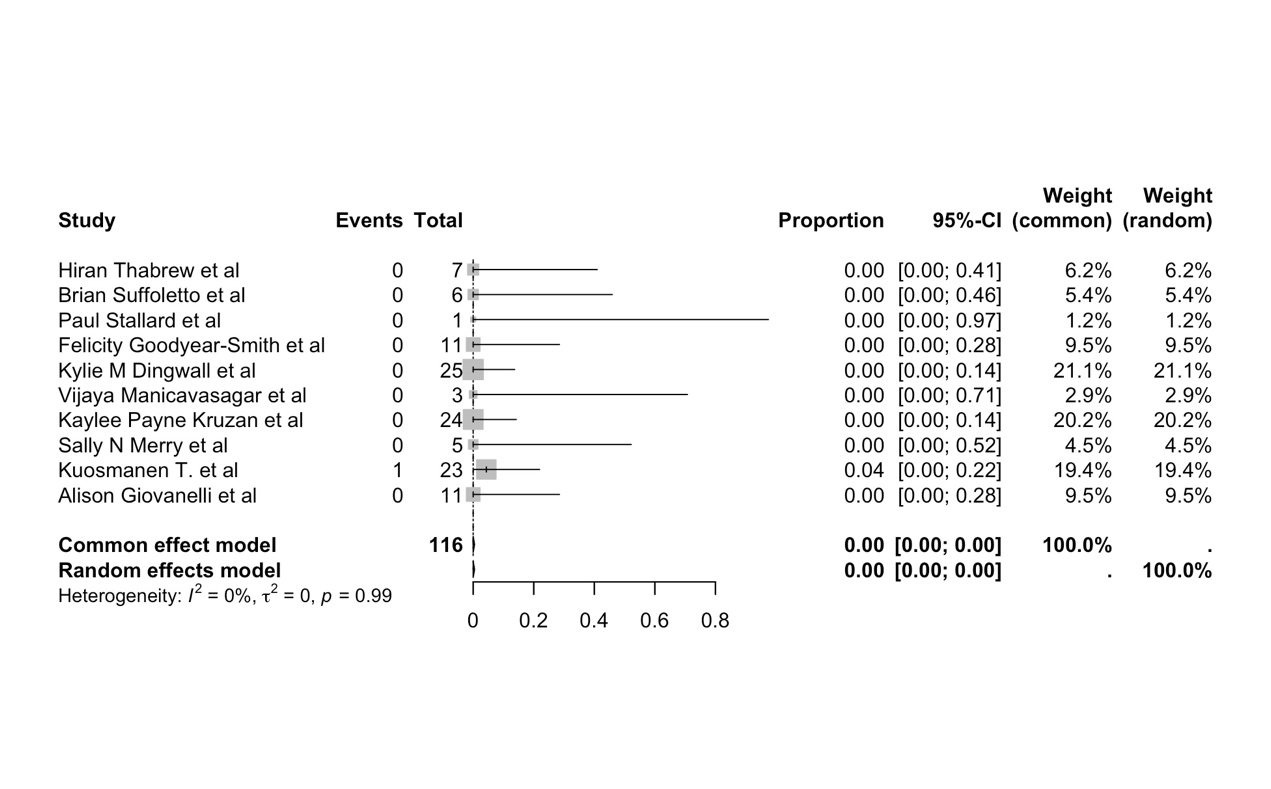


Figure 5d. Proportion of Universality (F4) in SP group


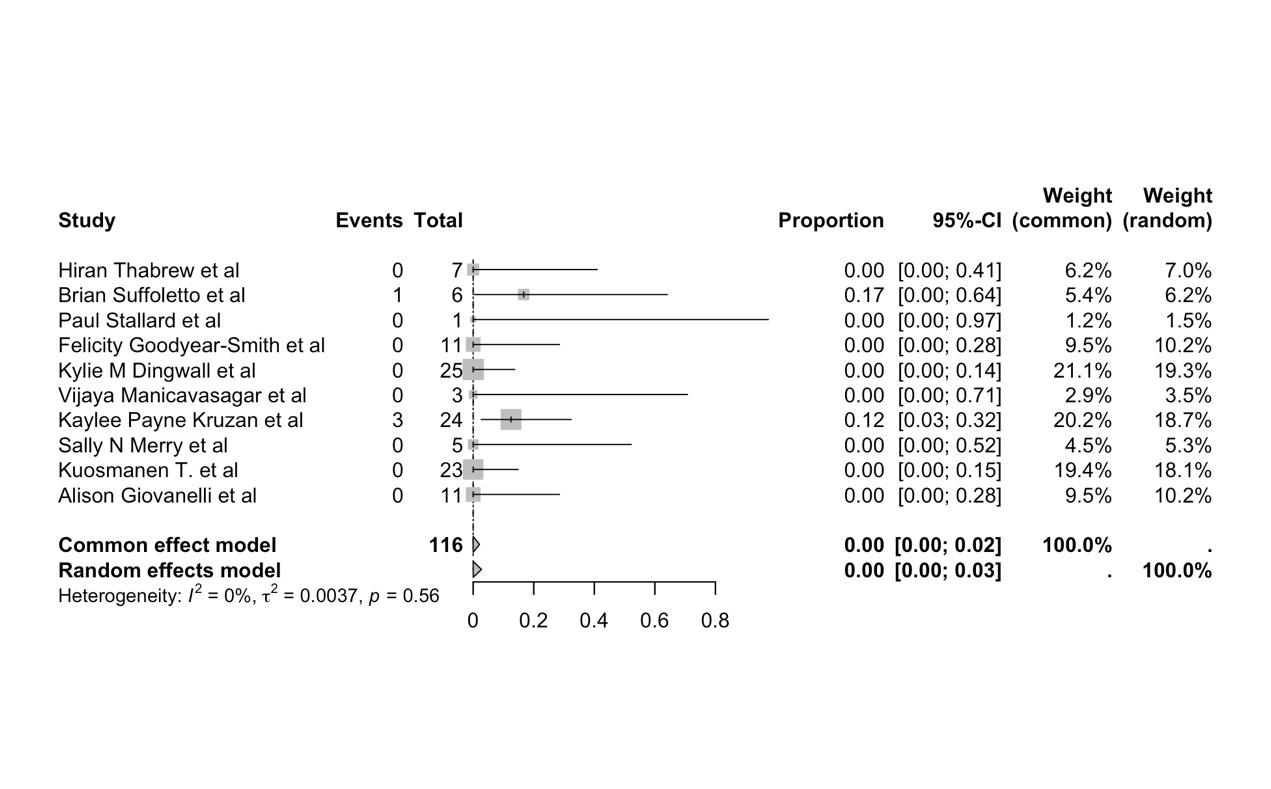


Figure 5e. Proportion of Endorsements (F5) in SP group


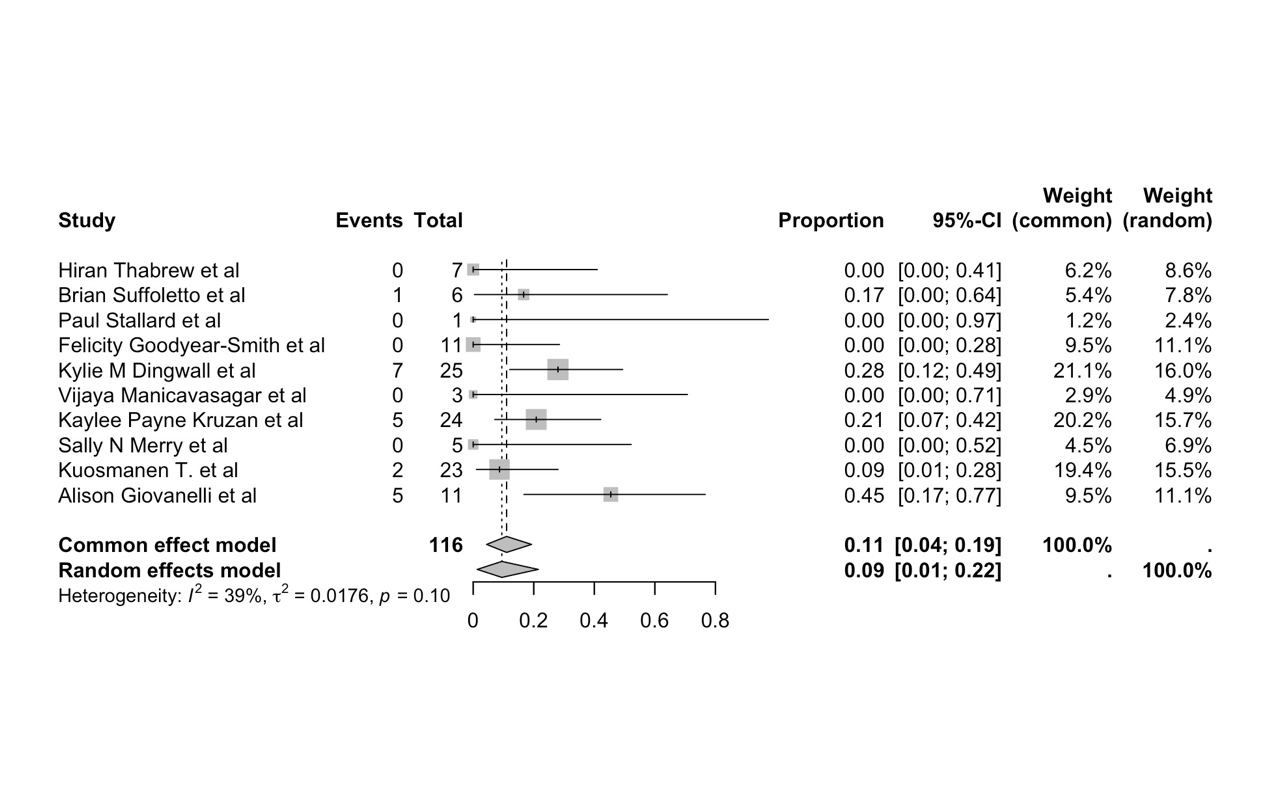


Figure 5f. Proportion of Content Engagement (F6) in SP group


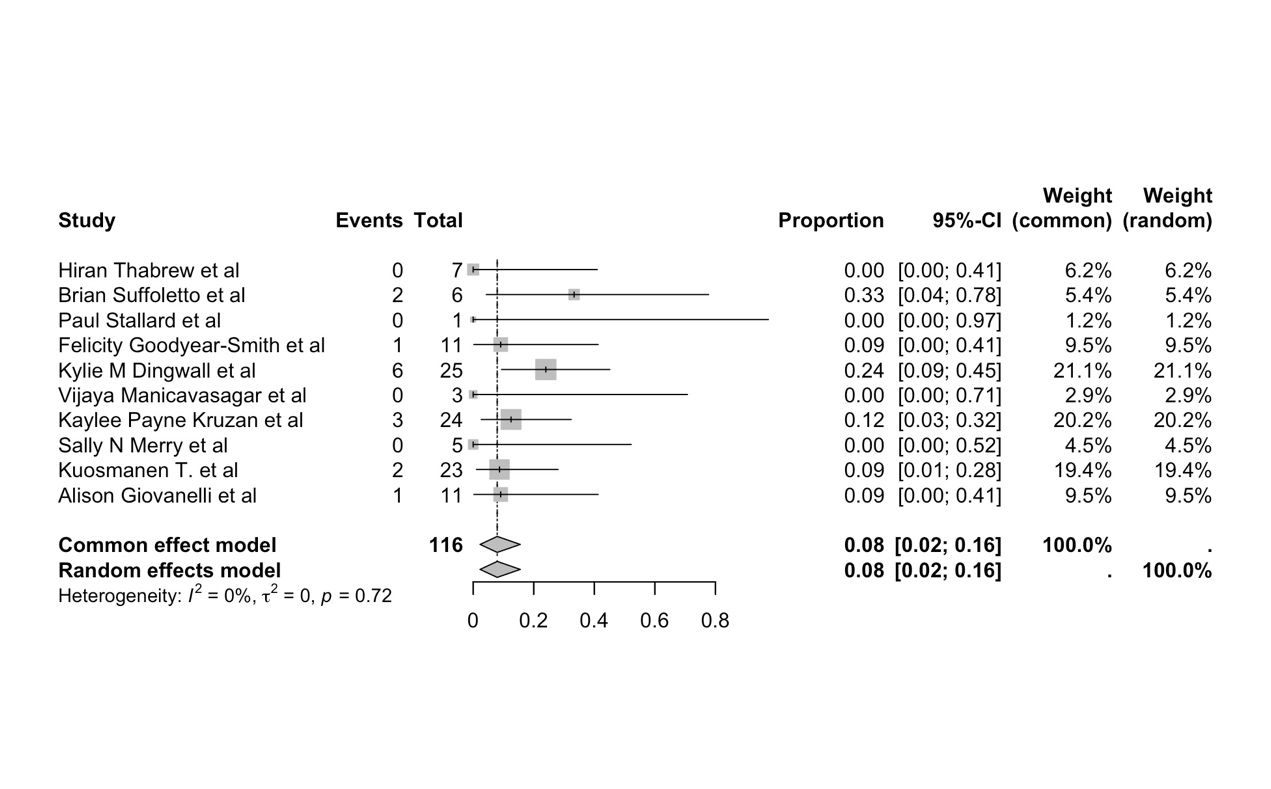


Figure 5g. Proportion of Design Harmony (F7) in SP group


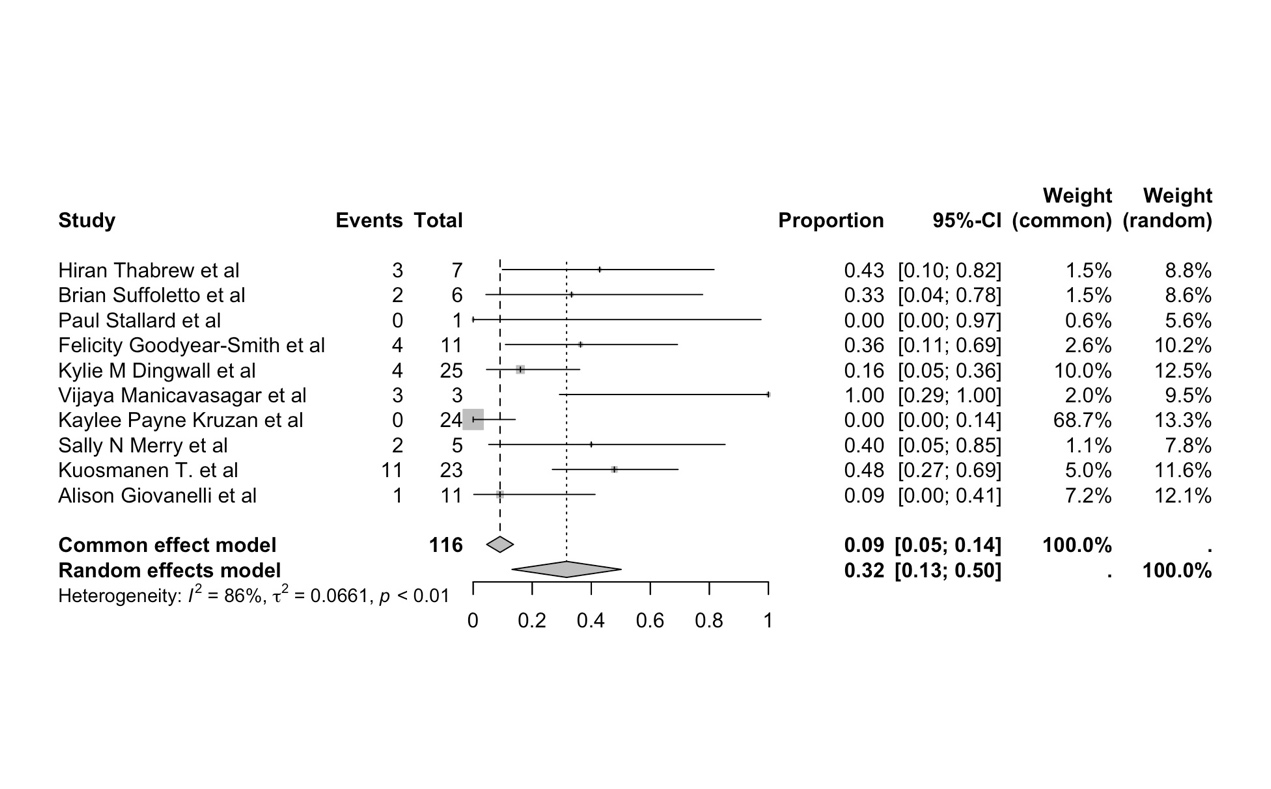


Figure 5h. Proportion of High Quality and Effect (F8) in SP group


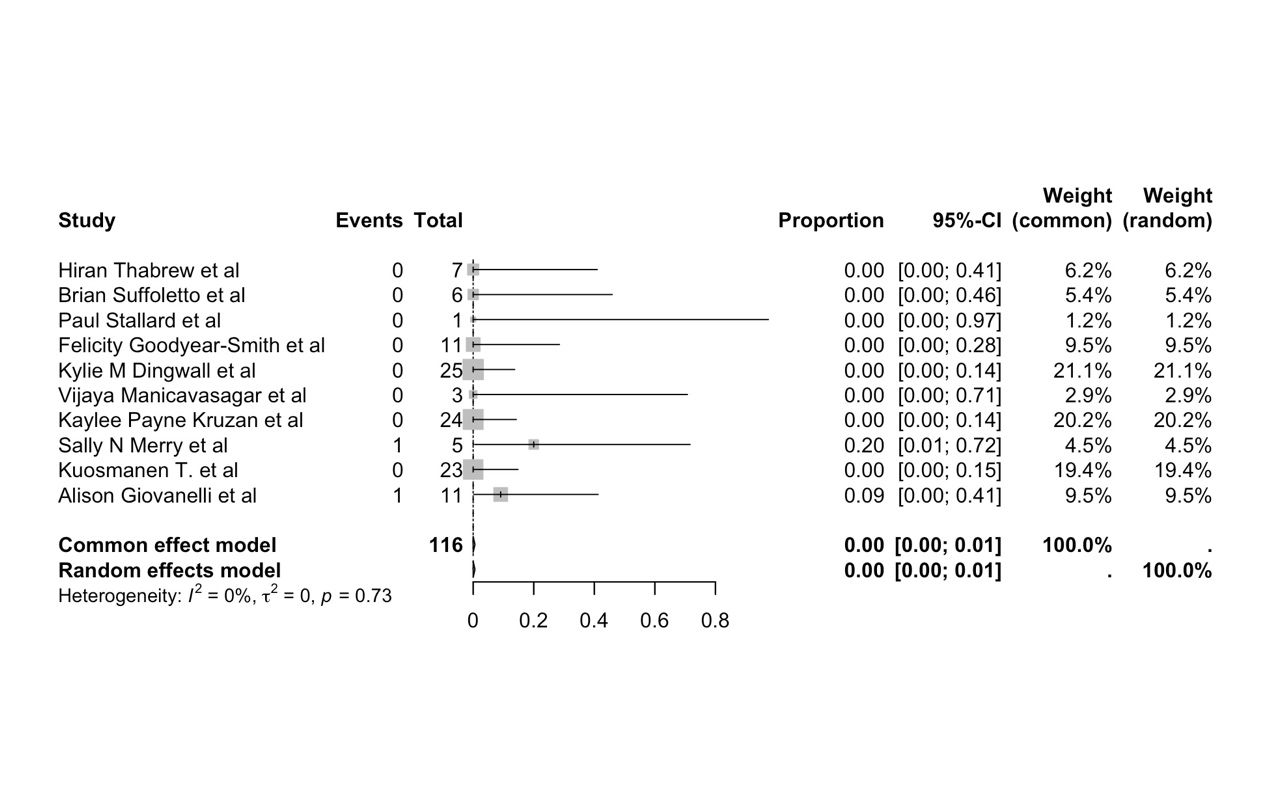


Figure 5i. Proportion of Appropriate Duration and Schedule (F9) in SP group


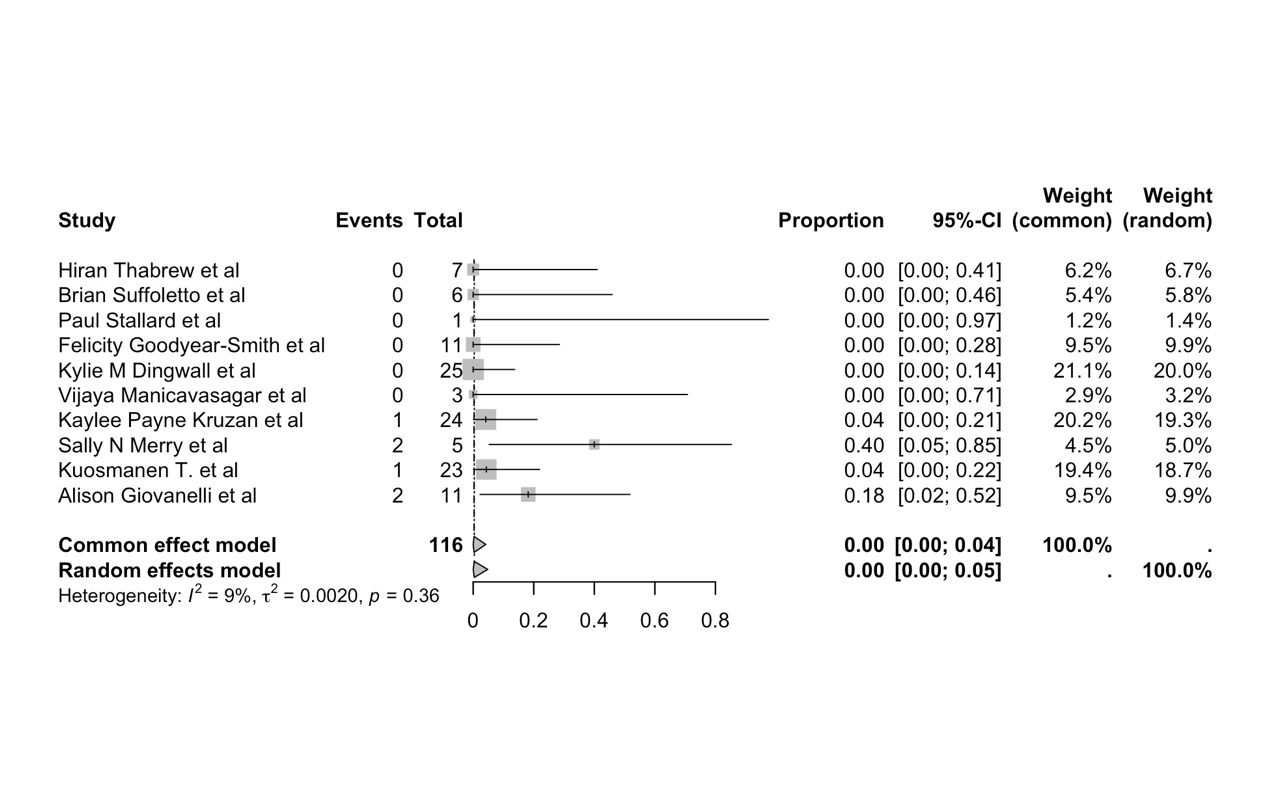


Figure 5j. Proportion of Accessibility (F10) in SP group


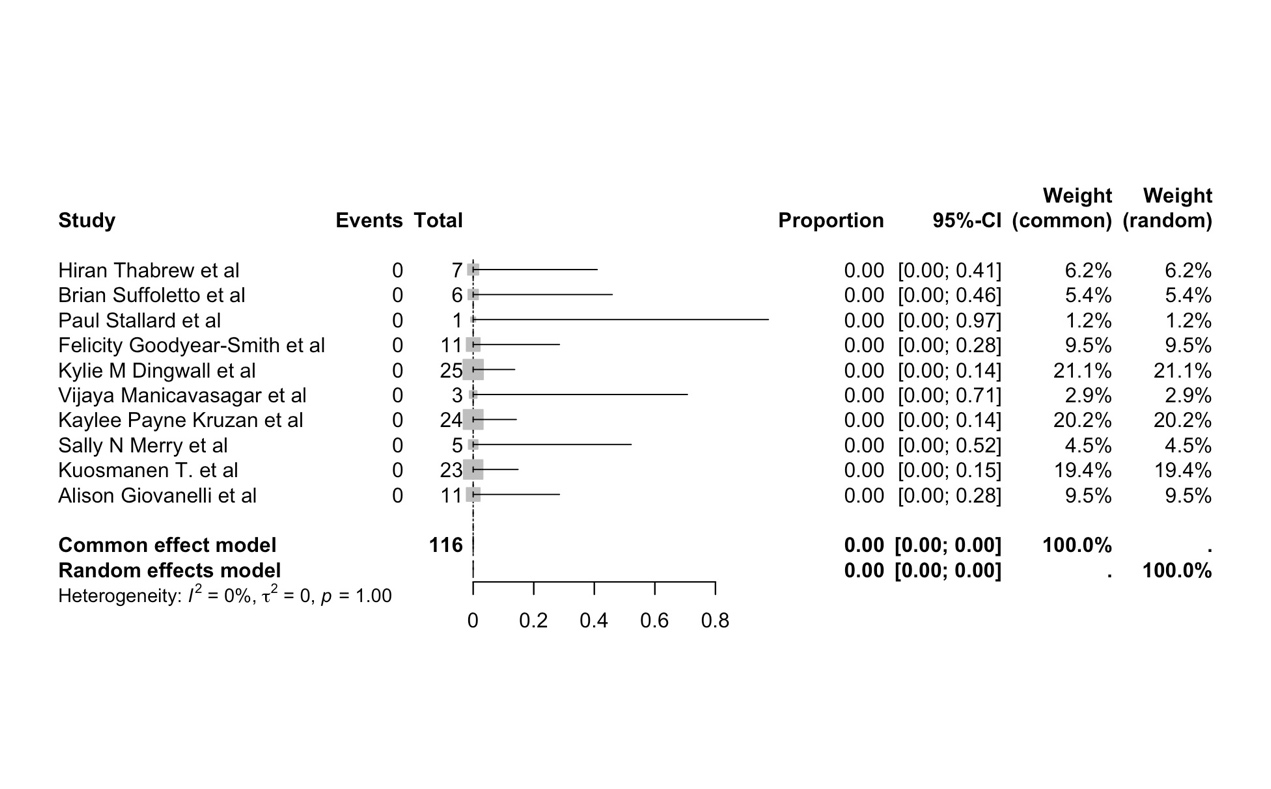


Figure 5k. Proportion of Beneficial Characteristics (F11) in SP group


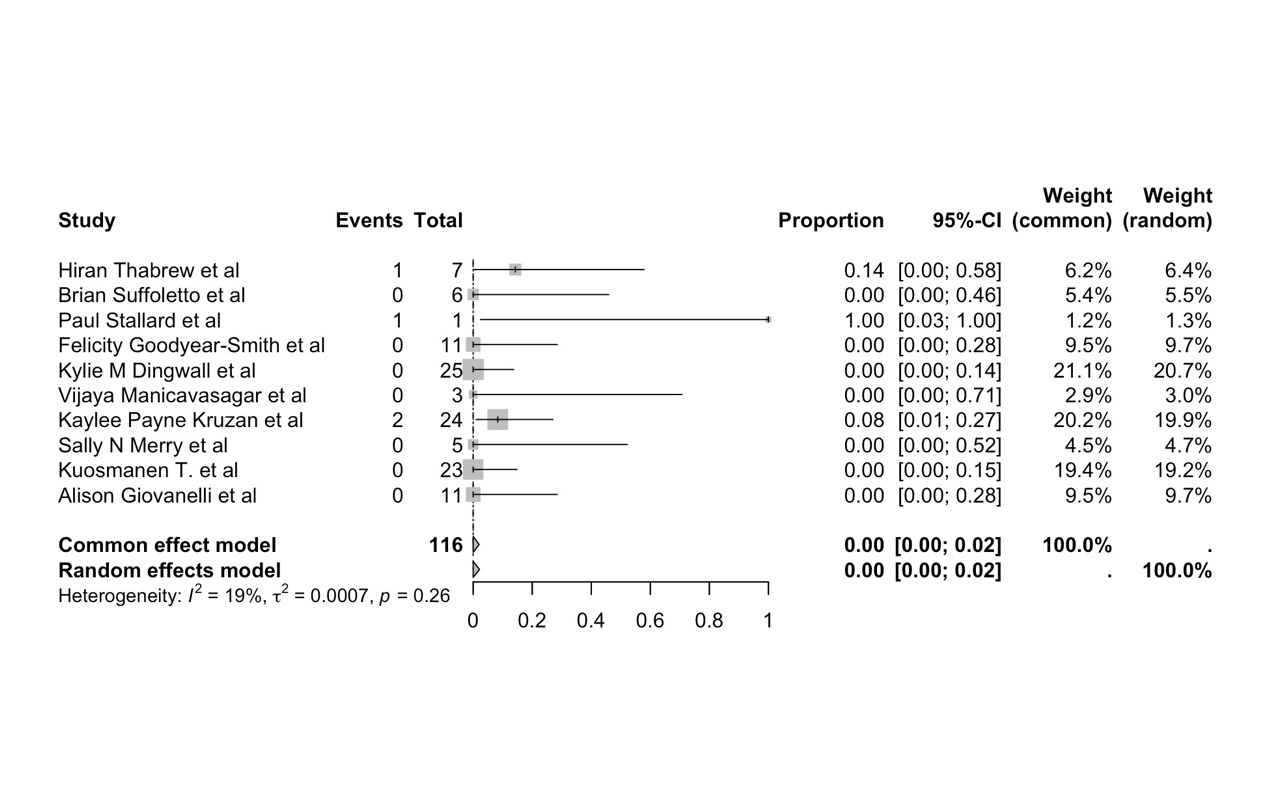


Figure 5l. Proportion of Needs and Disposition (F12) in SP group


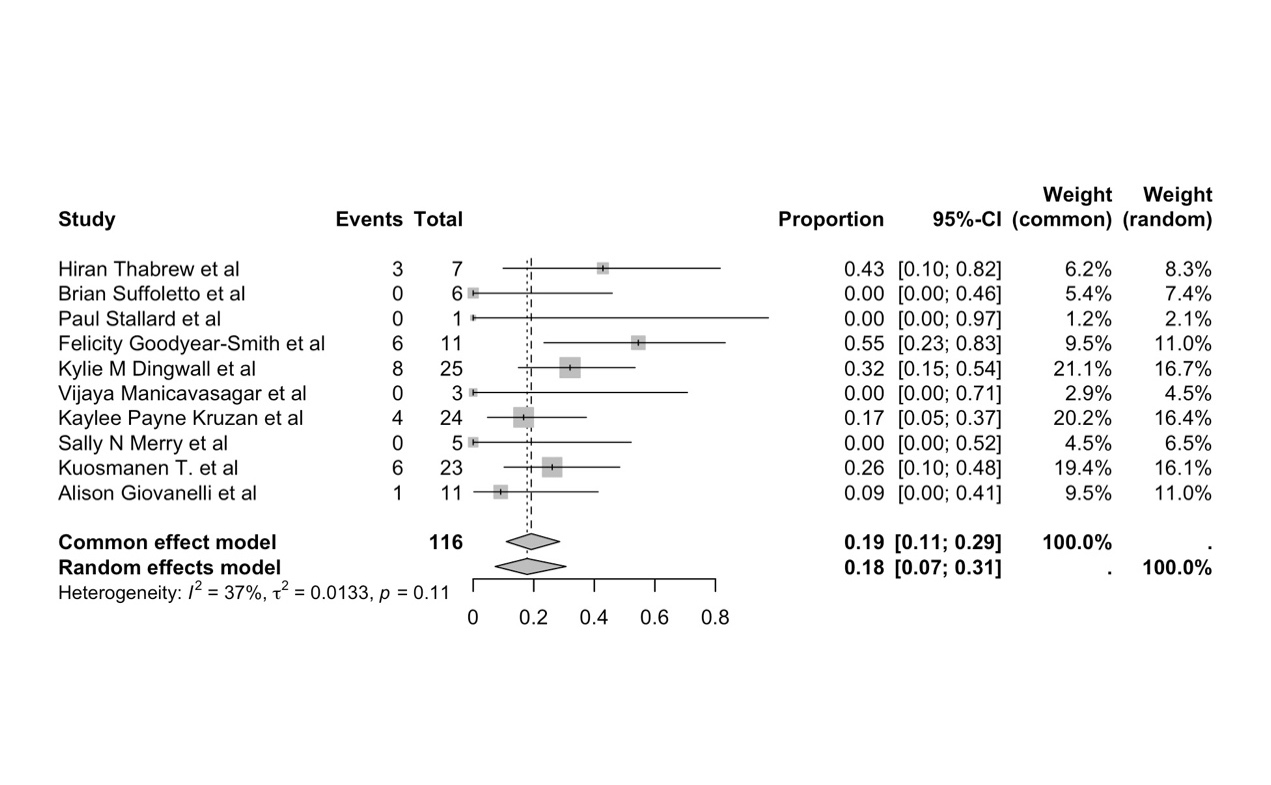


Figure 5m. Proportion of Perceived Benefits (F13) in SP group


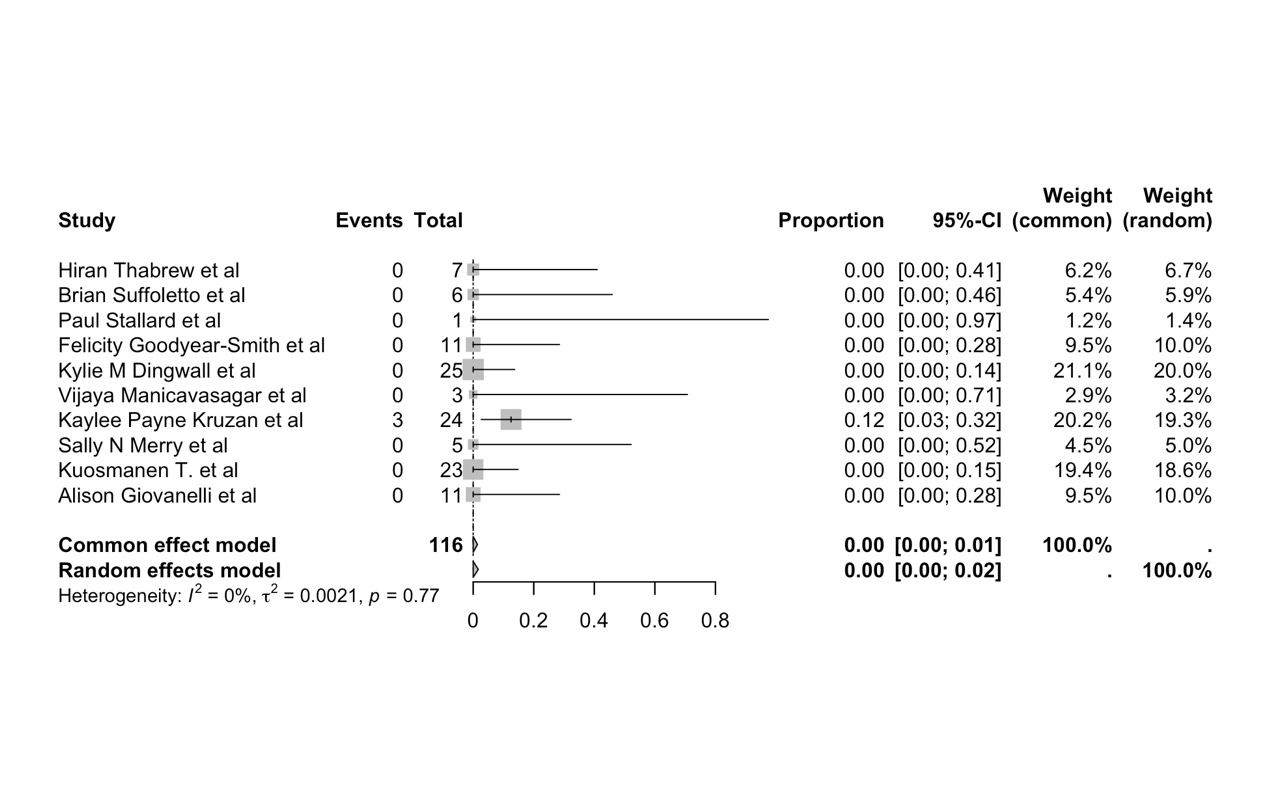


Figure 5n. Proportion of Supportive Environment (F14) in SP group

Single Platform (SP)

Barriers


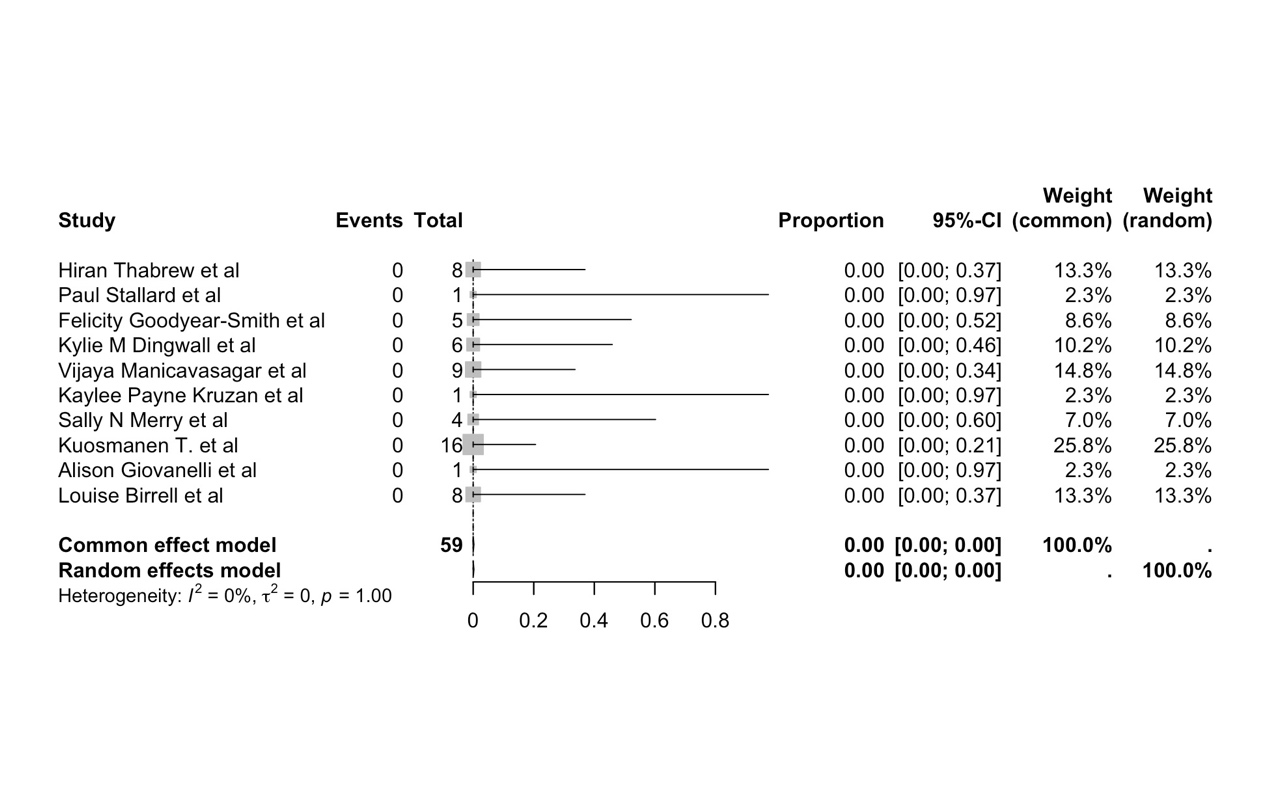


Figure 6a. Proportion of Integration with Schools (B1), Content Gaps (B2) in SP group


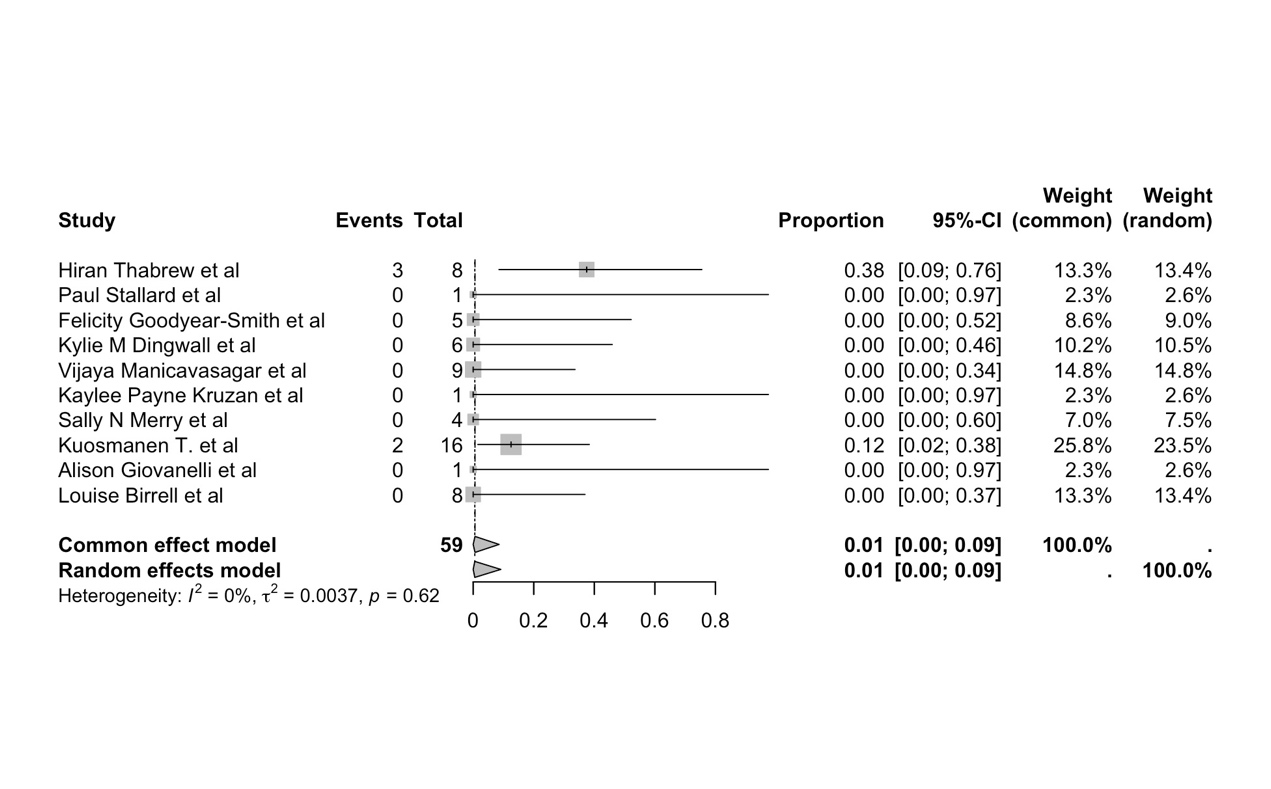


Figure 6b. Proportion of Design Limitations (B3) in SP group


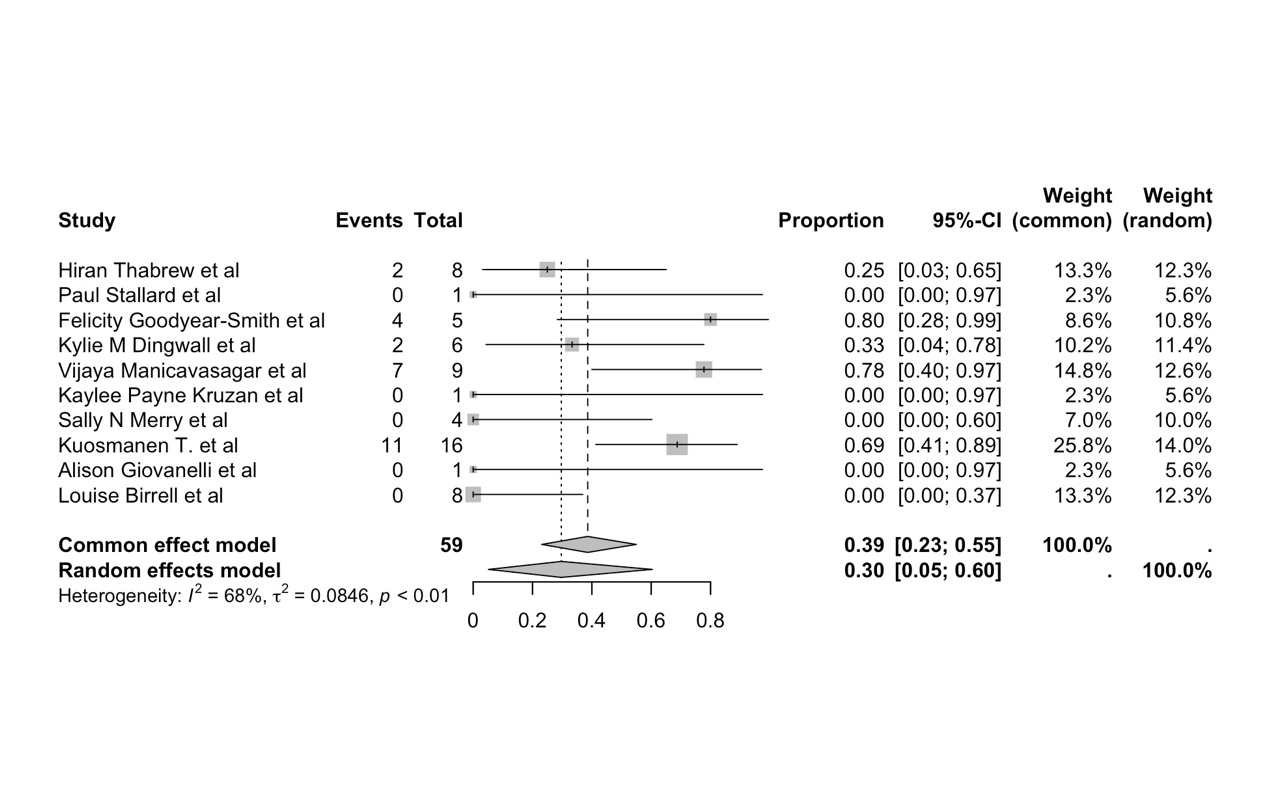


Figure 6c. Proportion of Low Quality and Effect (B4) in SP group


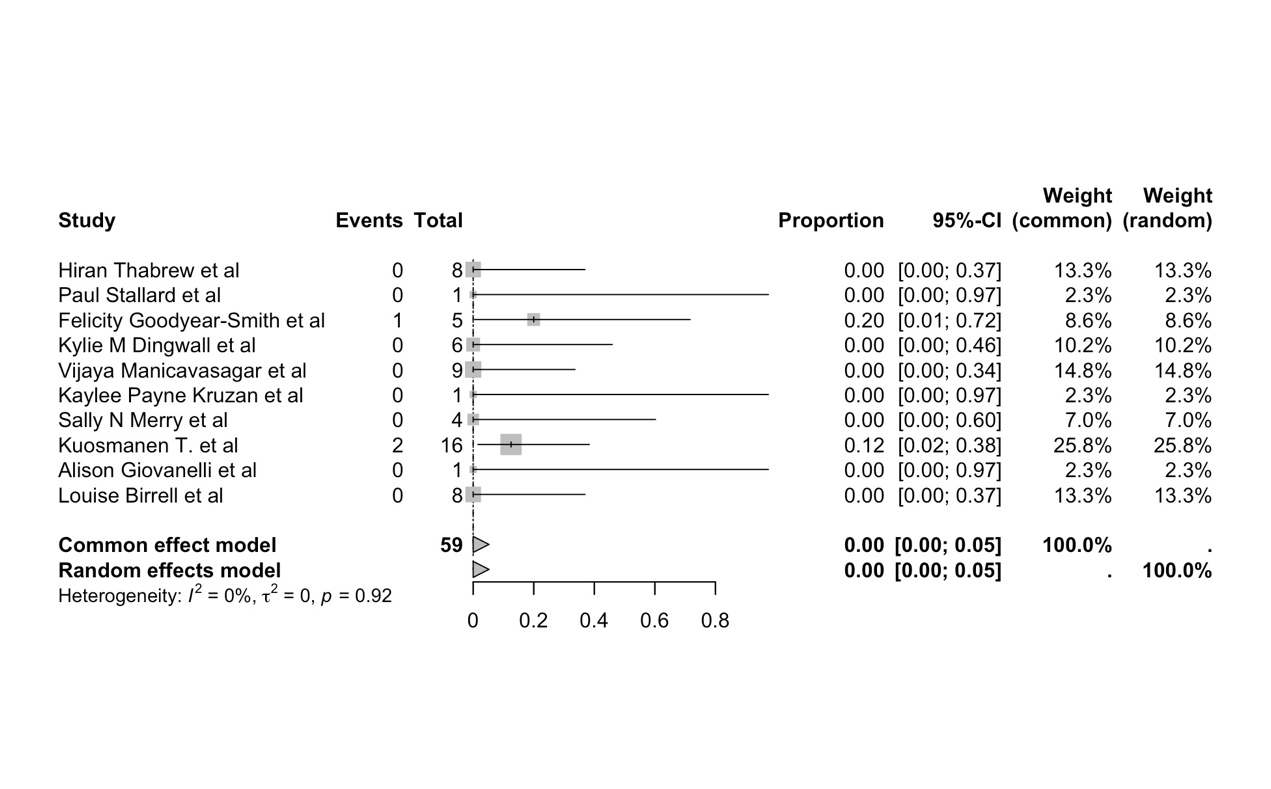


Figure 6d. Proportion of Inappropriate Duration and Schedule (B5) in SP group


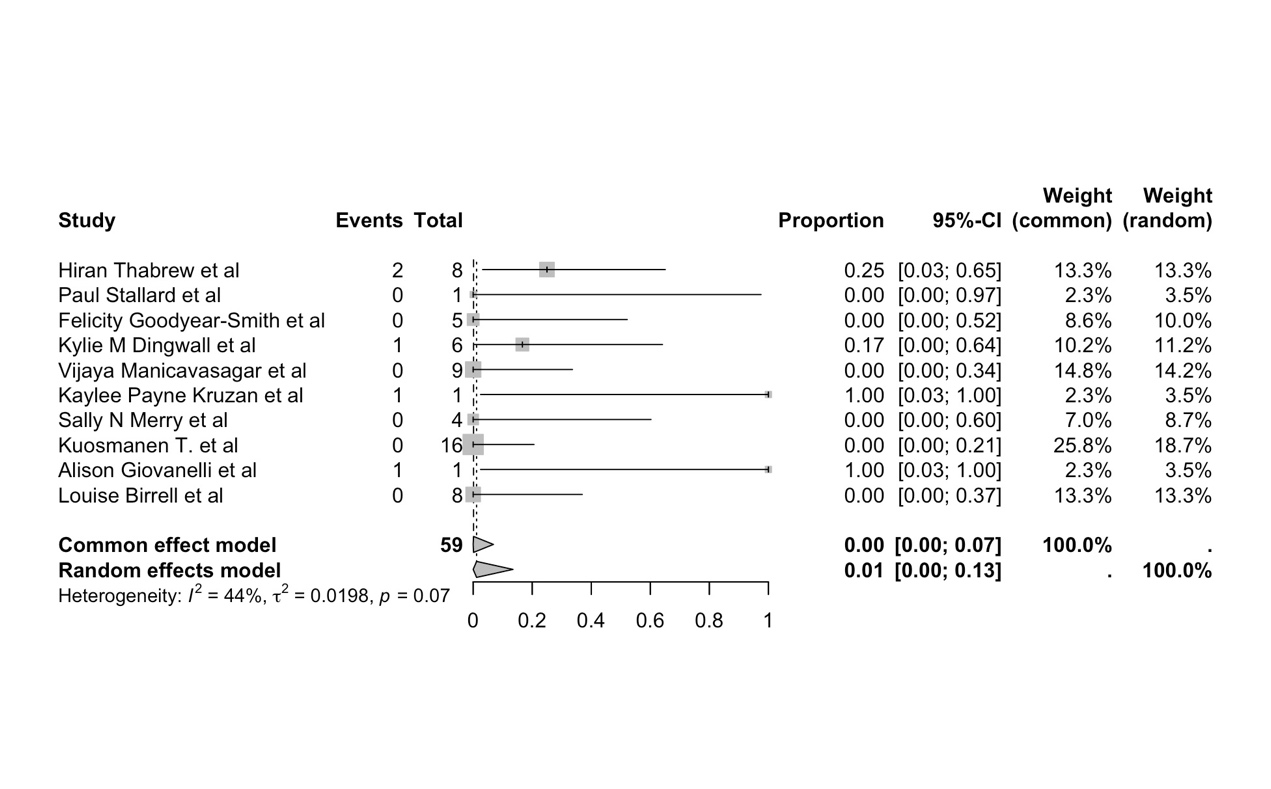


Figure 6e. Proportion of Inaccessibility (B6) in SP group


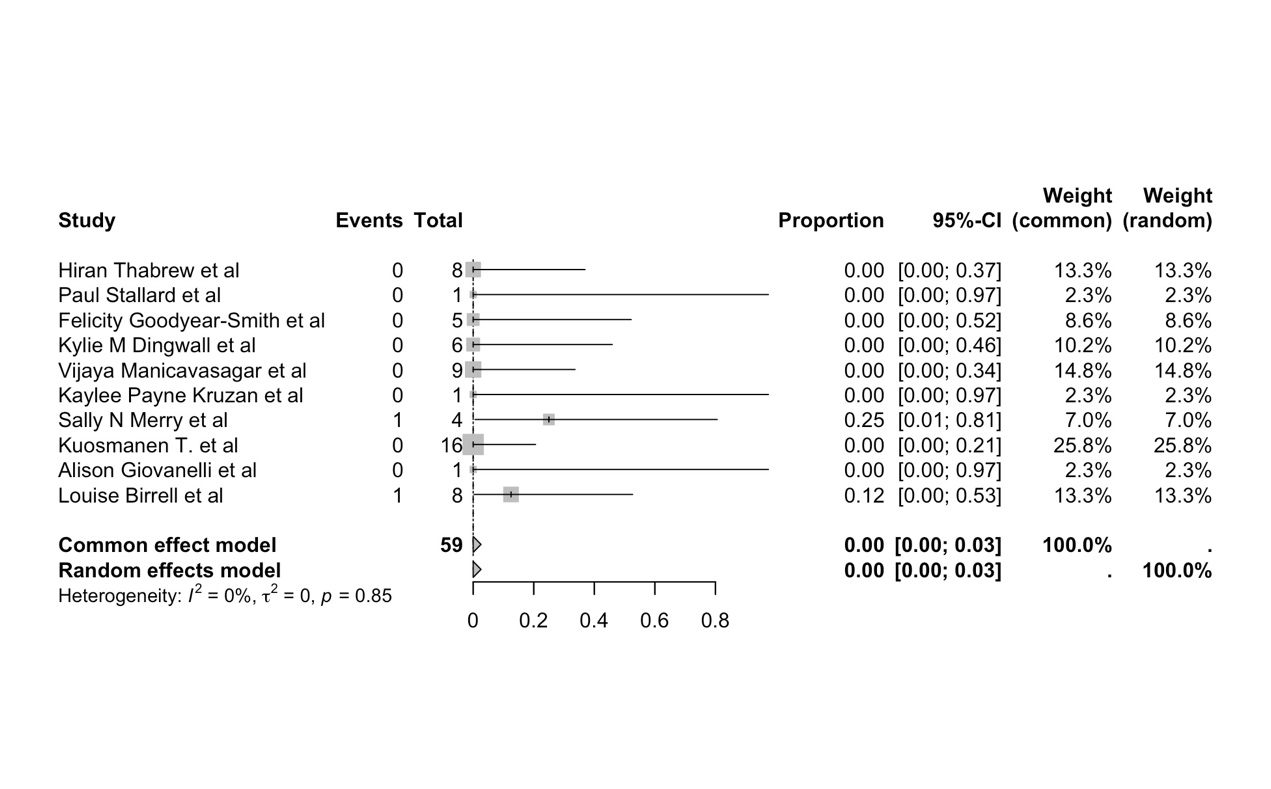


Figure 6f. Proportion of Detrimental Characteristics (B7) in SP group


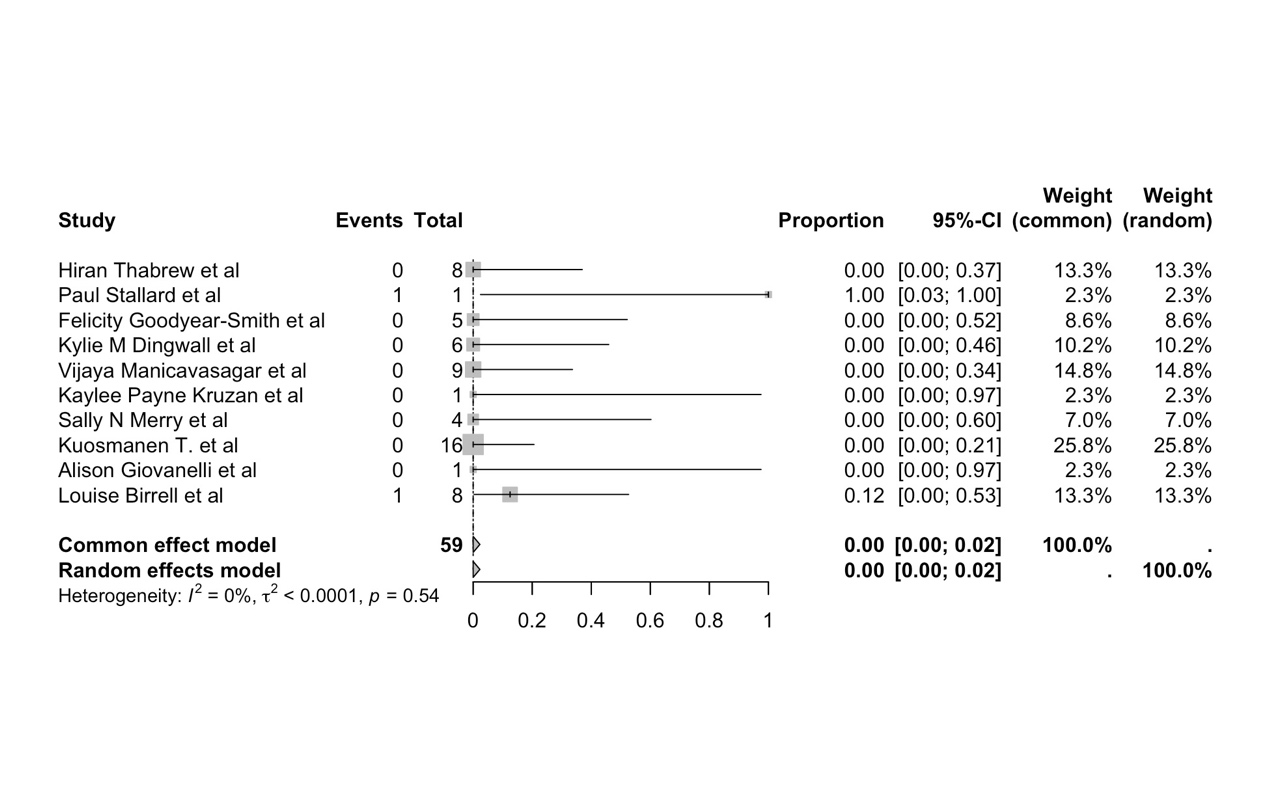


Figure 6g. Proportion of Motivational Challenges (B8) in SP group


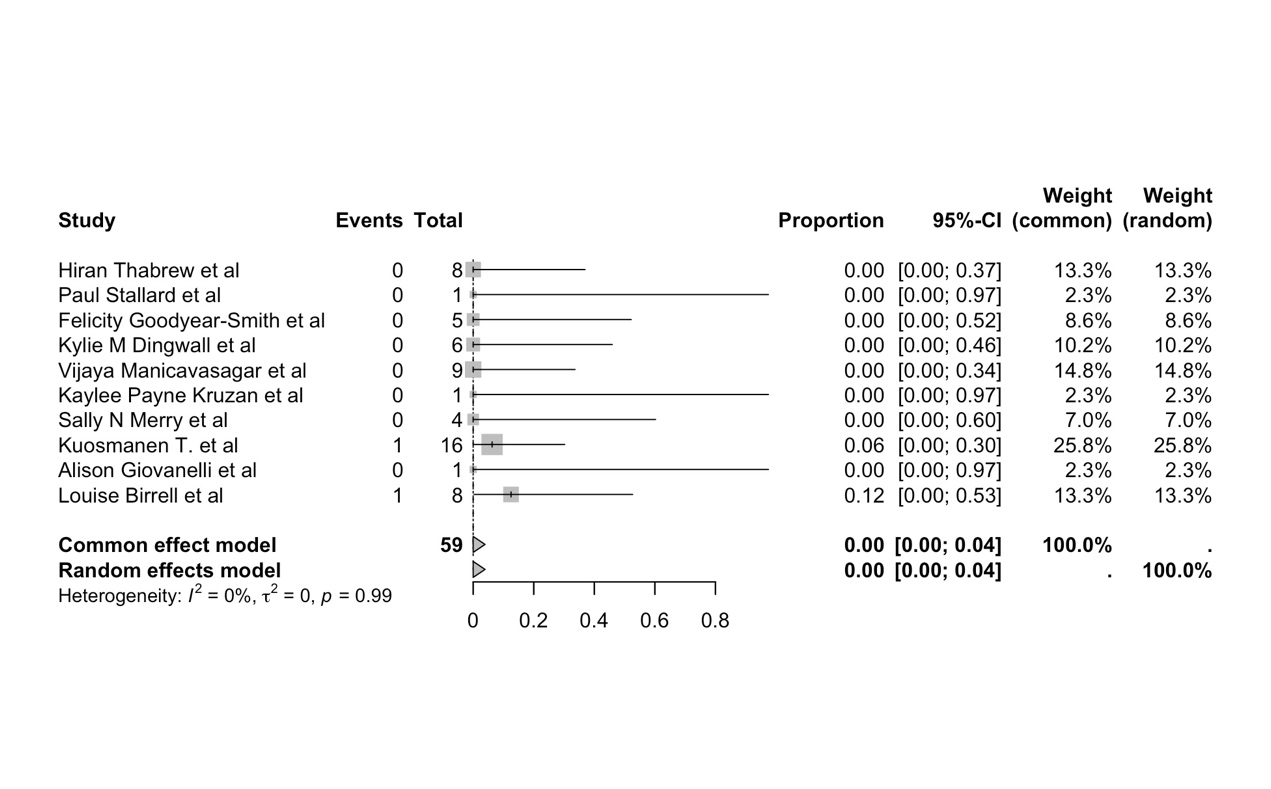


Figure 6h. Proportion of Perceived Risks (B9) in SP group


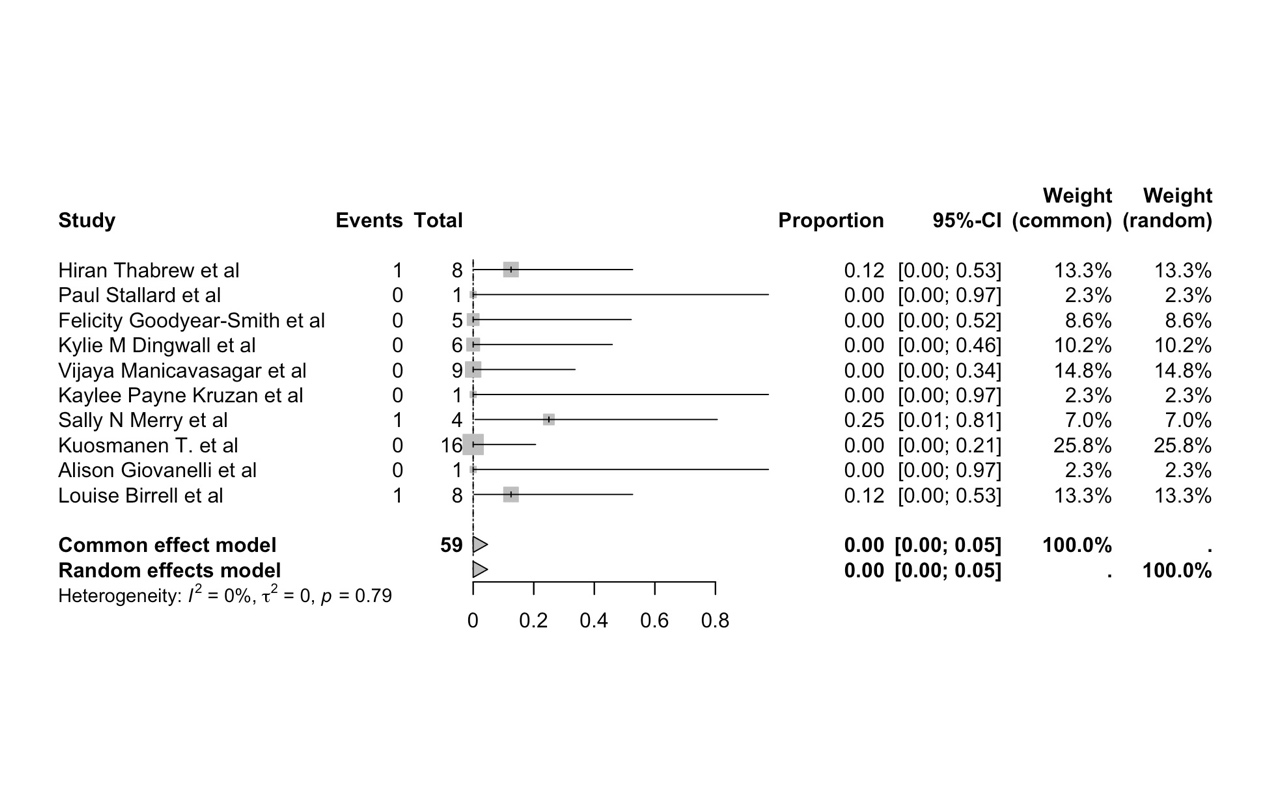


Figure 6i. Proportion of Question (B10) in SP group


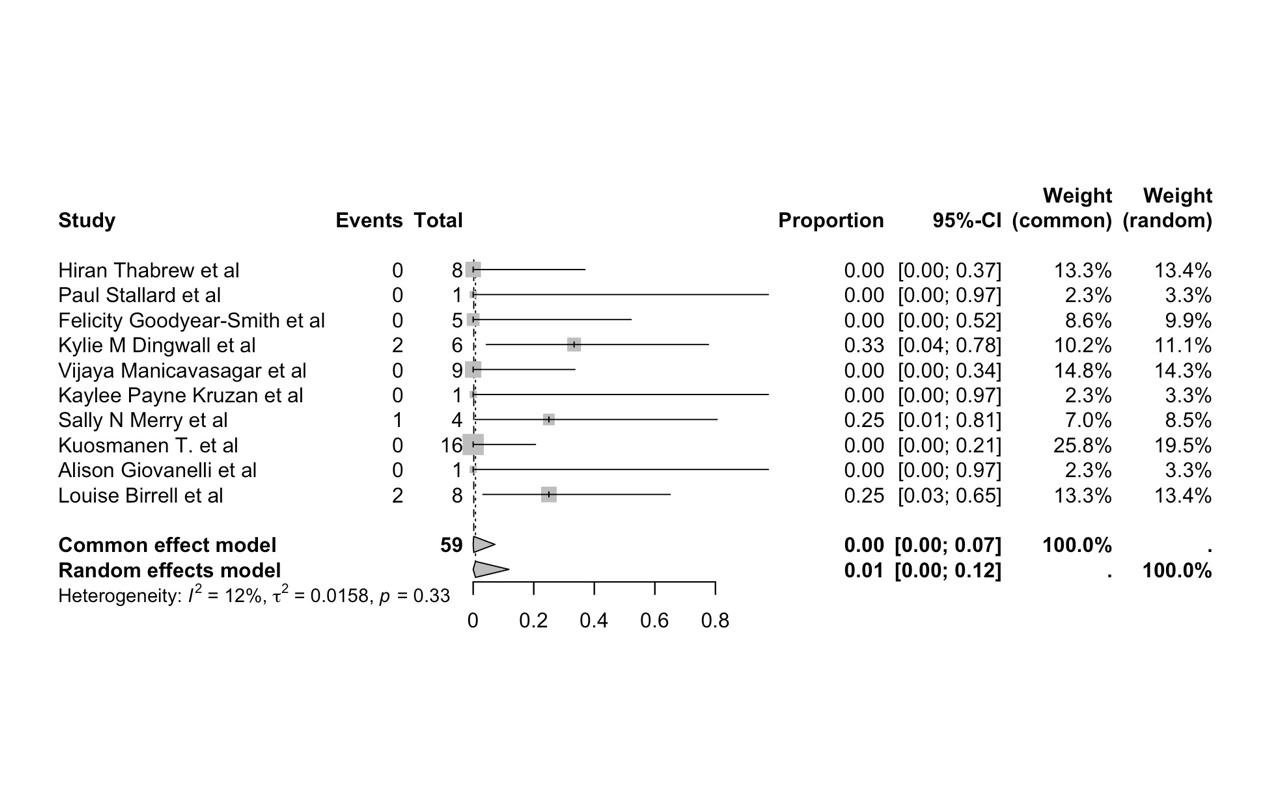


Figure 6j. Proportion of Retention Issues (B11) in SP group


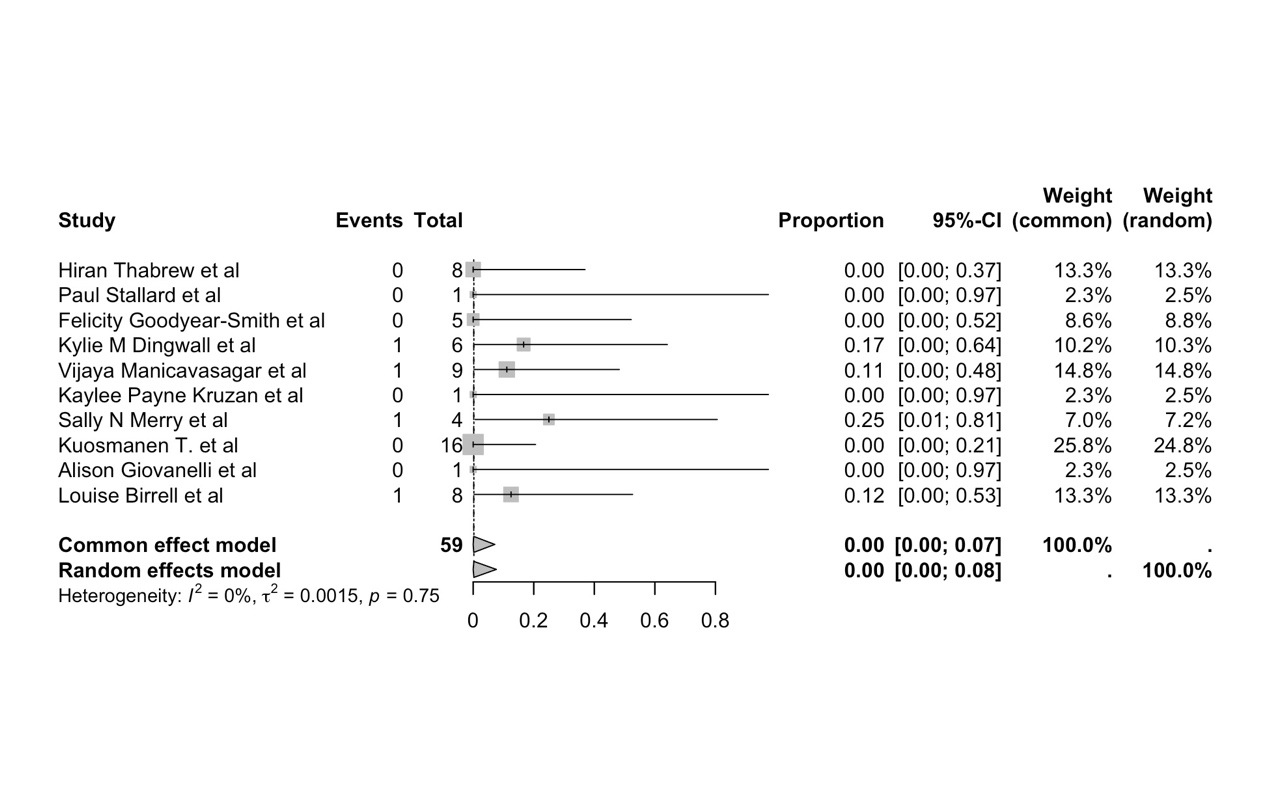


Figure 6k. Proportion of No/Limited Time (B12) in SP group


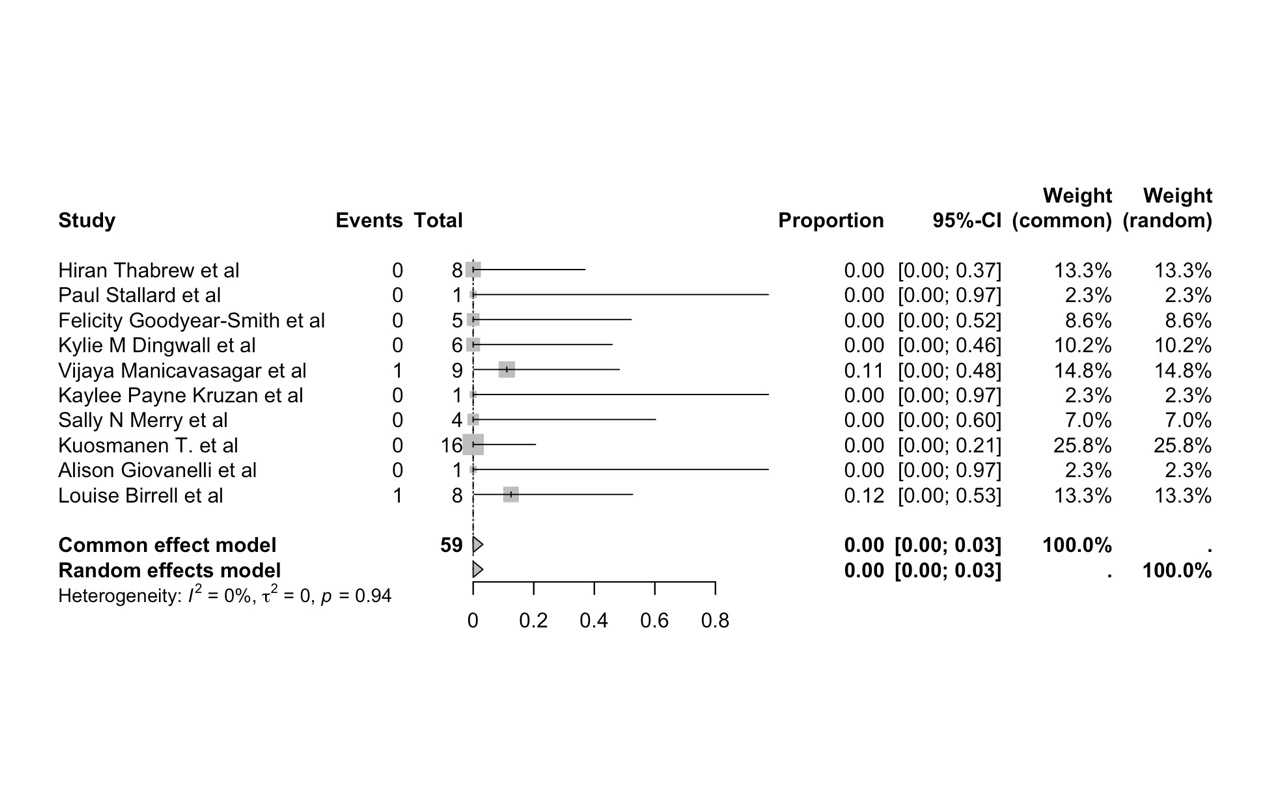


Figure 6l. Proportion of Technical Issues (B13) in SP group

Multiple Platforms (MP)

Facilitators


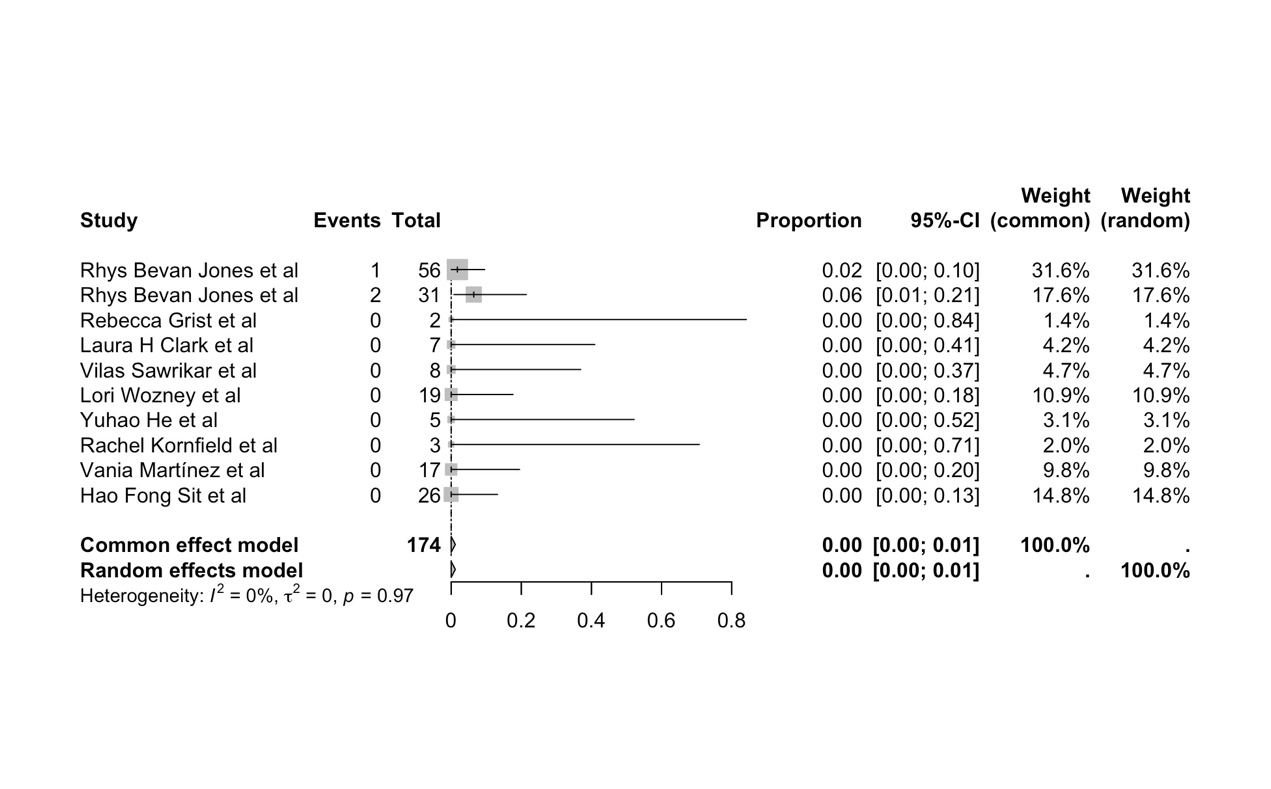


Figure 7a. Proportion of Integration with Schools and Other Resources (F1) in MP group


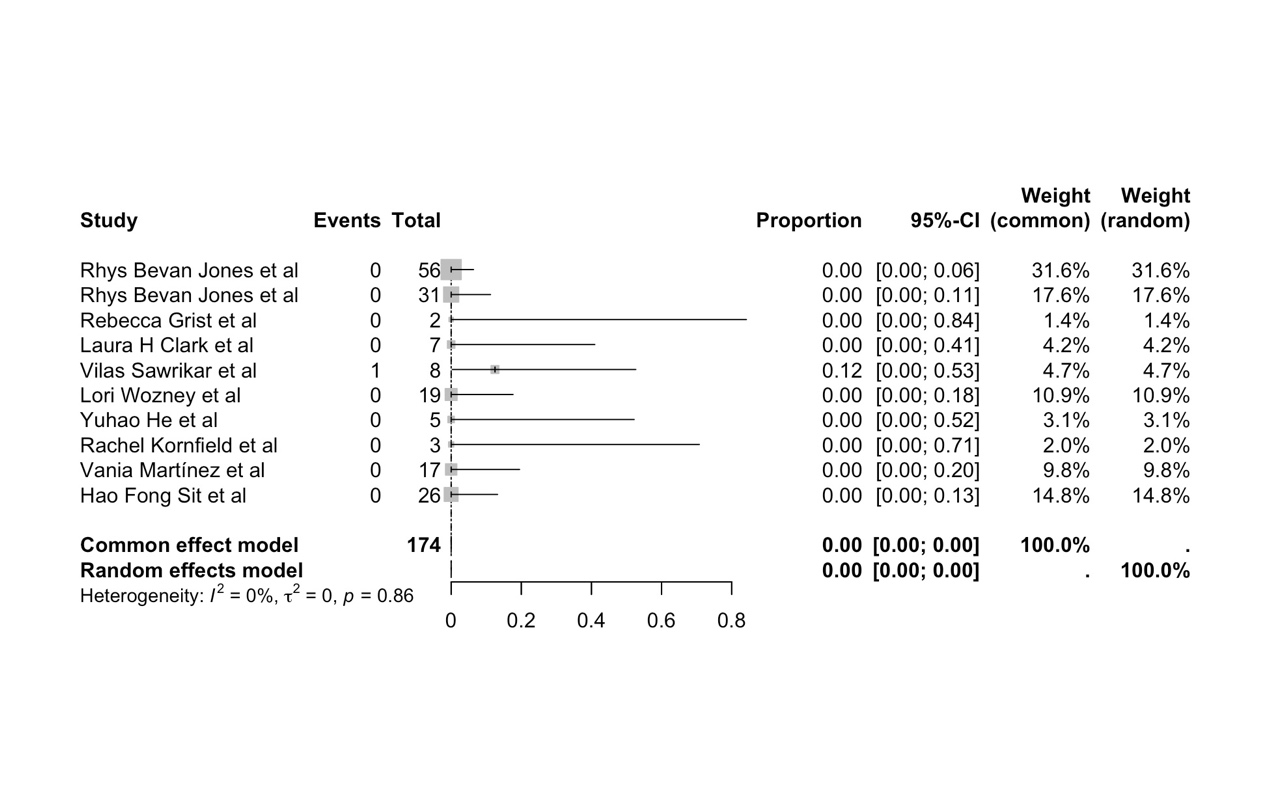


Figure 7b. Proportion of Social Norms (F2) in MP group


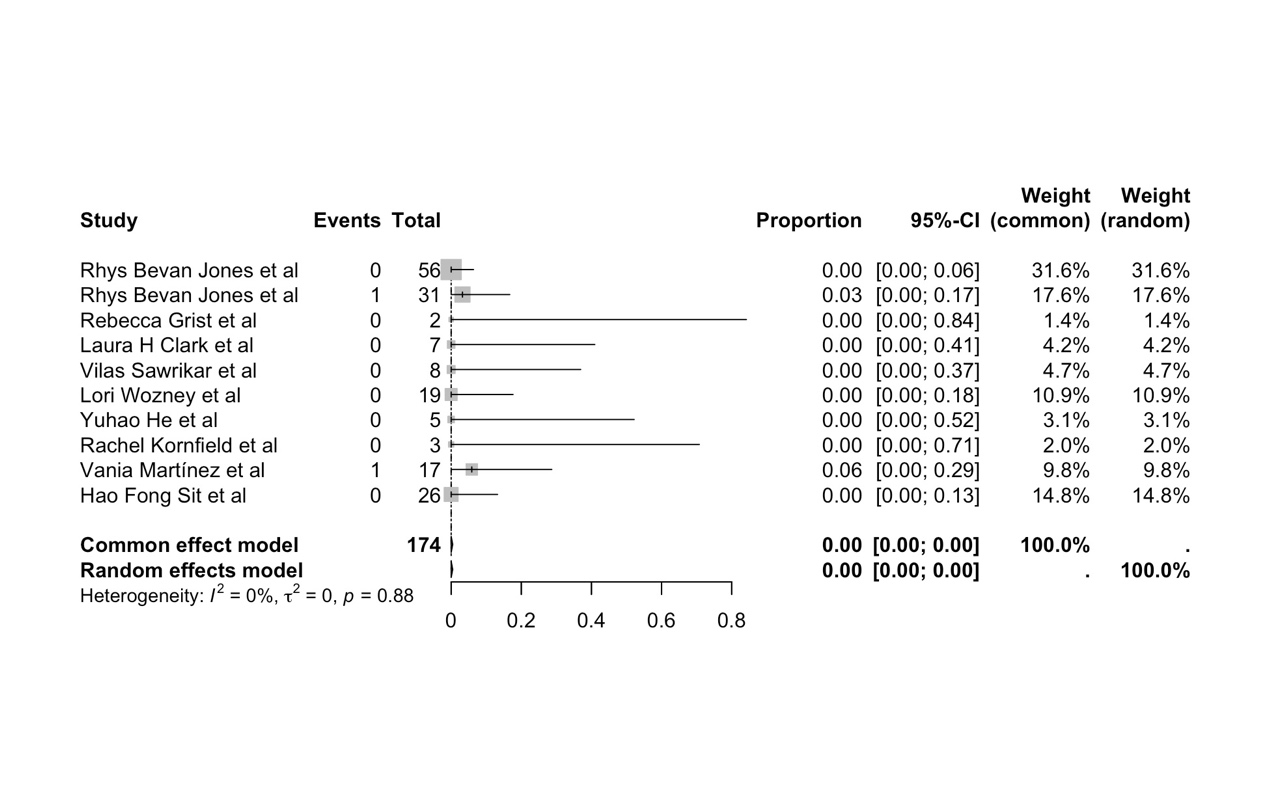


Figure 7c. Proportion of Strategic Marketing (F3) in MP group


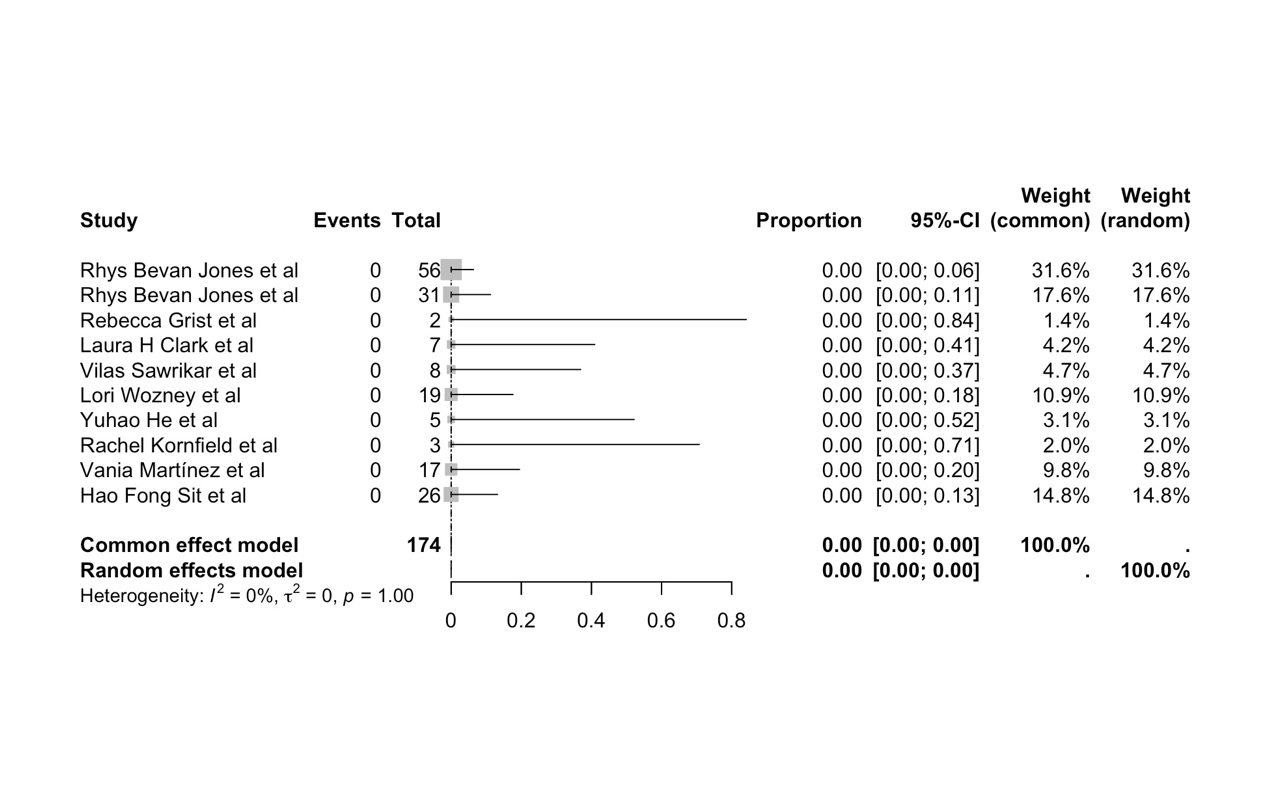


Figure 7d. Proportion of Universality (F4), Endorsements (F5) in MP group


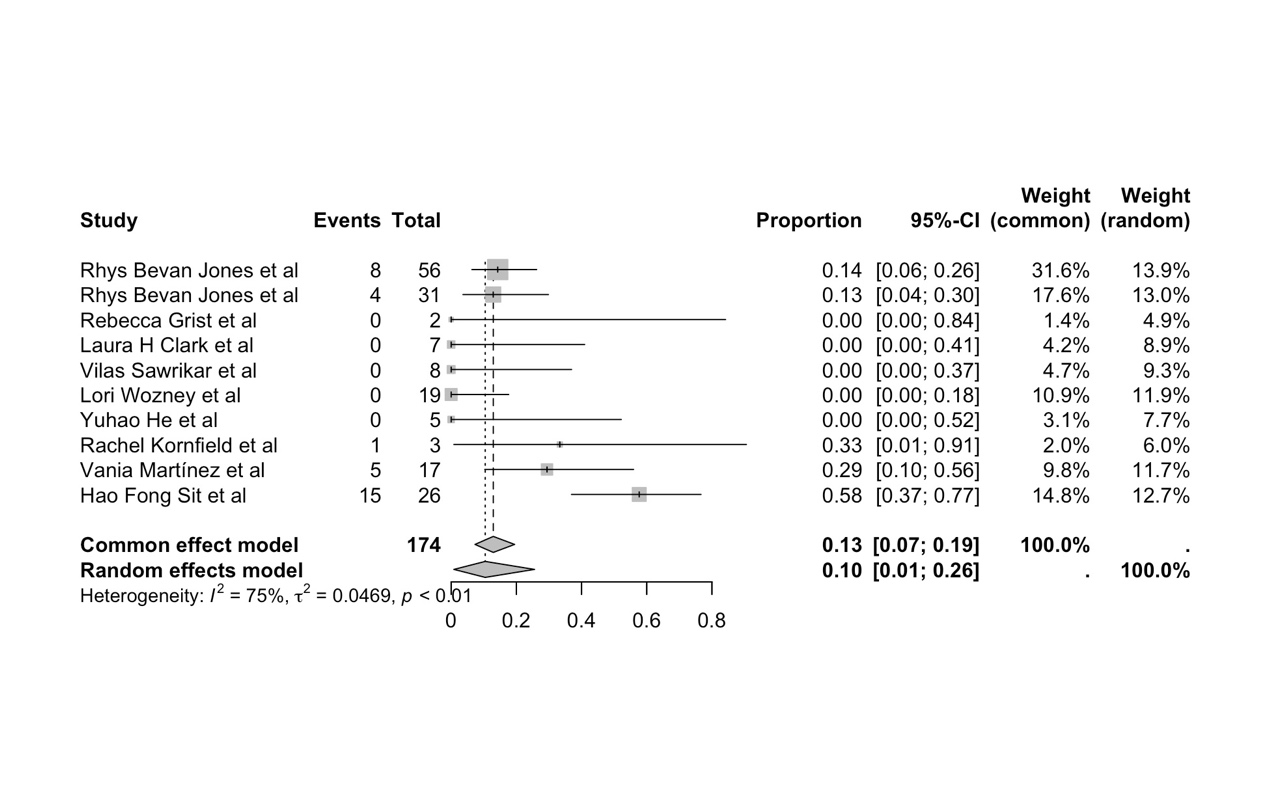


Figure 7e. Proportion of Content Engagement (F6) in MP group


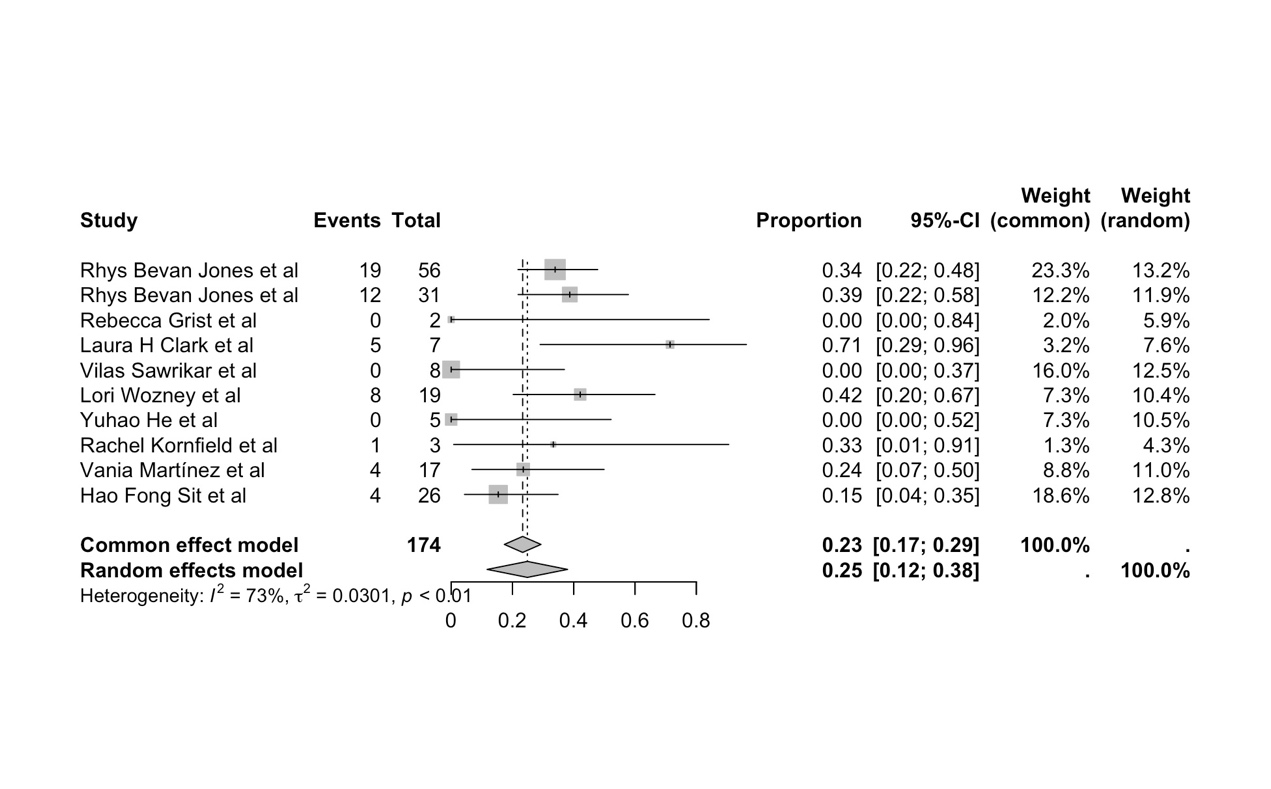


Figure 7f. Proportion of Design Harmony (F7) in MP group


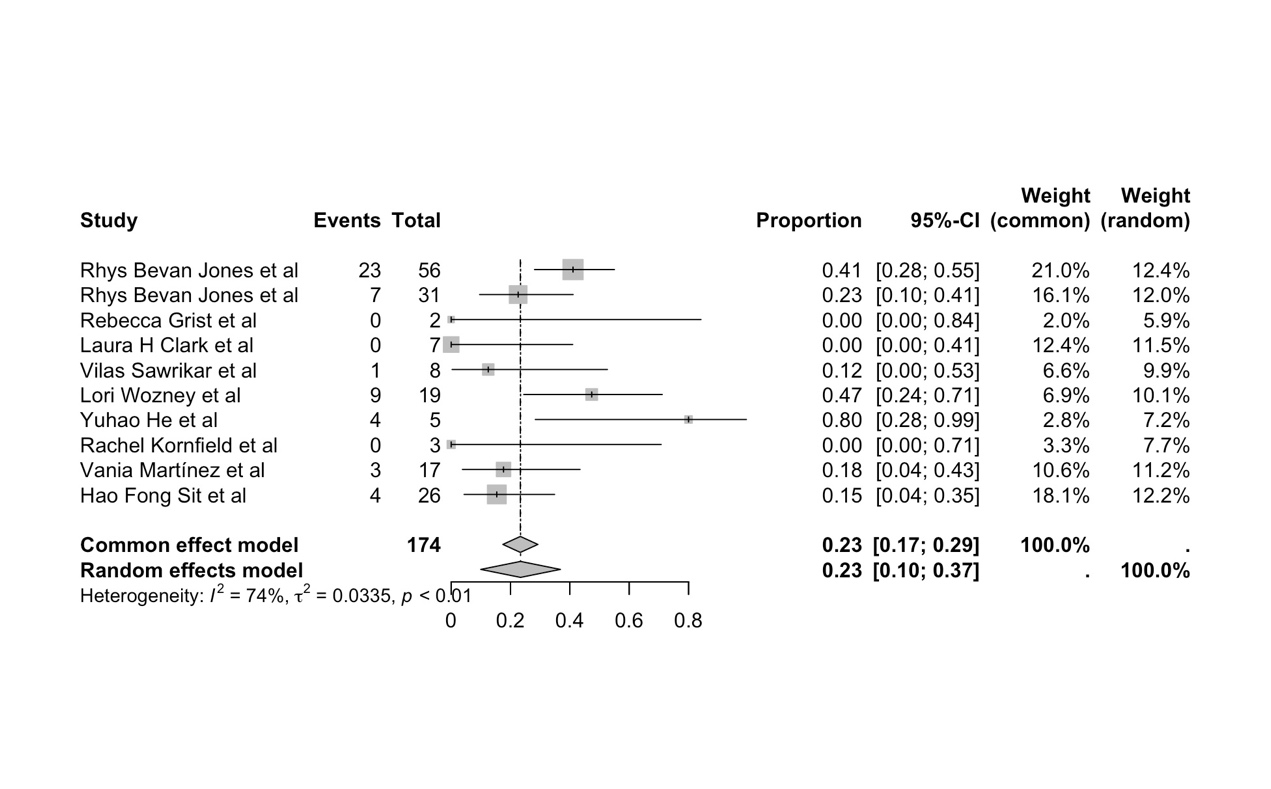


Figure 7g. Proportion of High Quality and Effect (F8) in MP group


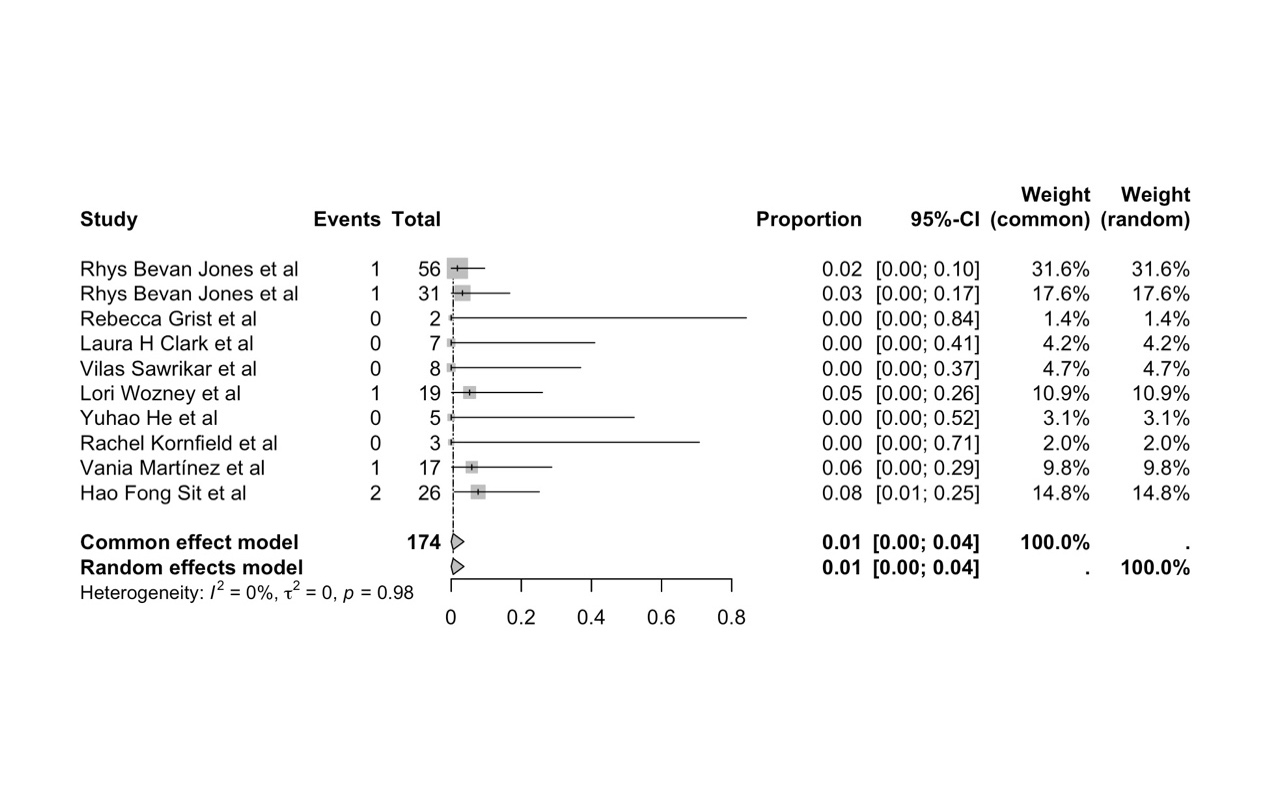


Figure 7h. Proportion of Appropriate Duration and Schedule (F9) in MP group


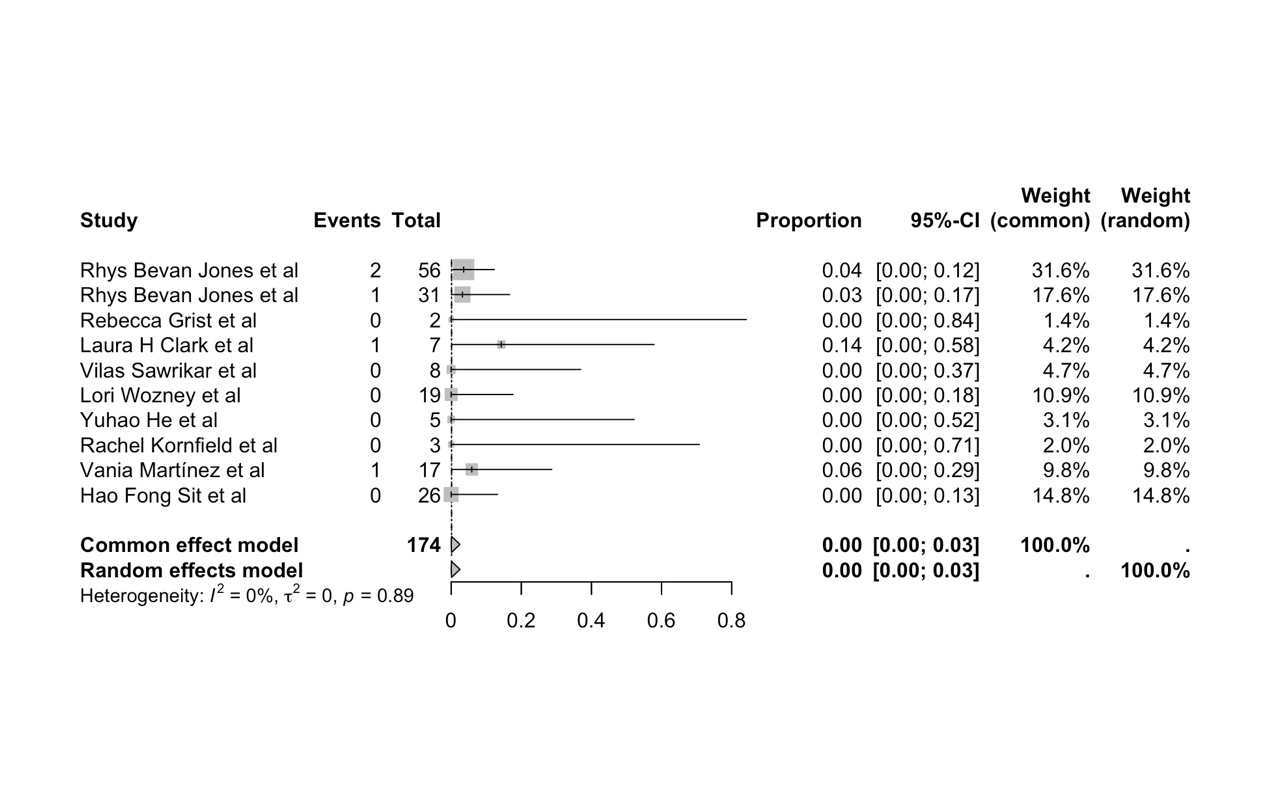


Figure 7i. Proportion of Accessibility (F10) in MP group


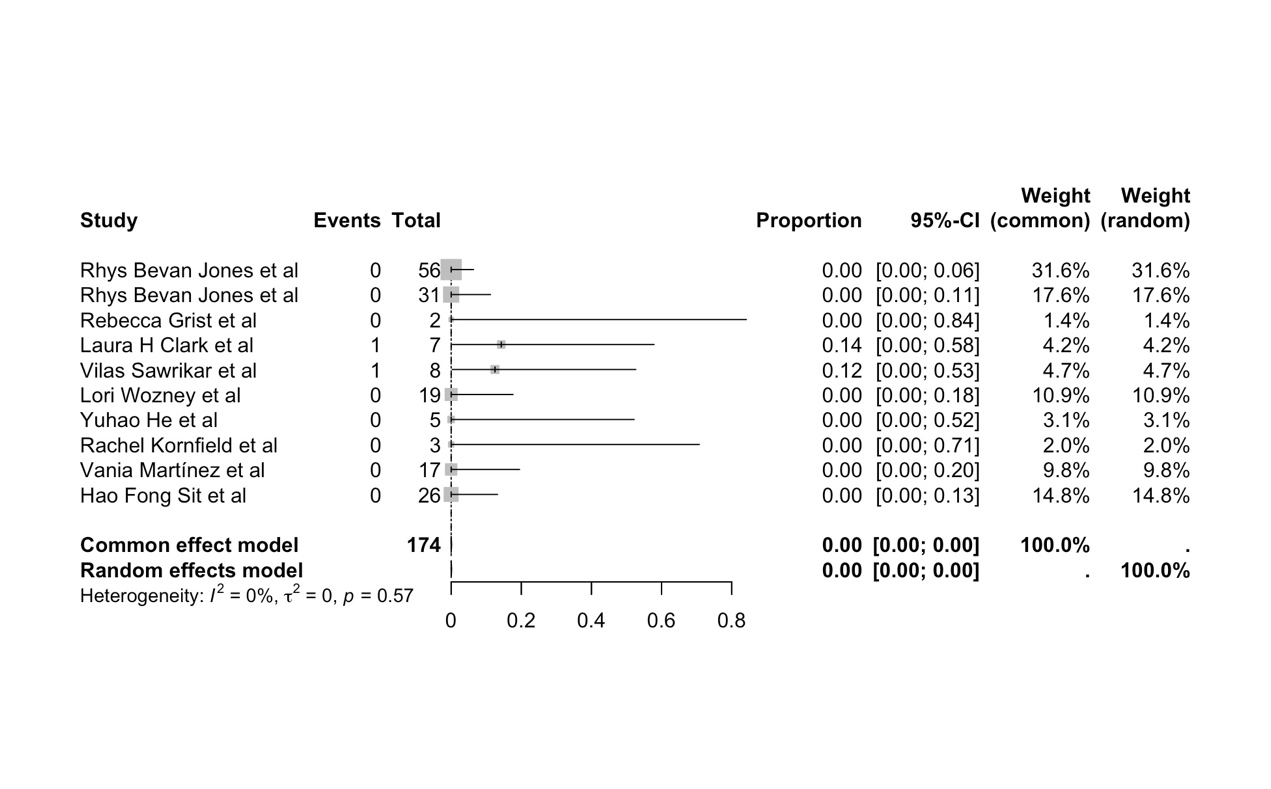


Figure 7j. Proportion of Beneficial Characteristics (F11) in MP group


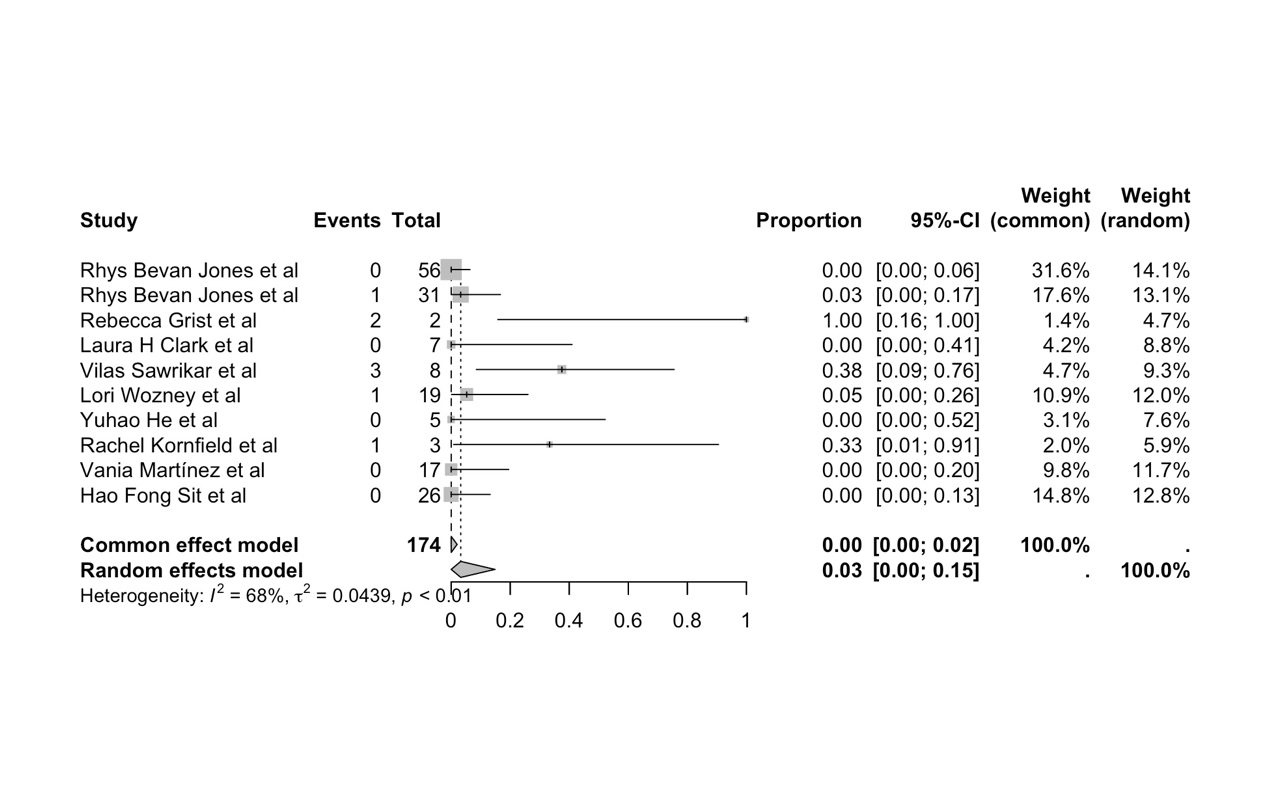


Figure 7k. Proportion of Needs and Disposition (F12) in MP group


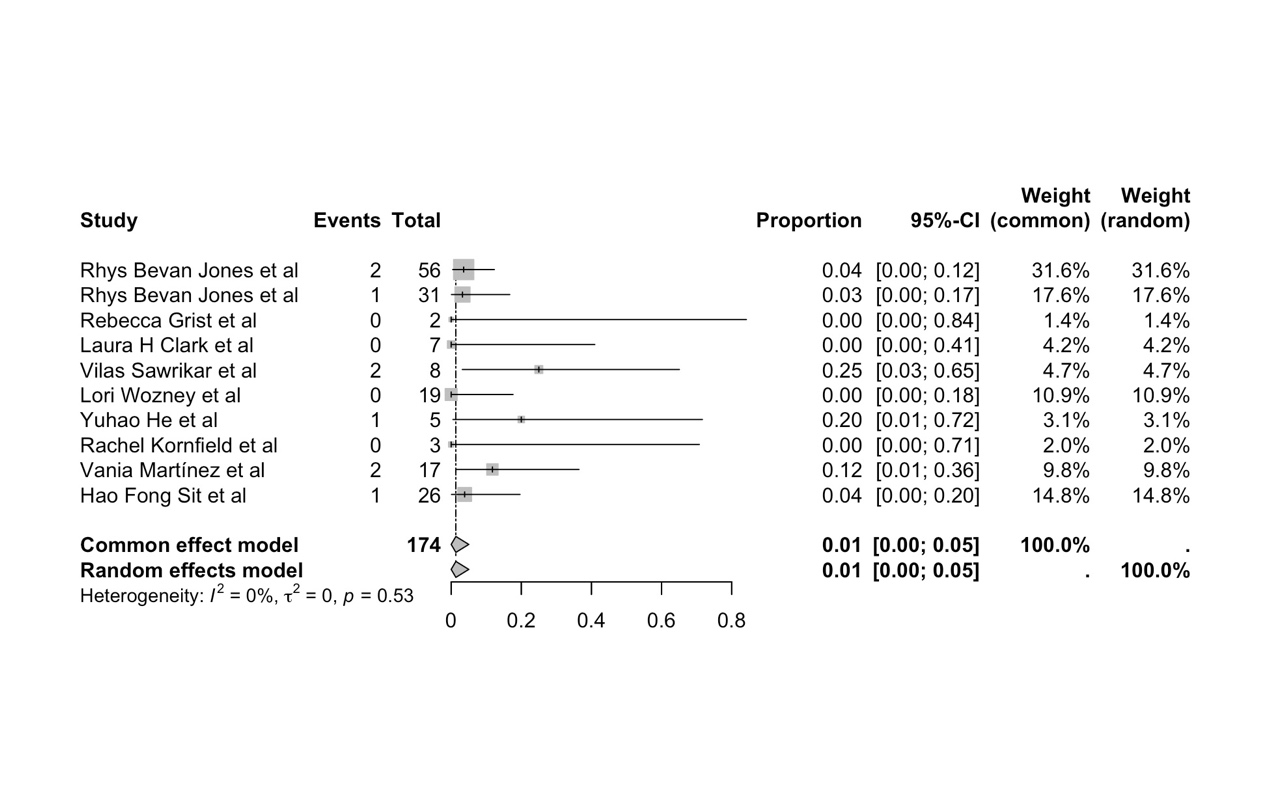


Figure 7l. Proportion of Perceived Benefits (F13) in MP group


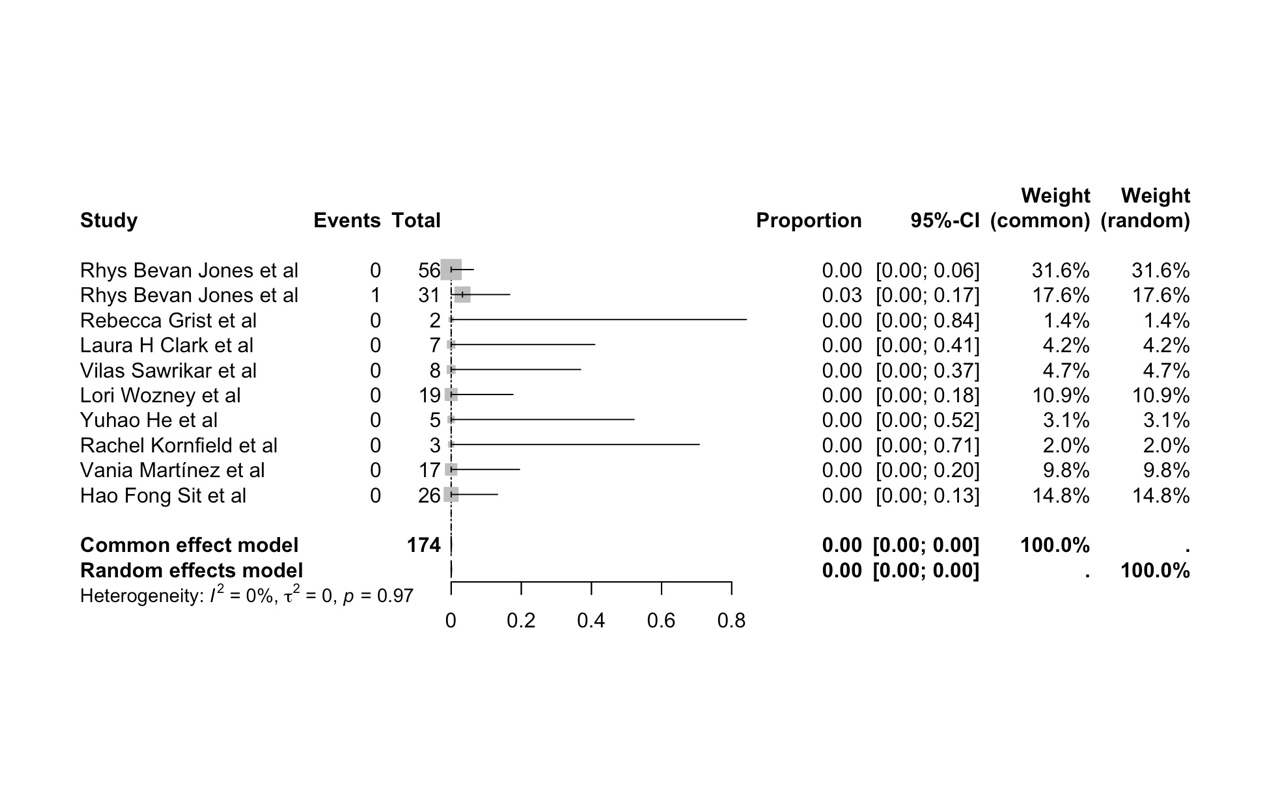


Figure 7m. Proportion of Supportive Environment (F14) in MP group

Multiple Platforms (MP)

Barriers


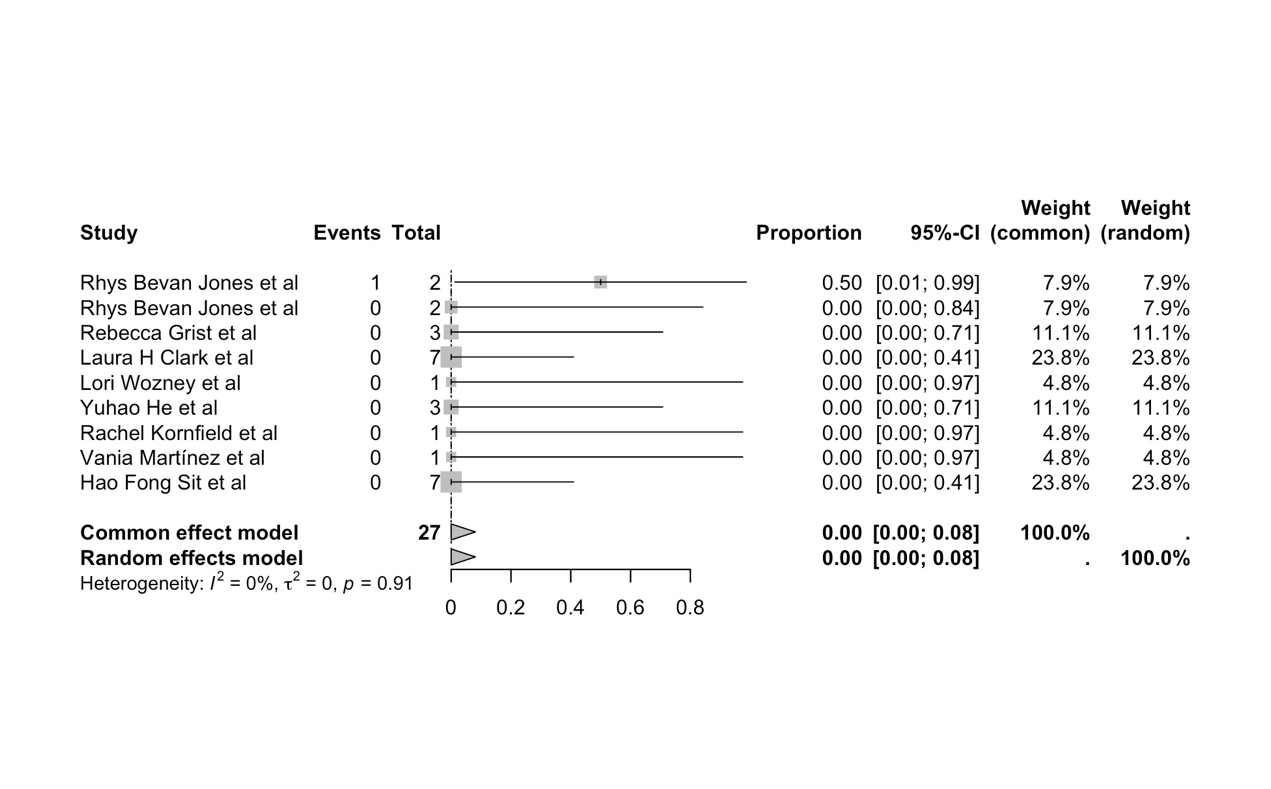


Figure 8a. Proportion of Integration with Schools (B1) in MP group


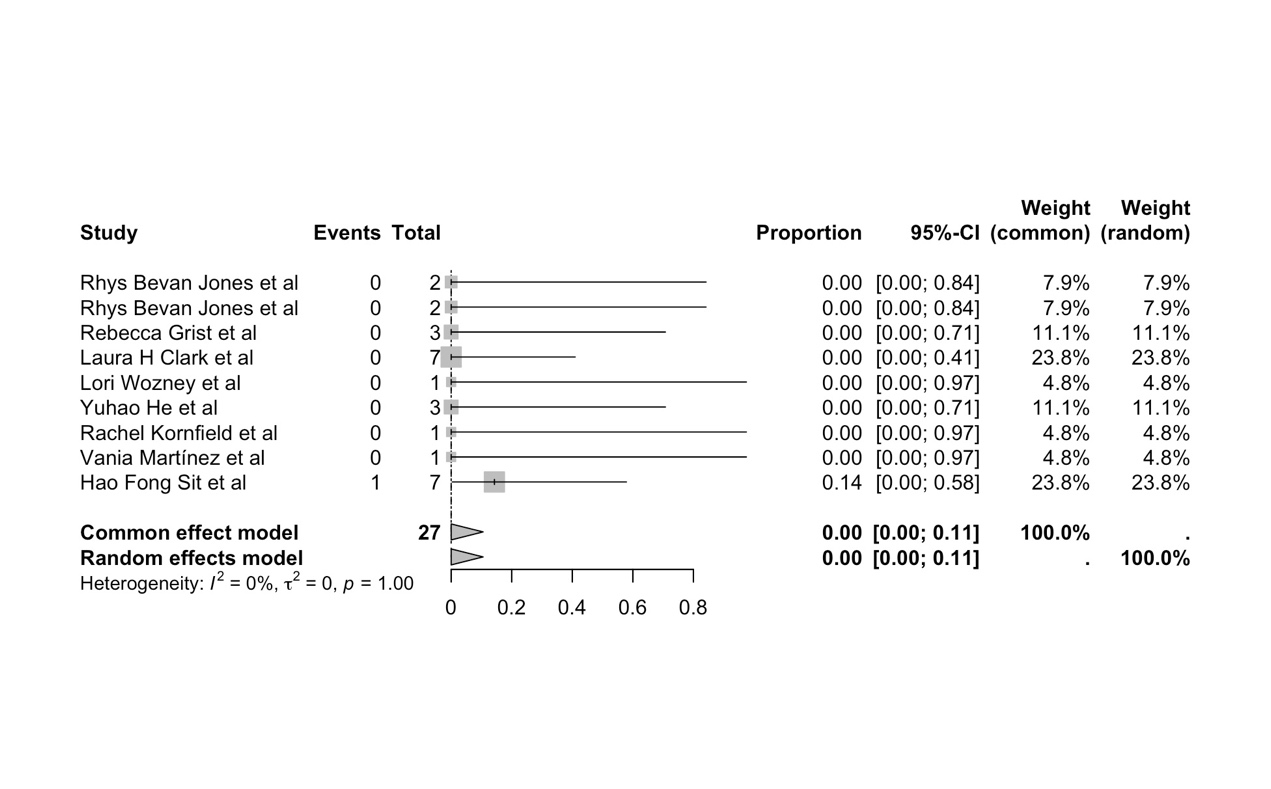


Figure 8b. Proportion of Content Gaps (B2) in MP group


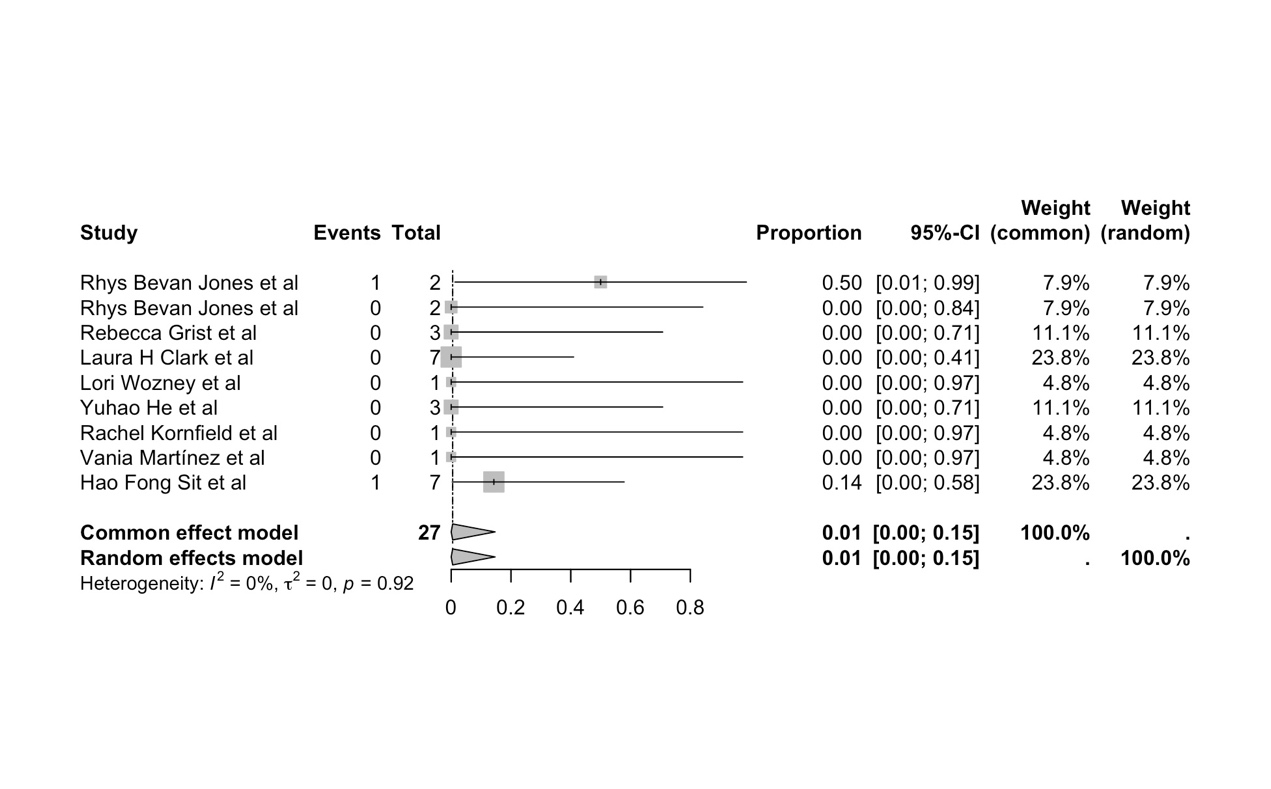


Figure 8c. Proportion of Design Limitations (B3) in MP group


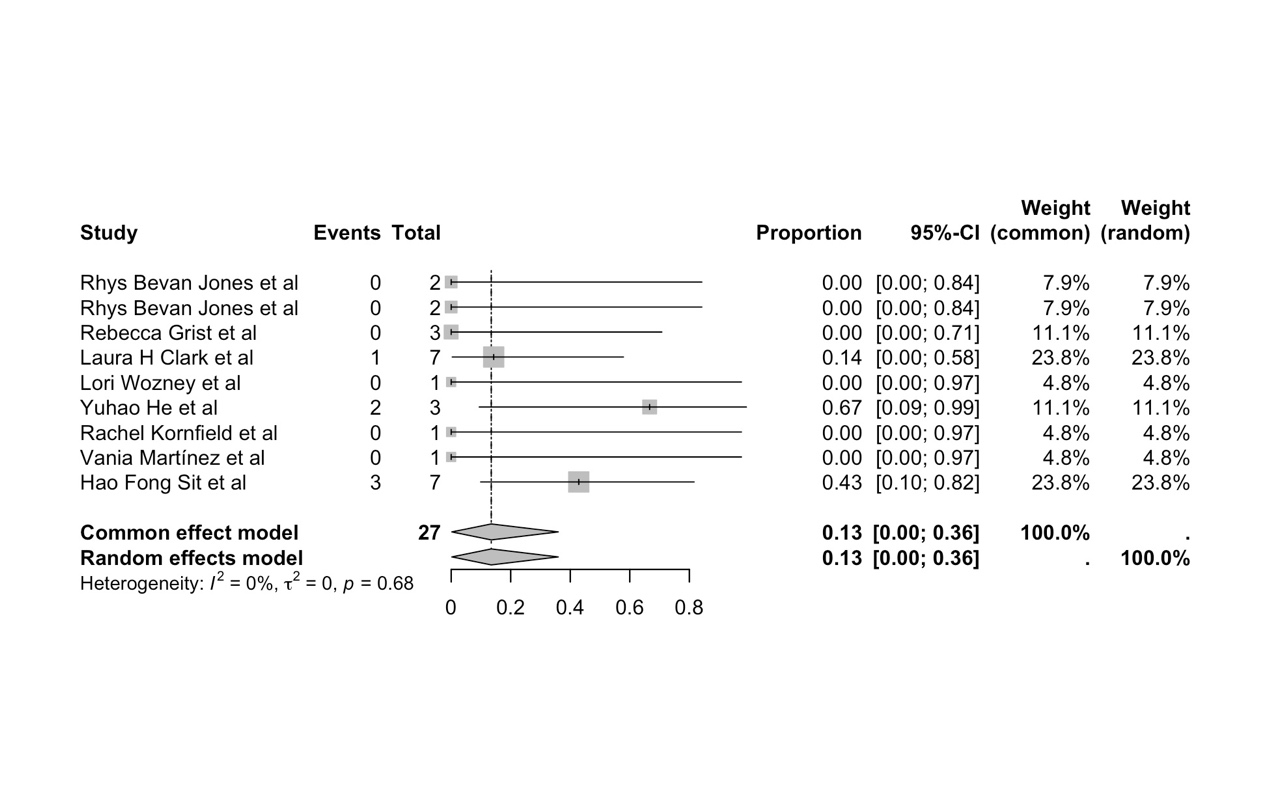


Figure 8d. Proportion of Low Quality and Effect (B4) in MP group


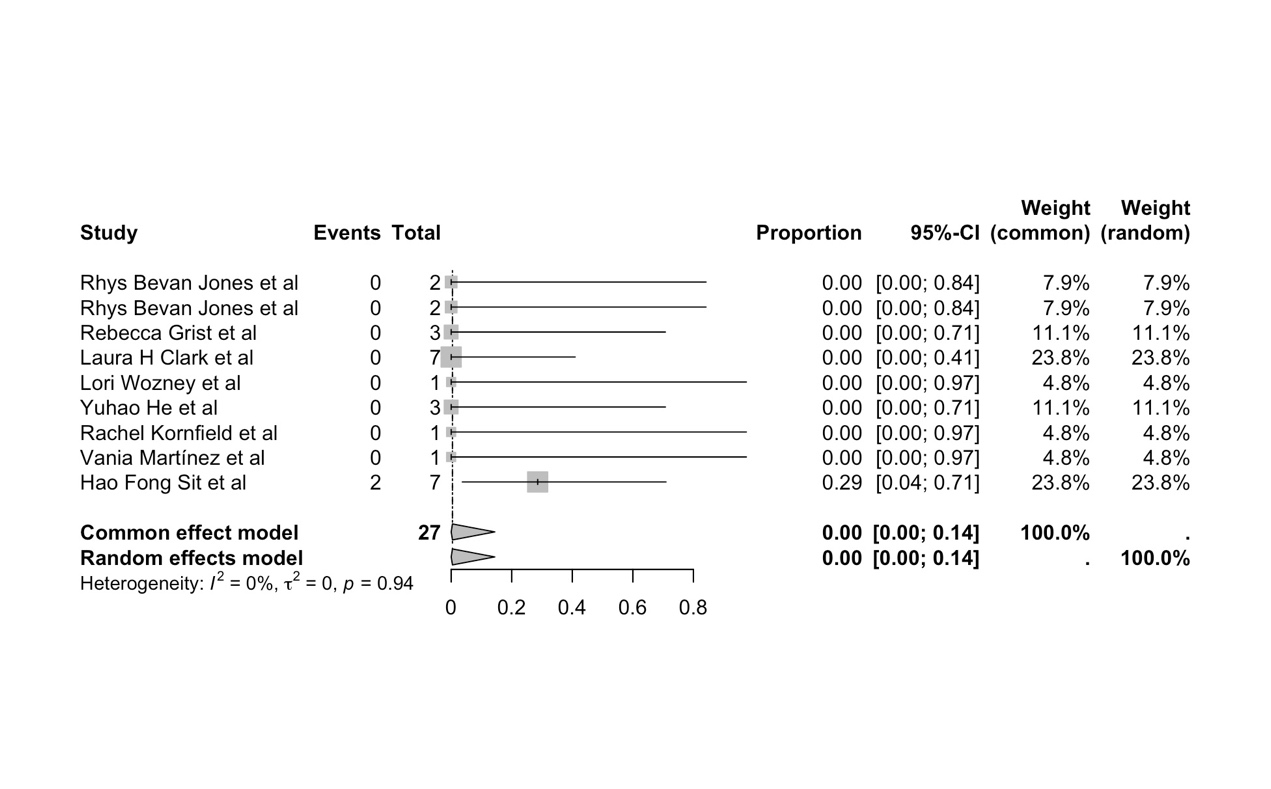


Figure 8e. Proportion of Inappropriate Duration and Schedule (B5) in MP group


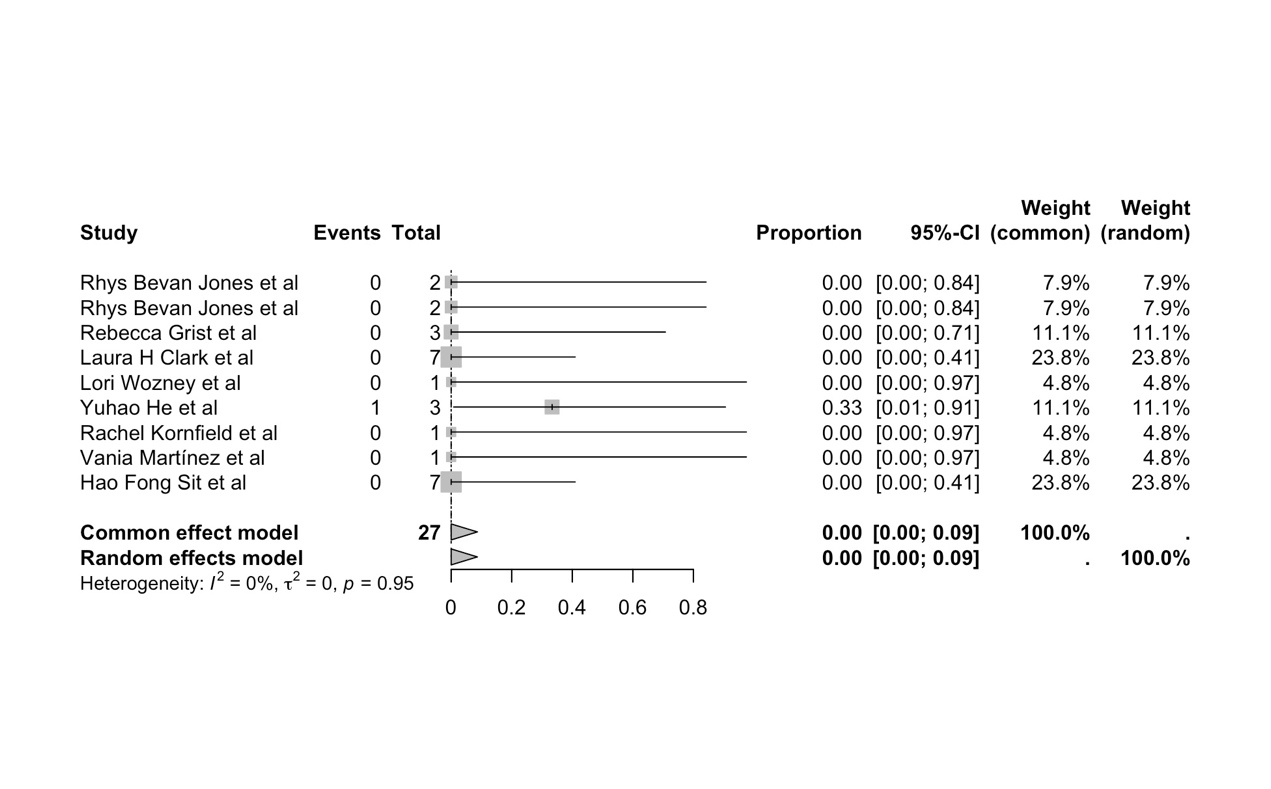


Figure 8f. Proportion of Inaccessibility (B6) in MP group


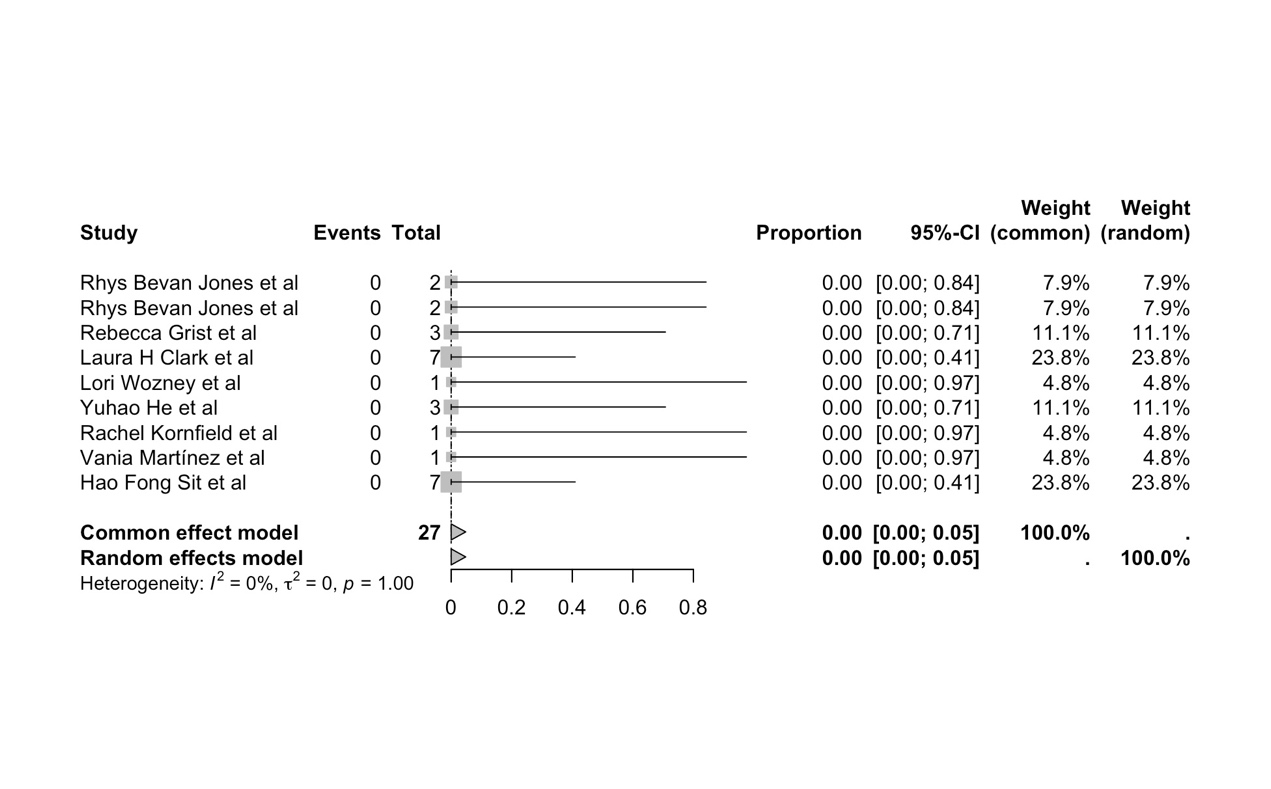


Figure 8g. Proportion of Detrimental Characteristics (B7) in MP group


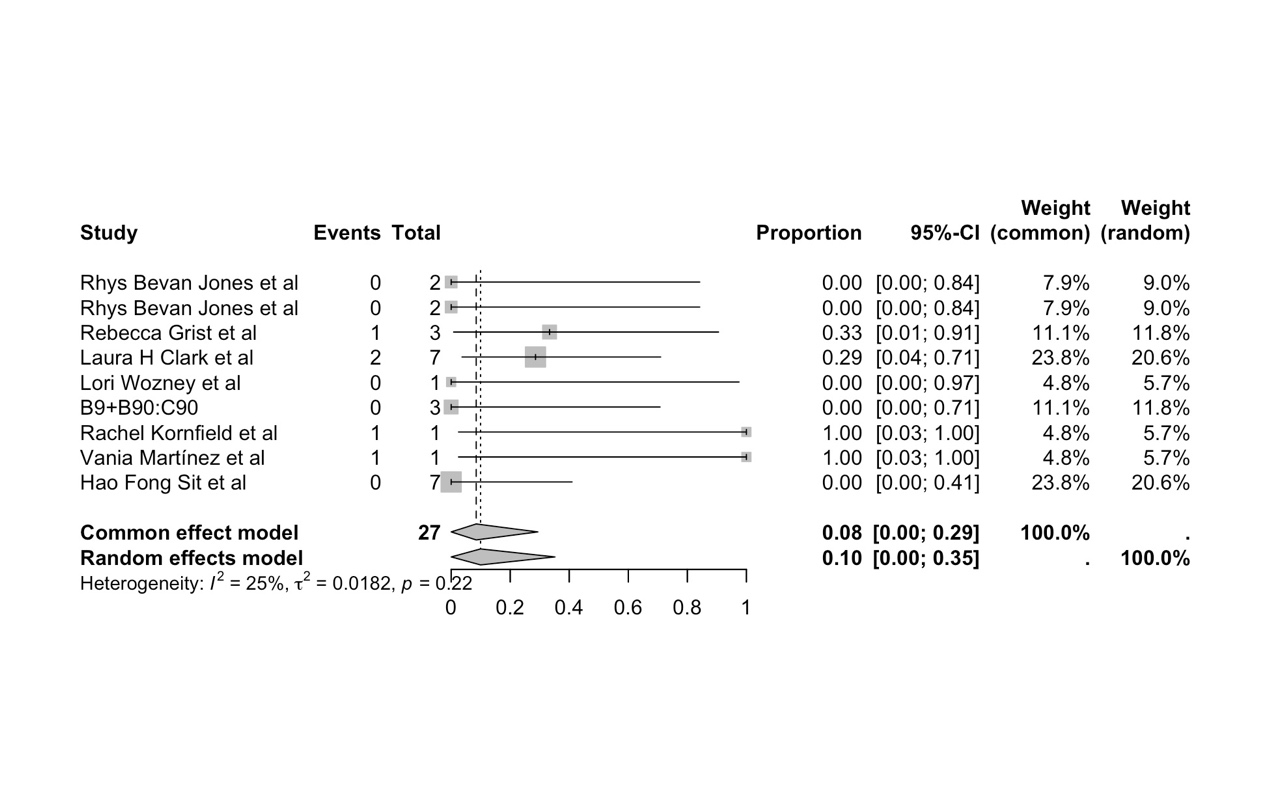


Figure 8h. Proportion of Motivational Challenges (B8) in MP group


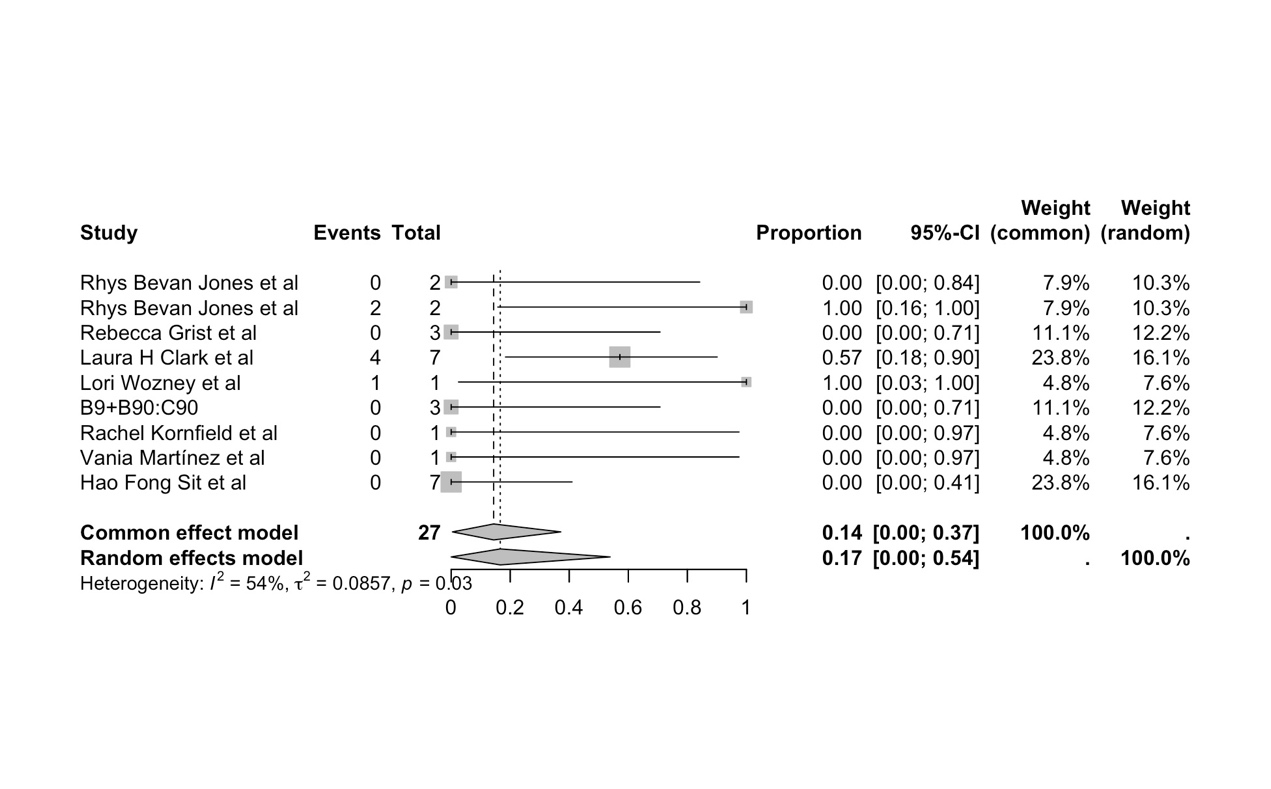


Figure 8i. Proportion of Perceived Risks (B9) in MP group


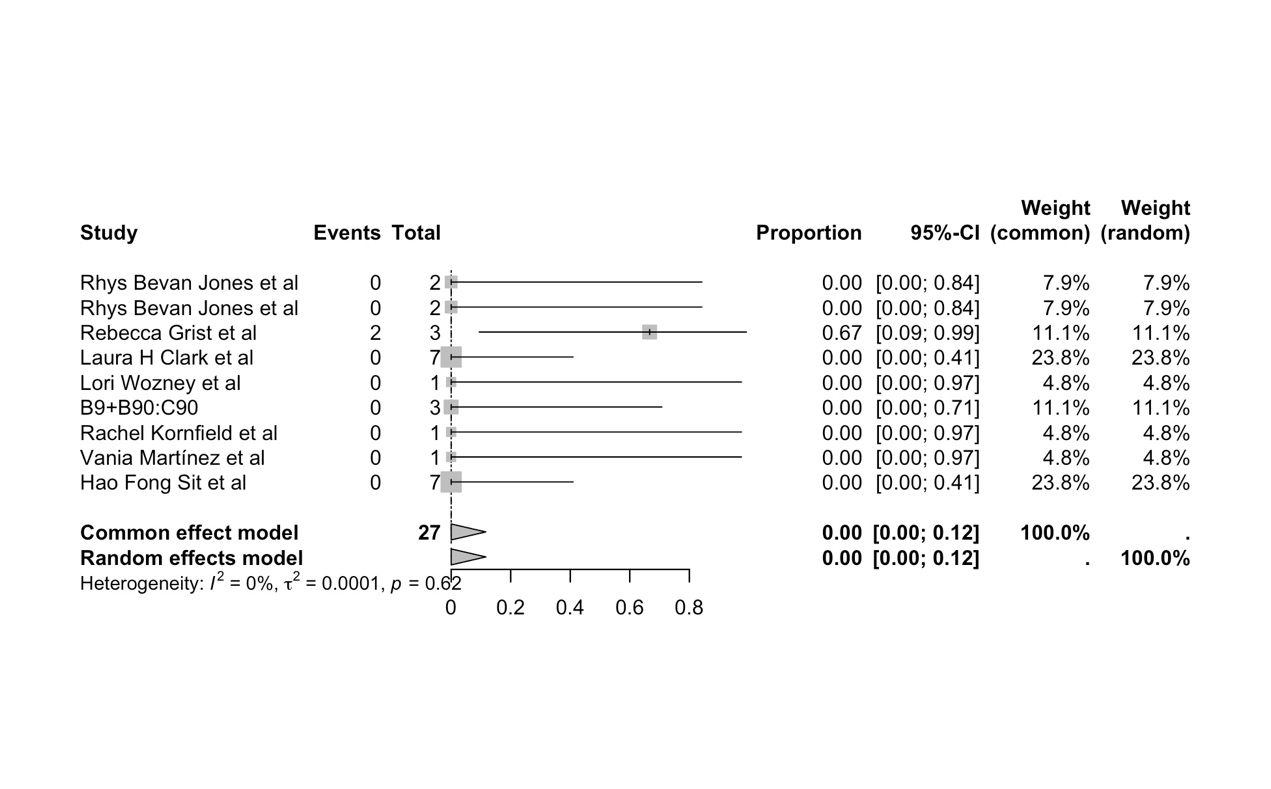


Figure 8j. Proportion of Question (B10) in MP group


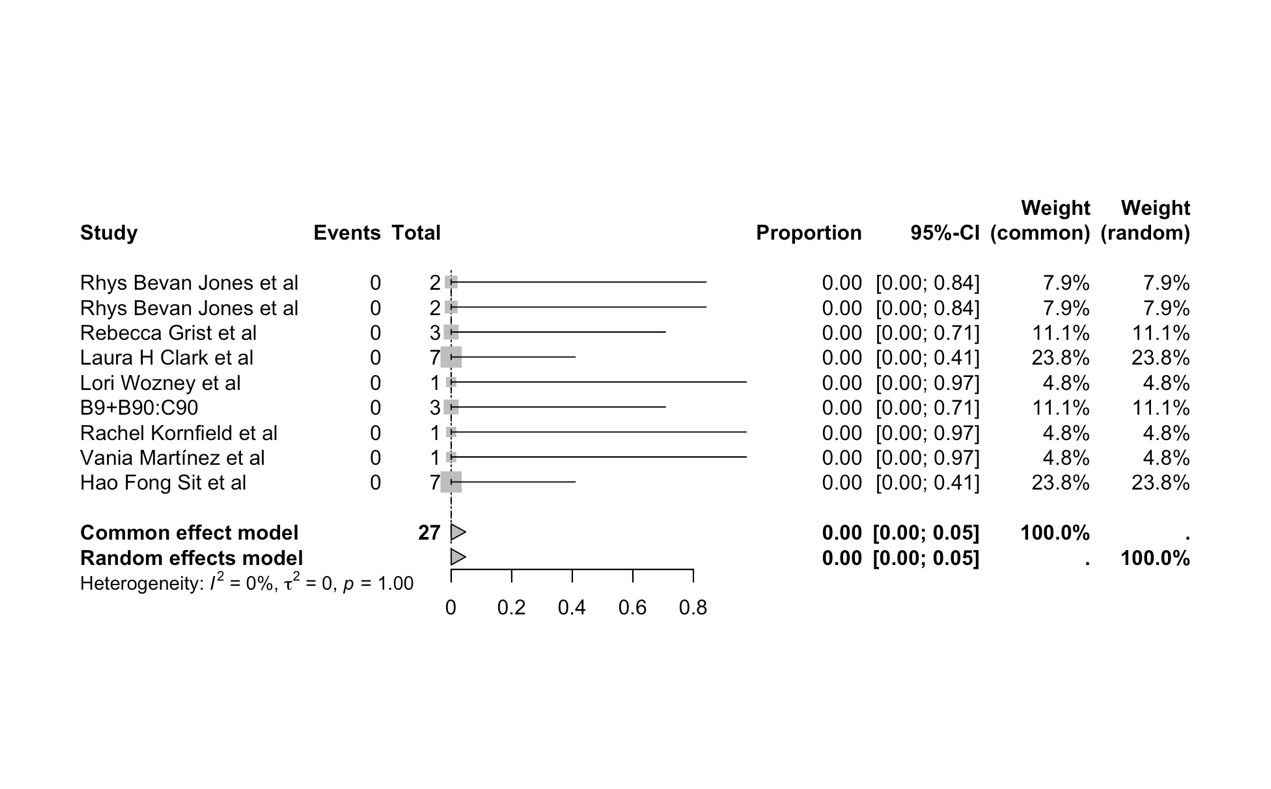


Figure 8k. Proportion of Retention Issues (B11), No/Limited Time (B12), Technical Issues (B13) in MP group
